# Design, Synthesis and Structure-Activity Relationship Studies of Meridianin Derivatives as Novel JAK/STAT3 Signaling Inhibitors

**DOI:** 10.3390/ijms23042199

**Published:** 2022-02-16

**Authors:** Jian-Qiang Zhang, Rui Li, Xue-Yang Dong, Na He, Rui-Juan Yin, Meng-Ke Yang, Jie-Yu Liu, Ri-Lei Yu, Chen-Yang Zhao, Tao Jiang

**Affiliations:** 1 Key Laboratory of Marine Drugs, Ministry of Education, School of Medicine and Pharmacy, Ocean University of China, Qingdao 266003, China; jqzzhang@163.com (J.-Q.Z.); lirui901220@126.com (R.L.); dong_xueyang@163.com (X.-Y.D.); hena@stu.ouc.edu.cn (N.H.); yinruijuan@ouc.edu.cn (R.-J.Y.); yangmengke@stu.ouc.edu.cn (M.-K.Y.); ryu@ouc.edu.cn (R.-L.Y.); 2 Innovation Platform of Marine Drug Screening & Evaluation, Qingdao National Laboratory for Marine Science and Technology, Qingdao 266100, China; ljy1401613736@163.com; 3 Laboratory for Marine Drugs and Bioproducts, Qingdao National Laboratory for Marine Science and Technology, Qingdao 266237, China

**Keywords:** JAK/STAT3 signaling pathway, meridianin derivatives, isothiouronium, antitumor activity

## Abstract

Hyperactivation of Janus kinase (JAK)/signal transducer and activator of transcription 3 (STAT3) signaling is an attractive therapeutic target for tumor therapy. Herein, forty-eight novel meridianin derivatives were designed and synthesized, and their antitumor activity was evaluated in vitro both for activity optimization and structure–activity relationship (SAR) study. The results indicated that most derivatives exhibited significantly improved antitumor activity, especially for compound **6e**. The compound **6e** contains an isothiouronium linked by an alkyl chain consisting of six carbon atoms with IC_50_ ranging from 1.11 to 2.80 μM on various cancer cell lines. Consistently, the **6e** dose dependently induced the apoptosis of A549 and DU145 cells, in which STAT3 is constitutively active. Western blotting assays indicated that the phosphorylation levels of JAK1, JAK2 and STAT3 were inhibited by **6e** at 5 μM without significant change in the total STAT3 level. Moreover, **6e** also suppressed the expression of STAT3 downstream genes, including c-Myc, Cyclin D1 and Bcl-XL at 10 μM. An additional in vivo study revealed that **6e** at the dose of 10 mg/kg could potently inhibit the DU145 xenograft tumor without obvious body weight loss. These results clearly indicate that **6e** could be a potential antitumor agent by targeting the JAK/STAT3 signaling pathway.

## 1. Introduction

The Janus kinase (JAK) and Signal transducer and activator of transcription (STAT) signaling pathway is essential in the regulation of various biological processes, including immune responses, cell division, hematopoiesis and tumor formation [1,2,3,4]. Various cytokines and growth factors transmit signals through the JAK/STAT signaling pathway, which consists of tyrosine kinase-associated receptors, JAKs and downstream transcription factor STATs [5,6]. After stimulated by cytokines, such as type I and type II interferons or IL-6, the JAKs phosphorylate each other at tyrosine residues and then phosphorylate and activate STAT proteins, which themselves dimerize and translocate to the nucleus, where they regulate gene transcription. Aberrant activation of the JAK/STAT signaling pathway has been closely associated with many diseases. Four members of the JAK family have been identified in mammals, including JAK1, JAK2, JAK3 and TYK2. The mammalian STAT family has seven members, comprising STAT1–4, STAT5(a/b) and STAT6 [1]. Among them, STAT3 is the most well-studied and is broadly hyperactivated in a variety of cancers and closely associated with tumor cell proliferation and metastasis [7,8,9]. Therefore, the JAK/STAT signaling pathway, especially STAT3 signaling, has emerged as a promising drug target for cancer treatment strategies.

Meridianins A–G (Figure S1) are marine-derived indole alkaloids isolated from the South Atlantic tunicate Aplidium meridianum, which have been demonstrated to exhibit a number of biological activities, such as antitumor activity, protein kinase inhibitory activity in a low micromolar range, antimalarial activity, antituberculosis activity and anti-Alzheimer’s disease activity [10,11,12,13,14,15]. In particular, they exhibit potent activity against the Clks and Dyrk kinase *families*, which are emerging as medicinally relevant targets involved in cancer and Alzheimer’s disease, respectively [16]. Furthermore, Meijer and coworkers found that the azaindole analogs of the meridianins, referred to as meriolins, are potent CDK9 inhibitors [17,18]. This potent activity, coupled with their antiproliferative properties, has established meriolins as significant new leads for cancer therapeutics. Due to the promising biological activities and unique chemical structures, meridianins as the lead compounds have attracted a great deal of interest in medicinal chemistry [11,16,19].

These promising results led us to expand our efforts in the synthesis of new diversely substituted meridianin derivatives. Herein, forty-eight novel meridianin derivatives were obtained in this study, and JAK/STAT3 hyperactivated human cancer cell lines were evaluated. The results indicated that meridianins (A, C, D and G) displayed weak cancer cell growth inhibition in four tested cell lines. By comparison, **1a**–**g**, **2a**–**g**, **3a**–**g** and **4a**–**g** showed significantly increased inhibitory activities but remain to be improved.

Isothiourea is a positively charged group with significant pharmacological activities, such as anesthesia, antibacterial and antitumor [19,20,21,22,23,24,25]. Our previous work showed that isothiouronium-modified analogs greatly enhanced anticancer activities, in addition to their unique Golgi localization, compared to the unmodified compounds [26].

With the aim of improving the antitumor activity, meridianins derivatives **5a**–**g** and **6a**–**g** were obtained by incorporating isothiourea groups at the N1 position with different lengths of carbon alkyl chains. The compound **6e** exhibited better antitumor activity than positive control Gefitinib. To further investigate the structure–activity relationship, the analogs of compound **6e** with different lengths of carbon alkyl chains or without isothiourea were obtained, and their antitumor activities were evaluated in the same cell lines. An additional in vivo study revealed that **6e** exhibited significant antitumor activity. Finally, the possible mechanism of **6e** was investigated preliminarily by molecular docking, Western blot, flow cytometry analysis assay and immunohistochemistry (IHC) analyses.

## 2. Results and Discussion

### 2.1. Chemistry

**Scheme 1:**
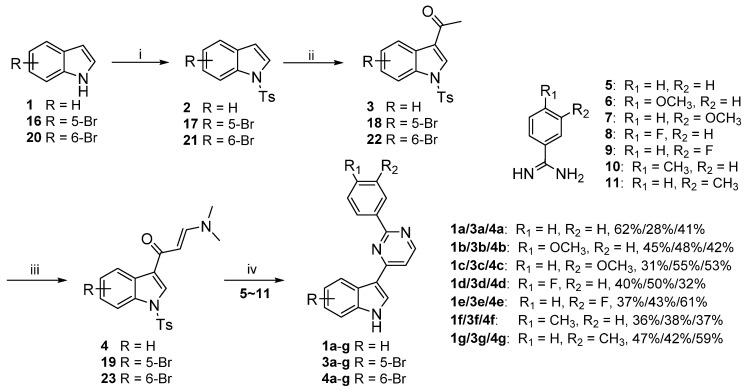
Synthesis of the final target compounds **1a**–**g**, **3a**–**g** and **4a**–**g**. Reagents and conditions: (i) TsCl, NaOH, CH_3_CN, rt, 4 h; (ii) Ac_2_O, AlCl_3_, DCM, rt, 3 h; (iii) DMF-DMA, DMF, 110 °C, 3 h; (iv) K_2_CO_3_, 2-methoxyethanol, 120 °C, 12 h.

**Scheme 2:**
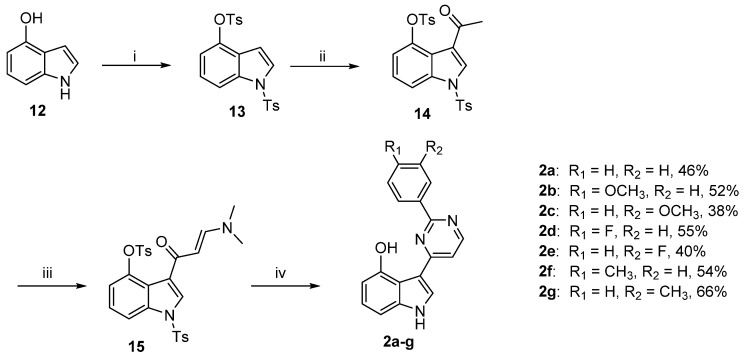
Synthesis of the final target compounds **2a**–**g**. Reagents and conditions: (i) TsCl, NaH, DMF, 0 °C, 2 h; (ii) Ac_2_O, AlCl_3_, DCM, rt, 3 h; (iii) DMF-DMA, DMF, 110 °C, 3 h; (iv) **5**–**11**, K_2_CO_3_, 2-methoxyethanol, 120 °C, 12 h.

**Scheme 3:**
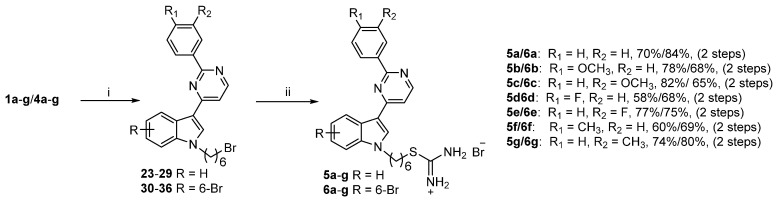
Synthesis of the final target compounds **5a**–**g** and **6a**–**g**. Reagents and conditions: (i) 1,6-dibromohexane, K_2_CO_3_, DMF, 50 °C 12 h; (ii) thiourea, isopropanol, 80 °C, 14 h.

**Scheme 4:**
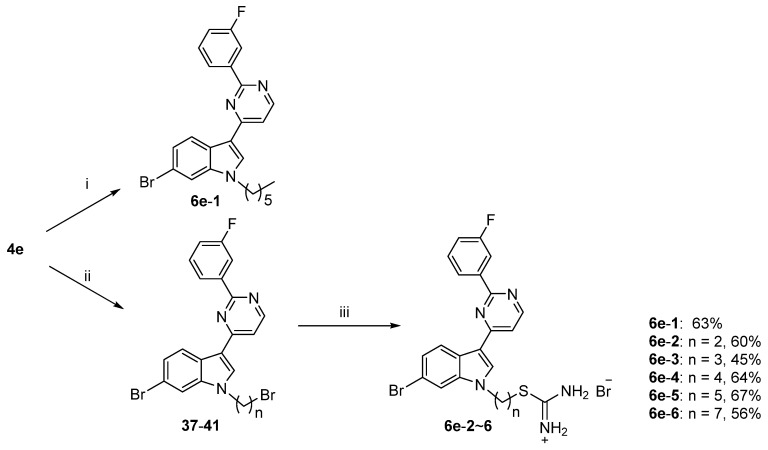
Synthesis of the final target compounds **6e-1**–**6**. Reagents and conditions: (i) 1-bromohexane, K_2_CO_3_, DMF, 50 °C 12 h; (ii) dibromoalkyl chain, K_2_CO_3_, DMF, 50 °C 12 h; (iii) thiourea, isopropanol, 80 °C, 14 h.

The reaction sequence employed to synthesize the target compounds is outlined in Scheme 1, Scheme 2, Scheme 3 and Scheme 4.

The marine natural products indole alkaloids (meridianin A, C, D and G) were synthesized in four steps starting from commercially available indoles [27]. Firstly, the indolic nitrogen was protected by the reaction with tosyl chloride in the presence of NaOH and in acetonitrile, leading to the formation of compounds **2**, **17** and **21** in 53–94% yields. Then, the C-3 position of indoles was acetylated using acetic anhydride and aluminum chloride inmethylene chloride to give derivatives **3**, **18** and **22** in 73–79% yields. The enaminone intermediates proceeded with DMF/dimethylformamide-dimethylacetal (DMF-DMA) in 69–79% yield. Finally, compounds **1a**–**g**, **3a**–**g** and **4a**–**g** were obtained from enaminone intermediates using **5**–**11** in 2-methoxyethanol in the presence of potassium carbonate in considerable yields (Scheme 1). As depicted in Scheme 2, the indolic nitrogen and 4-hydroxyl of compound **12** were tosyl chloride-protected to get compounds **13** in 85% yield. Then, the preparation of the corresponding **2a**–**g** derivatives was undertaken using a similar synthetic pathway in 38–66% yields. As shown in Scheme 3 and Scheme 4, isothiouronium derivatives **5a**–**g**, **6a**–**g** and **6e-2**–**6** were synthesized in medium yield by introducing 1,6-dibromohexane or a dibromoalkyl chain with different carbon chains without any purification and then boiling isopropanol in the presence of thiourea. In addition, the compound **6e-1** was prepared by using introducing 1-bromohexane in 63% yield.

### 2.2. Biological Activity Assessments

#### 2.2.1. Cell Viability Assay and SAR Analysis

To evaluate the antitumor activities of meridianin A, C, D, G and their derivatives, four JAK/STAT3 overactivated human cancer cell lines: HeLa, MDA-MB-231, A549 and DU145 were examined. Initially, the meridianins (A, C, D and G) and their derivatives **1a**–**g**, **2a**–**g**, **3a**–**g** and **4a**–**g** were prepared and evaluated for cell growth inhibitory activities against the cancer cell lines (Table 1). The results indicated that meridianins A, C, D and G displayed weak cancer cell growth inhibition in the four tested cell lines. By comparison, meridianin derivatives **1a**–**g**, **2a**–**g**, **3a**–**g** and **4a**–**g** showed significantly increased inhibitory activities. With the aim of improving the antitumor activity, compounds **5a**–**g** and **6a**–**g** were obtained by incorporating isothiourea groups at the C1 position of meridianins D and G with different lengths of carbon alkyl chains. Notably, the antitumor effects of the compounds were significantly enhanced, almost all compounds had a IC_50_ less than 10 μM (Table 2). Among them, the most potent compound **6e** inhibited the growth of HeLa, MDA-MB-231, A549 and DU145 cells with IC_50_ values of 1.11, 1.22, 2.80 and 1.13 μM, which exhibited better activity than the positive control (Gefitinib), respectively. Based on the above results, the analogs of compound **6e** with different carbon chain lengths or without isothiourea were obtained, and the antitumor activity results are shown in Table 3. The structure–activity relationship suggests that reducing or increasing the number of carbon atoms will lead to decreased antiproliferative activity. On the other hand, compound **6e-1** was found to be completely inactive with IC_50_ values greater than 100 μM, indicating that the substitution of the isothiourea group significantly contributed to the antitumor activity. The inhibitory effect of compound **6e** on the proliferation of the four normal cell lines HUVEC, L02, L929 and MCF10A was determined by the MTT assay. The data showed that **6e** had low toxicity compared to normal cells, predicting that it may be relatively safe in vivo (Table 4).

#### 2.2.2. Compound **6e** Inhibited Cancer Cell Proliferation and Induced Cell Apoptosis

**Figure 1:**
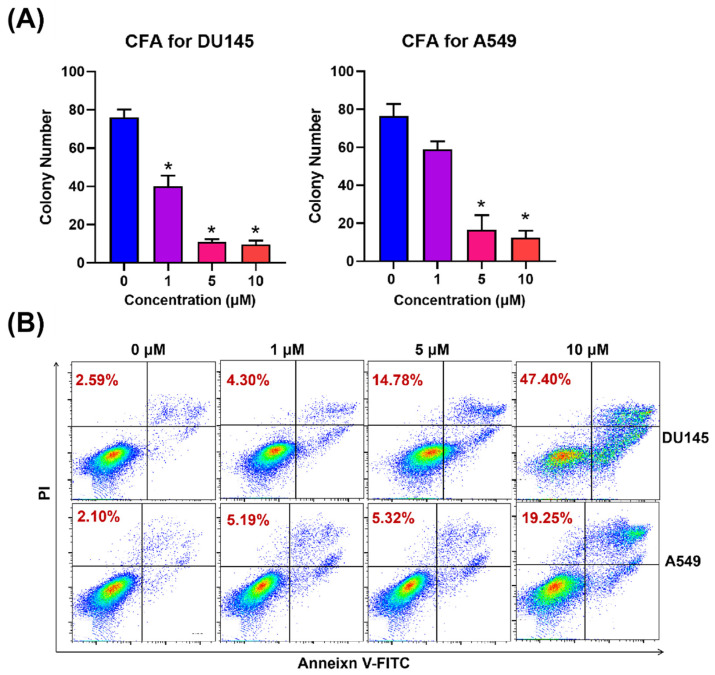
(**A**) Effect of compound **6e** on colony formation in DU145 and A549 cells. (**B**) Compound **6e** induced A549 and DU145 cancer cells apoptosis in vitro. A549 and DU145 cells were incubated with **6e** at different concentrations (0–10 μM) for 24 h. Annexin V/PI staining was carried out, and the percentage of apoptotic cells was further determined using flow cytometry. * Statistically significant (*n* = 2, *p* < 0.05).

To evaluate the antiproliferative activities of compound **6e** in the cell models, its effects on DU145 and A549 colony survival were evaluated. The results of the CFA analysis showed that **6e** significantly inhibited the proliferation of both cells, and the effect was enhanced with the increasing **6e** concentration. The effect of compound **6e** on inducing tumor cell apoptosis was analyzed in Figure 1B. A549 and DU145 cells were incubated with **6e** at different concentrations for 24 h. Annexin V-FITC/PI staining was carried out, and the percentage of apoptotic cells was further determined using flow cytometry. The results showed that the **6e** dose dependently induced the apoptosis of the A549 and DU145 cells. As shown in Figure 1B, in A549, the induced apoptosis rates at 0, 1, 5 and 10 μM were 2.59%, 4.30%, 14.78% and 45.40%, respectively. In DU145, the induced apoptosis rates at 0, 1, 5 and 10 μM were 2.10%, 5.19%, 5.32% and 19.25%, respectively.

#### 2.2.3. Molecular Docking

**Figure 2:**
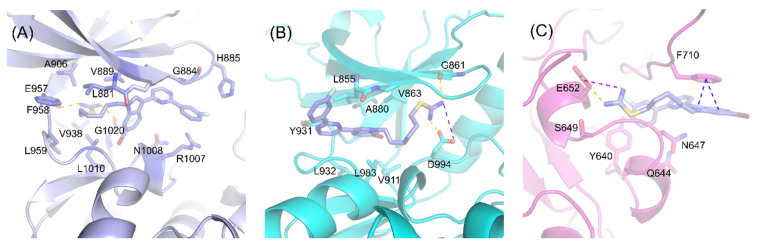
Binding modes of the compound **6e** with JAK1, JAK2 and STAT3. The compound **6e** is shown in blue, while the JAK1 (**A**), JAK2 (**B**) and STAT3 (**C**) are shown in purple, blue and pink, respectively. The H-bonds, π–π and salt bridges were shown in dashed lines.

Molecular docking was performed for understanding the interaction mechanisms between compound **6e** with JAK1 (PDB ID:4I5C), JAK2 (PDB ID:5CF5) and STAT3 (PDB ID:1BG1) [28], respectively. For JAK1, the results showed that the hydrophobic fatty chain was accommodated at a hydrophobic pocket mainly defined by residues Leu881, Val889, Ala906, Val938, Phe958, Leu959 and Leu1010 (Figure 2A). The hydrogen in the imine of **6e** engages H-bond formation with Glu957 and Gly1020. For JAK2, the docking poses suggested that the imine group of **6e** interacts with a carboxyl group of Asp994 and carbonyl group of Gly861 by forming two hydrogen bonds (Figure 2B). In addition, the nitrogen of imine on **6e** also forms a salt bridge interaction with Asp994. While, for STAT3, Phe710 forms π–π interactions with the indole ring of **6e**, Glu652 forms a hydrogen bond and a salt bridge with the H atom and the N atom on imine, respectively (Figure 2C) [29].

#### 2.2.4. Compound **6e** Inhibits the Expression of JAK/STAT3 Target Genes

**Figure 3:**
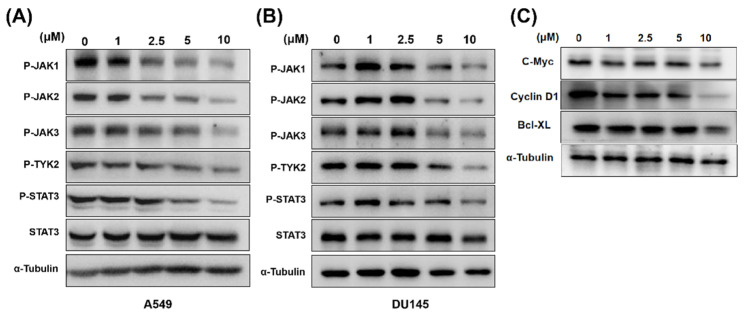
The effect of **6e** on overexpressed proteins in the JAK/STAT3 signaling pathway by Western blot analysis. (**A**) **6e** decreased levels of JAK1, JAK2 and STAT3 in A549 cells. (**B**) **6e** decreased levels of JAK1, JAK2 and STAT3 in DU145 cells. (**C**) **6e** inhibited the expression of JAK/STAT3 downstream genes c-Myc, Cy-clin D1 and Bcl-XL in DU145 cells.

The ability of compound **6e** to inhibit the phosphorylation of JAK/STAT3 was determined in A549 and DU145 cells. As shown in Figure 3A,B, after 24 h of treatment with 5-μM **6e**, the decreased levels of JAK1, JAK2 and STAT3 were observed in both A549 and DU145 cells, but no significant change was seen with the total STAT3 protein expression. Moreover, compound **6e** significantly inhibited the expression of JAK/STAT3 downstream genes c-Myc, Cyclin D1 and Bcl-XL at 10 μM after 24 h of treatment (Figure 3C). Therefore, pretreatment with compound **6e** suppressed the JAK/STAT3 signaling pathway and its downstream gene expressions, which were consistent with the above results obtained in vitro.

#### 2.2.5. Compound **6e** Inhibited Tumor Growth in a Mouse Breast Cancer Model

**Figure 4:**
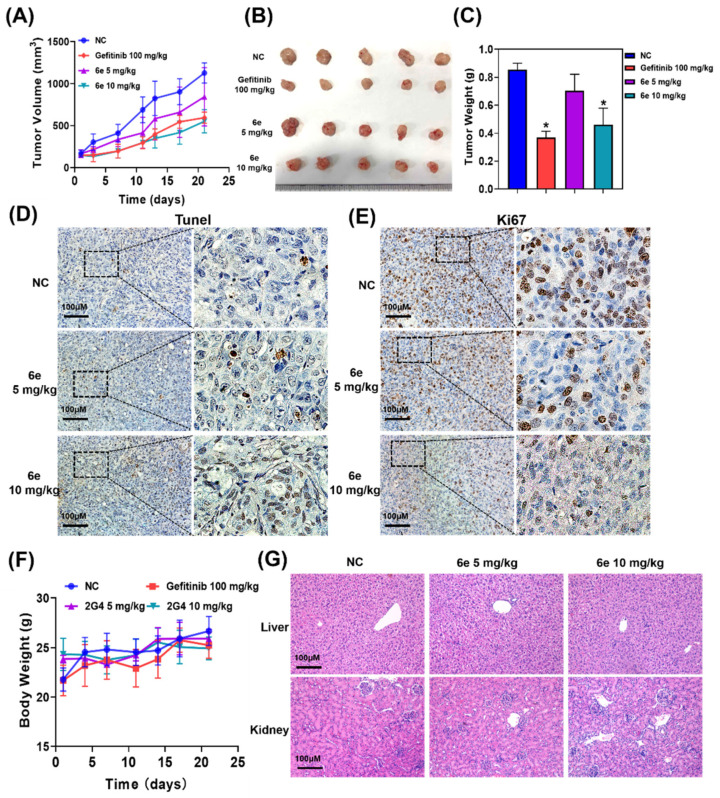
The results of **6e** on the DU145 tumor-bearing model in vivo therapeutic. (**A**) Growth curve of subcutaneous tumor-bearing DU145 treated with **6e**. (**B**) Anatomical mice tumor tissues treated with **6e**. (**C**) The weight of the tumors in each group treated with **6e**. (**D**) Ki67 and (**E**) Tunel staining in DU145 tumors treated with different doses of **6e**. (**F**) Changes in the body weights of mice in each group during administration. (**G**) H&E sections of the liver and kidneys of treated mice in each group. * Statistically significant (*n* = 5, *p* < 0.05).

To further investigate the antitumor potential in vivo, we evaluated the effects of compound **6e** in a nude mice tumor model (Figure 4). After the solid tumor was established, twenty-four nude mice were randomly divided into four groups, which were the vehicle control group, **6e** groups (5 mg/kg and 10 mg/kg) and Gefitinib-positive control group. All the compounds were taken by intragastric gavage. Compound **6e** at the dose of 10 mg/kg could significantly inhibit tumor growth, and the tumor inhibition rate of **6e** was over 40%, which was comparable to that of the positive control (Figure 4A–C). Subsequently, Ki67 and Tunel staining were performed on tumor sections, which showed that tumor proliferation marker Ki67 was significantly inhibited, and the proportion of apoptotic cells that were marked by Tunel-positive staining also significantly increased with **6e** treatment at a dose of 10 mg/kg (Figure 4D,E). Moreover, during the administration period, the weight of the nude mice did not increase or decrease significantly, indicating that there was no obvious biological toxicity of **6e** (Figure 4F). H&E staining was performed on the liver and kidneys of each group of mice to observe the hepatic and renal toxicity of **6e**. The results showed that no significant liver and kidney damage was observed in all the groups of mice, suggesting that **6e** was less toxic at therapeutic doses (Figure 4G).

#### 2.2.6. Immunohistochemical (IHC) Analysis

**Figure 5:**
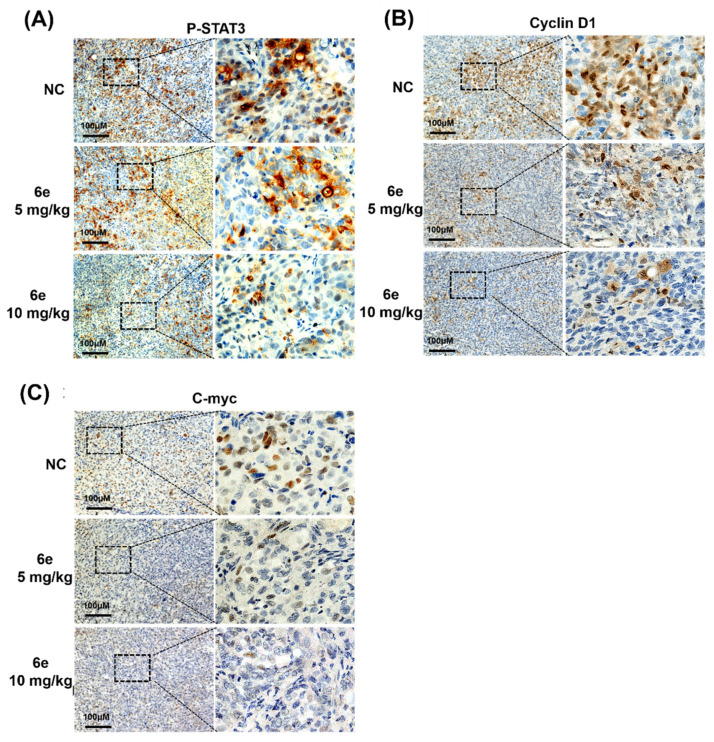
Immunohistochemistry of the protein expressions in a DU145 xenograft model. Formalin-fixed paraffin-embedded sections from DU145 xenograft tumors were stained with H&E (**A**) p-STAT3, (**B**) cyclin D1 and (**C**) c-Myc antibodies.

To further test the inhibitory effects of **6e** on JAK/STAT3 signaling, the IHC analysis of nude mice inoculated with DU145 tumor cells was performed. As shown in Figure 5, after **6e** treatment, the intratumoral staining of p-STAT3, cyclin D1 and c-Myc in DU145-inoculated mice was significantly lower than those in the NC group, and their staining levels decreased while the dose of **6e** increased. Therefore, compound **6e** may exert antitumor effects by inhibiting the JAK/STAT3 signaling pathway both in vitro and in vivo.

## 3. Conclusions

In summary, a novel series of meridianin derivatives was obtained and biologically evaluated. Initially, the meridianins (A, C, D and G) and their four series derivatives of compounds **1a**–**g**, **2a**–**g**, **3a**–**g** and **4a**–**g** were prepared, and the results indicated that the meridianins (A, C, D and G) displayed weak inhibitory activity on four JAK/STAT3 overactivated human cancer cell lines: HeLa, MDA-MB-231, A549 and DU145, whereas most of the meridianin derivatives exerted promising inhibitory activity on the tested cell lines. To improve the antitumor activity, meridianin derivatives **5a**–**g** and **6a**–**g** were designed and synthesized by incorporating isothiourea groups at the N1 position with different lengths of carbon alkyl chains. Surprisingly, the antitumor effects of the isothiouronium-modified compounds were significantly enhanced, with IC_50_ less than 10 μM. Among them, the most potent compound, **6e** with an alkyl chain of six carbon atoms, had an IC_50_ that ranged from 1.11 to 2.80 μM in various cancer cell lines, which was superior to the positive control, Gefitinib. The structure–activity relationship (SAR) study indicated that isothiouronium modified by N-alkylation with 6C alkyl chains may contribute the most to antitumor activity. It is worth noting that **6e** had low toxicity to normal cells. The Western blotting assays suggested that treatment with compound **6e** could decrease the phosphorylation level of JAK1, JAK2 and STAT3 at 5 μM but did not affect the total STAT3 level. Moreover, **6e** also suppressed the expression of STAT3 downstream genes, including c-Myc, cyclin D1 and Bcl-XL. Consistently, **6e** dose-dependently inhibited the proliferation and induced the apoptosis of A549 and DU145 cells. Molecular docking studies demonstrated that an H-bond is the main type of interaction between compound **6e** and the JAK1/JAK2 kinases, and **6e** could also tightly bind to the STAT3 SH2 domain. An additional in vivo study revealed that the application of **6e** at a dose of 10 mg/kg could significantly inhibit the DU145 xenograft tumor growth without an obvious body weight loss, which was comparable to that of the positive control. Taken together, these results clearly indicated that **6e** could be a highly potent antitumor agent by targeting the JAK/STAT3 signaling pathway. In addition, the pharmacokinetic properties of compound **6e** will be further investigated in the future.

## 4. Materials and Methods

### 4.1. Chemistry

All commercially available starting materials and solvents were purchased from commercial vendors and used without further purification. Reactions were monitored using analytical thin-layer chromatography (TLC) on precoated silica gel GF254 plates (Qingdao Haiyang Chemical Plant, Qingdao, China) and visualized under ultraviolet light (254 nm and 365 nm). Column chromatography was performed on silica gel (200–300 mesh). Melting points were determined on a Mitamura-Riken micro-hot stage and uncorrected. ^1^H and ^13^C NMR spectra were recorded on the Broker AVANCE NEO and Agilent DD2 500 with 400 or 500 MHz for proton (^1^H NMR) and 100 or 125 MHz for carbon (^13^C NMR), respectively. The chemical shifts (*δ*) were expressed in parts per million (ppm) downfield, and the coupling constant (*J*) values were described as hertz. High-resolution (ESI) MS spectra were recorded using a QTOF-2 Micromass spectrometer. The purity of the final compounds for biological evaluation was higher than 95% by analytical HPLC analysis with the Primaide 1210 system.

Compounds **1**–**4**, **16**–**19** and **20**–**23** were prepared according to the procedure published by Dong et al., and the spectroscopic data for the intermediates were identical to those described in the literature [30].

#### 4.1.1. General Procedure for the Synthesis of **1a**–**g**, **3a**–**g** and **4a**–**g** Meridianin Analogs

To the solution of intermediates **4**, **19** and **23** (1.0 equiv.) in 2-methoxyethanol (5 mL) was added **5**–**11** (2.5 equiv.) and potassium carbonate (2.0 equiv.), respectively. The reaction mixture was stirred at 120 °C for 20 h under a nitrogen atmosphere. Then, the mixture was poured into ice water and extracted with ethyl acetate (3 × 50 mL). The combined organic layers were washed with brine and dried over magnesium sulfate anhydrous. After filtration, the solvent was removed under vacuum, and the residue was purified by silica gel column chromatography (petroleum ether/ethyl acetate 10:1) to give the final target compounds **1a**–**g**, **3a**–**g** and **4a**–**g**.

3-(2-phenylpyrimidin-4-yl)-1*H*-indole (**1a**). Yield: 62%; pale yellow solid; m.p. 137–139 °C; ^1^H NMR (500 MHz, DMSO-*d_6_*): *δ* 11.94 (s, 1H), 8.75–8.71 (m, 1H), 8.68 (d, *J* = 7.6 Hz, 1H), 8.54 (d, *J* = 7.0 Hz, 2H), 8.50 (d, *J* = 2.5 Hz, 1H), 7.83 (d, *J* = 5.3 Hz, 1H), 7.60 (t, *J* = 7.2 Hz, 2H), 7.57–7.55 (m, 1H), 7.53 (d, *J* = 7.8 Hz, 1H), 7.31–7.22 (m, 2H); ^13^C NMR (125 MHz, DMSO-*d_6_*): *δ* 163.78, 162.85, 157.10, 138.72, 137.86, 131.09, 130.25, 129.25, 128.23, 125.80, 122.92, 122.25, 121.60, 114.68, 113.87, 112.81; HRMS: *m*/*z* [M + H]^+^ calcd for C_18_H_13_N_3_, 272.1182; found, 272.1189.

3-(2-(4-methoxyphenyl)pyrimidin-4-yl)-1*H*-indole (**1b**). Yield: 45%; white solid; m.p. 194–196 °C; ^1^H NMR (500 MHz, DMSO-*d_6_*): *δ* 11.90 (d, *J* = 8.4 Hz, 1H), 8.66 (s, 1H), 8.51–8.44 (m, 1H), 7.74 (dd, *J* = 5.5, 3.0 Hz, 1H), 7.54–7.49 (m, 1H), 7.27 (d, *J* = 7.0 Hz, 1H), 7.13 (dd, *J* = 8.4, 3.4 Hz, 1H), 3.85 (d, *J* = 3.2 Hz, 1H); ^13^C NMR (125 MHz, DMSO-*d_6_*): *δ* 163.49, 162.56, 161.76, 156.86, 137.71, 131.10, 129.95, 129.71, 125.69, 122.75, 122.14, 121.39, 114.47, 113.89, 113.81, 112.66, 55.75; HRMS: *m*/*z* [M + H]^+^ calcd for C_19_H_15_ON_3_, 302.1288; found, 302.1296.

3-(2-(3-methoxyphenyl)pyrimidin-4-yl)-1*H*-indole (**1c**). Yield: 31%; white solid; m.p. 188–190 °C; ^1^H NMR (500 MHz, DMSO-*d_6_*): *δ* 11.91 (s, 1H), 8.71 (d, *J* = 5.4 Hz, 1H), 8.68–8.63 (m, 1H), 8.49 (d, *J* = 2.9 Hz, 1H), 8.12 (d, *J* = 7.7 Hz, 1H), 8.08 (s, 1H), 7.82 (d, *J* = 5.4 Hz, 1H), 7.50 (dd, *J* = 9.3, 6.7 Hz, 2H), 7.26–7.20 (m, 2H), 7.12 (dd, *J* = 8.1, 2.2 Hz, 1H), 3.89 (s, 3H); ^13^C NMR (125 MHz, DMSO-*d_6_*): *δ* 163.38, 162.67, 160.00, 156.94, 140.06, 137.72, 130.23, 130.13, 125.68, 122.81, 122.08, 121.42, 120.46, 116.90, 114.64, 113.69, 112.98, 112.72, 55.56; HRMS: *m*/*z* [M + H]^+^ calcd for C_19_H_15_ON_3_, 302.1288; found, 302.1295.

3-(2-(4-fluorophenyl)pyrimidin-4-yl)-1*H*-indole (**1d**). Yield: 40%; white solid; m.p. 126–128 °C; ^1^H NMR (500 MHz, DMSO-*d_6_*): *δ* 11.94 (s, 1H), 8.70 (d, *J* = 5.4 Hz, 1H), 8.62 (dd, *J* = 6.4, 2.2 Hz, 1H), 8.56 (dd, *J* = 8.8, 5.8 Hz, 2H), 8.49 (d, *J* = 2.3 Hz, 1H), 7.81 (d, *J* = 5.4 Hz, 1H), 7.53–7.49 (m, 1H), 7.41 (t, *J* = 8.8 Hz, 2H), 7.24 (pd, *J* = 7.0, 3.5 Hz, 2H); ^13^C NMR (125 MHz, DMSO-*d_6_*): *δ* 165.24, 163.27, 162.75, 156.99, 137.73, 135.06, 130.44, 130.37, 130.23, 125.62, 122.81, 122.06, 121.48, 116.13, 115.96, 114.48, 113.65, 112.70; HRMS: *m*/*z* [M + H]^+^ calcd for C_18_H_12_N_3_F, 290.1088; found, 290.1096.

3-(2-(3-fluorophenyl)pyrimidin-4-yl)-1*H*-indole (**1e**). Yield: 37%; white solid; m.p. 128–130 °C; ^1^H NMR (500 MHz, DMSO-*d_6_*): *δ* 11.95 (s, 1H), 8.73 (d, *J* = 5.4 Hz, 1H), 8.61 (d, *J* = 7.5 Hz, 1H), 8.51 (d, *J* = 2.9 Hz, 1H), 8.38 (d, *J* = 7.8 Hz, 1H), 8.21 (d, *J* = 9.8 Hz, 1H), 7.85 (d, *J* = 5.5 Hz, 1H), 7.64 (dd, *J* = 14.1, 7.9 Hz, 1H), 7.52 (d, *J* = 7.4 Hz, 1H), 7.39 (td, *J* = 8.4, 2.4 Hz, 1H), 7.29–7.23 (m, 2H); ^13^C NMR (125 MHz, DMSO-*d_6_*): *δ* 163.97, 162.82, 162.43 (d, *J* = 3.2 Hz), 162.04, 157.05, 141.18 (d, *J* = 7.6 Hz), 137.75, 131.23 (d, *J* = 8.1 Hz), 130.39, 125.60, 124.12 (d, *J* = 2.5 Hz), 122.85, 121.94, 121.54, 117.87, 117.70, 115.04, 114.53, 114.34, 113.55, 112.76; HRMS: *m*/*z* [M + H]^+^ calcd for C_18_H_12_N_3_F, 290.1088; found, 290.1096.

3-(2-(p-tolyl)pyrimidin-4-yl)-1*H*-indole (**1f**). Yield: 36%; white solid; m.p. 193–195 °C; ^1^H NMR (500 MHz, DMSO-*d_6_*): *δ* 11.90 (s, 1H), 8.68 (d, *J* = 5.1 Hz, 1H), 8.64 (d, *J* = 7.2 Hz, 1H), 8.45 (s, 1H), 8.40 (d, *J* = 7.8 Hz, 2H), 7.77 (d, *J* = 5.0 Hz, 1H), 7.51 (d, *J* = 7.6 Hz, 1H), 7.38 (d, *J* = 7.5 Hz, 2H), 7.28–7.21 (m, 2H), 2.39 (s, 3H); ^13^C NMR (125 MHz, DMSO-*d_6_*): *δ* 163.71, 162.60, 156.93, 140.72, 137.70, 135.89, 129.96, 129.75, 128.08, 125.64, 122.80, 122.09, 121.46, 114.28, 113.78, 112.69, 21.48; HRMS: *m*/*z* [M + H]^+^ calcd for C_19_H_15_N_3_, 286.1339; found, 286.1346.

3-(2-(m-tolyl)pyrimidin-4-yl)-1*H*-indole (**1g**). Yield: 47%; white solid; m.p. 121–123 °C; ^1^H NMR (500 MHz, DMSO-*d_6_*): *δ* 11.92 (s, 1H), 8.71 (d, *J* = 5.4 Hz, 1H), 8.66 (d, *J* = 7.5 Hz, 1H), 8.48 (d, *J* = 2.5 Hz, 1H), 8.34 (d, *J* = 8.1 Hz, 2H), 7.81 (d, *J* = 5.4 Hz, 1H), 7.52 (d, *J* = 7.9 Hz, 1H), 7.47 (t, *J* = 7.5 Hz, 1H), 7.36 (d, *J* = 7.3 Hz, 1H), 7.25 (dd, *J* = 14.6, 7.3 Hz, 2H), 2.45 (s, 3H); ^13^C NMR (125 MHz, DMSO-*d_6_*): *δ* 163.73, 162.63, 156.94, 138.56, 138.14, 137.73, 131.58, 130.06, 129.02, 128.74, 125.67, 125.31, 122.77, 122.08, 121.44, 114.50, 113.79, 112.70, 21.71; HRMS: *m*/*z* [M + H]^+^ calcd for C_19_H_15_N_3_, 286.1339; found, 286.1347.

5-bromo-3-(2-phenylpyrimidin-4-yl)-1*H*-indole (**3a**). Yield: 28%; white solid; m.p. 201–203 °C; ^1^H NMR (500 MHz, DMSO-*d*_6_): *δ* 12.11 (s, 1H), 8.86 (d, *J* = 2.0 Hz, 1H), 8.75 (d, *J* = 5.4 Hz, 1H), 8.55 (d, *J* = 3.0 Hz, 1H), 8.50–8.46 (m, 2H), 7.83 (d, *J* = 5.4 Hz, 1H), 7.58 (tdd, *J* = 6.8, 3.8, 1.7 Hz, 3H), 7.49 (d, *J* = 8.6 Hz, 1H), 7.37 (dd, *J* = 8.6, 2.0 Hz, 1H); ^13^C NMR (125 MHz, DMSO-*d*_6_): *δ* 163.68, 162.17, 157.25, 138.52, 136.40, 131.39, 131.11, 129.14, 127.99, 127.43, 125.31, 124.51, 114.71, 114.61, 114.07, 113.30; HRMS: *m*/*z* [M + H]^+^ calcd for C_18_H_12_N_3_Br, 350.0287; found, 350.0287.

5-bromo-3-(2-(4-methoxyphenyl)pyrimidin-4-yl)-1*H*-indole (**3b**). Yield: 48%; pale yellow solid; m.p. 97–99 °C; ^1^H NMR (500 MHz, DMSO-*d*_6_): *δ* 12.08 (s, 1H), 8.84 (s, 1H), 8.68 (d, *J* = 5.4 Hz, 1H), 8.51 (s, 1H), 8.43 (d, *J* = 8.7 Hz, 2H), 7.75 (d, *J* = 5.4 Hz, 1H), 7.49 (d, *J* = 8.6 Hz, 1H), 7.36 (dd, *J* = 8.6, 1.6 Hz, 1H), 7.12 (d, *J* = 8.8 Hz, 2H), 3.86 (s, 3H); ^13^C NMR (125 MHz, DMSO-*d*_6_): *δ* 163.52, 162.01, 161.85, 157.13, 136.37, 131.23, 130.99, 129.59, 127.42, 125.26, 124.49, 114.68, 114.49, 114.00, 113.89, 113.41, 55.82; HRMS calcd for C_19_H_14_N_3_^81^Br [M + H]^+^ 382.0373, found: 382.0382.

5-bromo-3-(2-(3-methoxyphenyl)pyrimidin-4-yl)-1*H*-indole (**3c**). Yield: 55%; white solid; m.p. 192–194 °C; ^1^H NMR (500 MHz, DMSO-*d*_6_): *δ* 12.11 (s, 1H), 8.91 (s, 1H), 8.73 (d, *J* = 5.4 Hz, 1H), 8.55 (d, *J* = 2.9 Hz, 1H), 8.08 (dd, *J* = 10.7, 4.5 Hz, 2H), 7.83 (d, *J* = 5.4 Hz, 1H), 7.52–7.45 (m, 2H), 7.37 (dd, *J* = 8.6, 1.7 Hz, 1H), 7.12 (dd, *J* = 8.1, 2.4 Hz, 1H), 3.95 (s, 3H); ^13^C NMR (125 MHz, DMSO-*d*_6_): *δ* 163.42, 162.18, 160.04, 157.14, 139.95, 136.40, 131.44, 130.20, 127.45, 125.33, 124.58, 120.45, 117.82, 114.72, 114.64, 114.07, 113.23, 112.01, 55.67; HRMS calcd for C_19_H_14_N_3_^81^Br [M + H]^+^ 382.0373, found: 382.0382.

5-bromo-3-(2-(4-fluorophenyl)pyrimidin-4-yl)-1*H*-indole (**3d**). Yield: 50%; white solid; m.p. 88–90 °C; ^1^H NMR (500 MHz, DMSO-*d*_6_): *δ* 12.13 (s, 1H), 8.79 (d, *J* = 1.2 Hz, 1H), 8.73 (d, *J* = 5.4 Hz, 1H), 8.55 (d, *J* = 2.8 Hz, 1H), 8.51 (dd, *J* = 8.5, 5.9 Hz, 2H), 7.82 (d, *J* = 5.4 Hz, 1H), 7.49 (d, *J* = 8.6 Hz, 1H), 7.44–7.34 (m, 3H); ^13^C NMR (125 MHz, DMSO-*d*_6_): *δ* 165.29, 163.32, 162.80, 162.18, 157.28, 136.41, 135.03, 131.48, 130.27, 127.35, 125.33, 124.34, 116.17, 116.00, 114.74, 114.56, 114.09, 113.23; HRMS: *m*/*z* [M + H]^+^ calcd for C_18_H_11_N_3_BrF, 368.0193; found, 368.0189.

5-bromo-3-(2-(3-fluorophenyl)pyrimidin-4-yl)-1*H*-indole (**3e**). Yield: 43%; white solid; m.p. 205–206 °C; ^1^H NMR (500 MHz, DMSO-*d*_6_): *δ* 12.13 (s, 1H), 8.81 (s, 1H), 8.74 (d, *J* = 5.4 Hz, 1H), 8.55 (d, *J* = 2.8 Hz, 1H), 8.31 (d, *J* = 7.8 Hz, 1H), 8.17 (d, *J* = 10.3 Hz, 1H), 7.85 (d, *J* = 5.4 Hz, 1H), 7.62 (dd, *J* = 14.1, 7.8 Hz, 1H), 7.49 (d, *J* = 8.6 Hz, 1H), 7.43–7.33 (m, 2H); ^13^C NMR (125 MHz, DMSO-*d*_6_): *δ* 163.98, 162.45, 162.25, 162.05, 157.27, 141.09 (d, *J* = 7.6 Hz), 136.41, 131.59, 131.18 (d, *J* = 8.1 Hz), 127.36, 125.34, 124.42, 123.97 (d, *J* = 2.3 Hz), 117.95, 117.78, 115.06, 114.73, 114.51, 114.32, 114.14, 113.13; HRMS: *m*/*z* [M + H]^+^ calcd for C_18_H_11_N_3_BrF, 368.0193; found, 368.0198.

5-bromo-3-(2-(p-tolyl)pyrimidin-4-yl)-1*H*-indole (**3f**). Yield: 38%; pale yellow solid; m.p. 197–199 °C; ^1^H NMR (500 MHz, DMSO-*d*_6_): *δ* 12.09 (s, 1H), 8.86 (d, *J* = 1.7 Hz, 1H), 8.71 (d, *J* = 5.4 Hz, 1H), 8.53 (s, 1H), 8.37 (d, *J* = 8.1 Hz, 2H), 7.79 (d, *J* = 5.4 Hz, 1H), 7.49 (d, *J* = 8.6 Hz, 1H), 7.41–7.35 (m, 3H), 2.41 (s, 3H); ^13^C NMR (125 MHz, DMSO-*d*_6_): *δ* 163.75, 162.07, 157.19, 140.89, 136.38, 135.85, 131.27, 129.75, 127.97, 127.44, 125.27, 124.55, 114.69, 114.32, 114.03, 113.35, 21.44; HRMS: *m*/*z* [M + H]^+^ calcd for C_19_H_14_N_3_Br, 364.0444; found, 368.0444.

5-bromo-3-(2-(m-tolyl)pyrimidin-4-yl)-1H-indole (**3g**). Yield: 42%; pale yellow solid; m.p. 167–169 °C; ^1^H NMR (500 MHz, DMSO-*d*_6_): *δ* 12.09 (s, 1H), 8.94 (s, 1H), 8.73 (d, *J* = 5.4 Hz, 1H), 8.54 (d, *J* = 2.8 Hz, 1H), 8.35 (s, 1H), 8.27 (d, *J* = 7.7 Hz, 1H), 7.81 (d, *J* = 5.4 Hz, 1H), 7.51–7.44 (m, 2H), 7.40–7.35 (m, 2H), 2.47 (s, 3H); ^13^C NMR (125 MHz, DMSO-*d*_6_): *δ* 163.68, 162.08, 157.19, 138.44, 138.21, 136.37, 131.68, 131.32, 129.06, 128.73, 127.50, 125.25, 125.18, 124.75, 114.69, 114.45, 114.05, 113.30, 21.68; HRMS: *m*/*z* [M + H]^+^ calcd for C_19_H_14_N_3_Br, 364.0444; found, 368.0446.

6-bromo-3-(2-phenylpyrimidin-4-yl)-1*H*-indole (**4a**). Yield: 41%; pale yellow solid; m.p. 175–177 °C; ^1^H NMR (500 MHz, DMSO-*d*_6_): *δ* 12.03 (s, 1H), 8.74 (d, *J* = 5.4 Hz, 1H), 8.60 (d, *J* = 8.5 Hz, 1H), 8.51 (t, *J* = 4.4 Hz, 3H), 7.82 (d, *J* = 5.4 Hz, 1H), 7.70 (d, *J* = 1.5 Hz, 1H), 7.60–7.53 (m, 3H), 7.40 (dd, *J* = 8.5, 1.6 Hz, 1H); ^13^C NMR (125 MHz, DMSO-*d*_6_): *δ* 163.68, 162.26, 157.16, 138.58, 138.40, 131.05, 130.98, 129.15, 128.13, 124.70, 124.37, 123.85, 115.46, 115.27, 114.66, 113.90; HRMS: *m*/*z* [M + H]^+^ calcd for C_18_H_13_N_3_Br, 350.0287; found, 350.0288.

6-bromo-3-(2-(4-methoxyphenyl)pyrimidin-4-yl)-1*H*-indole (**4b**). Yield: 42%; white solid; m.p. >210 °C; ^1^H NMR (500 MHz, DMSO-*d*_6_): *δ* 12.00 (s, 1H), 8.68 (d, *J* = 5.4 Hz, 1H), 8.58 (d, *J* = 8.5 Hz, 1H), 8.47 (dd, *J* = 13.9, 5.7 Hz, 3H), 7.74 (d, *J* = 5.4 Hz, 1H), 7.69 (s, 1H), 7.38 (d, *J* = 8.5 Hz, 1H), 7.11 (d, *J* = 8.7 Hz, 2H), 3.85 (s, 3H); ^13^C NMR (125 MHz, DMSO-*d*_6_): *δ* 163.54, 162.08, 161.80, 157.07, 138.56, 130.93, 130.79, 129.75, 124.71, 124.27, 123.85, 115.41, 115.25, 114.49, 114.00, 113.92, 55.76; HRMS calcd for C_19_H_14_ON_3_^81^Br [M + H]^+^ 382.0373, found: 382.0384.

6-bromo-3-(2-(3-methoxyphenyl)pyrimidin-4-yl)-1*H*-indole (**4c**). Yield: 53%; yellow solid; m.p. 180–181 °C; ^1^H NMR (500 MHz, DMSO-*d*_6_): *δ* 12.03 (s, 1H), 8.74 (d, *J* = 5.4 Hz, 1H), 8.60 (d, *J* = 8.5 Hz, 1H), 8.51 (d, *J* = 2.8 Hz, 1H), 8.09 (d, *J* = 7.7 Hz, 1H), 8.05 (s, 1H), 7.82 (d, *J* = 5.4 Hz, 1H), 7.70 (s, 1H), 7.49 (t, *J* = 7.9 Hz, 1H), 7.40 (d, *J* = 8.5 Hz, 1H), 7.12 (dd, *J* = 8.1, 1.6 Hz, 1H), 3.89 (s, 3H); ^13^C NMR (125 MHz, DMSO-*d*_6_): *δ* 163.45, 162.20, 160.00, 157.16, 139.90, 138.58, 130.96, 130.26, 124.71, 124.30, 123.76, 120.49, 117.11, 115.47, 115.31, 114.75, 113.85, 112.83, 55.58; HRMS calcd for C_19_H_14_ON_3_^81^Br [M + H]^+^ 382.0373, found: 380.0377.

6-bromo-3-(2-(4-fluorophenyl)pyrimidin-4-yl)-1*H*-indole (**4d**). Yield: 32%; white solid; m.p. 187–188 °C; ^1^H NMR (500 MHz, DMSO-*d*_6_): *δ* 12.04 (s, 1H), 8.73 (d, *J* = 5.4 Hz, 1H), 8.59–8.52 (m, 3H), 8.52 (d, *J* = 2.9 Hz, 1H), 7.82 (d, *J* = 5.4 Hz, 1H), 7.70 (d, *J* = 1.7 Hz, 1H), 7.43–7.36 (m, 3H); ^13^C NMR (125 MHz, DMSO-*d*_6_): *δ* 165.27, 163.30, 162.81, 162.27, 157.22, 138.59, 130.46 (d, *J* = 8.7 Hz), 131.06, 130.50, 130.43, 124.65, 124.37, 123.79, 116.16, 115.99, 115.46, 115.29, 114.59, 113.83; HRMS: *m*/*z* [M + H]^+^ calcd for C_18_H_13_N_3_Br, 368.0193; found, 368.0193.

6-bromo-3-(2-(3-fluorophenyl)pyrimidin-4-yl)-1*H*-indole (**4e**). Yield: 61%; white solid; m.p. >210 °C; ^1^H NMR (500 MHz, DMSO-*d*_6_): *δ* 12.06 (s, 1H), 8.76 (d, *J* = 5.4 Hz, 1H), 8.57–8.52 (m, 2H), 8.35 (d, *J* = 7.8 Hz, 1H), 8.17 (d, *J* = 10.1 Hz, 1H), 7.86 (d, *J* = 5.4 Hz, 1H), 7.70 (d, *J* = 1.5 Hz, 1H), 7.63 (dd, *J* = 14.1, 7.9 Hz, 1H), 7.41 (dd, *J* = 8.5, 1.7 Hz, 2H); ^13^C NMR (125 MHz, DMSO-*d*_6_): *δ* 163.96, 162.50 (d, *J* = 3.2 Hz), 162.35, 162.03, 157.28, 138.60, 131.26, 130.77, 127.17, 124.62, 124.44, 124.18, 123.66, 115.50, 115.33, 115.16, 114.55, 114.37, 113.72; HRMS: *m*/*z* [M + H]^+^ calcd for C_18_H_11_N_3_BrF, 368.0193; found, 368.0192.

6-bromo-3-(2-(p-tolyl)pyrimidin-4-yl)-1*H*-indole (**4f**). Yield: 37%; yellow solid; m.p. 192–194 °C; ^1^H NMR (500 MHz, DMSO-*d*_6_): *δ* 12.02 (s, 1H), 8.71 (d, *J* = 5.4 Hz, 1H), 8.59 (d, *J* = 8.5 Hz, 1H), 8.49 (d, *J* = 2.8 Hz, 1H), 8.39 (d, *J* = 8.0 Hz, 2H), 7.78 (d, *J* = 5.4 Hz, 1H), 7.70 (d, *J* = 1.1 Hz, 1H), 7.39 (dd, *J* = 13.5, 4.7 Hz, 3H), 2.40 (s, 3H); ^13^C NMR (125 MHz, DMSO-*d*_6_): *δ* 163.75, 162.16, 157.09, 140.78, 138.57, 135.75, 130.87, 129.76, 128.11, 124.71, 124.31, 123.84, 115.43, 115.26, 114.38, 113.95, 21.49; HRMS: *m*/*z* [M + H]^+^ calcd for C_19_H_14_N_3_Br, 364.0444; found, 364.0443.

6-bromo-3-(2-(m-tolyl)pyrimidin-4-yl)-1*H*-indole (**4g**). Yield: 59%; pale yellow solid; m.p. 203–204 °C; ^1^H NMR (500 MHz, DMSO-*d*_6_): *δ* 12.02 (s, 1H), 8.73 (d, *J* = 5.4 Hz, 1H), 8.59 (d, *J* = 8.5 Hz, 1H), 8.50 (d, *J* = 2.7 Hz, 1H), 8.30 (d, *J* = 9.2 Hz, 2H), 7.81 (d, *J* = 5.4 Hz, 1H), 7.70 (d, *J* = 1.1 Hz, 1H), 7.46 (t, *J* = 7.5 Hz, 1H), 7.41 (dd, *J* = 8.5, 1.3 Hz, 1H), 7.36 (d, *J* = 7.5 Hz, 1H), 2.45 (s, 3H); ^13^C NMR (125 MHz, DMSO-*d*_6_): *δ* 163.78, 162.15, 157.17, 138.57, 138.40, 138.21, 131.67, 130.89, 129.07, 128.73, 125.32, 124.70, 124.34, 123.80, 115.44, 115.27, 114.60, 113.93, 21.69; HRMS: *m*/*z* [M + H]^+^ calcd for C_19_H_14_N_3_Br, 364.0444; found, 364.0443.

#### 4.1.2. General Procedure for Synthesis of **2a**–**g** Meridianin Analogs

To a stirring solution of 4-hydroxyindole (**12**) in dry DMF (10 mL) was added sodium hydride (5.0 equiv.) at 0 °C, and the mixture was stirred for 30 min. Then, *p*-toluenesulfonyl (3.0 equiv.) was added. After stirring for 4 h at room temperature. the reaction was quenched with saturated NaHCO_3_ solution and extracted with ethyl acetate (3 × 50 mL). The combined organic layers were washed with brine and dried over magnesium sulfate anhydrous. After filtration, the solvent was removed under vacuum, and the residue was purified by silica gel column chromatography (petroleum ether/ethyl acetate 10:1) to give compound **13**.

1-tosyl-1*H*-indol-4-yl 4-methylbenzenesulfonate (**13**). Yield: 85%; pale yellow solid; m.p. 100–102 °C; ^1^H NMR (400 MHz, DMSO-*d_6_*): *δ* 7.89 (d, *J* = 8.5 Hz, 1H), 7.88 (d, *J* = 3.4 Hz, 1H), 7.85 (s, 1H), 7.78 (t, *J* = 4.1 Hz, 1H), 7.71 (s, 1H), 7.69 (s, 1H), 7.38 (dd, *J* = 15.4, 7.3 Hz, 4H), 7.32 (d, *J* = 8.2 Hz, 1H), 6.93 (d, *J* = 8.0 Hz, 1H), 6.51 (d, *J* = 3.7 Hz, 1H), 2.38 (s, 3H), 2.33 (s, 3H); ^13^C NMR (100 MHz, DMSO*-d_6_*): *δ* 146.42, 146.38, 141.98, 135.99, 134.31, 131.72, 130.84, 130.62, 128.71, 128.33, 127.28, 125.88, 124.78, 117.02, 112.81, 105.63, 21.62, 21.52; HRMS: *m*/*z* [M + H]^+^ calcd for C_22_H_20_O_5_NS_2_, 442.0777; found, 442.0789.

To a stirring solution of acetic anhydride (2.0 equiv.) in dry dichloromethane (8 mL) was added aluminum chloride (5.0 equiv.) at 0 °C. Then, compound **13** in dry dichloromethane (8 mL) was added dropwise, and the mixture was stirred for 2 h at room temperature. The reaction was quenched with saturated aqueous NH_4_Cl and extracted with ethyl acetate (3 × 50 mL). The combined organic layers were washed with brine and dried over magnesium sulfate anhydrous. After filtration, the solvent was removed under vacuum, and the residue was purified by silica gel column chromatography (petroleum ether/ethyl acetate 8:1) to give compound **14**.

3-acetyl-1-tosyl-1*H*-indol-4-yl 4-methylbenzenesulfonate (**14**). Yield: 70%; pale yellow solid; m.p. 163–165 °C; ^1^H NMR (400 MHz, DMSO-*d_6_*): *δ* 8.66 (s, 1H), 8.04 (s, 1H), 8.02 (s, 1H), 7.95 (d, *J* = 7.9 Hz, 1H), 7.52 (s, 1H), 7.50 (s, 1H), 7.47 (s, 1H), 7.45 (s, 1H), 7.39 (t, *J* = 8.2 Hz, 1H), 7.34 (s, 1H), 7.32 (s, 1H), 6.90–6.85 (m, 1H), 2.48 (s, 3H), 2.38 (s, 3H), 2.36 (s, 3H); ^13^C NMR (100 MHz, DMSO-*d_6_*): *δ* 192.02, 147.08, 146.02, 142.26, 136.52, 134.25, 133.66, 131.87, 131.07, 130.27, 128.79, 128.67, 127.81, 126.84, 126.70, 122.26, 120.73, 119.30, 112.91, 105.45, 64.26, 29.61, 21.60; HRMS: *m*/*z* [M + H]^+^ calcd for C_24_H_22_O_6_NS_2_, 484.0883; found, 484.0891.

To a solution of compound **14** in DMF (5 mL) was added DMF-DMA (1.5 equiv.). The reaction mixture was stirred at 110 °C for 5 h under a nitrogen atmosphere. Then, the mixture was poured into ice water and extracted with ethyl acetate (3 × 50 mL). The combined organic layers were washed with brine and dried over magnesium sulfate anhydrous. After filtration, the solvent was removed under vacuum, and the residue was purified by silica gel column chromatography (petroleum ether/ethyl acetate 2:1) to give intermediate **15**.

(*E*)-3-(3-(dimethylamino)acryloyl)-1-tosyl-1*H*-indol-4-yl 4-methylbenzenesulfonate (**15**). Yield: 64%; pale yellow solid; m.p. 129–131 °C; ^1^H NMR (400 MHz, DMSO-*d_6_*): *δ* 8.09 (s, 1H), 7.97 (s, 1H), 7.95 (d, *J* = 2.0 Hz, 2H), 7.93 (s, 1H), 7.60 (s, 1H), 7.58 (s, 1H), 7.43 (s, 2H), 7.41 (s, 1H), 7.36 (d, *J* = 8.2 Hz, 1H), 7.33 (s, 1H), 7.31 (s, 1H), 7.01 (d, *J* = 8.0 Hz, 1H), 5.40 (d, *J* = 12.5 Hz, 1H), 3.09 (d, *J* = 24.6 Hz, 3H), 2.81 (d, *J* = 16.5 Hz, 3H), 2.37 (s, 3H), 2.34 (d, *J* = 6.3 Hz, 3H); ^13^C NMR (100 MHz, DMSO-*d_6_*): *δ* 170.81, 162.77, 154.45, 146.69, 145.76, 142.23, 136.55, 134.01, 132.10, 130.94, 130.23, 129.12, 128.76, 127.58, 126.13, 121.74, 118.13, 112.70, 60.22, 45.73, 37.16, 36.24, 36.24, 31.23, 21.58, 21.22, 14.54; HRMS: *m*/*z* [M + H]^+^ calcd for C_27_H_27_O_6_N_2_S_2_, 539.1305; found, 539.1316.

To a solution of intermediate **15** in 2-methoxyethanol (5 mL) was added **5**–**11** (2.5 equiv.) and potassium carbonate (2.0 equiv.), respectively. The reaction mixture was stirred at 120 °C for 20 h under a nitrogen atmosphere. Then, the mixture was poured into ice water and extracted with ethyl acetate (3 × 50 mL). The combined organic layers were washed with brine and dried over magnesium sulfate anhydrous. After filtration, the solvent was removed under vacuum, and the residue was purified by silica gel column chromatography (petroleum ether/ethyl acetate 10:1) to give get the final target compounds **2a**–**g**.

3-(2-phenylpyrimidin-4-yl)-1*H*-indol-4-ol (**2a**). Yield: 46%; pale yellow solid; m.p. 195–197 °C; ^1^H NMR (500 MHz, DMSO-*d*_6_): *δ* 13.44 (s, 1H), 12.07 (s, 1H), 8.74 (d, *J* = 5.6 Hz, 1H), 8.53 (s, 1H), 8.23 (dd, *J* = 6.7, 1.7 Hz, 2H), 8.00 (d, *J* = 5.6 Hz, 1H), 7.61 (dd, *J* = 9.7, 4.9 Hz, 3H), 7.06 (t, *J* = 7.9 Hz, 1H), 6.90 (d, *J* = 8.0 Hz, 1H), 6.50 (d, *J* = 7.7 Hz, 1H); ^13^C NMR (125 MHz, DMSO-*d*_6_): *δ* 163.56, 161.32, 157.80, 152.12, 139.93, 137.36, 131.38, 130.71, 129.39, 128.30, 125.23, 114.73, 114.59, 113.51, 106.42, 103.47; HRMS: *m*/*z* [M + H]^+^ calcd for C_18_H_13_ON_3_, 288.1131; found, 288.1129.

3-(2-(4-methoxyphenyl)pyrimidin-4-yl)-1*H*-indol-4-ol (**2b**). Yield: 52%; pale yellow solid; m.p. >210 °C; ^1^H NMR (500 MHz, DMSO-*d*_6_): *δ* 13.55 (s, 1H), 12.00 (s, 1H), 8.68 (d, *J* = 5.6 Hz, 1H), 8.50 (d, *J* = 2.8 Hz, 1H), 8.21 (d, *J* = 8.8 Hz, 2H), 7.91 (d, *J* = 5.6 Hz, 1H), 7.15 (d, *J* = 8.8 Hz, 2H), 7.05 (t, *J* = 7.9 Hz, 1H), 6.89 (d, *J* = 7.9 Hz, 1H), 6.49 (d, *J* = 7.6 Hz, 1H), 3.86 (s, 3H); ^13^C NMR (125 MHz, DMSO-*d*_6_): *δ* 163.24, 162.06, 161.15, 157.73, 152.16, 139.88, 130.49, 129.98, 129.64, 125.20, 114.79, 114.77, 113.83, 113.59, 106.32, 103.41, 55.86;HRMS: *m*/*z* [M + H]^+^ calcd for C_19_H_15_O_2_N_3_, 318.1238; found, 318.1237.

3-(2-(3-methoxyphenyl)pyrimidin-4-yl)-1*H*-indol-4-ol (**2c**). Yield: 38%; yellow solid; m.p. 207–209 °C; ^1^H NMR (500 MHz, DMSO-*d*_6_): *δ* 13.39 (s, 1H), 12.04 (s, 1H), 8.73 (d, *J* = 5.6 Hz, 1H), 8.53 (d, *J* = 2.7 Hz, 1H), 8.00 (d, *J* = 5.6 Hz, 1H), 7.81 (d, *J* = 7.8 Hz, 1H), 7.78 (s, 1H), 7.51 (t, *J* = 7.9 Hz, 1H), 7.16 (d, *J* = 8.1 Hz, 1H), 7.05 (t, *J* = 7.8 Hz, 1H), 6.89 (d, *J* = 8.0 Hz, 1H), 6.49 (d, *J* = 7.7 Hz, 1H), 3.87 (s, 3H); ^13^C NMR (125 MHz, DMSO-*d*_6_): *δ* 163.34, 161.31, 160.10, 157.75, 152.13, 139.92, 138.74, 130.73, 130.52, 125.24, 120.57, 117.21, 114.70, 113.50, 113.47, 106.46, 103.47, 55.68; HRMS: *m*/*z* [M + H]^+^ calcd for C_19_H_15_O_2_N_3_, 318.1237; found, 318.1231.

3-(2-(4-fluorophenyl)pyrimidin-4-yl)-1*H*-indol-4-ol (**2d**). Yield: 55%; yellow solid; m.p. 189–191 °C; ^1^H NMR (500 MHz, DMSO-*d*_6_): *δ* 13.39 (s, 1H), 12.04 (s, 1H), 8.72 (d, *J* = 5.6 Hz, 1H), 8.53 (d, *J* = 2.9 Hz, 1H), 8.27 (dd, *J* = 8.7, 5.6 Hz, 2H), 7.99 (d, *J* = 5.6 Hz, 1H), 7.44 (t, *J* = 8.8 Hz, 2H), 7.06 (t, *J* = 7.9 Hz, 1H), 6.90 (d, *J* = 8.0 Hz, 1H), 6.50 (d, *J* = 7.6 Hz, 1H); ^13^C NMR (125 MHz, DMSO-*d*_6_): *δ* 165.37, 163.39, 162.59, 161.34, 157.79, 152.06, 139.92, 133.85, 130.75, 125.26, 116.50, 116.33, 114.72, 114.53, 113.46, 106.42, 103.52; HRMS: *m*/*z* [M + H]^+^ calcd for C_18_H_12_ON_3_F, 306.1037; found, 306.1030.

3-(2-(3-fluorophenyl)pyrimidin-4-yl)-1*H*-indol-4-ol (**2e**). Yield: 40%; yellow solid; m.p. 159–161 °C; ^1^H NMR (500 MHz, DMSO-*d*_6_): *δ* 13.30 (s, 1H), 12.07 (s, 1H), 8.75 (d, *J* = 5.6 Hz, 1H), 8.55 (d, *J* = 3.1 Hz, 1H), 8.09–8.06 (m, 1H), 8.04 (d, *J* = 5.7 Hz, 1H), 7.67 (t, *J* = 8.0, 1H), 7.06 (t, *J* = 7.9, 2H), 6.89 (d, *J* = 8.1 Hz, 2H), 6.51 (d, *J* = 7.7 Hz, 1H); ^13^C NMR (125 MHz, DMSO-*d*_6_): *δ* 163.91, 162.28, 161.98, 161.45, 157.79, 152.02, 139.94, 136.91, 131.57, 130.97, 125.29, 118.37, 118.20, 115.10, 113.38, 106.83, 106.51, 103.57; HRMS: *m*/*z* [M + H]^+^ calcd for C_18_H_12_ON_3_F, 306.1037; found, 306.1033.

3-(2-(p-tolyl)pyrimidin-4-yl)-1*H*-indol-4-ol (**2f**). Yield: 54%; yellow solid; m.p. 175–177 °C; ^1^H NMR (500 MHz, DMSO-*d*_6_): *δ* 13.48 (s, 1H), 12.02 (s, 1H), 8.71 (d, *J* = 3.9 Hz, 1H), 8.51 (s, 1H), 8.12 (d, *J* = 6.9 Hz, 2H), 7.96 (d, *J* = 4.2 Hz, 1H), 7.40 (d, *J* = 7.3 Hz, 2H), 7.05 (t, *J* = 7.1 Hz, 1H), 6.89 (d, *J* = 7.2 Hz, 1H), 6.49 (d, *J* = 6.9 Hz, 1H), 2.40 (s, 3H); ^13^C NMR (125 MHz, DMSO-*d*_6_): *δ* 163.57, 161.23, 157.77, 152.15, 141.32, 139.90, 134.63, 130.57, 129.99, 128.27, 125.21, 114.74, 114.30, 113.56, 106.39, 103.42, 21.44; HRMS: *m*/*z* [M + H]^+^ calcd for C_19_H_15_ON_3_, 302.1288; found, 302.1281.

3-(2-(m-tolyl)pyrimidin-4-yl)-1*H*-indol-4-ol (**2g**). Yield: 66%; yellow solid; m.p. 144–146 °C; ^1^H NMR (500 MHz, DMSO-*d*_6_): *δ* 13.41 (s, 1H), 12.02 (s, 1H), 8.72 (d, *J* = 5.6 Hz, 1H), 8.52 (d, *J* = 3.0 Hz, 1H), 8.06 (s, 1H), 7.99 (t, *J* = 6.8 Hz, 2H), 7.48 (t, *J* = 7.6 Hz, 1H), 7.40 (d, *J* = 7.5 Hz, 1H), 7.05 (dd, *J* = 9.5, 6.2 Hz, 1H), 6.89 (d, *J* = 8.0 Hz, 1H), 6.49 (d, *J* = 7.7 Hz, 1H), 2.43 (s, 3H); ^13^C NMR (125 MHz, DMSO-*d*_6_): *δ* 163.67, 161.29, 157.75, 152.15, 139.92, 138.55, 137.34, 131.99, 130.64, 129.29, 129.03, 125.37, 125.22, 114.72, 114.52, 113.54, 106.44, 103.43, 21.61; HRMS: *m*/*z* [M + H]^+^ calcd for C_19_H_15_ON_3_, 302.1288; found, 302.1281.

#### 4.1.3. General Procedure for Synthesis of **5a**–**g** Meridianin Analogs

To the solution of **1a**–**g** in DMF (5 mL) was added 1,6-dibromohexane (5.0 equiv.), and the mixture was stirred at 50 °C for 5 h. Then, the reaction mixture was removed under vacuum, and the residue was poured into ice water and extracted with ethyl acetate (3 × 50 mL). The combined organic layers were washed with brine, dried over magnesium sulfate anhydrous and concentrated to give intermediates **23**–**29** and used in the next step without further purification. To a stirring solution of compounds **23**–**29** in ethanol was added thiocarbamide (2.0 equiv.), and the mixture was stirred at 65 °C for 3 h. Then, the solvent was removed under vacuum, and the residue was purified by silica gel column chromatography (dichloromethane/methanol 10:1) to give the final target compounds **5a**–**g**.

2-(6-(3-(2-phenylpyrimidin-4-yl)-1*H*-indol-1-yl)hexyl)isothiouronium (**5a**). Yield: 70%; yellow oily substance; ^1^H NMR (500 MHz, DMSO-*d_6_*): *δ* 8.97 (s, *J* = 28.0 Hz, 3H), 8.73 (d, *J* = 5.4 Hz, 1H), 8.68–8.64 (m, 1H), 8.56 (s, 1H), 8.53–8.49 (m, 2H), 7.77 (d, *J* = 5.4 Hz, 1H), 7.64–7.52 (m, 4H), 7.32–7.26 (m, 2H), 4.29 (t, *J* = 7.1 Hz, 2H), 3.11 (t, *J* = 7.3 Hz, 2H), 1.89–1.79 (m, 2H), 1.57 (dt, *J* = 14.9, 7.5 Hz, 2H), 1.46–1.36 (m, 2H), 1.31 (dt, *J* = 14.8, 7.3 Hz, 2H); ^13^C NMR (125 MHz, DMSO-*d_6_*): *δ* 170.30, 163.66, 162.26, 157.11, 138.51, 137.55, 133.02, 131.02, 129.13, 128.10, 126.13, 122.87, 122.35, 121.73, 114.51, 112.85, 111.20, 46.44, 30.49, 29.84, 28.71, 27.86, 26.06; HRMS calcd for C_25_H_27_N_5_S [M + H]^+^ 430.2060, found: 430.2065.

2-(6-(3-(2-(4-methoxyphenyl)pyrimidin-4-yl)-1*H*-indol-1-yl)hexyl)isothiouronium (**5b**). Yield: 78%; pale yellow solid; m.p. 191–193 °C; ^1^H NMR (500 MHz, DMSO-*d_6_*): *δ* 8.96 (s, 3H), 8.70 (d, *J* = 5.3 Hz, 1H), 8.64 (dd, *J* = 6.2, 2.7 Hz, 1H), 8.53 (s, 1H), 8.41 (d, *J* = 8.0 Hz, 2H), 7.73 (d, *J* = 5.4 Hz, 1H), 7.64–7.59 (m, 1H), 7.38 (d, *J* = 7.6 Hz, 2H), 7.33–7.25 (m, 2H), 4.29 (t, *J* = 6.6 Hz, 2H), 3.10 (t, *J* = 7.3 Hz, 2H), 2.40 (s, 3H), 1.88–1.80 (m, 2H), 1.56 (dd, *J* = 14.1, 6.9 Hz, 2H), 1.39 (dd, *J* = 14.0, 6.8 Hz, 2H), 1.35–1.26 (m, 2H); ^13^C NMR (125 MHz, DMSO-*d_6_*): *δ* 170.27, 163.72, 162.17, 157.06, 140.73, 137.54, 135.86, 132.91, 129.74, 128.08, 126.13, 122.83, 122.34, 121.69, 114.23, 112.90, 111.18, 46.43, 30.48, 29.85, 28.71, 27.86, 26.06, 21.51; HRMS calcd for C_26_H_29_ON_5_S [M + H]^+^ 460.2166, found: 460.2165.

2-(6-(3-(2-(3-methoxyphenyl)pyrimidin-4-yl)-1*H*-indol-1-yl)hexyl)isothiouronium (**5c**). Yield: 82%; pale yellow solid; m.p. 155–157 °C; ^1^H NMR (500 MHz, DMSO-*d_6_*): *δ* 8.99 (s, 3H), 8.73 (d, *J* = 5.4 Hz, 1H), 8.66 (dd, *J* = 5.7, 3.2 Hz, 1H), 8.55 (s, 1H), 8.11 (d, *J* = 7.7 Hz, 1H), 8.07 (s, 1H), 7.77 (d, *J* = 5.4 Hz, 1H), 7.64–7.60 (m, 1H), 7.50 (t, *J* = 7.9 Hz, 1H), 7.29 (dd, *J* = 6.1, 3.1 Hz, 2H), 7.13 (dd, *J* = 8.1, 1.9 Hz, 1H), 4.29 (t, *J* = 6.9 Hz, 2H), 3.89 (s, 3H), 3.11 (t, *J* = 7.3 Hz, 2H), 1.90-1.80 (m, 2H), 1.61–1.51 (m, 2H), 1.40 (dt, *J* = 14.5, 7.3 Hz, 2H), 1.31 (dt, *J* = 14.5, 7.5 Hz, 2H); ^13^C NMR (125 MHz, DMSO-*d_6_*): *δ* 170.31, 163.40, 162.23, 160.00, 157.06, 139.99, 137.55, 133.02, 130.25, 126.14, 122.88, 122.31, 121.68, 120.46, 116.88, 114.59, 113.05, 112.80, 111.23, 55.58, 46.44, 30.48, 29.84, 28.71, 27.86, 26.06; HRMS calcd for C_26_H_29_ON_5_S [M + H]^+^ 460.2166, found: 460.2166.

2-(6-(3-(2-(4-fluorophenyl)pyrimidin-4-yl)-1*H*-indol-1-yl)hexyl)isothiouronium (**5d**). Yield: 58%; yellow oily substance; ^1^H NMR (500 MHz, DMSO-*d_6_*): *δ* 8.99 (s, 3H), 8.71 (d, *J* = 5.4 Hz, 1H), 8.62 (dd, *J* = 6.0, 3.0 Hz, 1H), 8.58–8.53 (m, 3H), 7.77 (d, *J* = 5.5 Hz, 1H), 7.62 (dd, *J* = 6.0, 3.1 Hz, 1H), 7.40 (t, *J* = 8.8 Hz, 2H), 7.33 –7.26 (m, 2H), 4.28 (t, *J* = 7.0 Hz, 2H), 3.11 (t, *J* = 7.3 Hz, 2H), 1.84 (dt, *J* = 14.5, 7.2 Hz, 2H), 1.57 (dt, *J* = 14.7, 7.5 Hz, 2H), 1.40 (dt, *J* = 14.5, 7.3 Hz, 2H), 1.31 (dt, *J* = 15.1, 7.6 Hz, 2H); ^13^C NMR (125 MHz, DMSO-*d_6_*): *δ* 170.32, 165.26, 163.29, 162.76, 162.30, 157.12, 137.55, 134.99, 133.13, 130.42 (d, *J* = 8.7 Hz), 126.08, 122.88, 122.28, 121.75, 116.14, 115.97, 114.43, 112.77, 111.22, 46.45, 30.48, 29.83, 28.72, 27.85, 26.05; HRMS calcd for C_25_H_26_N_5_FS [M + H]; 448.1966, found: 448.1968.

2-(6-(3-(2-(3-fluorophenyl)pyrimidin-4-yl)-1*H*-indol-1-yl)hexyl)isothiouronium (**5e**). Yield: 77%; yellow oily substance; ^1^H NMR (500 MHz, DMSO-*d_6_*): *δ* 9.00 (s, 3H), 8.75 (d, *J* = 5.5 Hz, 1H), 8.62–8.57 (m, 2H), 8.36 (d, *J* = 7.8 Hz, 1H), 8.19 (dd, *J* = 6.4, 5.4 Hz, 1H), 7.81 (d, *J* = 5.5 Hz, 1H), 7.67–7.61 (m, 2H), 7.40 (td, *J* = 8.4, 2.0 Hz, 1H), 7.33–7.26 (m, 2H), 4.29 (t, *J* = 7.0 Hz, 2H), 3.11 (t, *J* = 7.3 Hz, 2H), 1.88–1.80 (m, 2H), 1.57 (dt, *J* = 14.8, 7.5 Hz, 2H), 1.40 (dt, *J* = 14.5, 7.3 Hz, 2H), 1.31 (dt, *J* = 14.9, 7.5 Hz, 2H); ^13^C NMR (125 MHz, DMSO-*d_6_*): *δ* 170.33, 163.97, 162.40 (d, *J* = 13.0 Hz), 162.04, 157.21, 141.11 (d, *J* = 7.6 Hz), 137.58, 133.28, 131.26 (d, *J* = 8.1 Hz), 126.05, 124.14, 122.91, 122.15, 121.80, 117.92, 117.76, 114.99, 114.53, 114.35, 112.66, 111.27, 46.47, 30.47, 29.84, 28.72, 27.85, 26.06; HRMS calcd for C_25_H_26_N_5_FS [M + H]^+^ 448.1966, found: 448.1974.

2-(6-(3-(2-(p-tolyl)pyrimidin-4-yl)-1*H*-indol-1-yl)hexyl)isothiouronium (**5f**). Yield: 60%; white solid; m.p. 205–207 °C; ^1^H NMR (500 MHz, DMSO-*d_6_*): *δ* 8.96 (s, 3H), 8.70 (d, *J* = 5.3 Hz, 1H), 8.64 (dd, *J* = 6.2, 2.7 Hz, 1H), 8.53 (s, 1H), 8.41 (d, *J* = 8.0 Hz, 2H), 7.73 (d, *J* = 5.4 Hz, 1H), 7.64–7.59 (m, 1H), 7.38 (d, *J* = 7.6 Hz, 2H), 7.33–7.25 (m, 2H), 4.29 (t, *J* = 6.6 Hz, 2H), 3.10 (t, *J* = 7.3 Hz, 2H), 2.40 (s, 3H), 1.88–1.80 (m, 2H), 1.56 (dd, *J* = 14.1, 6.9 Hz, 2H), 1.39 (dd, *J* = 14.0, 6.8 Hz, 2H), 1.35–1.26 (m, 2H); ^13^C NMR (125 MHz, DMSO-*d_6_*): *δ* 170.27, 163.72, 162.17, 157.06, 140.73, 137.54, 135.86, 132.91, 129.74, 128.08, 126.13, 122.83, 122.34, 121.69, 114.23, 112.90, 111.18, 46.43, 30.48, 29.85, 28.71, 27.86, 26.06, 21.51; HRMS calcd for C_26_H_29_N_5_S [M + H]^+^ 444.2216, found: 444.2228.

2-(6-(3-(2-(m-tolyl)pyrimidin-4-yl)-1*H*-indol-1-yl)hexyl)isothiouronium (**5g**). Yield: 74%; yellow solid; m.p. 125–127 °C; ^1^H NMR (500 MHz, DMSO-*d_6_*): *δ* 8.96 (s, 3H), 8.72 (d, *J* = 5.4 Hz, 1H), 8.68–8.63 (m, 1H), 8.54 (s, 1H), 8.32 (d, *J* = 7.2 Hz, 2H), 7.76 (d, *J* = 5.4 Hz, 1H), 7.65–7.59 (m, 1H), 7.47 (t, *J* = 7.7 Hz, 1H), 7.36 (d, *J* = 7.4 Hz, 1H), 7.32–7.26 (m, 2H), 4.29 (t, *J* = 6.9 Hz, 2H), 3.11 (t, *J* = 7.3 Hz, 2H), 2.45 (s, 3H), 1.89–1.80 (m, 2H), 1.59–1.53 (m, 2H), 1.40 (dt, *J* = 14.7, 7.3 Hz, 2H), 1.35–1.28 (m, 2H); ^13^C NMR (125 MHz, DMSO-*d_6_*): *δ* 170.30, 163.74, 162.19, 157.07, 138.48, 138.16, 137.54, 132.95, 131.64, 129.04, 128.72, 126.13, 125.31, 122.84, 122.31, 121.70, 114.45, 112.88, 111.20, 46.44, 30.49, 29.85, 28.71, 27.86, 26.06, 21.71; HRMS calcd for C_26_H_29_N_5_S [M + H]^+^ 444.2216, found: 444.2219.

#### 4.1.4. General Procedure for Synthesis of **6a**–**g** Meridianin Analogs

To the solution of **4a**–**g** in DMF (5 mL) was added 1,6-dibromohexane (5.0 equiv.), and the mixture was stirred at 50 °C for 5 h. Then, the reaction mixture was removed under vacuum, and the residue was poured into ice water and extracted with ethyl acetate (3 × 50 mL). The combined organic layers were washed with brine, dried over magnesium sulfate anhydrous and concentrated to give intermediates **30**–**36** and used in the next step without further purification. To a stirring solution of compounds **30**–**36** in ethanol was added thiocarbamide (2.0 equiv.), and the mixture was stirred at 65 °C for 3 h. Then, the solvent was removed under vacuum, and the residue was purified by silica gel column chromatography (dichloromethane/methanol 10:1) to give the final target compounds **6a**–**g**.

2-(6-(6-bromo-3-(2-phenylpyrimidin-4-yl)-1*H*-indol-1-yl)hexyl)isothiouronium (**6a**). Yield: 84%; white solid; m.p. 131–133 °C; ^1^H NMR (500 MHz, DMSO-*d*_6_): *δ* 9.20 (s, 3H), 8.75 (d, *J* = 5.4 Hz, 1H), 8.62–8.55 (m, 2H), 8.52–8.47 (m, 2H), 7.92 (d, *J* = 1.0 Hz, 1H), 7.78 (d, *J* = 5.4 Hz, 1H), 7.56 (p, *J* = 6.0 Hz, 3H), 7.43 (dd, *J* = 8.5, 1.4 Hz, 1H), 4.27 (t, *J* = 7.0 Hz, 2H), 3.06 (t, *J* = 7.2 Hz, 2H), 1.82 (dt, *J* = 14.5, 7.2 Hz, 2H), 1.57 (dt, *J* = 14.7, 7.3 Hz, 2H), 1.45–1.36 (m, 2H), 1.35–1.27 (m, 2H); ^13^C NMR (125 MHz, DMSO-*d*_6_): *δ* 169.33, 163.72, 161.83, 157.31, 138.45, 138.37, 133.72, 131.08, 129.16, 128.13, 125.07, 124.63, 124.02, 115.83, 114.62, 114.05, 113.10, 46.55, 30.35, 29.86, 28.85, 27.88, 26.02; HRMS: *m*/*z* [M + H]^+^ calcd for C_25_H_26_N_5_BrS, 510.1145; found, 510.1130.

2-(6-(6-bromo-3-(2-(4-methoxyphenyl)pyrimidin-4-yl)-1*H*-indol-1-yl)hexyl)isothiouronium (**6b**). Yield: 68%; pale yellow solid; m.p. 54–56 °C; ^1^H NMR (500 MHz, DMSO-*d*_6_): *δ* 9.32 (s, 3H), 8.74 (d, *J* = 4.6 Hz, 1H), 8.57 (dd, *J* = 26.4, 14.5 Hz, 2H), 8.13–7.99 (m, 2H), 7.92 (s, 1H), 7.78 (d, *J* = 4.3 Hz, 1H), 7.53–7.38 (m, 2H), 7.12 (d, *J* = 7.4 Hz, 1H), 4.27 (t, 2H), 3.88 (s, 3H), 3.03 (t, 2H), 1.81 (dt, 2H), 1.56 (dt, 2H), 1.40 (m, 2H), 1.30 (m, 2H); ^13^C NMR (125 MHz, DMSO-*d*_6_): *δ* 168.64, 163.46, 161.80, 160.00, 157.26, 139.86, 138.44, 133.73, 130.26, 125.09, 124.56, 123.94, 120.49, 117.08, 115.84, 114.73, 114.08, 113.05, 112.89, 55.58, 46.54, 30.25, 29.86, 28.94, 27.87, 26.02; HRMS: *m*/*z* [M + H]^+^ calcd for C_26_H_28_N_5_^81^BrS, 540.1250; found, 540.1236.

2-(6-(6-bromo-3-(2-(3-methoxyphenyl)pyrimidin-4-yl)-1H-indol-1-yl)hexyl)isothiouronium (**6c**). Yield: 65%; yellow solid; m.p. 120–122 °C; ^1^H NMR (500 MHz, DMSO-*d*_6_): *δ* 9.32 (s, 3H), 8.68 (d, *J* = 5.3 Hz, 1H), 8.58 (d, *J* = 8.6 Hz, 1H), 8.54 (s, 1H), 8.44 (d, *J* = 8.8 Hz, 2H), 7.91 (s, 1H), 7.69 (d, *J* = 5.3 Hz, 1H), 7.41 (dd, *J* = 8.5, 1.4 Hz, 1H), 7.11 (d, *J* = 8.9 Hz, 2H), 4.26 (t, *J* = 6.9 Hz, 2H), 3.85 (s, 3H), 3.03 (t, *J* = 7.1 Hz, 2H), 1.80 (dd, *J* = 14.4, 7.2 Hz, 2H), 1.61–1.53 (m, 2H), 1.39 (dd, *J* = 14.3, 7.2 Hz, 2H), 1.31 (d, *J* = 6.6 Hz, 2H); ^13^C NMR (125 MHz, DMSO-*d*_6_): *δ* 168.51, 163.55, 161.81, 161.68, 157.18, 138.42, 133.58, 130.89, 129.76, 125.08, 124.52, 124.03, 115.78, 114.49, 114.01, 113.89, 113.20, 55.77, 46.52, 30.24, 29.87, 28.95, 27.88, 26.03; HRMS: *m*/*z* [M + H]^+^ calcd for C_26_H_28_N_5_^81^BrS, 540.1250; found, 540.1234.

2-(6-(6-bromo-3-(2-(4-fluorophenyl)pyrimidin-4-yl)-1*H*-indol-1-yl)hexyl)isothiouronium (**6d**). Yield: 68%; yellow oily substance; ^1^H NMR (500 MHz, DMSO-*d*_6_): *δ* 9.33 (s, 3H), 8.73 (d, *J* = 5.4 Hz, 1H), 8.60–8.48 (m, 4H), 7.92 (d, *J* = 1.2 Hz, 1H), 7.77 (d, *J* = 5.4 Hz, 1H), 7.44–7.34 (m, 3H), 4.26 (t, *J* = 7.0 Hz, 2H), 3.03 (t, *J* = 7.1 Hz, 2H), 1.80 (dd, *J* = 14.3, 7.2 Hz, 2H), 1.56 (dd, *J* = 14.1, 7.2 Hz, 2H), 1.44–1.37 (m, 2H), 1.34–1.26 (m, 2H); ^13^C NMR (125 MHz, DMSO-*d*_6_): *δ* 168.61, 165.28, 163.30, 162.80, 161.86, 157.31, 138.44, 134.87, 133.83, 130.50, 130.43, 125.02, 124.62, 123.97, 116.15, 115.90 (d, *J* = 19.0 Hz), 114.56, 114.05, 113.02, 46.55, 30.24, 29.85, 28.95, 27.86, 26.02; HRMS: *m*/*z* [M + H]^+^ calcd for C_25_H_25_N_5_^81^BrFS, 528.1050; found, 528.1035.

2-(6-(6-bromo-3-(2-(3-fluorophenyl)pyrimidin-4-yl)-1*H*-indol-1-yl)hexyl)isothiouronium (**6e**). Yield: 75%; brown solid; m.p. 124–126 °C; ^1^H NMR (500 MHz, DMSO-*d*_6_): *δ* 9.31 (s, 3H), 8.76 (d, *J* = 5.4 Hz, 1H), 8.59 (s, 1H), 8.53 (d, *J* = 8.5 Hz, 1H), 8.34 (d, *J* = 7.8 Hz, 1H), 8.17 (d, *J* = 10.4 Hz, 1H), 7.92 (d, *J* = 1.3 Hz, 1H), 7.81 (d, *J* = 5.4 Hz, 1H), 7.63 (dd, *J* = 14.0, 7.9 Hz, 1H), 7.45–7.35 (m, 2H), 4.27 (t, *J* = 7.1 Hz, 2H), 3.05 (t, *J* = 7.2 Hz, 2H), 1.81 (dd, *J* = 14.5, 7.3 Hz, 2H), 1.57 (dt, *J* = 14.5, 7.4 Hz, 2H), 1.40 (dt, *J* = 14.5, 7.3 Hz, 2H), 1.35–1.27 (m, 2H); ^13^C NMR (125 MHz, DMSO-*d*_6_): *δ* 168.99, 163.96, 162.50 (d, *J* = 3.0 Hz), 161.97 (d, *J* = 13.8 Hz), 157.38, 140.97 (d, *J* = 7.7 Hz), 138.46, 133.96, 131.26 (d, *J* = 8.1 Hz), 124.99, 124.69, 124.16, 123.83, 117.98, 117.81, 115.86, 115.12, 114.56, 114.38, 114.10, 112.92, 46.56, 30.29, 29.85, 28.90, 27.87, 26.02; HRMS calcd for C_25_H_25_N_5_BrFS [M + H]^+^ 528.1050, found: 528.1056.

2-(6-(6-bromo-3-(2-(p-tolyl)pyrimidin-4-yl)-1*H*-indol-1-yl)hexyl)isothiouronium (**6f**). Yield: 69%; yellow solid; m.p. 130–131 °C; ^1^H NMR (500 MHz, DMSO-*d*_6_): *δ* 9.33 (s, 3H), 8.71 (d, *J* = 5.3 Hz, 1H), 8.61–8.53 (m, 2H), 8.38 (d, *J* = 8.1 Hz, 2H), 7.91 (s, 1H), 7.74 (d, *J* = 5.3 Hz, 1H), 7.39 (dd, *J* = 25.4, 8.2 Hz, 3H), 4.26 (t, *J* = 6.8 Hz, 2H), 3.04 (t, *J* = 7.1 Hz, 2H), 2.40 (s, 3H), 1.86–1.75 (m, 2H), 1.55 (dd, *J* = 14.1, 7.1 Hz, 2H), 1.39 (dd, *J* = 14.2, 7.2 Hz, 2H), 1.31 (d, *J* = 6.7 Hz, 2H); ^13^C NMR (125 MHz, DMSO-*d*_6_): *δ* 168.93, 163.77, 161.74, 157.24, 140.80, 138.42, 135.73, 133.63, 129.76, 128.11, 125.08, 124.56, 124.02, 115.80, 114.36, 114.02, 113.15, 46.53, 30.26, 29.86, 28.91, 27.87, 26.02, 21.50; HRMS calcd for C_26_H_29_N_5_^81^BrS [M + H]^+^ 524.1301, found: 524.1306.

2-(6-(6-bromo-3-(2-(m-tolyl)pyrimidin-4-yl)-1H-indol-1-yl)hexyl)isothiouronium (**6g**). Yield: 80%; brown solid; m.p. 125–127 °C; ^1^H NMR (500 MHz, DMSO-*d*_6_): *δ* 9.19 (s, 3H), 8.73 (d, *J* = 5.4 Hz, 1H), 8.61–8.53 (m, 2H), 8.29 (d, *J* = 7.6 Hz, 2H), 7.92 (d, *J* = 1.3 Hz, 1H), 7.76 (d, *J* = 5.4 Hz, 1H), 7.49–7.41 (m, 2H), 7.36 (d, *J* = 7.6 Hz, 1H), 4.27 (t, *J* = 7.1 Hz, 2H), 3.08 (t, *J* = 7.3 Hz, 2H), 2.44 (s, 3H), 1.82 (dt, *J* = 14.6, 7.3 Hz, 2H), 1.57 (dt, *J* = 14.7, 7.4 Hz, 2H), 1.40 (dt, *J* = 14.6, 7.3 Hz, 2H), 1.30 (dt, *J* = 14.8, 7.4 Hz, 2H); ^13^C NMR (125 MHz, DMSO-*d*_6_): *δ* 169.66, 163.80, 161.76, 157.26, 138.43, 138.35, 138.21, 133.65, 131.70, 129.07, 128.73, 125.34, 125.07, 124.60, 123.99, 115.82, 114.56, 114.04, 113.13, 46.54, 30.38, 29.86, 28.82, 27.87, 26.02, 21.69; HRMS calcd for C_26_H_29_N_5_^81^BrS [M + H]^+^ 524.1301, found: 524.1310.

#### 4.1.5. General Procedure for Synthesis of **6e-1**

To a solution of **4e** in DMF (5 mL) was added 1-bromohexane (5.0 equiv.), and the mixture was stirred at 50 °C for 5 h. Then, the reaction mixture was removed under vacuum, and the residue was poured into ice water and extracted with ethyl acetate (3 × 50 mL). The combined organic layers were washed with brine and dried over anhydrous magnesium sulfate. After filtration, the solvent was removed under vacuum, and the residue was purified by silica gel column chromatography (petroleum ether/ethyl acetate 5:1) to give get the final target compound **6e-1**.

2-(6-(6-bromo-3-(2-(3-fluorophenyl)pyrimidin-4-yl)-1*H*-indol-1-yl)hexyl)isothiouronium bromide (**6e-1**). Yield: 63%; pale yellow solid; m.p. 85–87 °C; ^1^H NMR (400 MHz, DMSO-*d_6_*): *δ* 8.78 (d, *J* = 5.4 Hz, 1H), 8.60 (s, 1H), 8.54 (d, *J* = 8.6 Hz, 1H), 8.36 (d, *J* = 7.9 Hz, 1H), 8.19 (ddd, *J* = 10.6, 2.5, 1.5 Hz, 1H), 7.94 (d, *J* = 1.6 Hz, 1H), 7.82 (d, *J* = 5.5 Hz, 1H), 7.68–7.60 (m, 1H), 7.42 (ddd, *J* = 10.6, 8.3, 1.9 Hz, 2H), 4.29 (t, *J* = 7.1 Hz, 2H), 1.26 (dd, *J* = 16.6, 10.1 Hz, 8H), 0.84 (d, *J* = 7.0 Hz, 3H); ^13^C NMR (100 MHz, DMSO*-d_6_*): *δ* 161.96, 157.41, 141.01, 138.52, 133.99, 131.30, 124.99, 124.70, 124.19, 123.83, 118.04, 117.82, 115.87, 115.11, 114.50, 114.16, 112.91, 46.65, 31.23, 30.03, 26.24, 22.47, 14.33; HRMS: *m*/*z* [M + H]^+^ calcd for C_24_H_24_N_3_BrF, 452.1132; found, 452.1145.

#### 4.1.6. General Procedure for Synthesis of **6e-2**–**6**

To a solution of **4e** in DMF (5 mL) was added 1,2-dibromoethane, 1,3-dibromopropane, 1,4-dibromobutane, 1,5-dibromopentane or 1,7-dibromoheptane (5.0 equiv.), respectively, and the mixture was stirred at 50 °C for 5 h. Then, the reaction mixture was removed under vacuum, and the residue was poured into ice water and extracted with ethyl acetate (3 × 50 mL). The combined organic layers were washed with brine, dried over anhydrous magnesium sulfate and concentrated to give intermediates **37**–**41** and used in the next step without further purification. To a stirring solution of compounds **37**–**41** in ethanol was added thiocarbamide (2.0 equiv.), and the mixture was stirred at 65 °C for 3 h. Then, the solvent was removed under vacuum, and the residue was purified by silica gel column chromatography (dichloromethane/methanol 10:1) to give the final target compounds **6e-2**–**6**.

2-(2-(6-bromo-3-(2-(3-fluorophenyl)pyrimidin-4-yl)-1*H*-indol-1-yl)ethyl)isothiouronium bromide (**6e-2**). Yield: 60%; pale yellow solid; m.p. 194 °C; ^1^H NMR (400 MHz, DMSO-*d_6_*): *δ* 9.15 (s, 2H), 9.01 (d, *J* = 25.1 Hz, 2H), 8.83 (d, *J* = 5.4 Hz, 1H), 8.62 (s, 1H), 8.54 (d, *J* = 8.6 Hz, 1H), 8.36 (d, *J* = 7.9 Hz, 1H), 8.22–8.17 (m, 1H), 8.02 (d, *J* = 1.6 Hz, 1H), 7.81 (d, *J* = 5.4 Hz, 1H), 7.65 (td, *J* = 8.0, 6.1 Hz, 1H), 7.49 (dd, *J* = 8.6, 1.7 Hz, 1H), 7.43 (td, *J* = 8.3, 2.2 Hz, 1H), 4.64 (t, *J* = 6.4 Hz, 2H), 3.79–3.67 (m, 2H); ^13^C NMR (100 MHz, DMSO-*d_6_*): *δ* 169.30, 162.59, 161.71, 157.67, 140.89, 138.43, 134.21, 131.42, 131.33, 125.04, 124.22, 123.77, 118.15, 117.94, 116.20, 115.24, 114.62, 114.37, 113.46, 45.41, 30.84; HRMS: *m*/*z* [M + H]^+^ calcd for C_21_H_18_N_5_^81^BrFS, 472.0424; found, 472.0427.

2-(3-(6-bromo-3-(2-(3-fluorophenyl)pyrimidin-4-yl)-1*H*-indol-1-yl)propyl)isothiouronium bromide (**6e-3**). Yield: 45%; pale yellow solid; m.p. 175–177 °C; ^1^H NMR (400 MHz, DMSO-*d_6_*): *δ* 9.12 (s, 2H), 8.98 (s, 2H), 8.80 (d, *J* = 5.4 Hz, 1H), 8.61 (s, 1H), 8.56 (d, *J* = 8.6 Hz, 1H), 8.36 (d, *J* = 7.9 Hz, 1H), 8.22–8.16 (m, 1H), 7.97 (d, *J* = 1.6 Hz, 1H), 7.83 (d, *J* = 5.5 Hz, 1H), 7.65 (td, *J* = 8.0, 6.1 Hz, 1H), 7.47 (dd, *J* = 8.6, 1.7 Hz, 1H), 7.42 (td, *J* = 8.3, 2.1 Hz, 1H), 4.41 (t, *J* = 6.9 Hz, 2H), 3.24–3.16 (m, 2H), 2.27–2.14 (m, 2H); ^13^C NMR (100 MHz, DMSO-*d_6_*): *δ* 170.02, 164,18, 162.52, 161.86, 157.51, 140.98, 140.83, 138.49, 133.91, 131.39, 124.99, 124.22, 123.91, 117.90, 116.06, 115.24, 114.06, 113.32, 45.32, 29.73, 27.85; HRMS: *m*/*z* [M + H]^+^ calcd for C_22_H_20_N_5_^81^BrFS, 486.0581; found, 486.0583.

2-(4-(6-bromo-3-(2-(3-fluorophenyl)pyrimidin-4-yl)-1*H*-indol-1-yl)butyl)isothiouronium (**6e-4**). Yield: 64%; pale yellow solid; m.p. 171–173 °C; ^1^H NMR (400 MHz, DMSO-*d_6_*): *δ* 9.05 (s, 2H), 8.95 (s, 2H), 8.79 (d, *J* = 5.5 Hz, 1H), 8.64 (s, 1H), 8.55 (d, *J* = 8.6 Hz, 1H), 8.36 (d, *J* = 7.9 Hz, 1H), 8.19 (ddd, *J* = 10.5, 2.5, 1.4 Hz, 1H), 7.99 (d, *J* = 1.6 Hz, 1H), 7.83 (d, *J* = 5.5 Hz, 1H), 7.65 (td, *J* = 8.0, 6.1 Hz, 1H), 7.49–7.44 (m, 1H), 7.41 (dd, *J* = 8.5, 2.2 Hz, 1H), 4.40–4.30 (m, 2H), 3.23 (dd, *J* = 15.2, 8.0 Hz, 2H), 1.99–1.89 (m, 2H), 1.64 (dt, *J* = 14.9, 7.6 Hz, 2H); ^13^C NMR (100 MHz, DMSO): *δ* 170.17, 162.52, 161.92, 161.80, 157.42, 140.93, 138.48, 134.04, 131.38, 124.93, 124.23, 123.88, 118.06, 117.89, 116.00, 115.14, 114.59, 114.19, 113.05, 46.05, 30.06, 28.88, 26.37; HRMS: *m*/*z* [M + H]^+^ calcd for C_23_H_22_N_5_^81^BrFS, 500.0737; found, 500.0740.

2-(5-(6-bromo-3-(2-(3-fluorophenyl)pyrimidin-4-yl)-1*H*-indol-1-yl)pentyl)isothiouronium (**6e-5**). Yield: 67%; pale yellow solid; m.p. 155–157 °C; ^1^H NMR (400 MHz, DMSO-*d_6_*): *δ* 9.02 (s, 2H), 8.92 (s, 2H), 8.78 (t, *J* = 7.4 Hz, 1H), 8.64 (s, 1H), 8.55 (d, *J* = 8.6 Hz, 1H), 8.36 (d, *J* = 7.9 Hz, 1H), 8.19 (ddd, *J* = 10.5, 2.5, 1.5 Hz, 1H), 7.96 (d, *J* = 1.6 Hz, 1H), 7.83 (d, *J* = 5.5 Hz, 1H), 7.65 (td, *J* = 8.0, 6.1 Hz, 1H), 7.45 (dd, *J* = 8.6, 1.7 Hz, 1H), 7.41 (dd, *J* = 8.3, 2.2 Hz, 1H), 4.37–4.28 (m, 2H), 3.15–3.11 (m, 3H), 1.87 (dd, *J* = 14.8, 7.3 Hz, 2H), 1.65 (dd, *J* = 14.5, 7.4 Hz, 3H), 1.43–1.37 (m, 2H); ^13^C NMR (101 MHz, DMSO-*d_6_*): *δ* 170.30, 162.53, 161.96, 157.41, 140.96, 138.52, 134.06, 131.37, 125.01, 124.77, 124.22, 123.85, 117.87, 115.93, 115.13, 114.41, 114.40, 114.13, 112.96, 46.48, 30.32, 29.47, 28.52, 25.56; HRMS: *m*/*z* [M + H]^+^ calcd for C_24_H_24_N_5_^81^BrFS, 514.0894; found, 514.0898.

2-(7-(6-bromo-3-(2-(3-fluorophenyl)pyrimidin-4-yl)-1*H*-indol-1-yl)heptyl)isothiouronium (**6e-6**). Yield: 56%; pale yellow solid; m.p. 121–123 °C; ^1^H NMR (400 MHz, DMSO-*d_6_*): *δ* 9.00 (s, 2H), 8.90 (s, 2H), 8.79 (d, *J* = 5.4 Hz, 1H), 8.62 (s, 1H), 8.55 (d, *J* = 8.6 Hz, 1H), 8.36 (d, *J* = 7.9 Hz, 1H), 8.22–8.16 (m, 1H), 7.95 (d, *J* = 1.6 Hz, 1H), 7.83 (d, *J* = 5.5 Hz, 1H), 7.65 (td, *J* = 8.0, 6.1 Hz, 1H), 7.46 (dd, *J* = 8.6, 1.7 Hz, 1H), 7.41 (dd, *J* = 8.3, 2.2 Hz, 1H), 4.31 (t, *J* = 7.0 Hz, 2H), 3.11 (t, *J* = 7.3 Hz, 2H), 1.88–1.79 (m, 2H), 1.57 (d, *J* = 6.7 Hz, 2H), 1.32 (d, *J* = 11.1 Hz, 6H); ^13^C NMR (100 MHz, DMSO-*d_6_*): *δ* 170.27, 161.96, 157.45, 141.50, 140.90, 138.52, 134.05, 131.41, 125.24, 124.87, 124.22, 123.86, 118.15, 117.84, 115.90, 115.12, 114.63, 114.19, 112.91, 46.61, 30.47, 30.00, 28.70, 28.38, 28.16, 26.42; HRMS: *m*/*z* [M + H]^+^ calcd for C_26_H_28_N_5_^81^BrFS, 542.1207; found, 542.1206.

### 4.2. Biological Evaluation

Antibodies against p-Tyr1022/1023-JAK1, p-Tyr1007/1008-JAK2, p-Tyr980/981-JAK3, p-Tyr1054/1055-TYK2, pTyr705-STAT3, STAT3, c-Myc, cyclin D1 and Bcl-XL were obtained from Cell Signaling Technology (Beverly, MA, USA), and antibodies against α-tubulin and GAPDH were purchased from Santa Cruz Biotechnology (Santa Cruz, CA, USA). Transfection reagent, protease inhibitor and phosphatase inhibitor were purchased from Millipore (Billerica, MA, USA). Polyvinylidene difluoride (PVDF) membranes and chemiluminescent horseradish peroxidase (HRP) substrate were purchased from Millipore (Billerica, MA, USA). Gefitinib was acquired from Selleckchem (Houston, TX, USA).

In vitro inhibitory activity. The resazurin indicator was used to evaluate the cell viability. HeLa, MDA-MB-231, A549, DU145, HUVEC, L02, L929 and MCF10A cells were seeded in 96-well plates in 50 µL at plating densities ranging from 4000 to 8000 cells/well, depending on the doubling time of individual cell lines. After incubation for 24 h, different concentrations of compounds were added, and then, the cells were further cultured for 72 h with 0.5% DMSO as the solvent control group, and 10 µL of resazurin solution (1 mg/mL) was directly added to each well as a redox indicator. Plates were incubated for 3 h to measure the absorbance of a SpectraMax@i3 (Molecular Devices, Madison, WI, USA) of each well at a 595-nm emission wavelength (549-nm excitation wavelength). Each treatment was performed in triplicate to reduce the experimental error. Results were analyzed with GraphPad Prism 6, and the data were shown as the mean ± SD.

Molecular Docking. All calculations were performed using the Molecular Docking program of MOE (version MOE 2020.09). The crystal structures of the proteins involved in this article were retrieved from the Protein Data Bank (PDB). Firstly, all compounds were treated through energy minimization. The parameters and charges were assigned with the MMFF94x force field. Secondly, after removing water molecules, each selected protein structure was treated by adding hydrogen atoms. Finally, the small molecules were docked into the pockets of the proteins defined by the originally bound ligands in the crystal structures, respectively. The poses were ranked by the scores from the GBVI/WSA-binding free energy calculations, and the results were analyzed using Pymol (1.8) (https://pymol.org/2/, accessed on 23 November 2021).

Colony formation assay. The colony formation assay was performed to examine the effect of compound **6e** on cell colony survival. DU145 and A549 cells were seeded in 6-well plates with 500~1000 cells/well. The second day, various concentrations of **6e** were added. After that, the cell culture medium was changed, and the corresponding concentration of **6e** was added every 2 days until the colonies were visible. About 14 days later, the cells were fixed using 4% paraformaldehyde fix solution (Beyotime, Shanghai, China) and stained with crystal violet (Beyotime). Then, we observed and calculated the number of colonies.

Flow cytometry analysis of apoptotic cells. An Annexin V-FITC/PI apoptosis kit (Invitrogen) was used to detect cell apoptosis. A549 and DU145 cells were cells at a density of 5 × 10^5^ per well cultured in regular growth medium in 6-well plates for 24 h and disposed induplicate with various concentrations of compound **6e** for 24 h. After 48 h later, A549 and DU145 were trypsinized, centrifuged and washed with precooled PBS twice with an Annexin V-FITC/PI apoptosis kit (Invitrogen) following the manufacturer’s instructions.

Western blot analysis. A549 and DU145 cells were plated in 6-well plates and cultured overnight, respectively, and different concentrations of compound **6e** were added for 2 h. The corresponding cells were collected, washed with PBS and lysed with cell lysis buffer to extract the total proteins. The extracted protein was loaded and subjected to SDS-PAGE electrophoresis, and then, the protein was transferred to a PDVF membrane and incubated in the corresponding primary antibody overnight. The next day, the primary antibody was recovered and labeled, and the corresponding secondary antibody was incubated. The immune complexes were detected using chemiluminescence HRP substrate (Millipore) and visualized by the Tanon 5200 Chemiluminescence Imaging System (Bio-tanon, Shanghai, China).

In vivo studies. Six-week-old male nude mice (SPF degree, 17–20 g weight, nu/nu) were obtained from Beijing Vital River Laboratory Animal Technology Co. Ltd. (Beijing, China). Nude mice were injected into the back with DU145 tumor cells (about 15 × 10^6^). After 2 weeks, the mice were randomly divided into four groups: blank control group (NC, DMSO), positive control Gefitinib group (PC, 100 mg/kg), compound **6e** group (5 mg/kg) and compound **6e** group (10 mg/kg), with 6 mice per group. The compound **6e** groups and the PC group were intraperitoneally injected or intragastric-administered every two days until the mice were sacrificed. The body weights of the nude mice were recorded every three days, and the tumor weights were recorded on the day of death of the nude mice All of the procedures were approved by the Committee of Experimental Animals of the Ocean University of China and conformed to the Guide for the Care and Use of Laboratory Animals published by the United States National Institutes of Health (NIH Publication No. 85-23, revised 1996).

Immunohistochemistry (IHC) analyses. The mouse tumor tissues were collected, fixed in 4% PFA for 72 h at 4 °C, embedded in paraffin and cut into sections. The sections were deparaffinized in xylene, rehydrated in graded ethanol, boiled in antigen retrieval solution [31] and then incubated with fresh 3% H_2_O_2_ to inactivate endogenous peroxidase. After PBS washing, the slides were blocked with fatty free milk and incubated with the primary antibody at 4 °C overnight, followed by incubation with the HRP-conjugated secondary antibody at room temperature (Boster, Wuhan, China), according to the manufacturer’s instructions. Finally, DAB color developing solution was added dropwise. A brown color in the cell membrane indicated positive staining. Images were captured using an upright fluorescence microscope (Olympus BX53, Tokyo, Japan). For hematoxylin and eosin staining (H&E staining), the tumor sections were incubated in hematoxylin solution and then counterstained with eosin.

Statistical analysis. Data were reported as the mean ± SEM. Statistical analyses and significance as measured by repeated measures ANOVA (followed by Dunnett’s posttest or Friedman test) were obtained using GraphPad Prism version 7.0 (GraphPad Software Inc., San Diego, CA, USA). *p* < 0.05 was considered significant.

## Figures and Tables

**Table 1:** Antiproliferative activity of meridianins A, C, D, G and their derivatives against human cancer cell lines.

Cpd.	IC_50_ ± SD (μM) ^a^			
	HeLa	MDA-MB-231	A549	DU145
**1a**	4.50 ± 0.41	4.39 ± 0.23	10.86 ± 0.52	39.16 ± 2.21
**1b**	11.72 ± 2.14	4.75 ± 0.28	>100	46.92 ± 1.31
**1c**	2.33 ± 0.03	4.23 ± 0.68	7.46 ± 0.54	12.61 ± 2.07
**1d**	4.35 ± 0.20	5.25 ± 0.27	11.45 ± 0.95	16.71 ± 2.28
**1e**	6.18 ± 0.16	>100	7.10 ± 0.01	>100
**1f**	>100	14.48 ± 0.59	8.84 ± 0.45	23.88 ± 2.82
**1g**	1.56 ± 0.09	8.57 ± 0.65	7.11 ± 0.31	11.23 ± 0.65
**2a**	3.76 ± 0.28	8.46 ± 1.24	7.63 ± 0.73	34.17 ± 3.72
**2b**	4.16 ± 0.11	8.59 ± 0.64	9.40 ± 0.64	29.15 ± 3.45
**2c**	2.15 ± 0.11	3.03 ± 0.50	4.25 ± 0.23	17.79 ± 0.83
**2d**	9.05 ± 0.61	13.11 ± 1.51	28.74 ± 8.43	>100
**2e**	2.86 ± 0.27	5.21 ± 0.24	5.12 ± 0.23	13.70 ± 4.37
**2f**	1.81 ± 0.04	8.13 ± 0.15	3.50 ± 0.08	8.86 ± 0.68
**2g**	2.70 ± 0.18	35.22 ± 2.40	17.15 ± 1.41	22.61 ± 2.47
**3a**	2.99 ± 0.44	11.30 ± 1.38	8.95 ± 0.68	19.08 ± 1.19
**3b**	4.86 ± 0.16	6.25 ± 0.34	7.73 ± 0.38	10.46 ± 0.45
**3c**	4.95 ± 0.04	7.46 ± 1.76	15.78 ± 1.11	27.57 ± 3.75
**3d**	26.63 ± 1.05	25.48 ± 1.70	>100	>100
**3e**	6.58 ± 0.16	9.69 ± 0.72	9.75 ± 0.87	12.85 ± 0.56
**3f**	7.37 ± 1.22	>100	10.02 ± 2.05	45.16 ± 2.44
**3g**	25.04 ± 0.82	>100	>100	>100
**4a**	25.07 ± 3.57	20.23 ± 2.09	25.07 ± 3.57	>100
**4b**	8.05 ± 0.85	8.60 ± 0.64	21.68 ± 1.70	>100
**4c**	2.55 ± 0.33	4.76 ± 0.37	9.49 ± 0.10	24.65 ± 0.50
**4d**	10.56 ± 0.42	9.06 ± 0.32	22.50 ± 2.78	18.08 ± 2.44
**4e**	17.53 ± 1.05	27.46 ± 1.26	>100	32.60 ± 1.42
**4f**	5.03 ± 0.70	8.56 ± 0.87	11.84 ± 1.29	26.01 ± 2.83
**4g**	1.67 ± 0.11	4.23 ± 0.06	10.04 ± 0.44	19.81 ± 0.28
Meridianin A	25.39 ± 0.35	>100	>100	>100
Meridianin C	24.07 ± 0.16	14.03 ± 2.63	23.54 ± 3.20	>100
Meridianin D	13.17 ± 2.71	>100	26.68 ± 0.63	>100
Meridianin G	22.52 ± 3.55	>100	>100	>100

^a^ The inhibitory effects of the compounds on the proliferation of the four cell lines were determined by the MTT assay. Results are expressed as the means ± SD. All experiments were independently performed at least three times.

**Table 2:** Antiproliferative activity of meridianin (D and G) derivatives against human cancer cell lines.

Cpd.	IC_50_ ± SD (μM) ^a^			
	HeLa	MDA-MB-231	A549	DU145
**5a**	14.56 ± 2.05	18.61 ± 1.10	8.68 ± 0.27	22.70 ± 2.03
**5b**	4.21 ± 0.15	4.67 ± 0.17	2.68 ± 0.30	6.04 ± 0.10
**5c**	7.53 ± 0.41	7.99 ± 0.82	8.85 ± 0.06	18.56 ± 3.40
**5d**	6.92 ± 0.23	5.46 ± 0.10	5.76 ± 0.12	6.67 ± 0.21
**5e**	8.31 ± 1.00	5.87 ± 0.21	8.23 ± 0.64	10.20 ± 0.15
**5f**	4.70 ± 0.08	2.95 ± 0.11	5.84 ± 0.25	6.47 ± 0.28
**5g**	2.19 ± 0.09	1.92 ± 0.11	3.65 ± 0.07	3.86 ± 0.31
**6a**	3.28 ± 0.08	4.59 ± 0.13	4.81 ± 0.21	5.04 ± 0.06
**6b**	9.23 ± 0.99	6.80 ± 1.33	6.94 ± 0.07	8.91 ± 0.55
**6c**	3.49 ± 0.13	3.20 ± 0.02	5.00 ± 0.31	5.46 ± 0.09
**6d**	7.73 ± 0.26	5.61 ± 0.16	9.86 ± 0.46	7.01 ± 0.32
**6e**	1.11 ± 0.04	1.22 ± 0.34	2.80 ± 0.18	1.13 ± 0.17
**6f**	9.34 ± 0.72	4.89 ± 0.16	7.32 ± 0.35	4.74 ± 0.03
**6g**	2.42 ± 0.15	5.51 ± 0.03	8.93 ± 1.07	6.38 ± 0.31
Gefitinib	7.51 ± 0.45	32.65 ± 0.31	13.08 ± 0.28	9.18 ± 0.06

^a^ The inhibitory effects of the compounds on the proliferation of the four indicated cell lines were determined by the MTT assay. Results are expressed as the means ± SD. All experiments were independently performed at least three times.

**Table 3:** Antiproliferative activity of compound **6e** analogs against human cancer cell lines.

Cpd.	IC_50_ ± SD (μM) ^a^			
	HeLa	MDA-MB-231	A549	DU145
**6e-1**	>100	>100	>100	>100
**6e-2**	>100	24.31 ± 0.77	>100	29.18 ± 0.85
**6e-3**	11.28 ± 0.77	9.65 ± 0.30	9.49 ± 1.18	15.30 ± 3.10
**6e-4**	8.19 ± 0.08	8.38 ± 1.07	8.85 ± 0.30	10.73 ± 0.91
**6e-5**	4.92 ± 0.09	7.88 ± 0.11	7.80 ± 0.19	7.55 ± 0.73
**6e-6**	12.08 ± 1.26	14.21 ± 0.37	10.42 ± 0.27	10.32 ± 2.16

^a^ The inhibitory effects of the compounds on the proliferation of the four cell lines were determined by the MTT assay. Results are expressed as the means ± SD. All experiments were independently performed at least three times.

**Table 4:** Antiproliferative activity of compound **6e** analogs against normal cell lines.

Cpd.	IC_50_ ± SD (μM) ^a^			
	HUVEC	L02	L929	MCF10A
**6e**	26.02 ± 2.16	>100	29.22 ± 1.47	41.64 ± 1.62

^a^ The inhibitory effect of the compound **6e** on the proliferation of the four cell lines was determined by the MTT assay. Results are expressed as the means ± SD. All experiments were independently performed at least three times.

## Data Availability

The authors declare that (the/all other) data supporting the findings of this study are available within the article (and its Supplementary Materials).

## Supplementary Materials

The following are available online at https://www.mdpi.com/article/10.3390/ijms23042199/s1.
