# Supplementary material for: Design, Synthesis and Structure-Activity Relationship Studies of Meridianin Derivatives as Novel JAK/STAT3 Signaling Inhibitors
Source: Int J Mol Sci. 2022 Feb 16;23(4):2199. doi: 10.3390/ijms23042199 (PMC8875316; doi:10.3390/ijms23042199)
Supplement: Supplementary file 1 [file ijms-23-02199-s001.zip › ijms-1585814-supplementary.pdf]

Supplementary Data for

This word file includes Figure S1, Copies of ^{1}H, ^{13}C NMR and HRMS spectra of the target products.

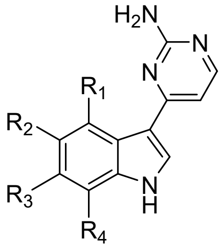

Meridianin A: R_1=OH, R_2=R_3=R_4=H
Meridianin B: R_1=OH, R_2=R_4=H, R_3=Br
Meridianin C: R_1=R_3=R_4=H, R_2=Br
Meridianin D: R_1=R_2=R_4, R_3=Br
Meridianin E: R_1=OH, R_2=R_3=H, R_4=Br
Meridianin F: R_1=R_4=H, R_2=R_3=Br
Meridianin G: R_1=R_2=R_3=R_4=H

Figure. S1. Structures of meridianins A-G

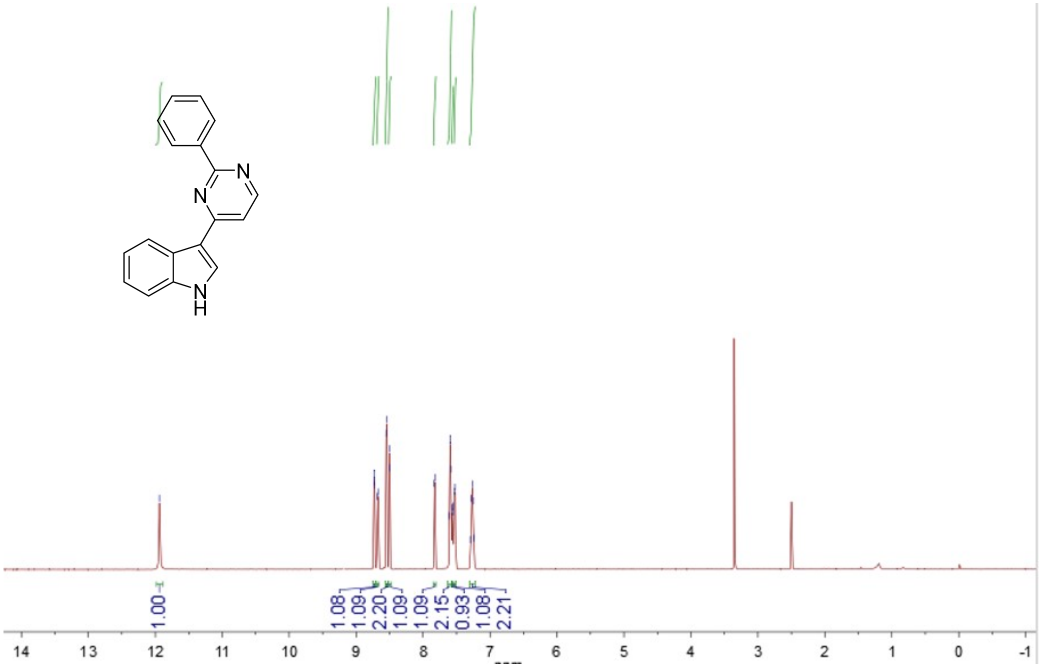

^{1}H NMR Spectrum of **1a**

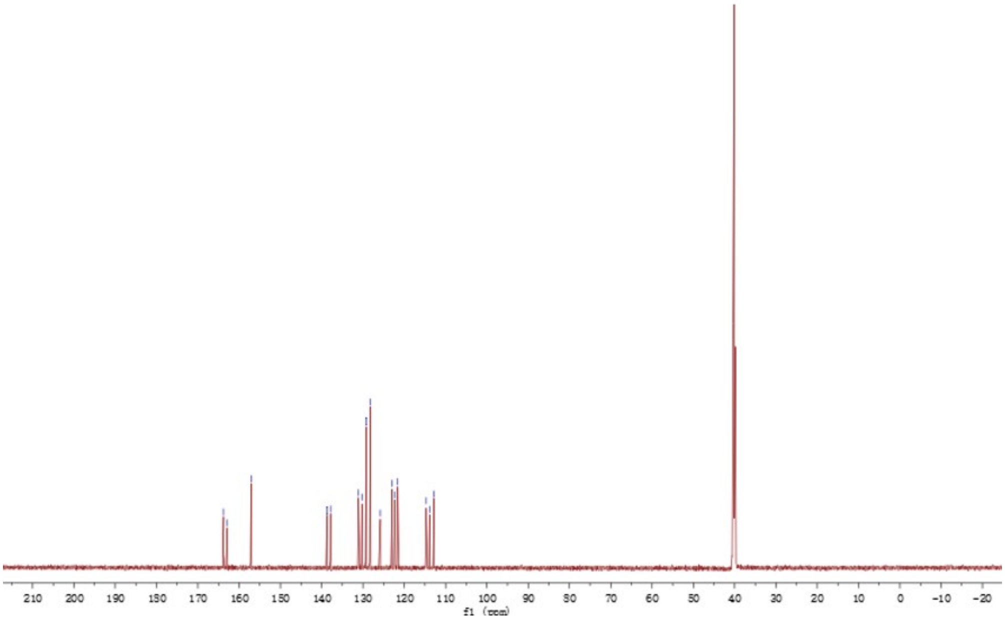

^{13}C NMR Spectrum of **1a**

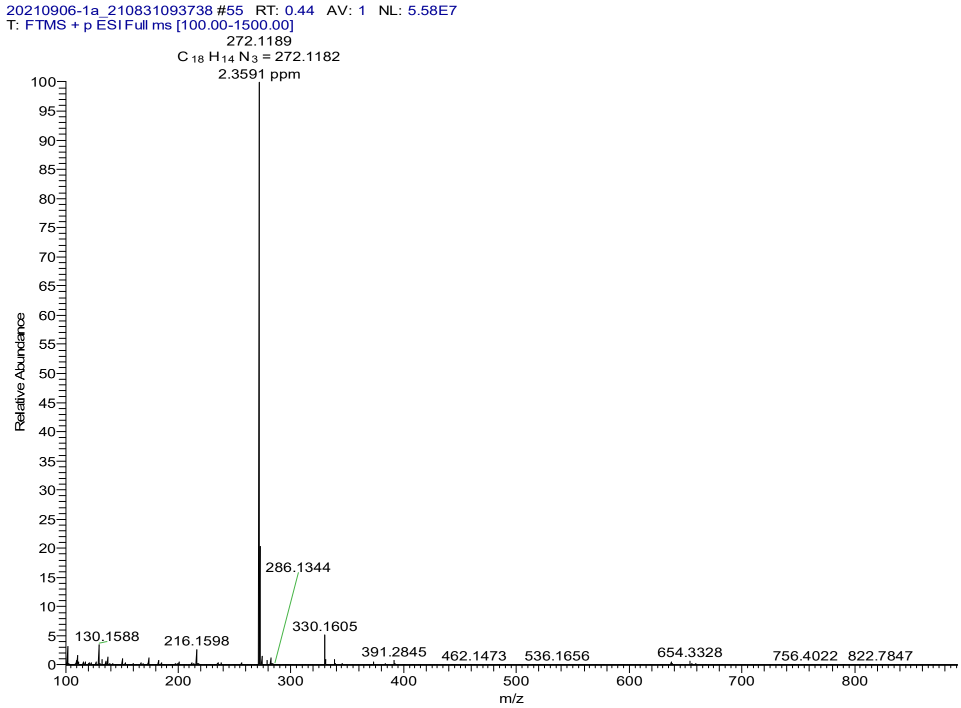

HRMS spectrum of **1a**

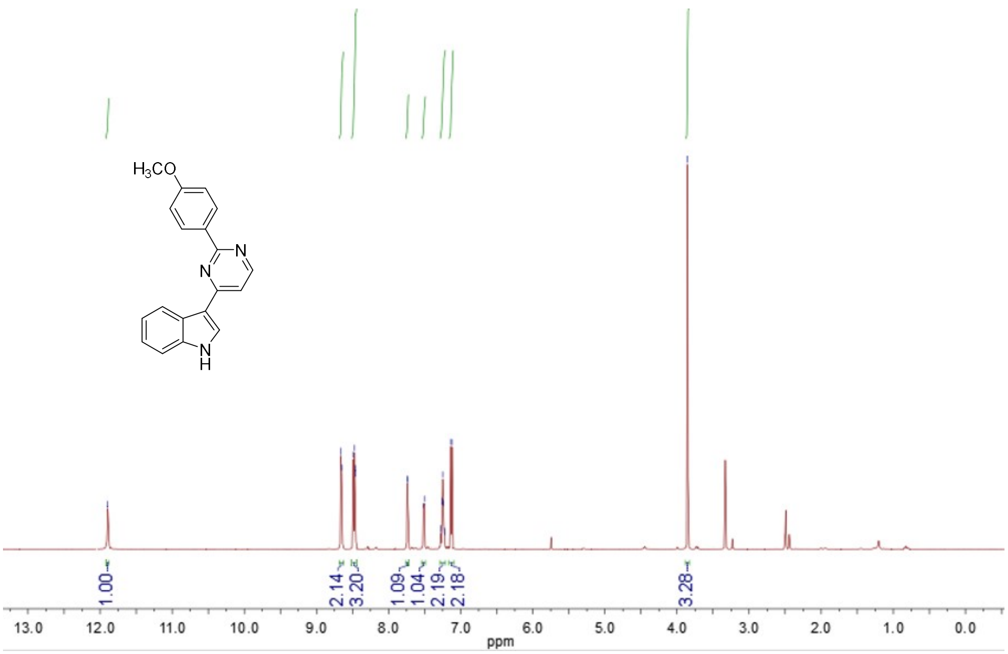

^{1}H NMR Spectrum of **1b**

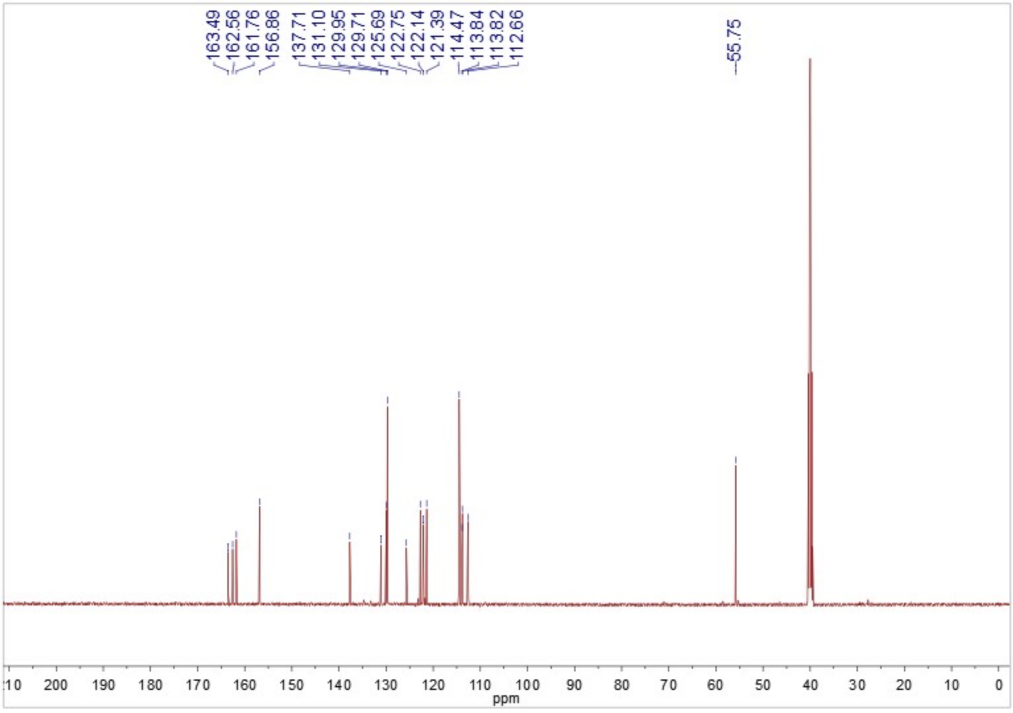

^{13}C NMR Spectrum of **1b**

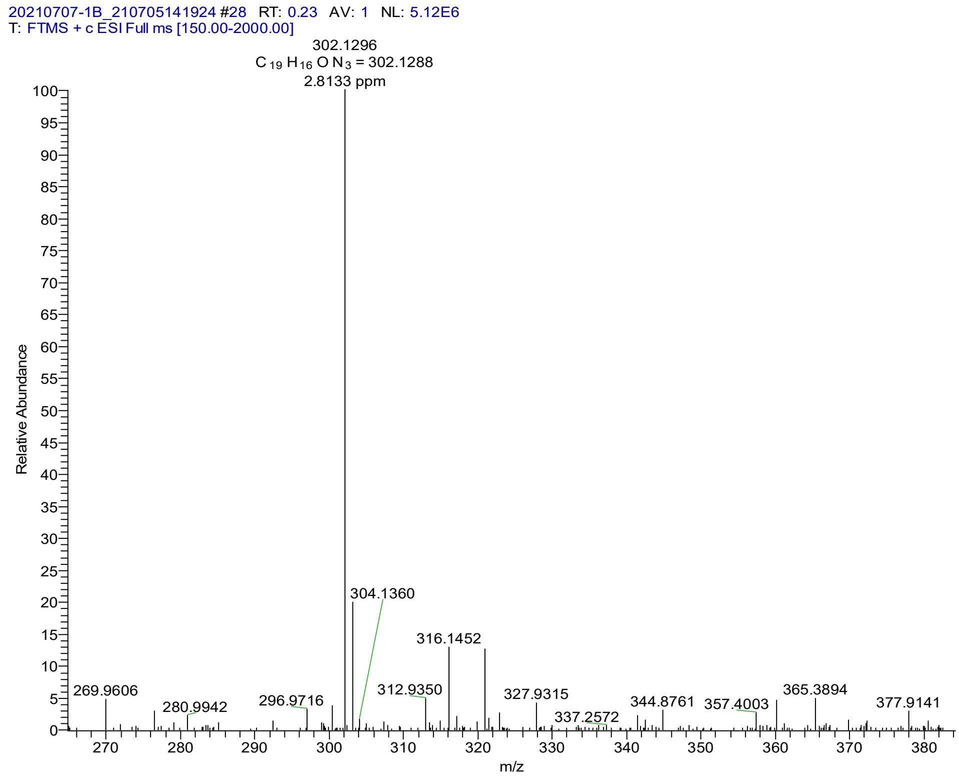

HRMS spectrum of **1b**

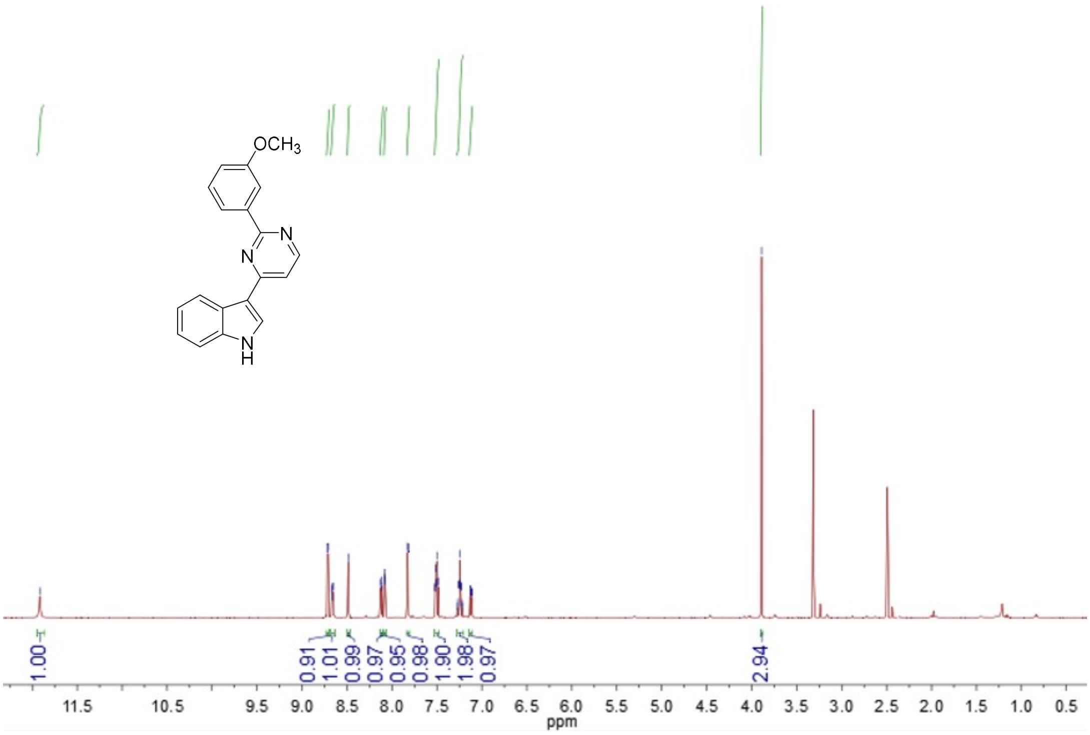

^{1}H NMR Spectrum of **1c**

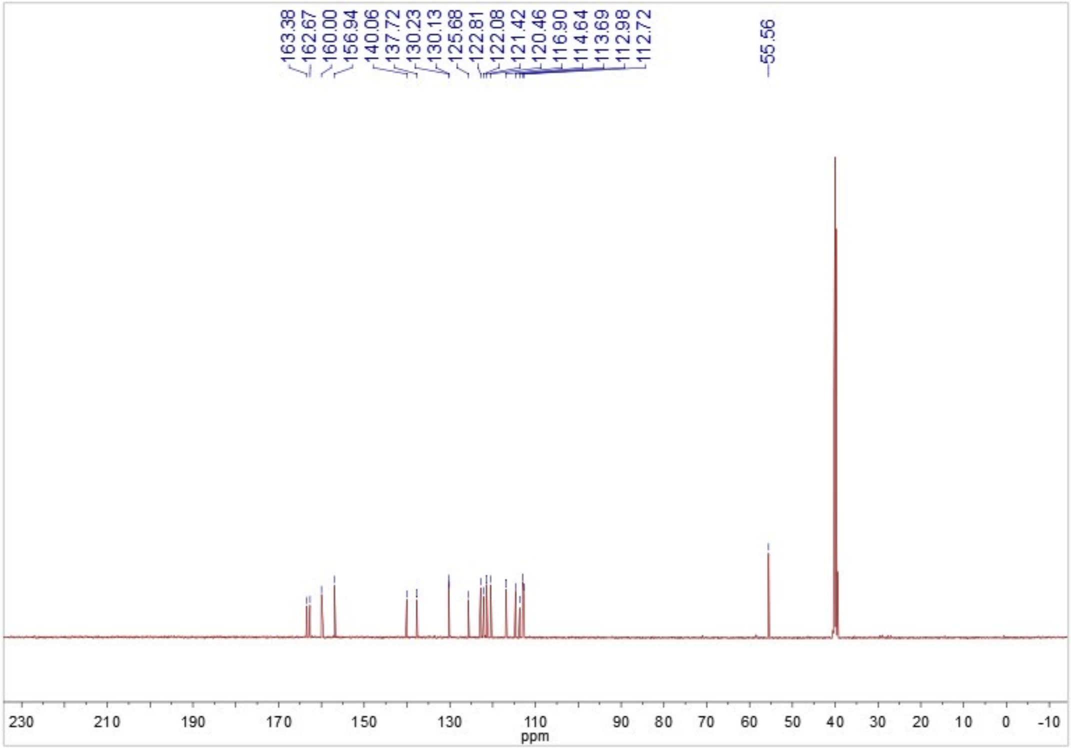

^{13}C NMR Spectrum of **1c**

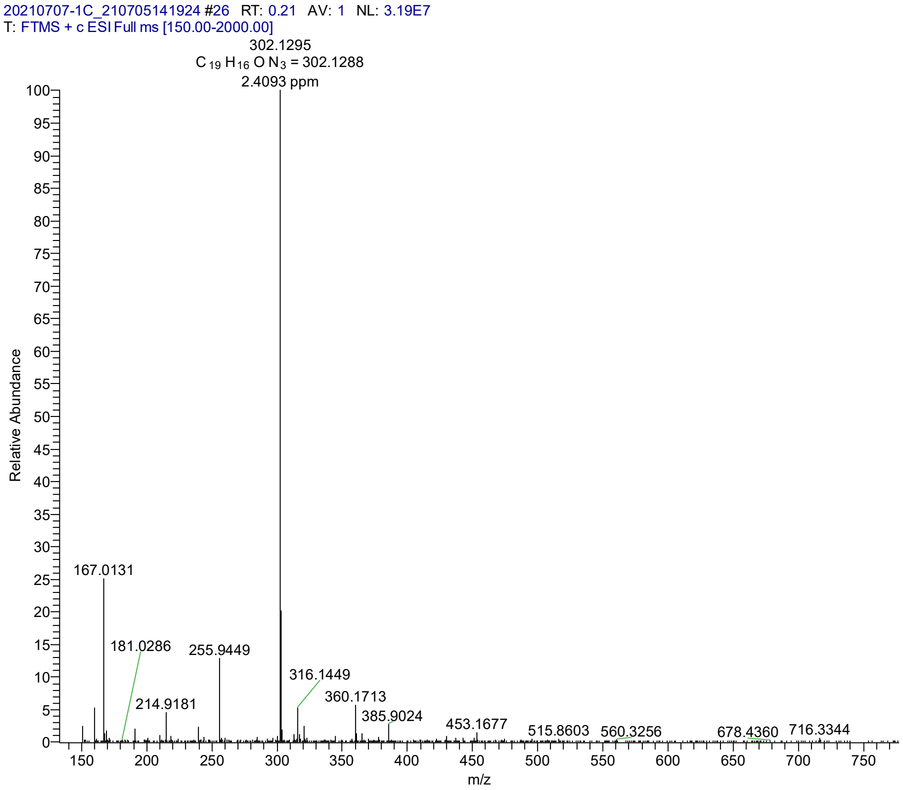

HRMS spectrum of **1c**

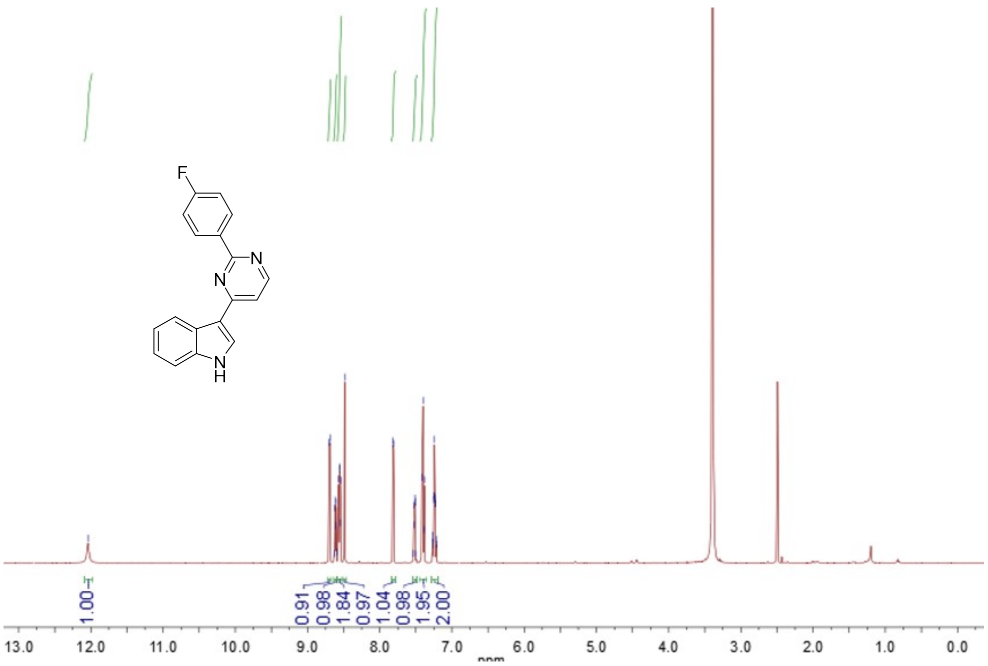

^{1}H NMR Spectrum of **1d**

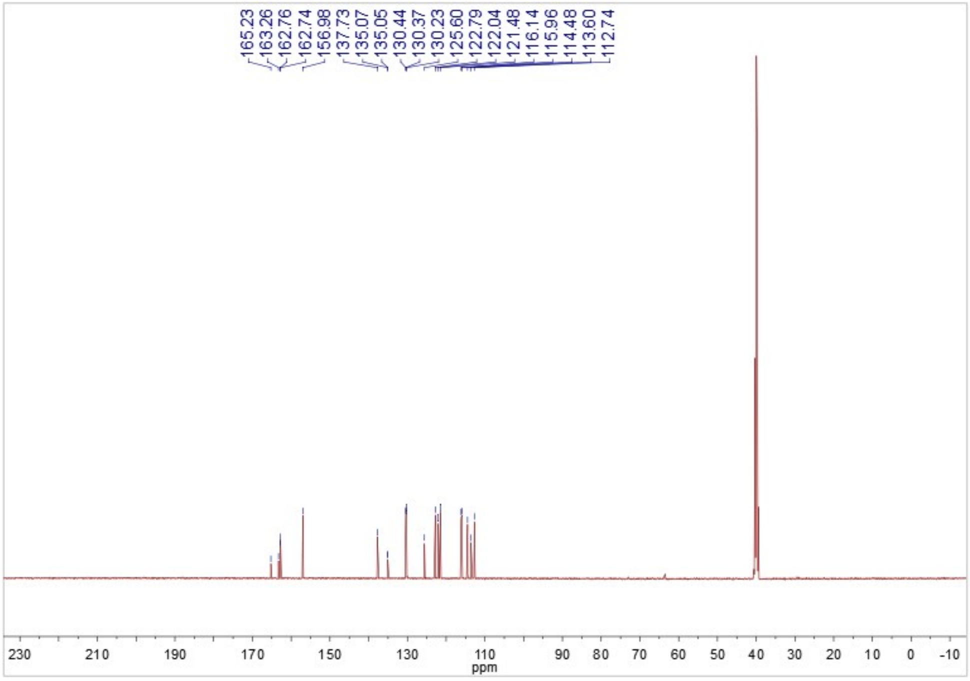

^{13}C NMR Spectrum of **1d**

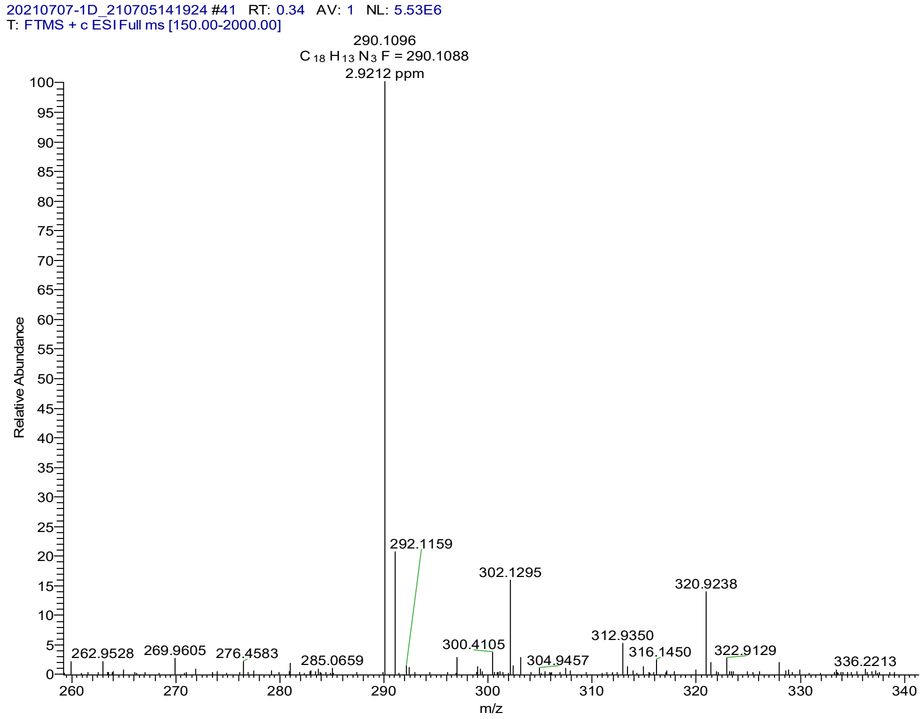

HRMS spectrum of **1d**

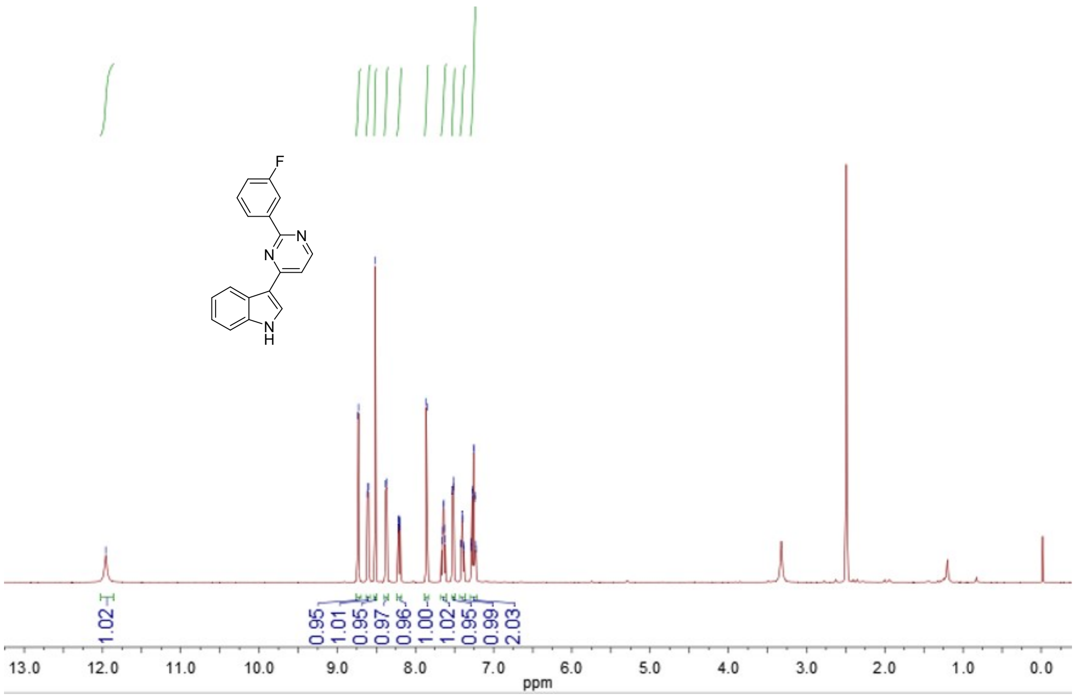

^{1}H NMR Spectrum of **1e**

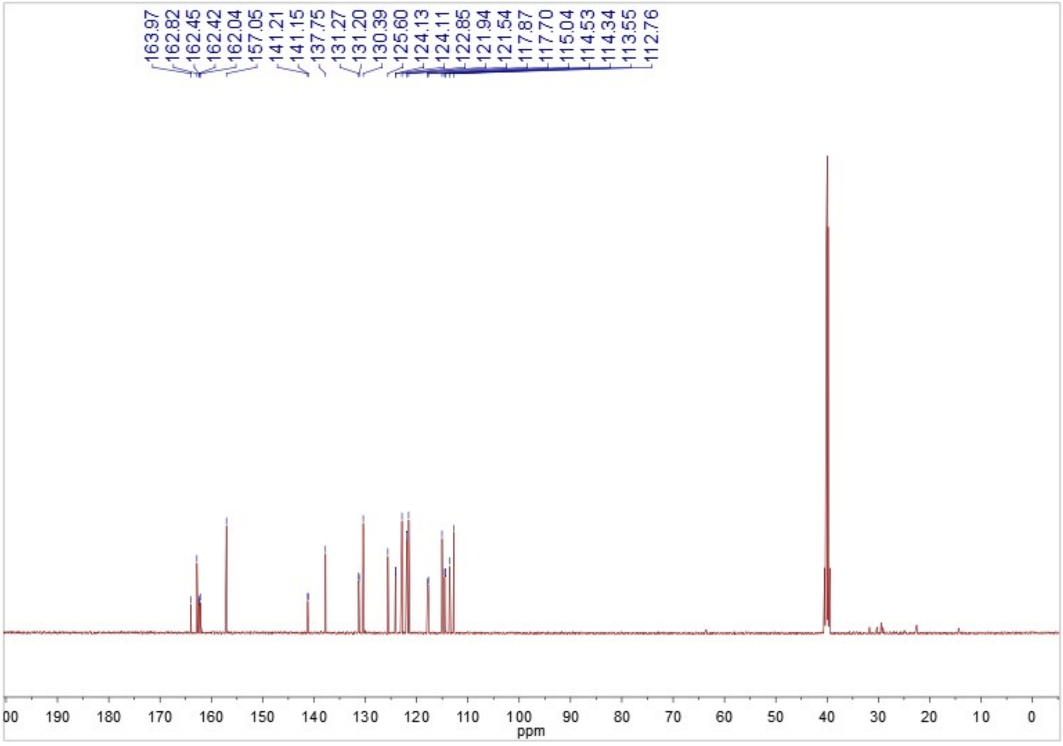

^{13}C NMR Spectrum of **1e**

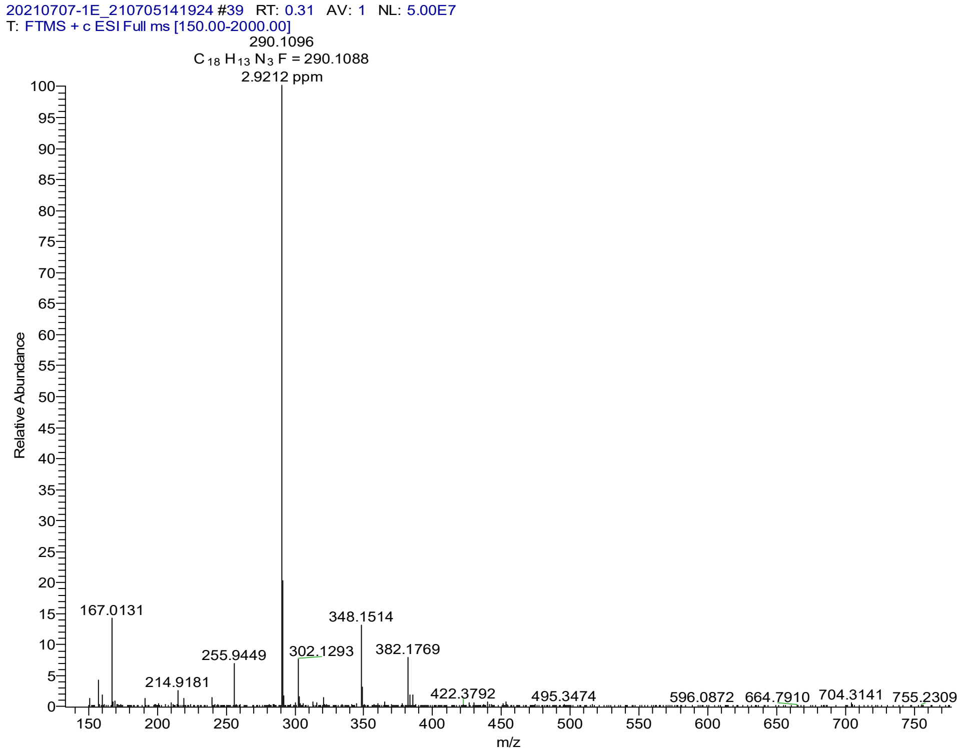

HRMS spectrum of **1e**

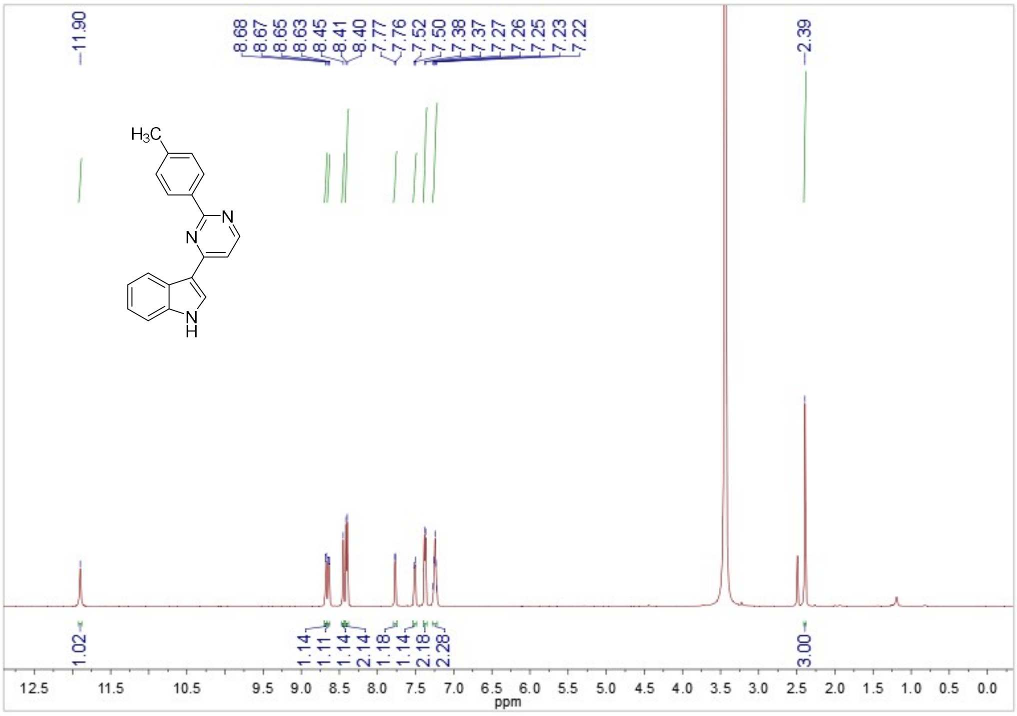

^{1}H NMR Spectrum of **1f**

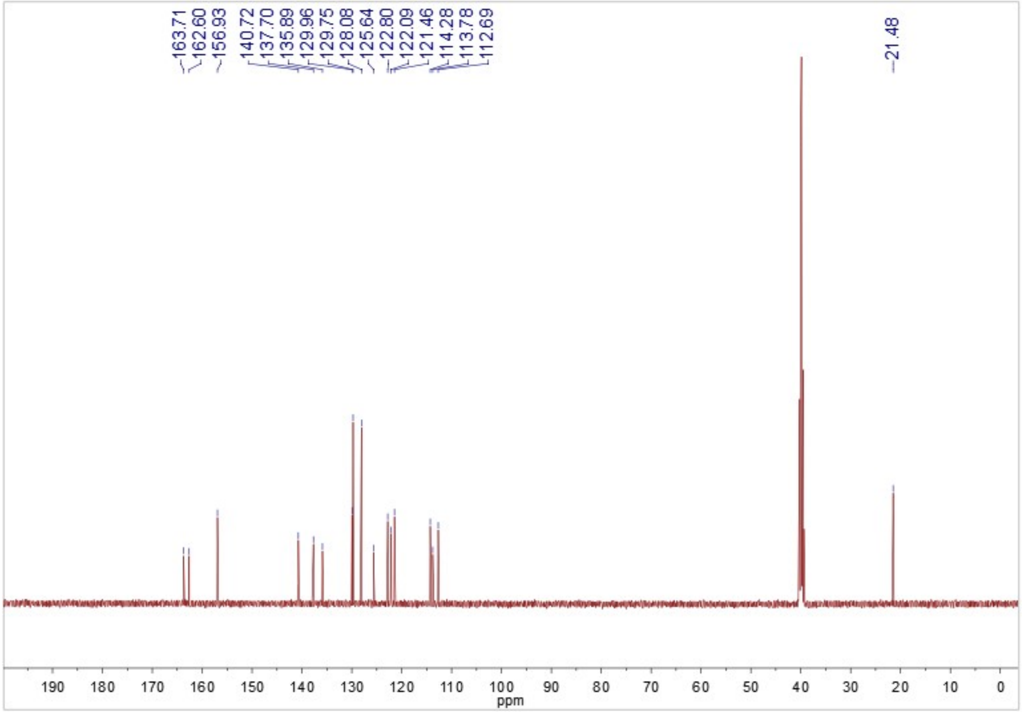

^{13}C NMR Spectrum of **1f**

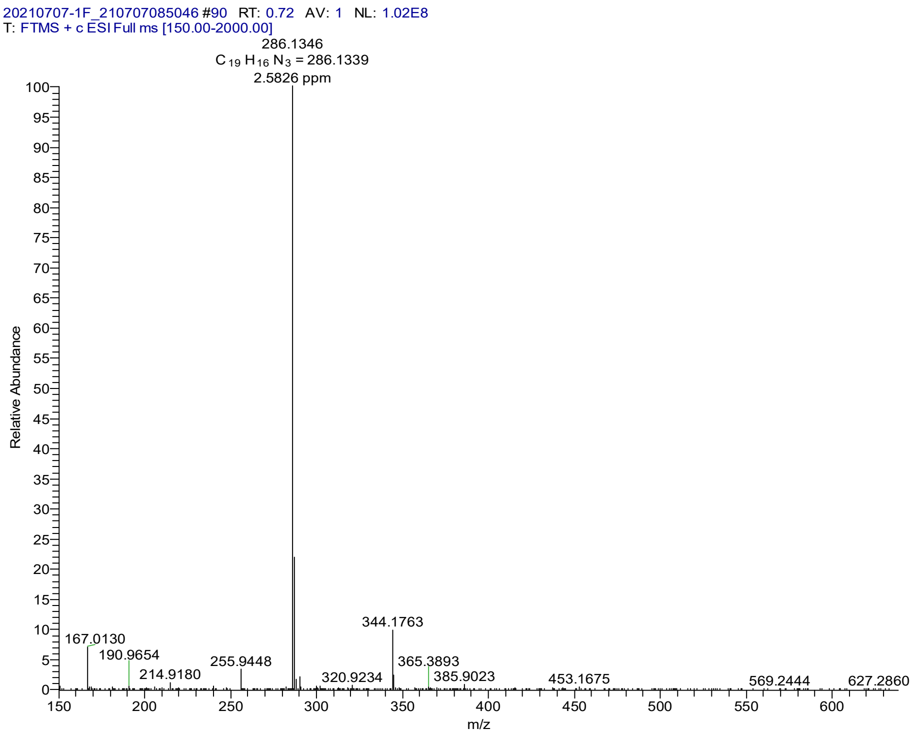

HRMS spectrum of **1f**

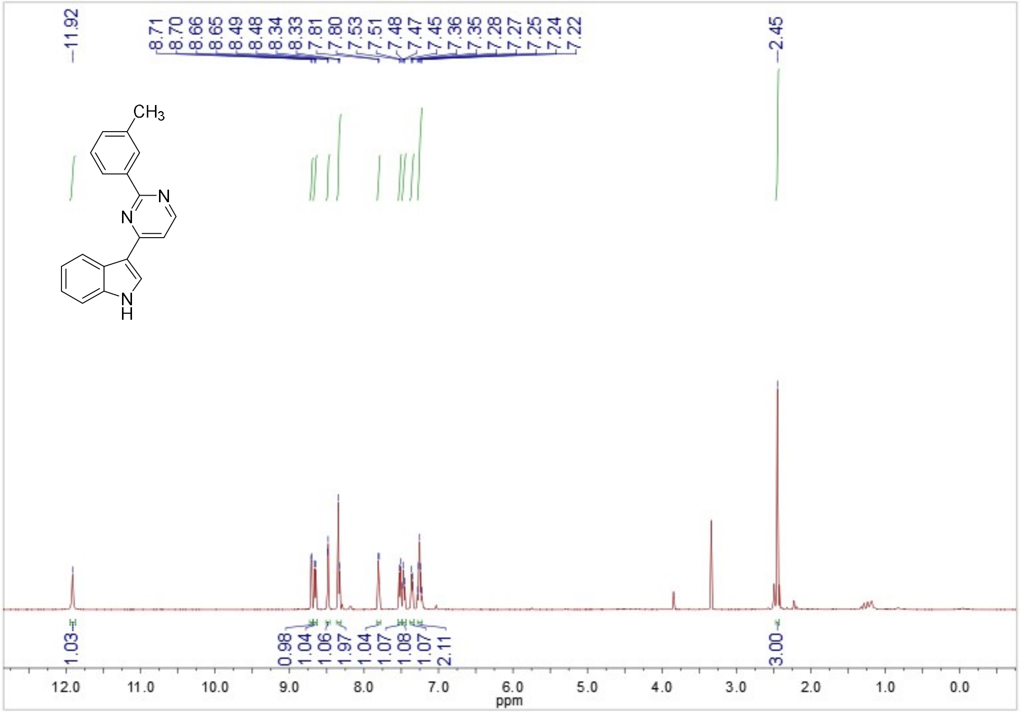

^{1}H NMR Spectrum of **1g**

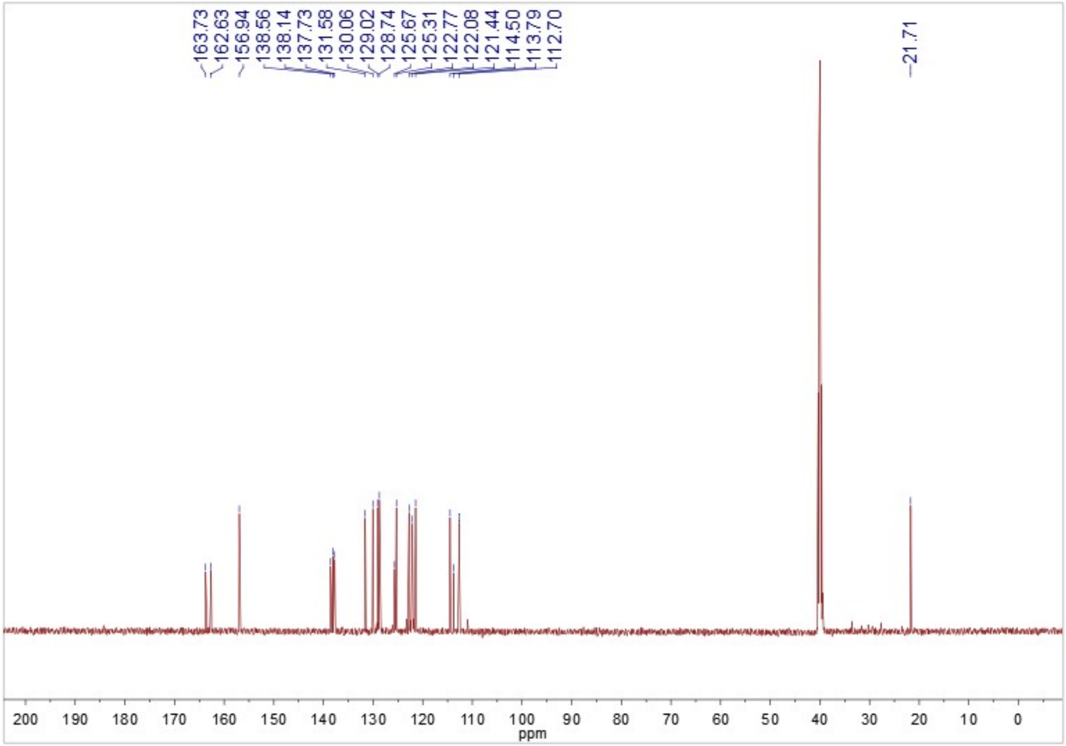

^{13}C NMR Spectrum of **1g**

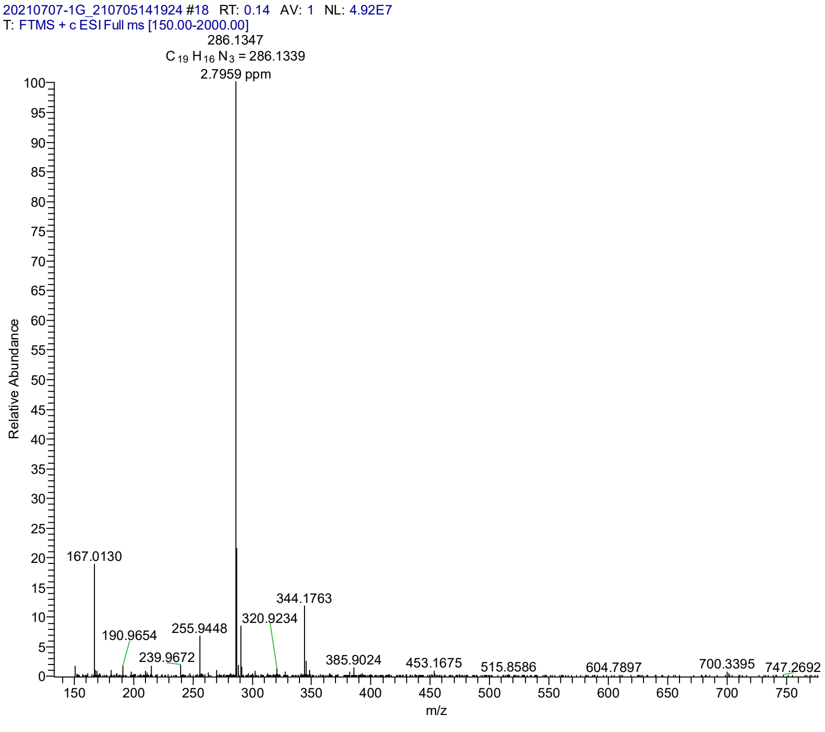

HRMS spectrum of **1g**

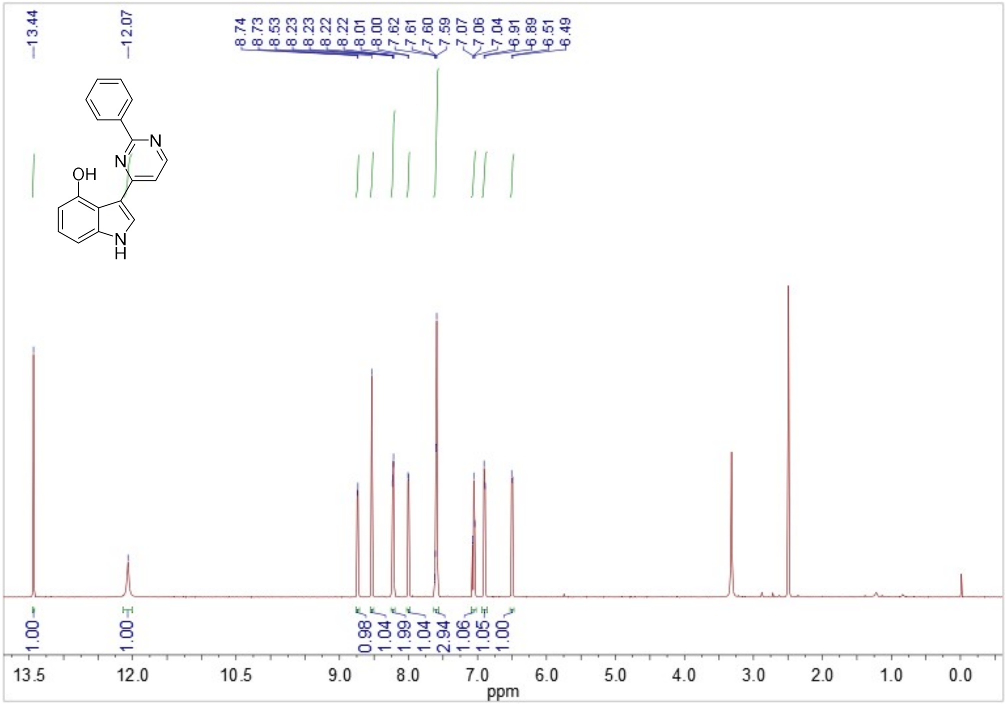

^{1}H NMR Spectrum of **2a**

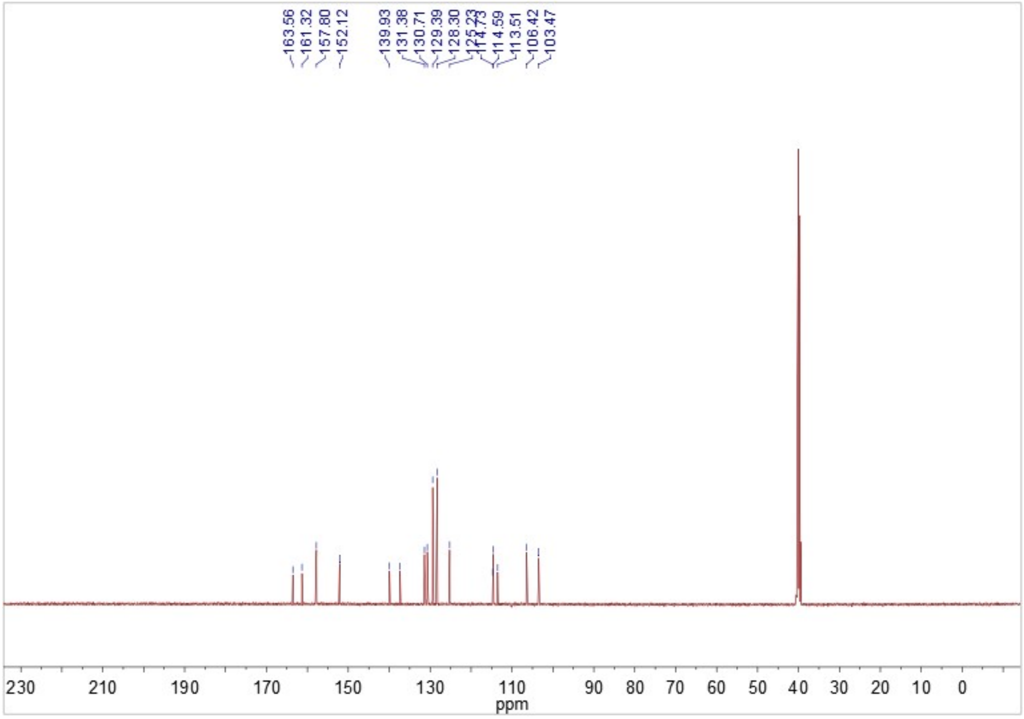

^{13}C NMR Spectrum of **2a**

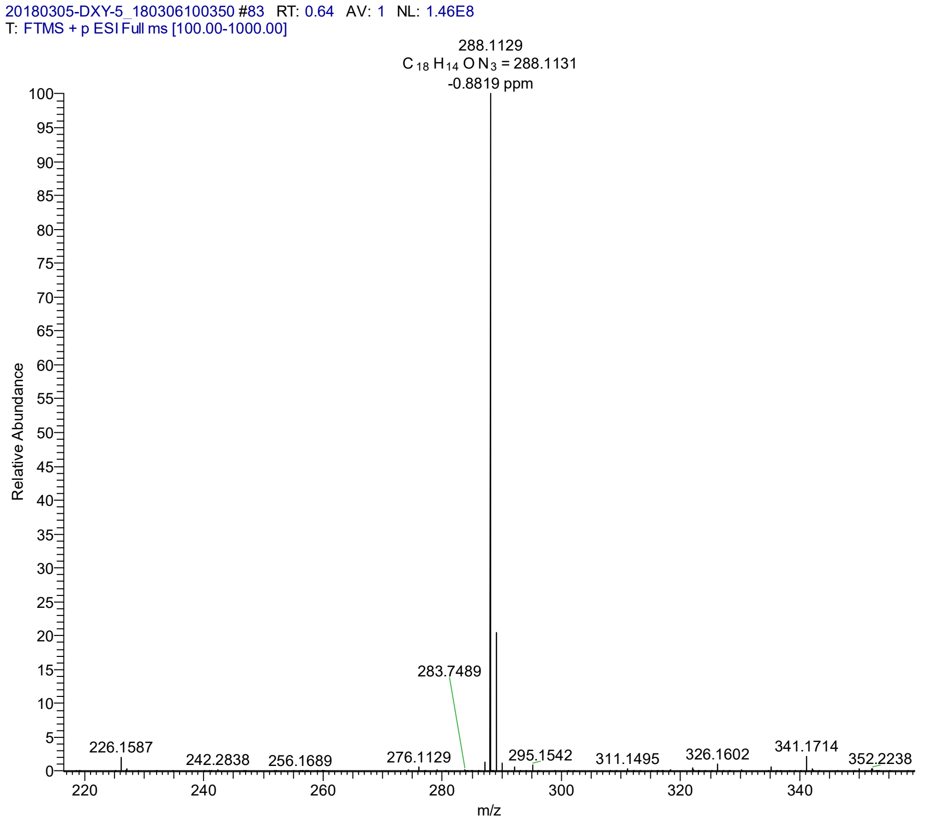

HRMS spectrum of **2a**

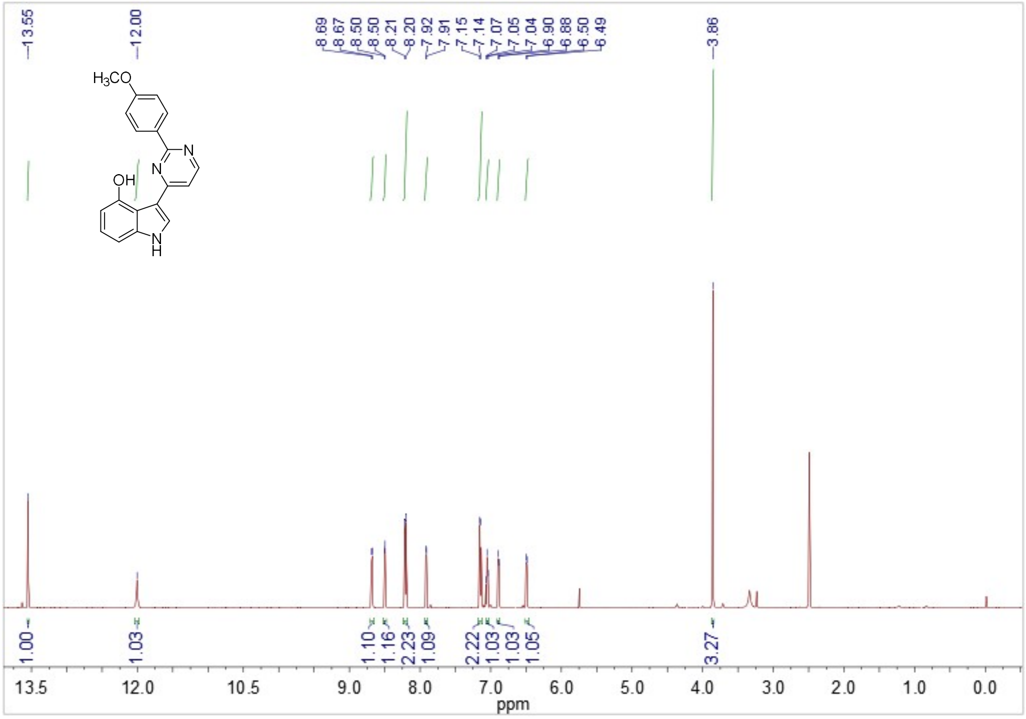

^{1}H NMR Spectrum of **2b**

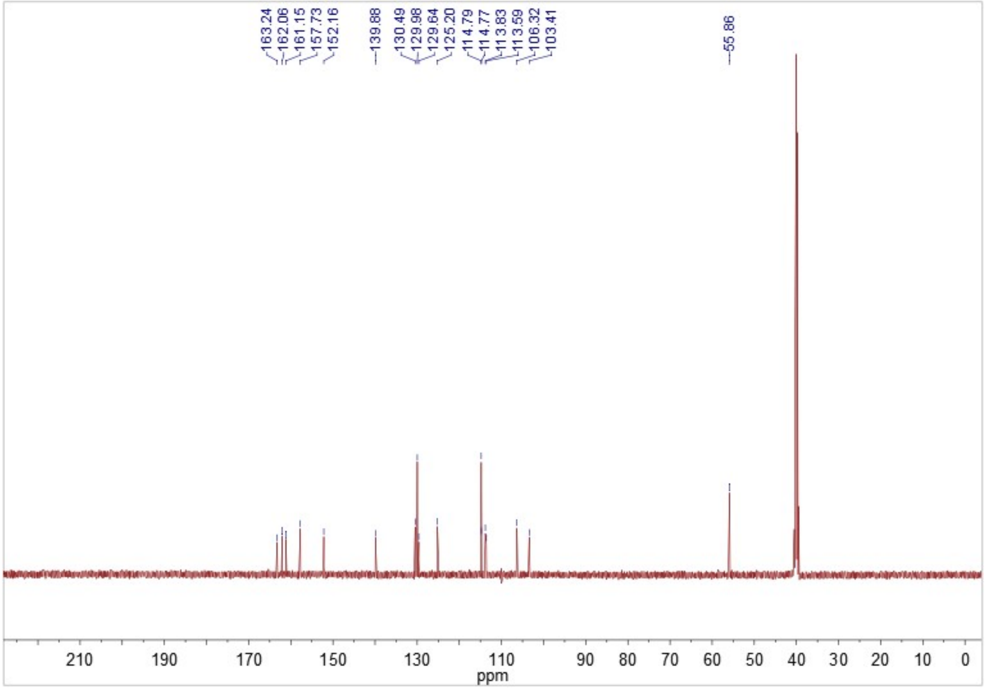

^{13}C NMR Spectrum of **2b**

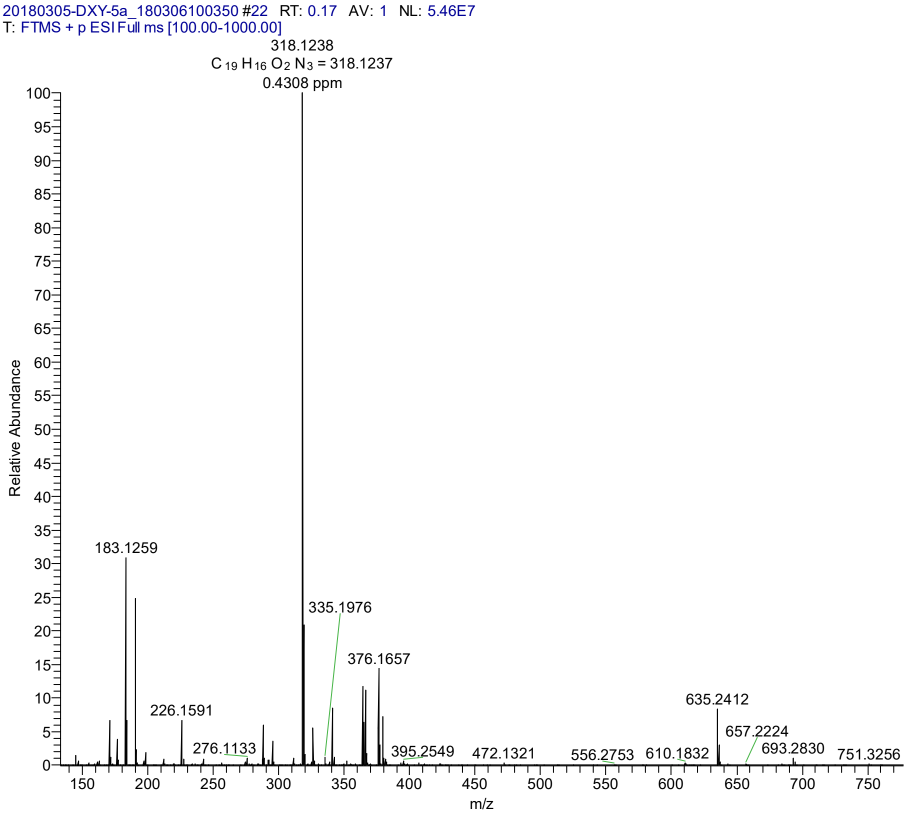

HRMS spectrum of **2b**

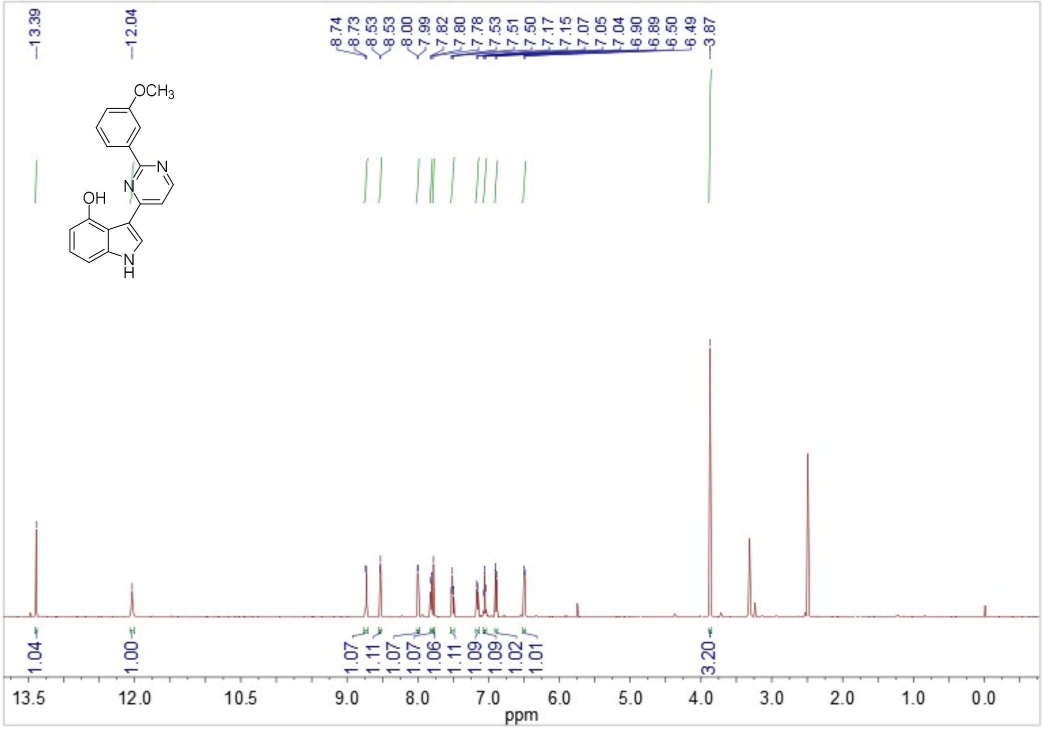

^{1}H NMR Spectrum of **2c**

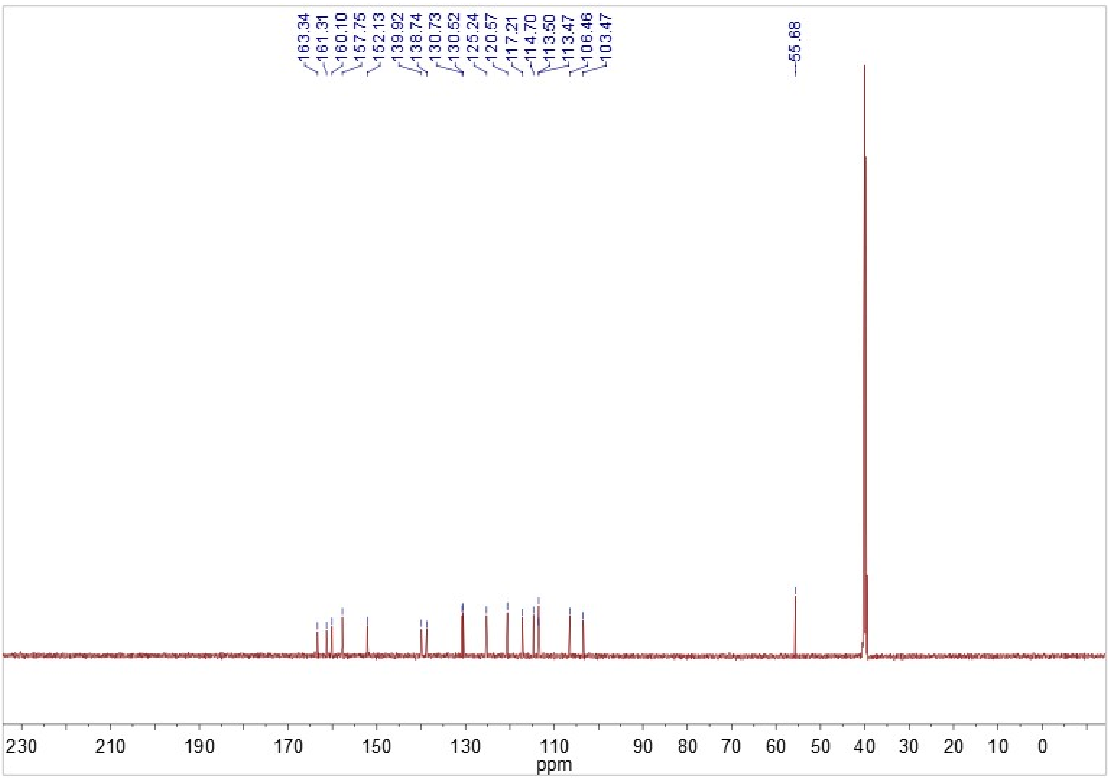

^{13}C NMR Spectrum of **2c**

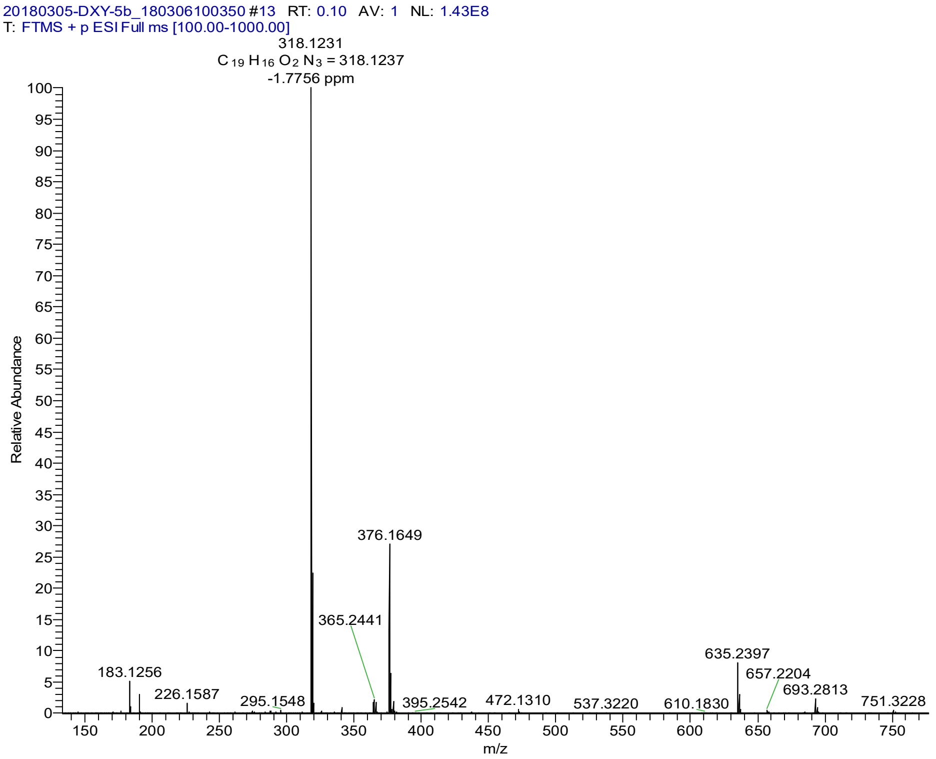

HRMS spectrum of **2c**

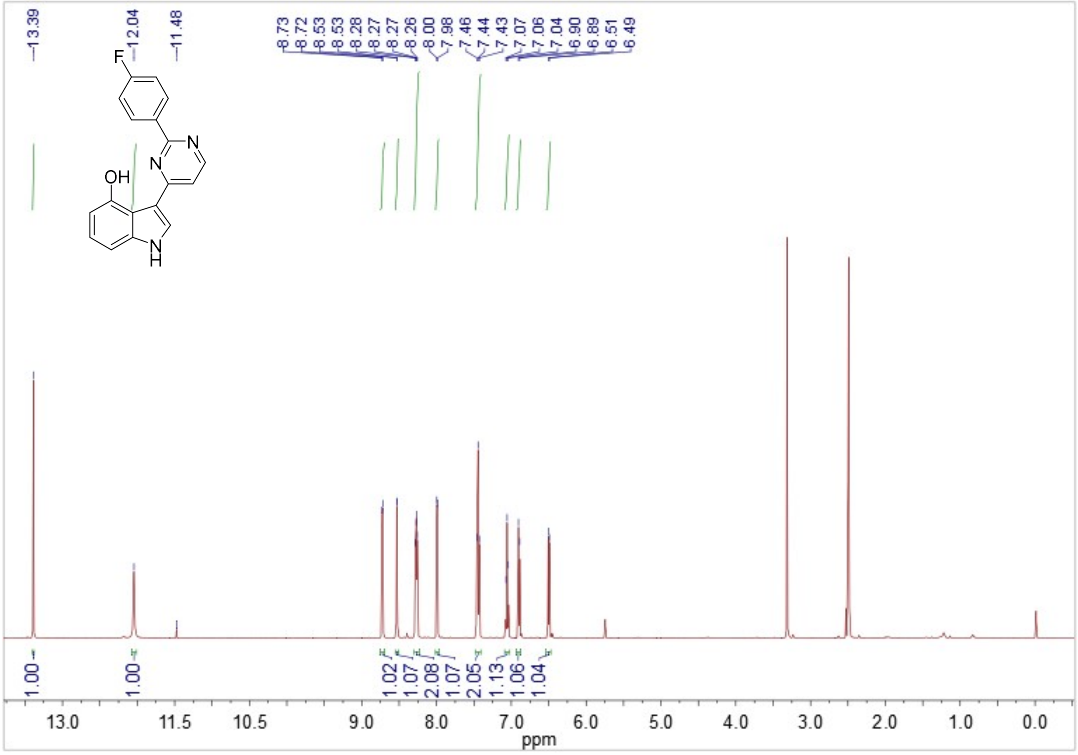

^{1}H NMR Spectrum of **2d**

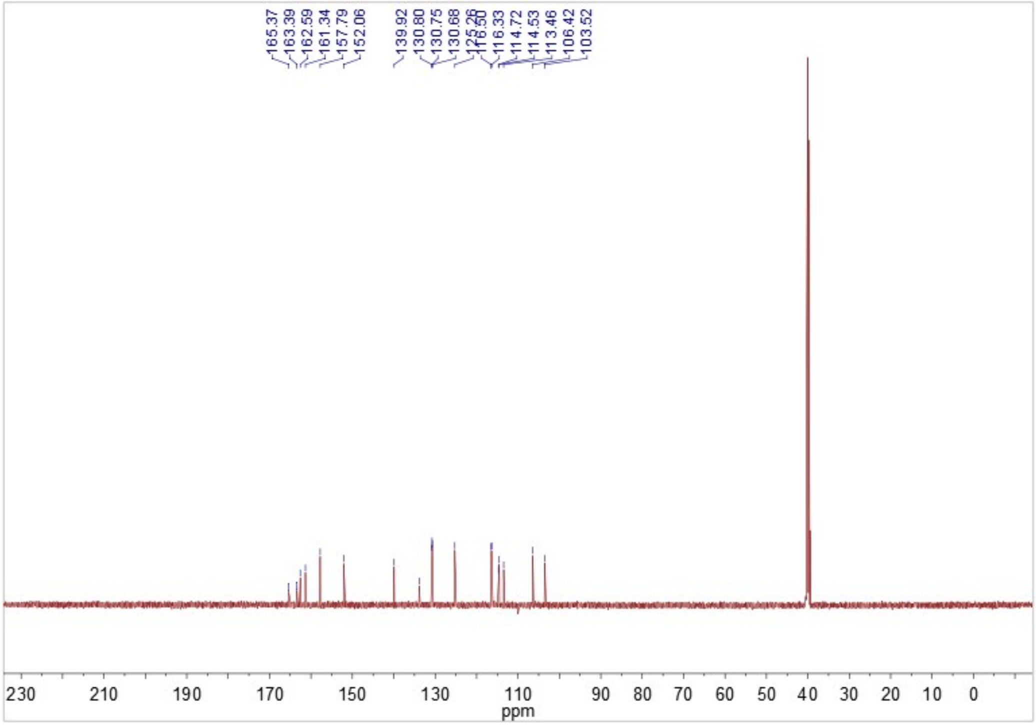

^{13}C NMR Spectrum of **2d**

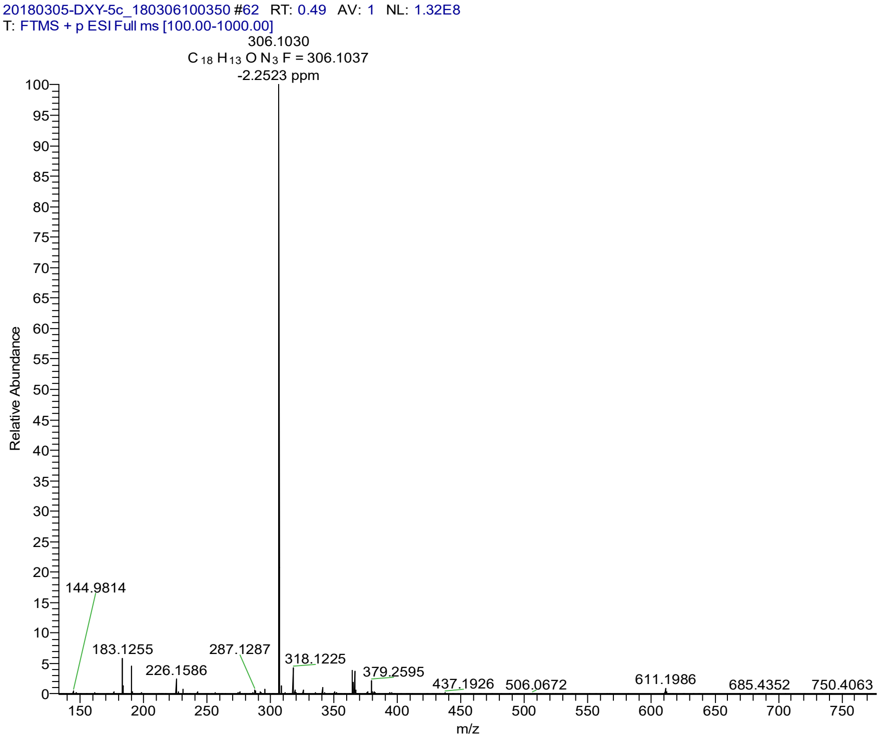

HRMS spectrum of **2d**

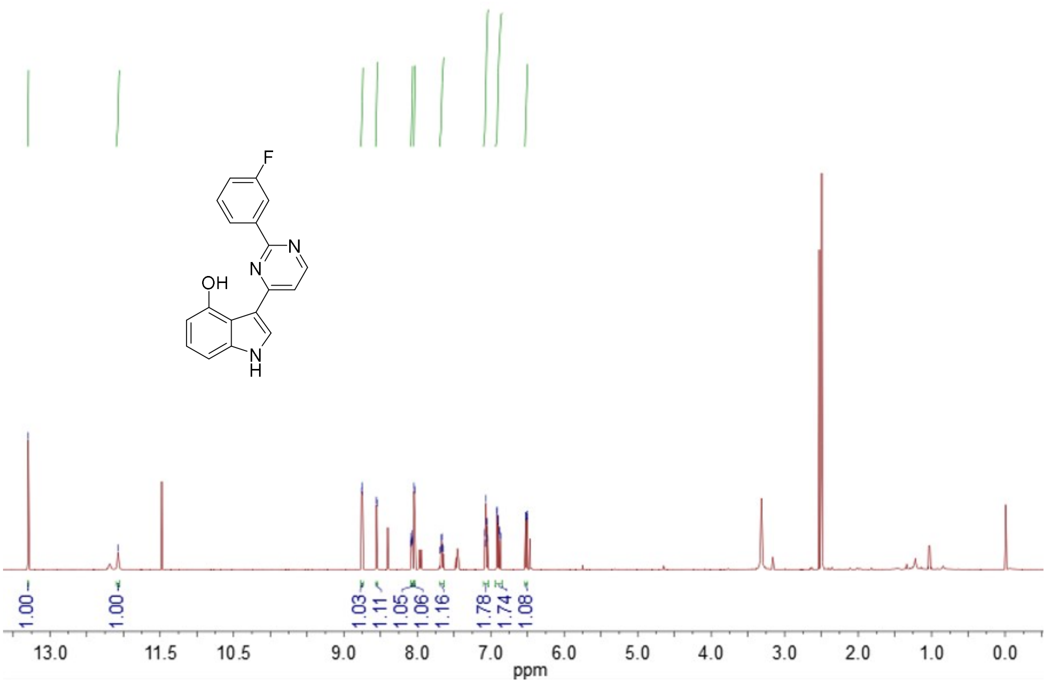

^{1}H NMR Spectrum of **2e**

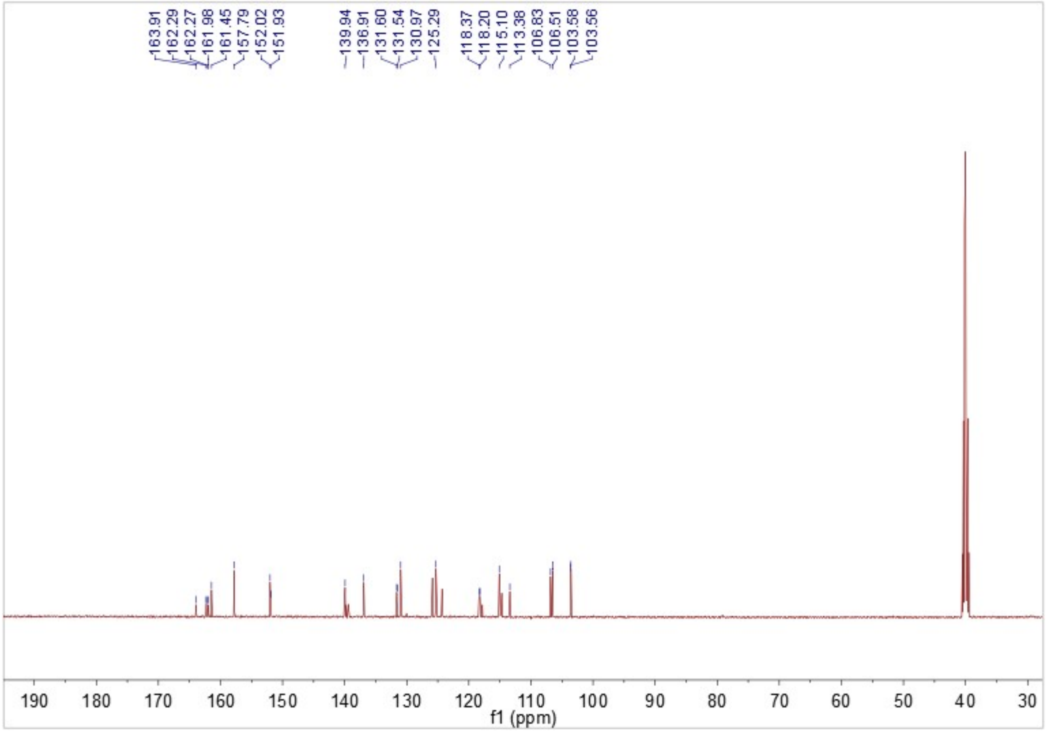

^{13}C NMR Spectrum of **2e**

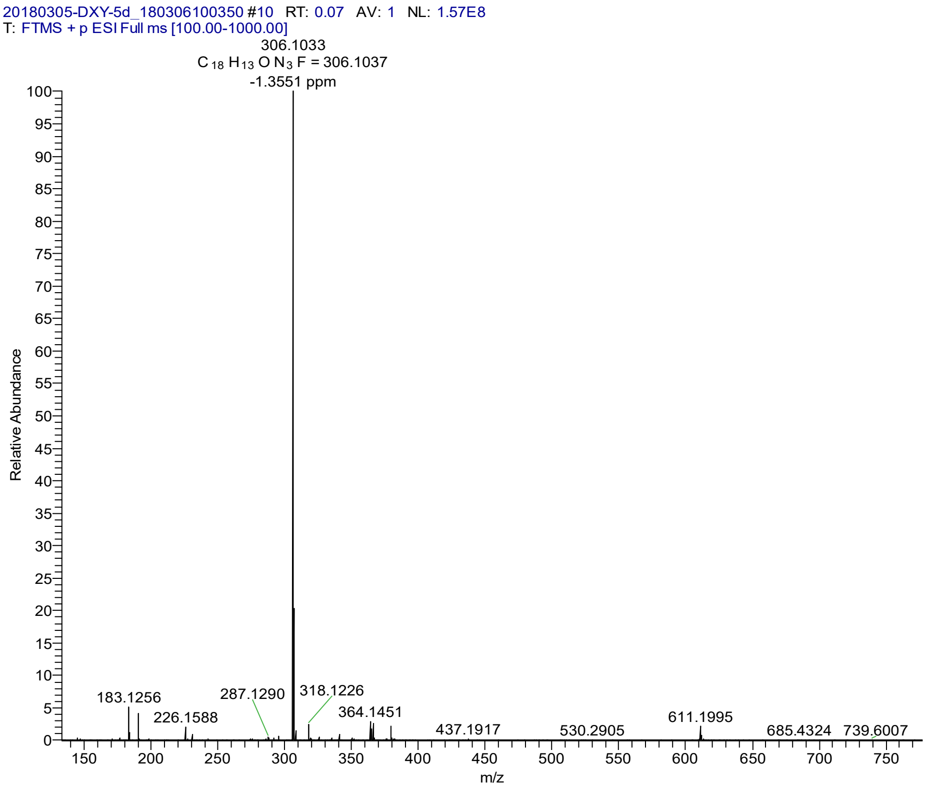

HRMS spectrum of **2e**

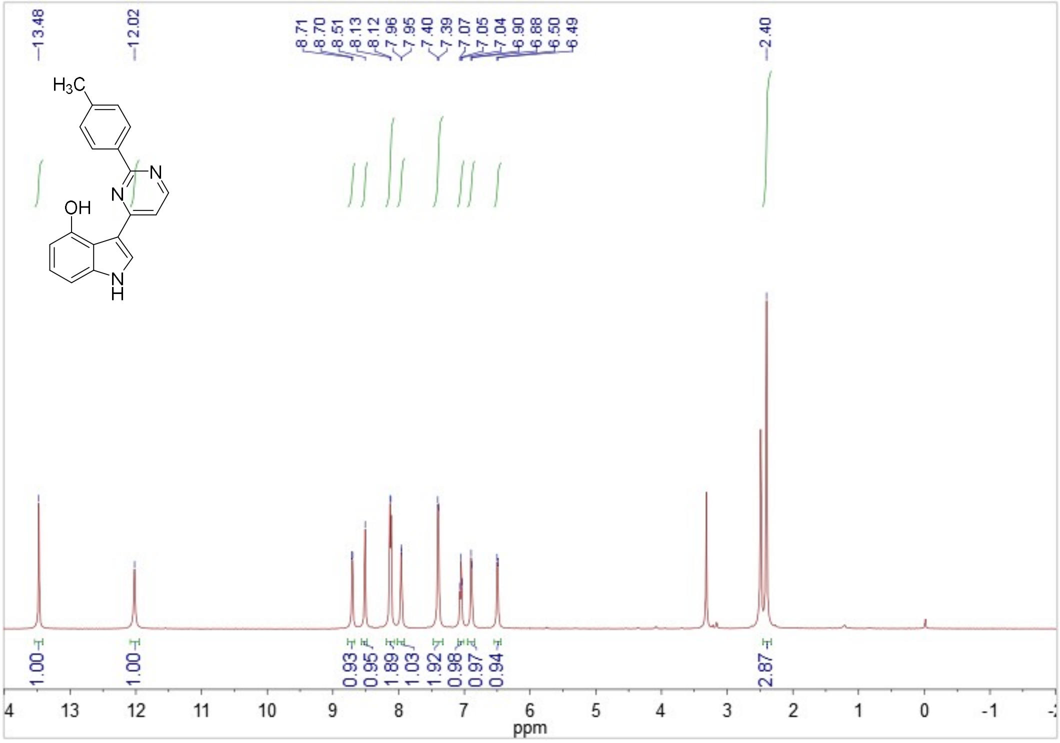

^{1}H NMR Spectrum of **2f**

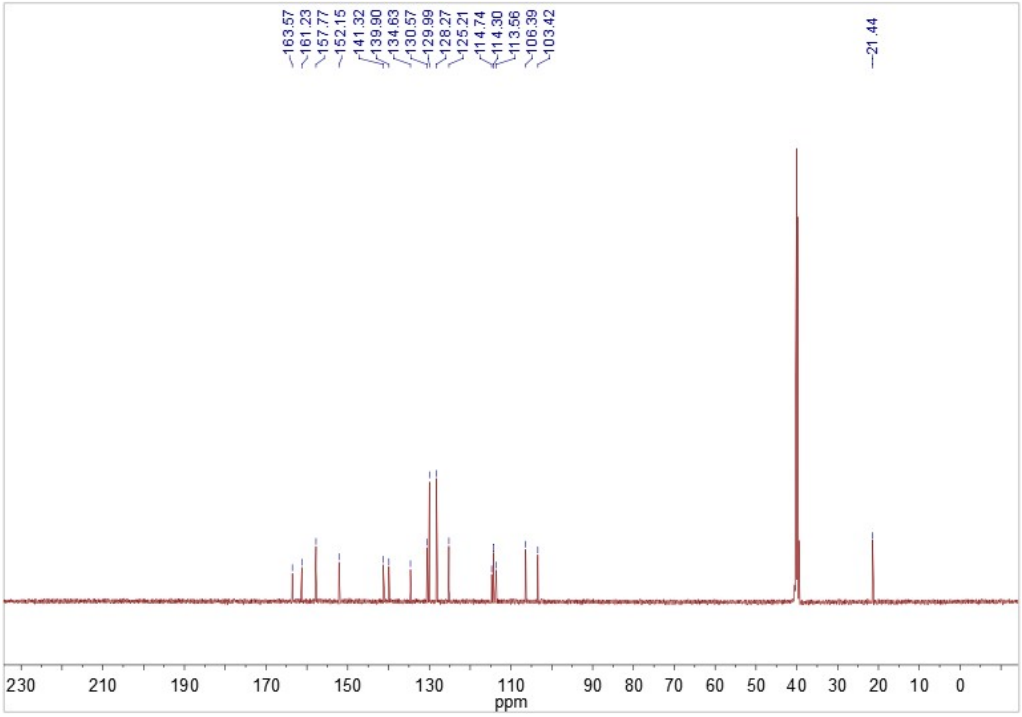

^{13}C NMR Spectrum of **2f**

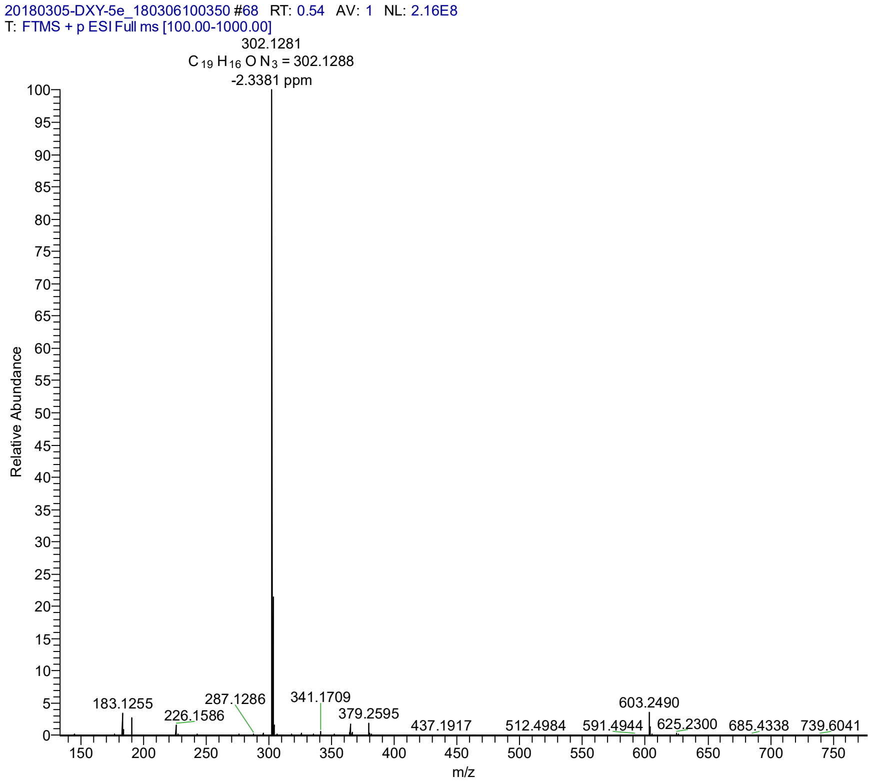

HRMS spectrum of **2f**

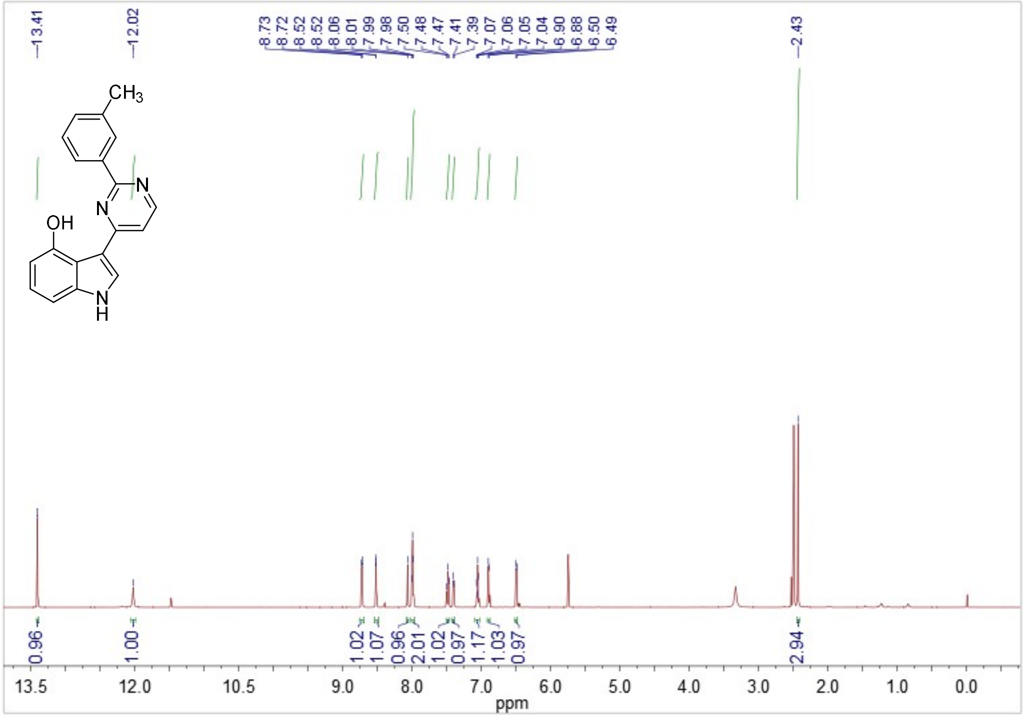

^{1}H NMR Spectrum of **2g**

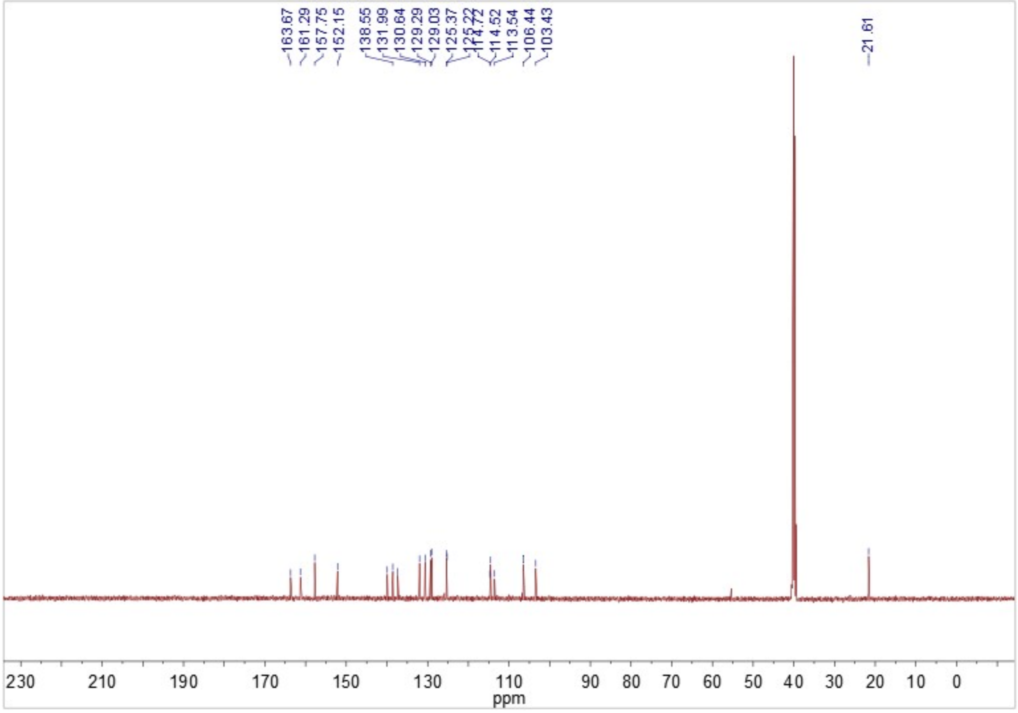

^{13}C NMR Spectrum of **2g**

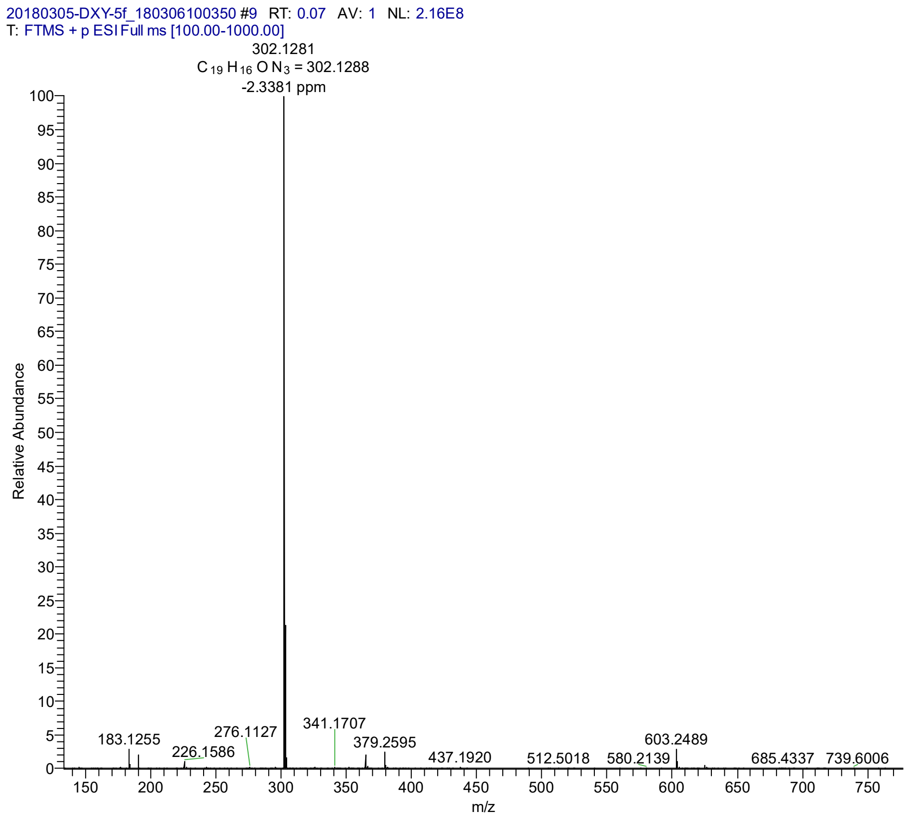

HRMS spectrum of **2g**

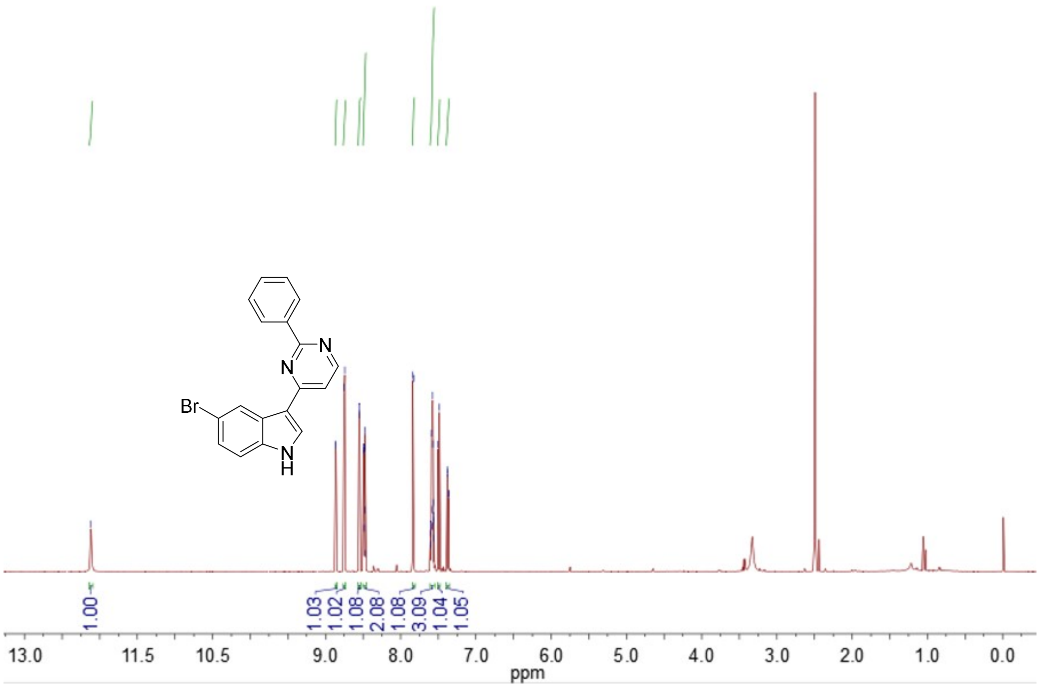

^{1}H NMR Spectrum of **3a**

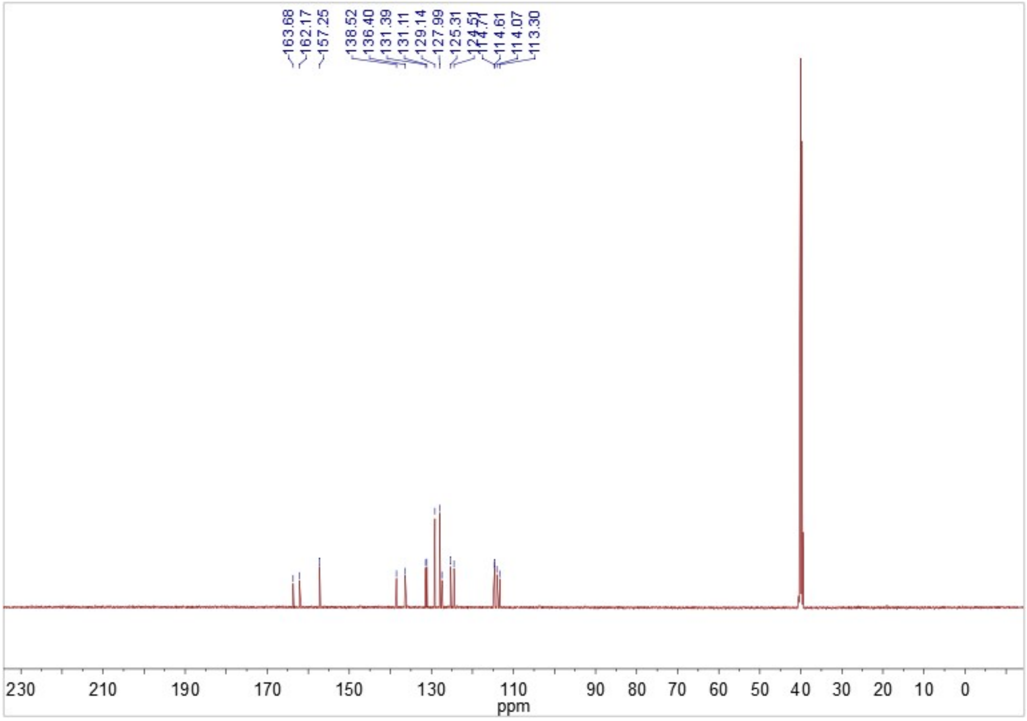

^{13}C NMR Spectrum of **3a**

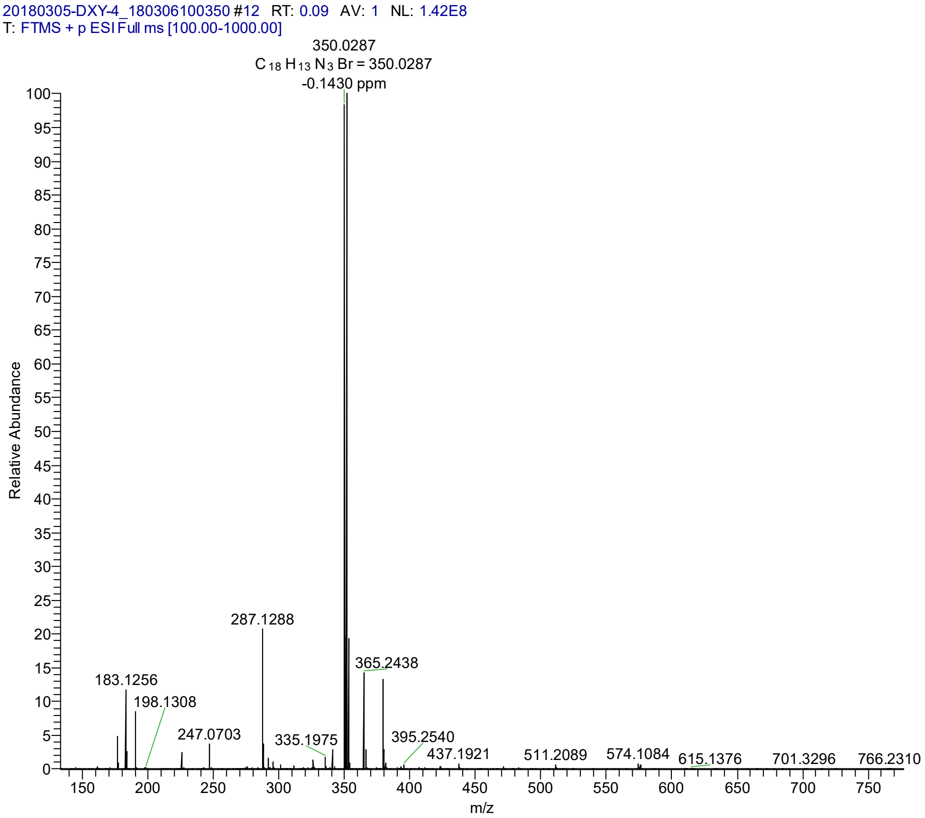

HRMS spectrum of **3a**

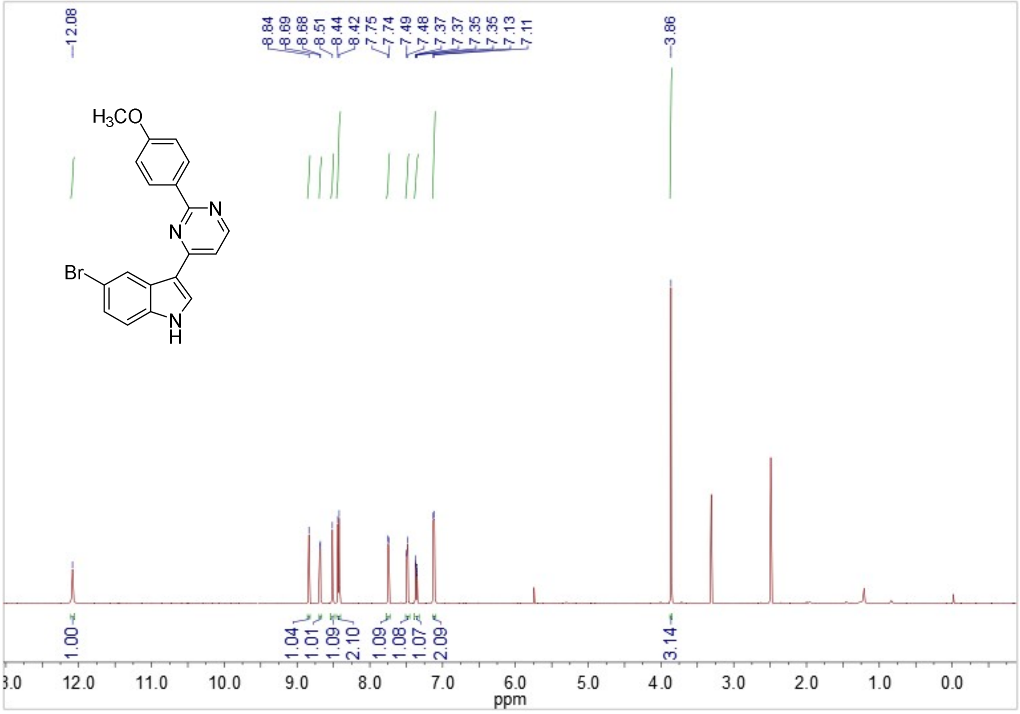

^{1}H NMR Spectrum of **3b**

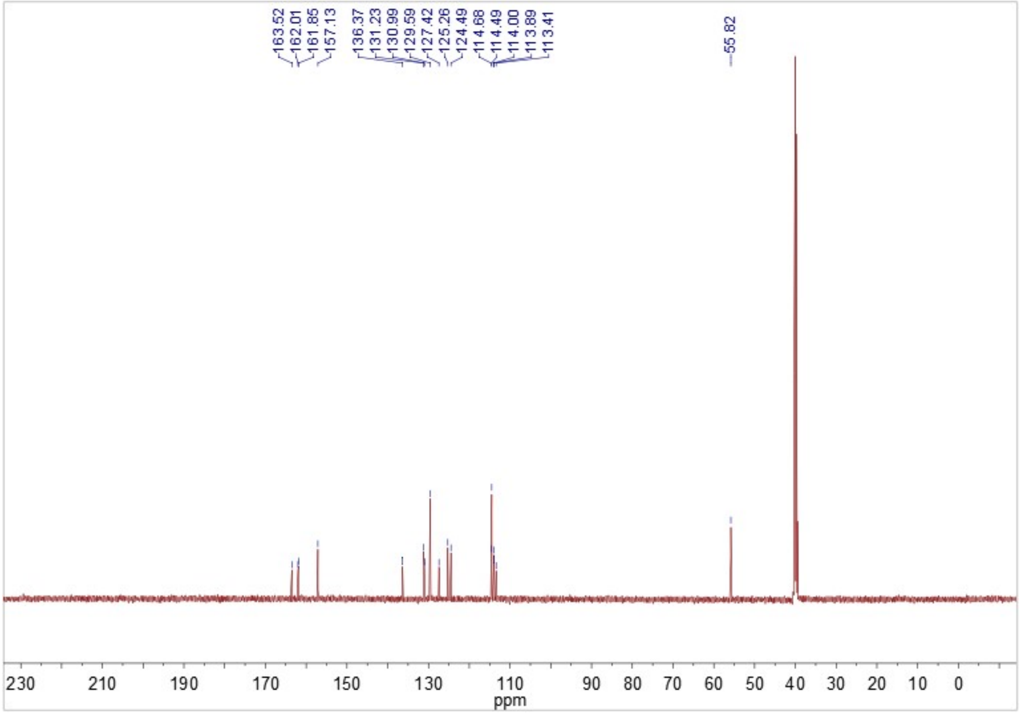

^{13}C NMR Spectrum of **3b**

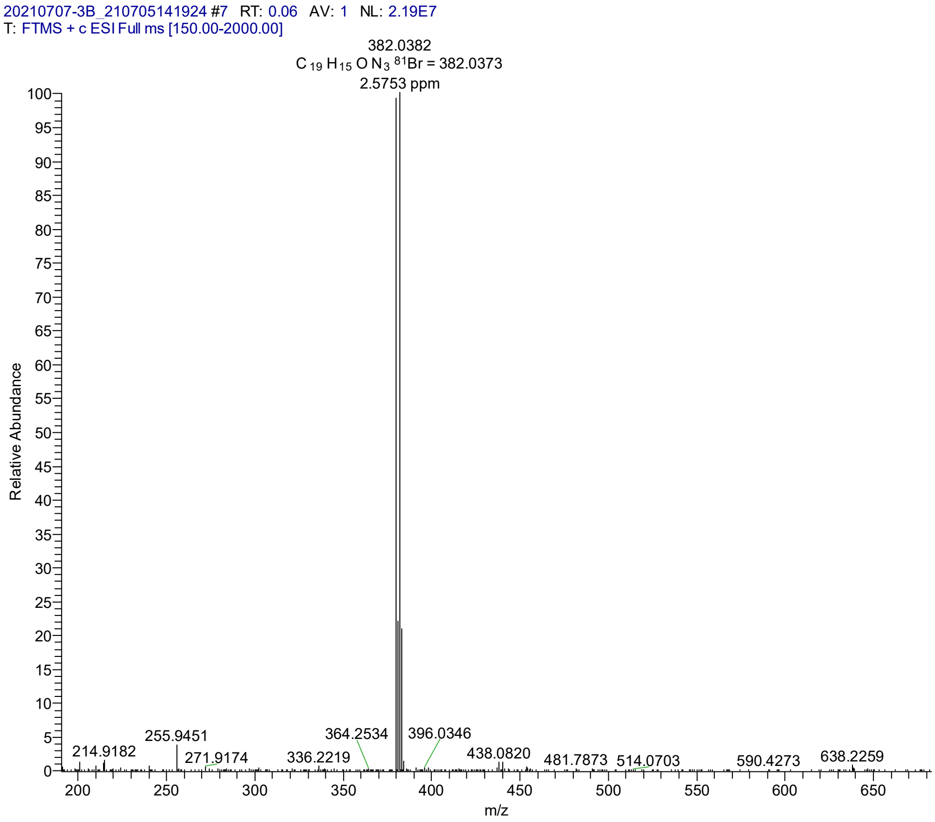

HRMS spectrum of **3b**

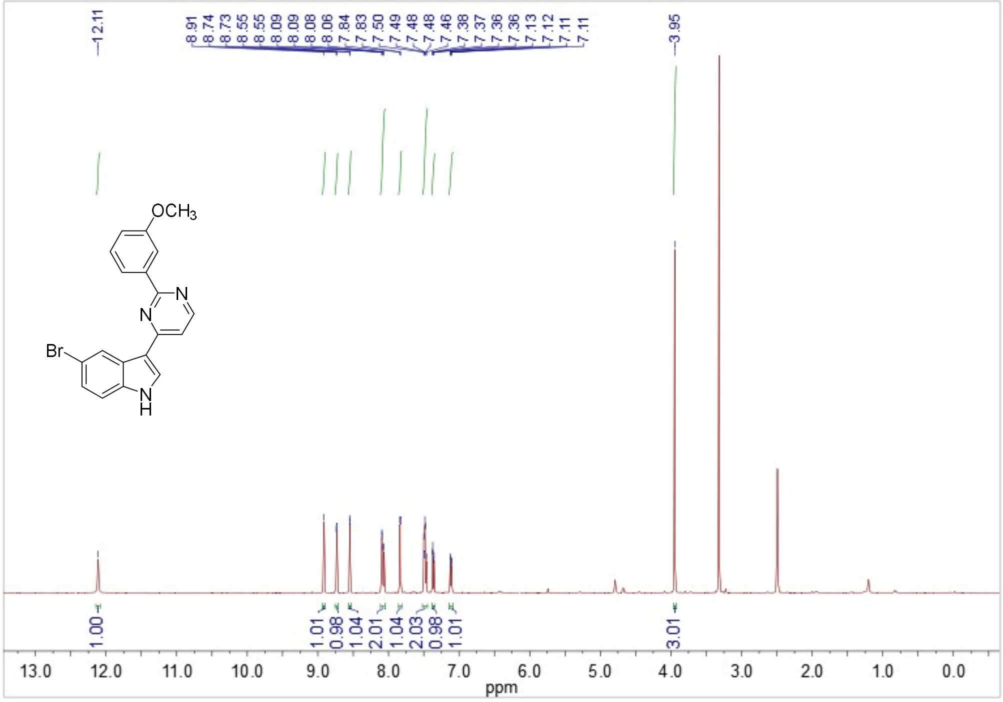

^{1}H NMR Spectrum of **3c**

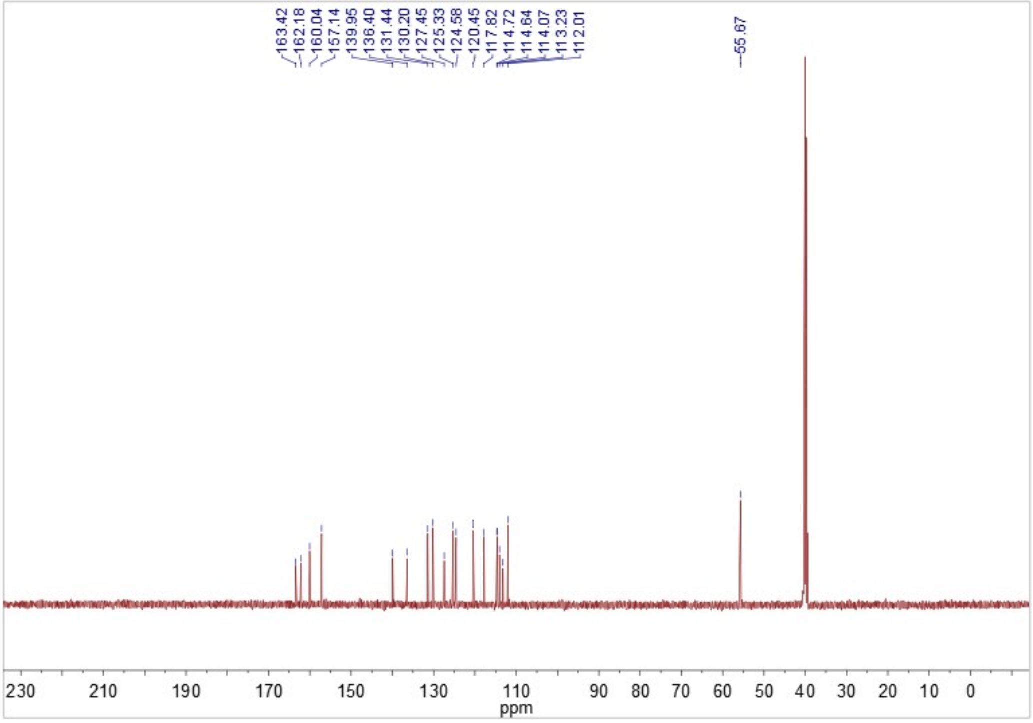

^{13}C NMR Spectrum of **3c**

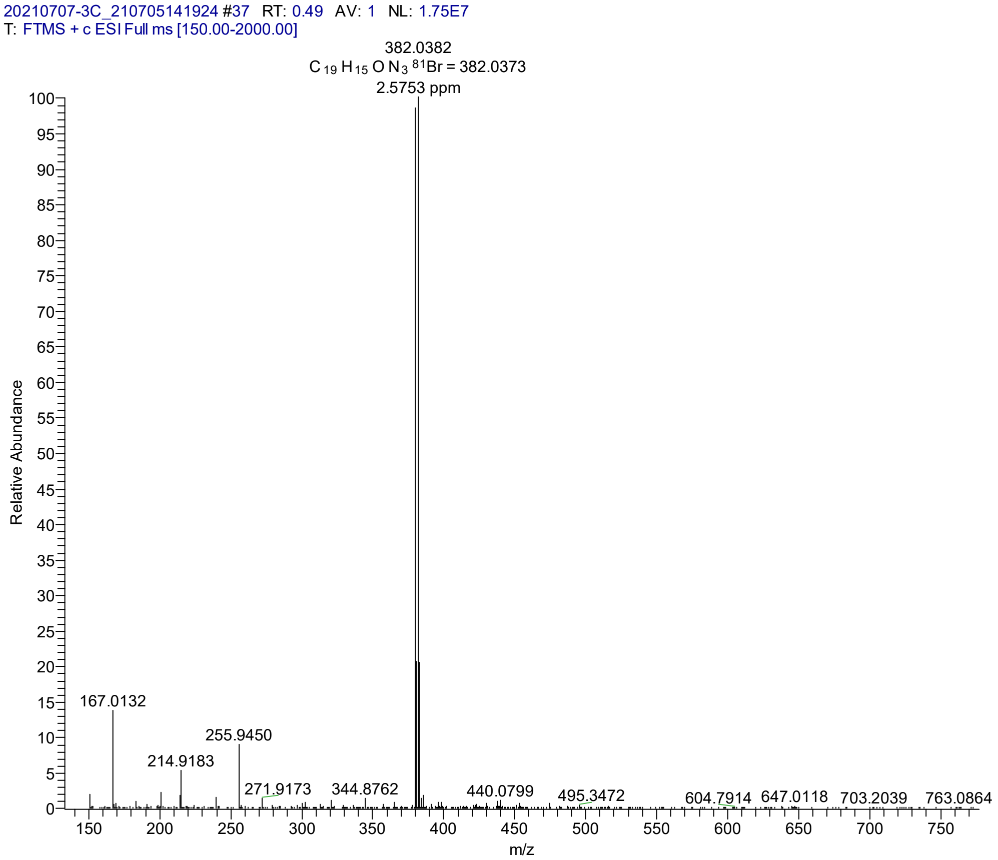

HRMS spectrum of **3c**

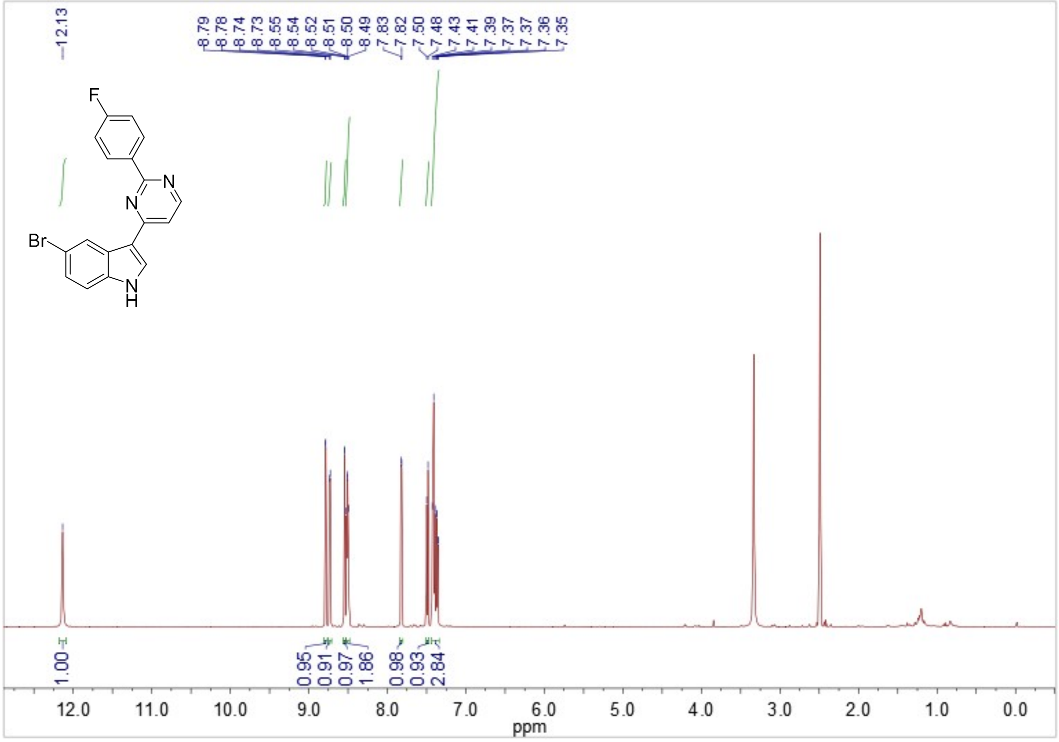

^{1}H NMR Spectrum of **3d**

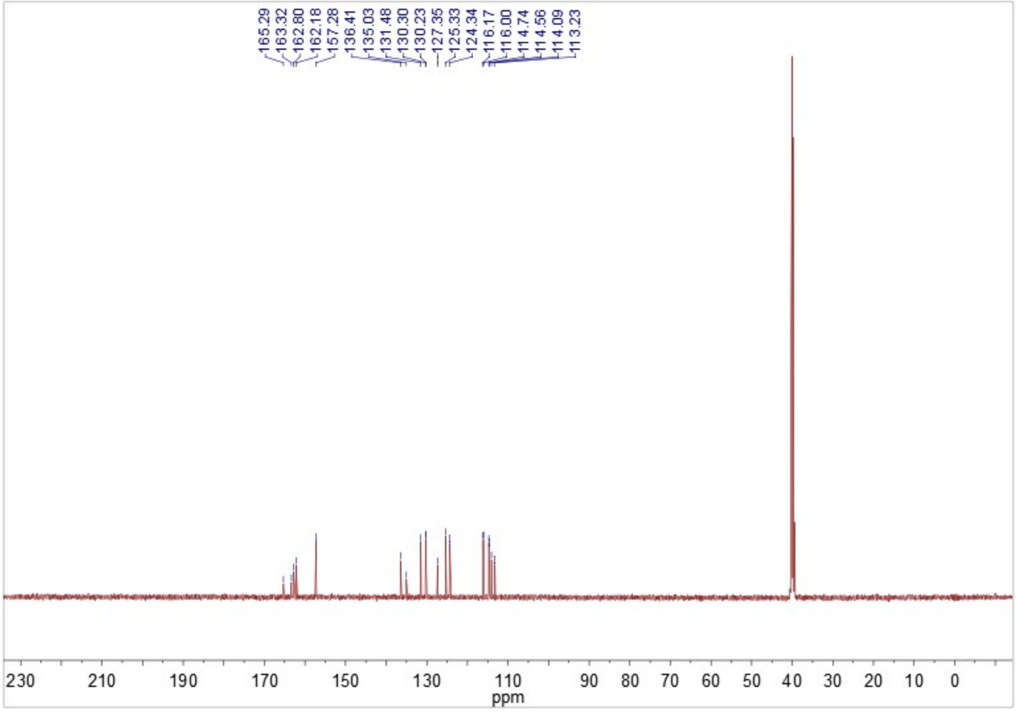

^{13}C NMR Spectrum of **3d**

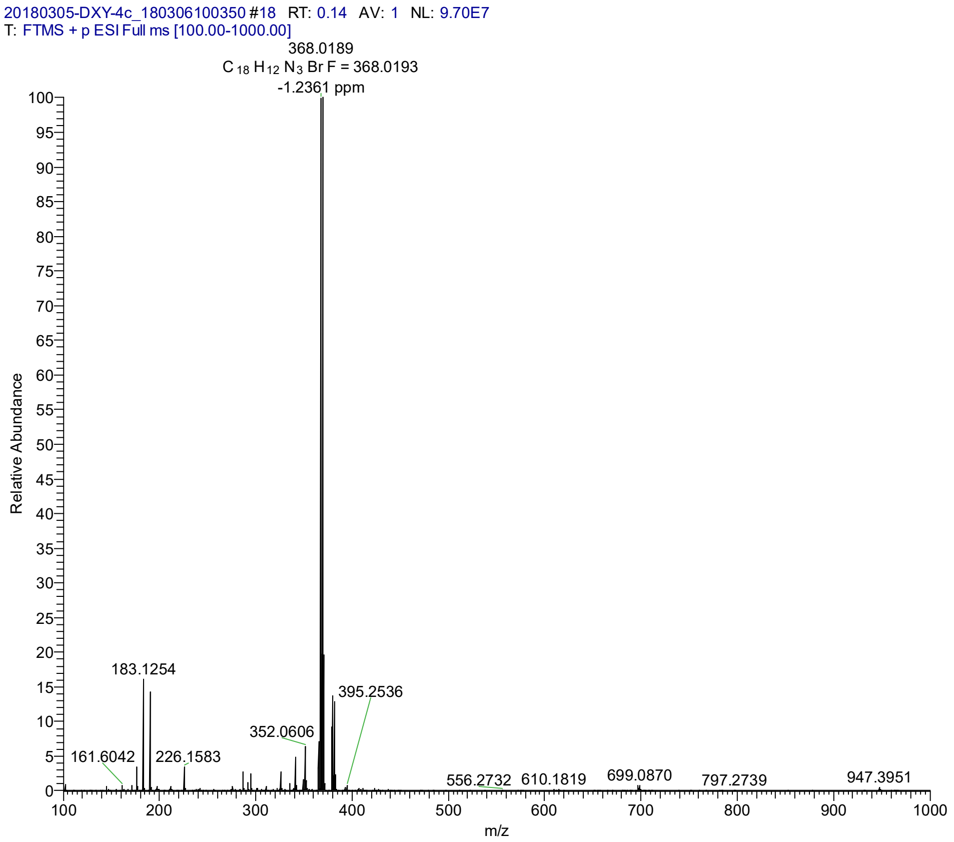

HRMS spectrum of **3d**

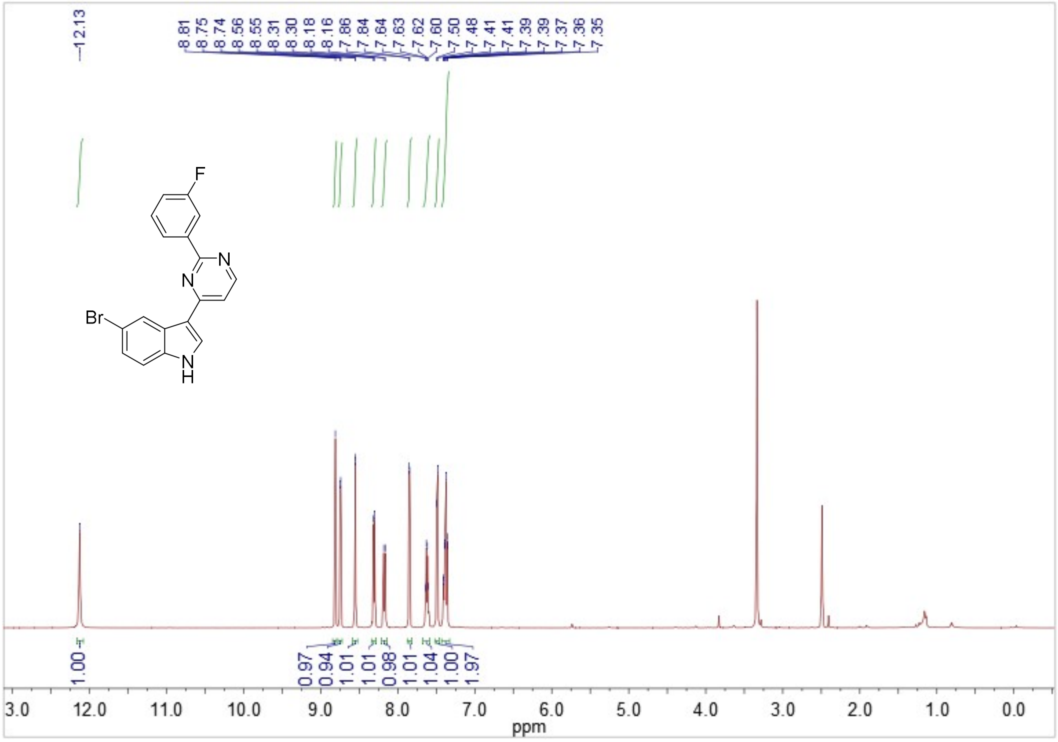

^{1}H NMR Spectrum of **3e**

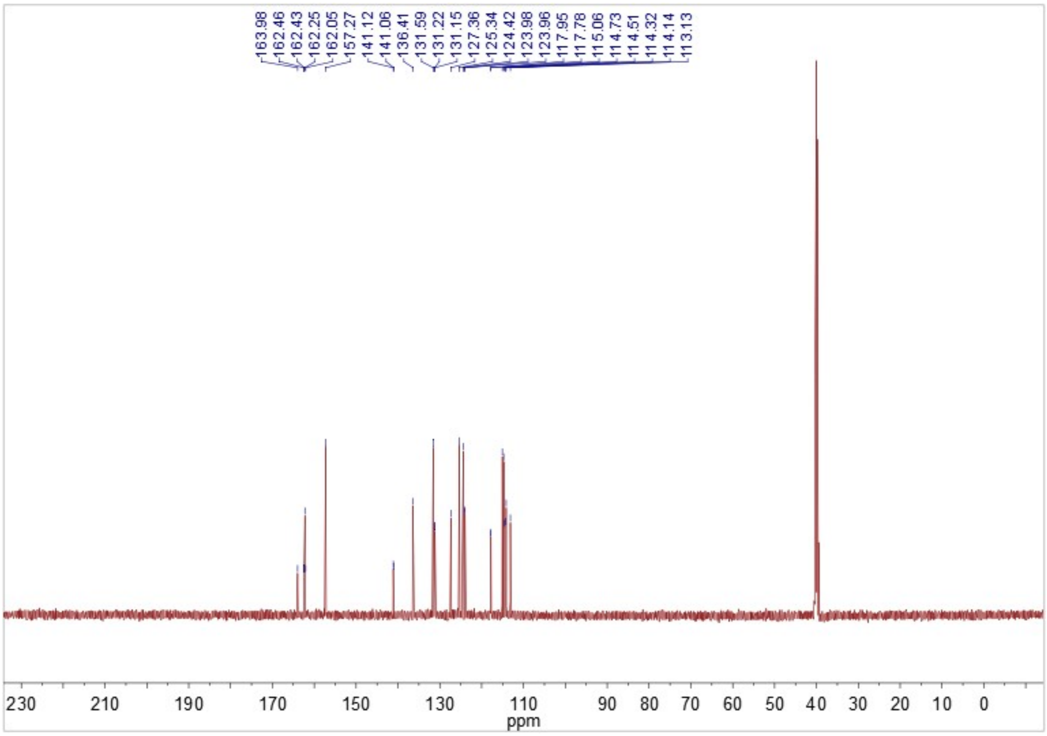

^{13}C NMR Spectrum of **3e**

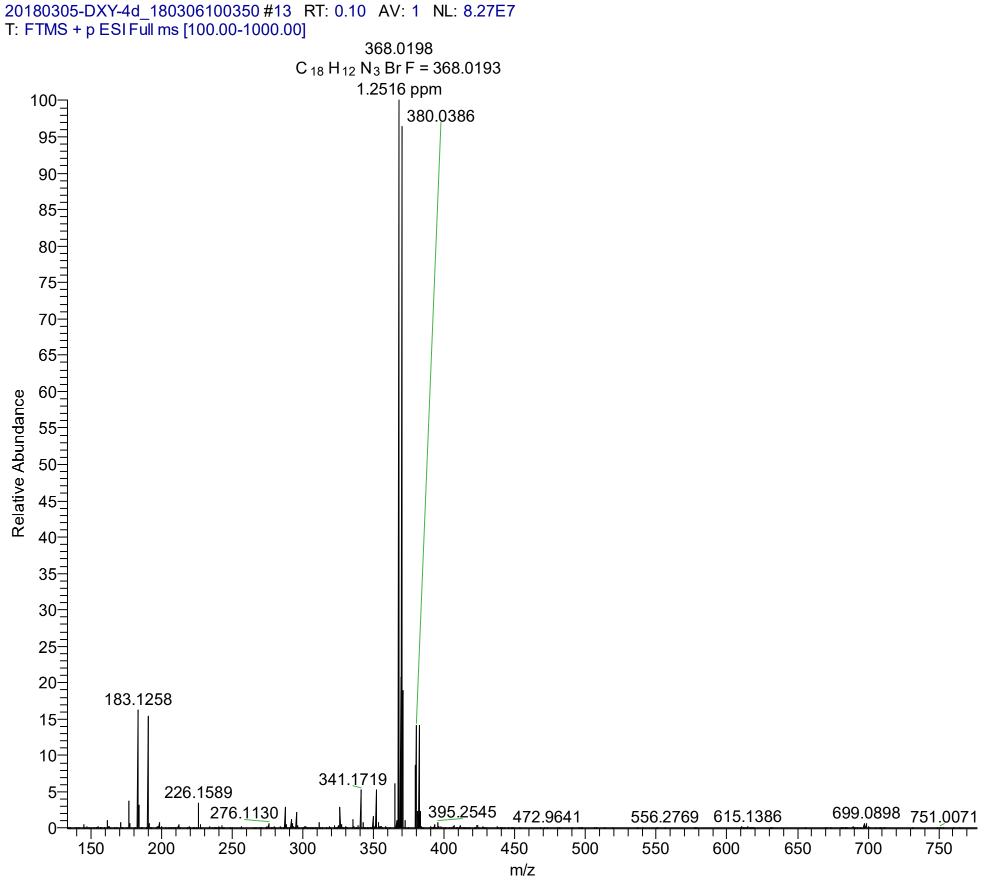

HRMS spectrum of **3e**

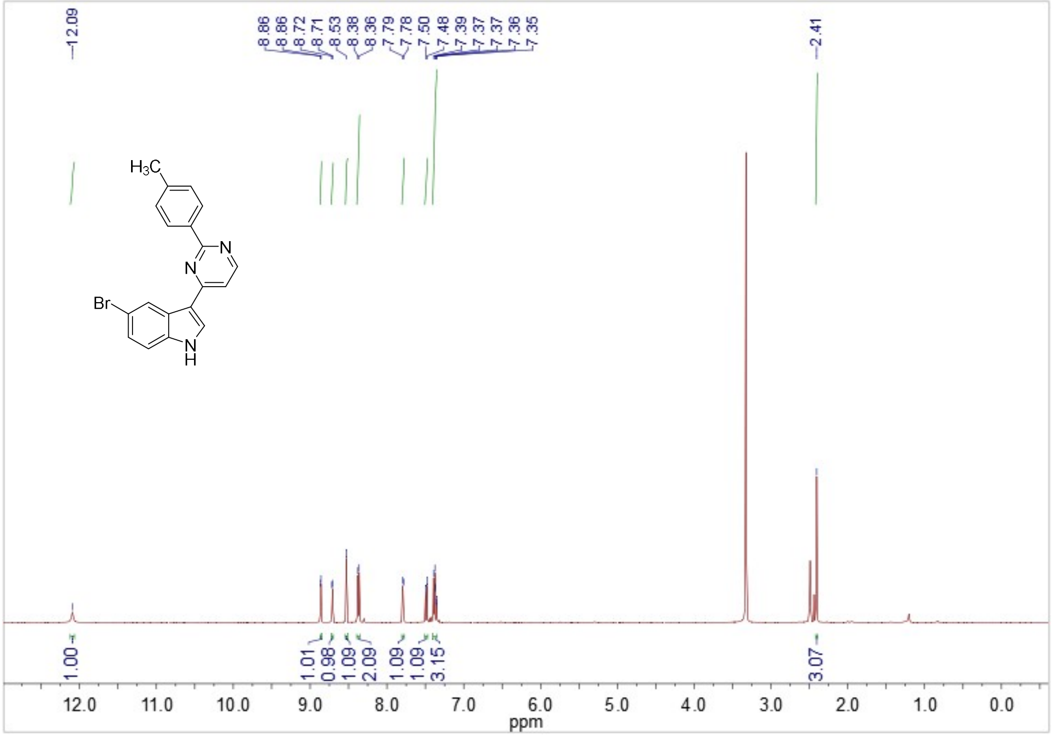

^{1}H NMR Spectrum of **3f**

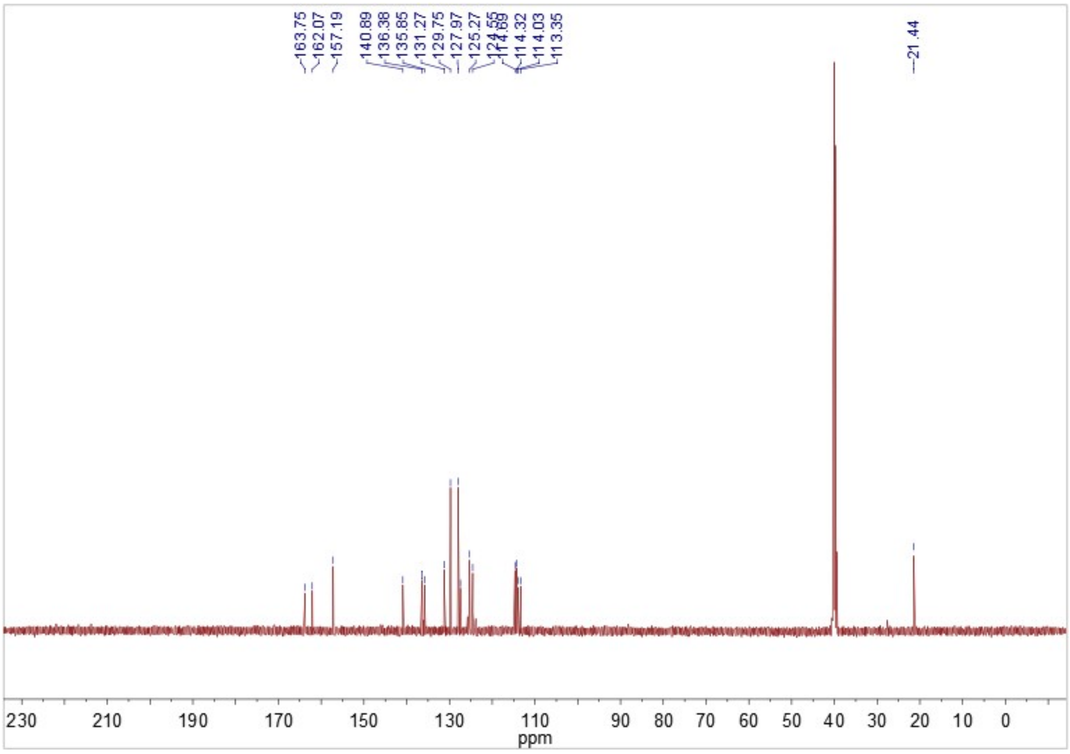

^{13}C NMR Spectrum of **3f**

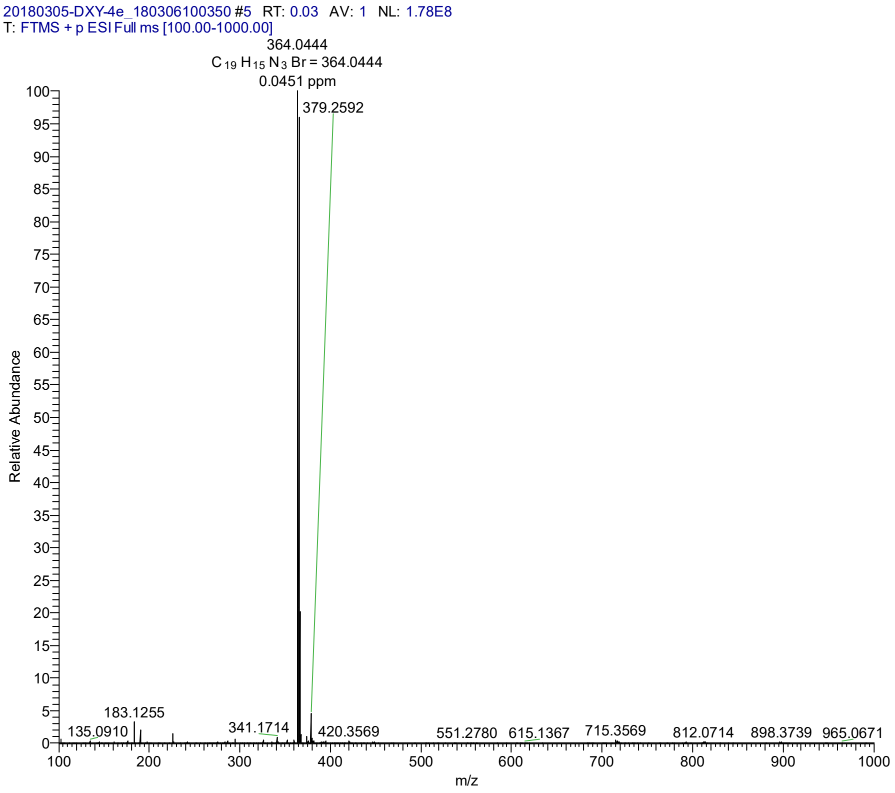

HRMS spectrum of **3f**

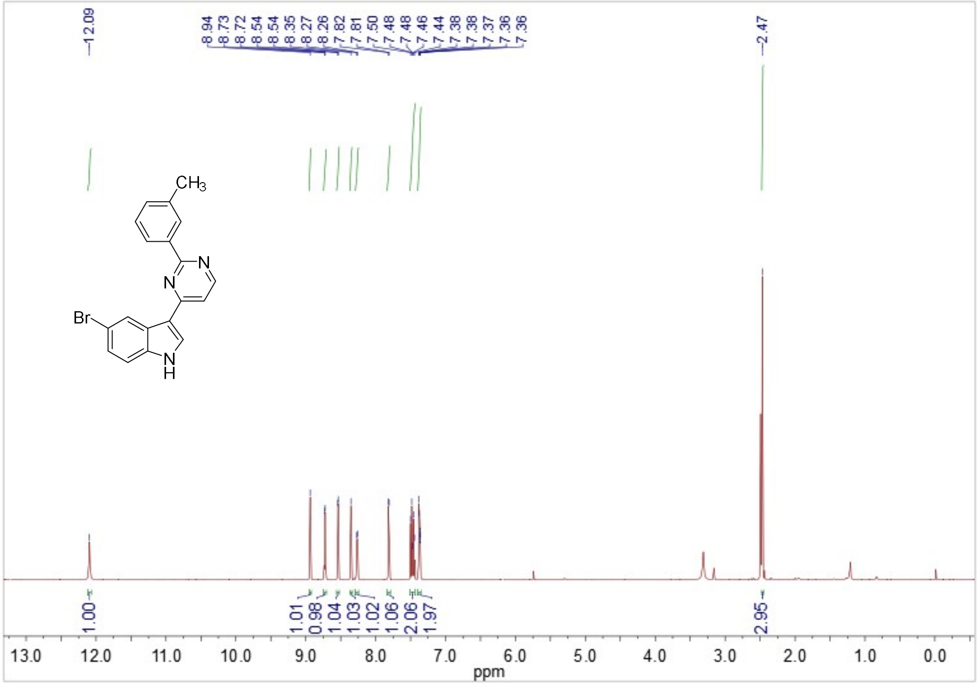

^{1}H NMR Spectrum of **3g**

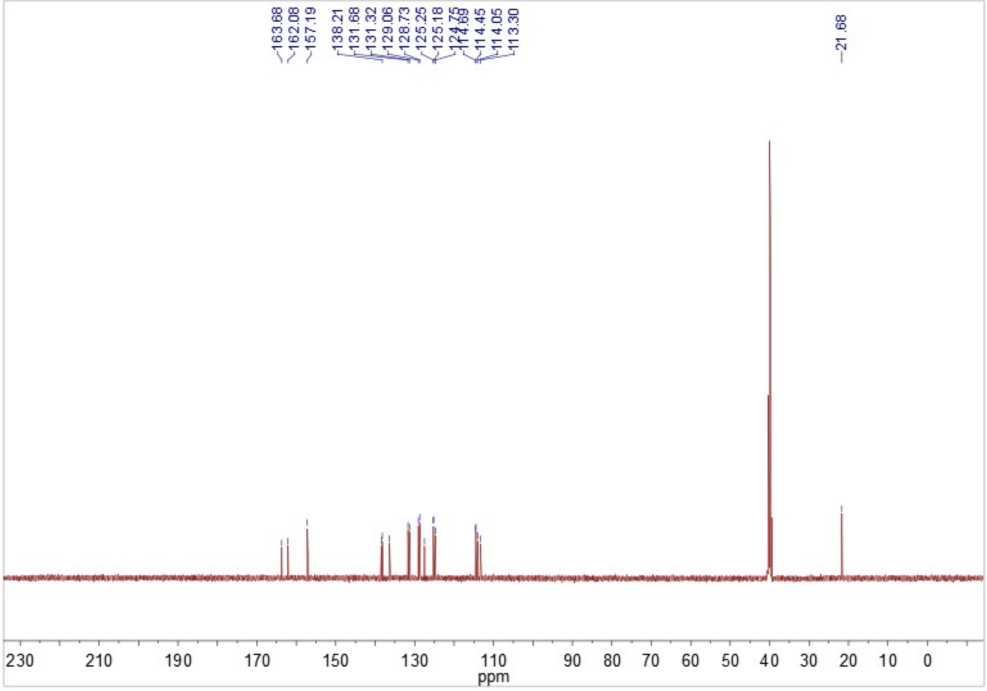

^{13}C NMR Spectrum of **3g**

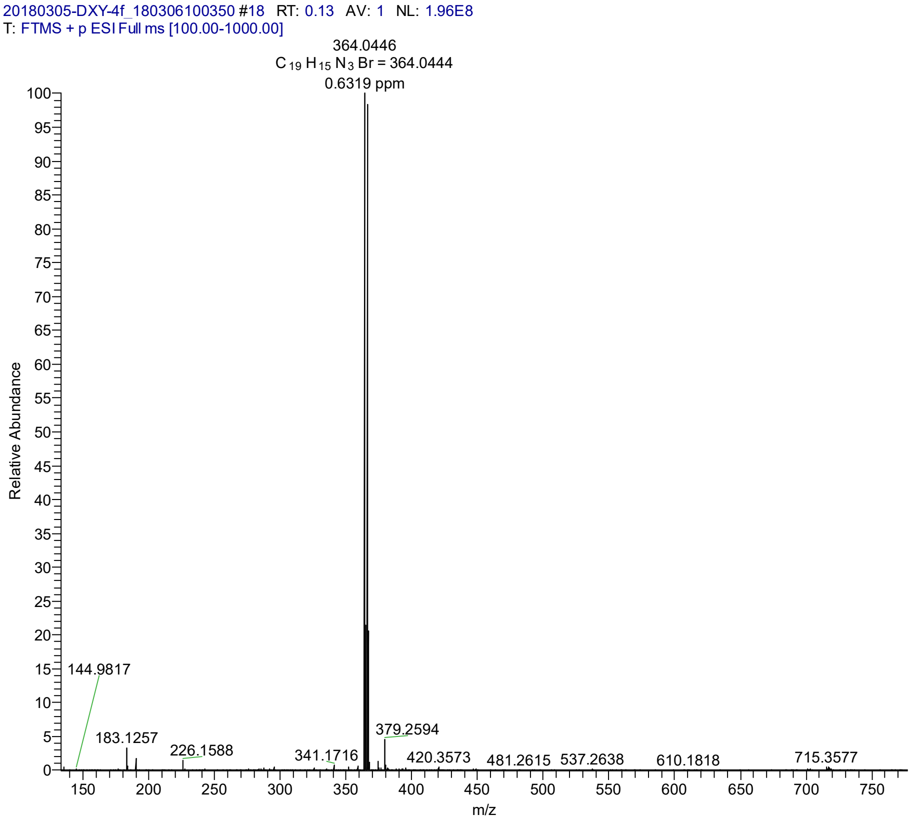

HRMS spectrum of **3g**

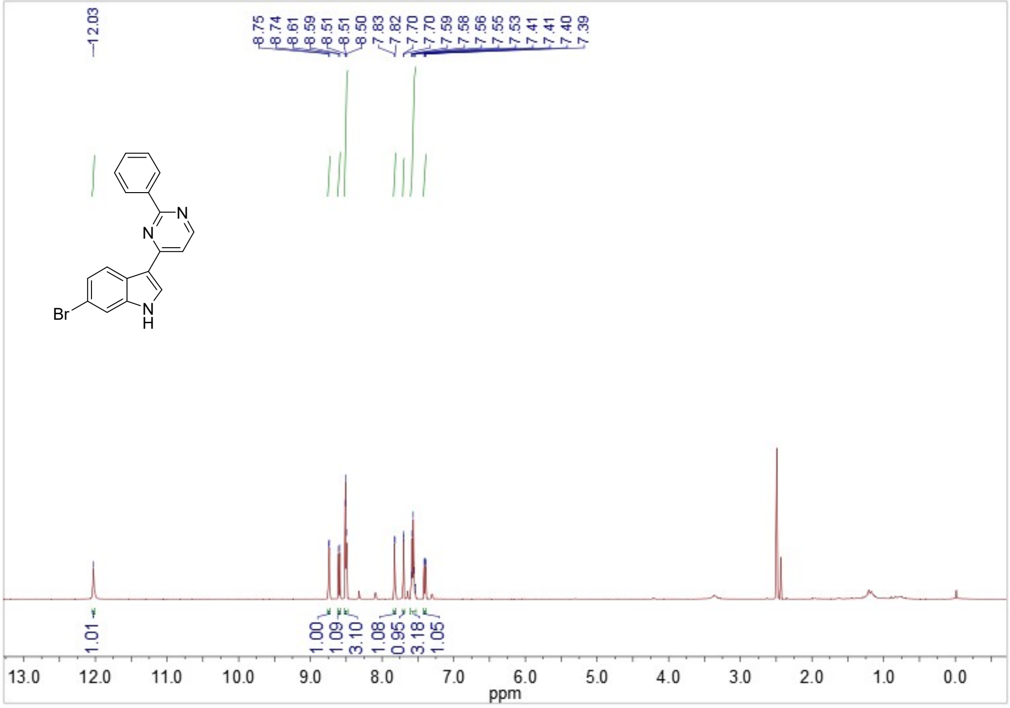

^{1}H NMR Spectrum of **4a**

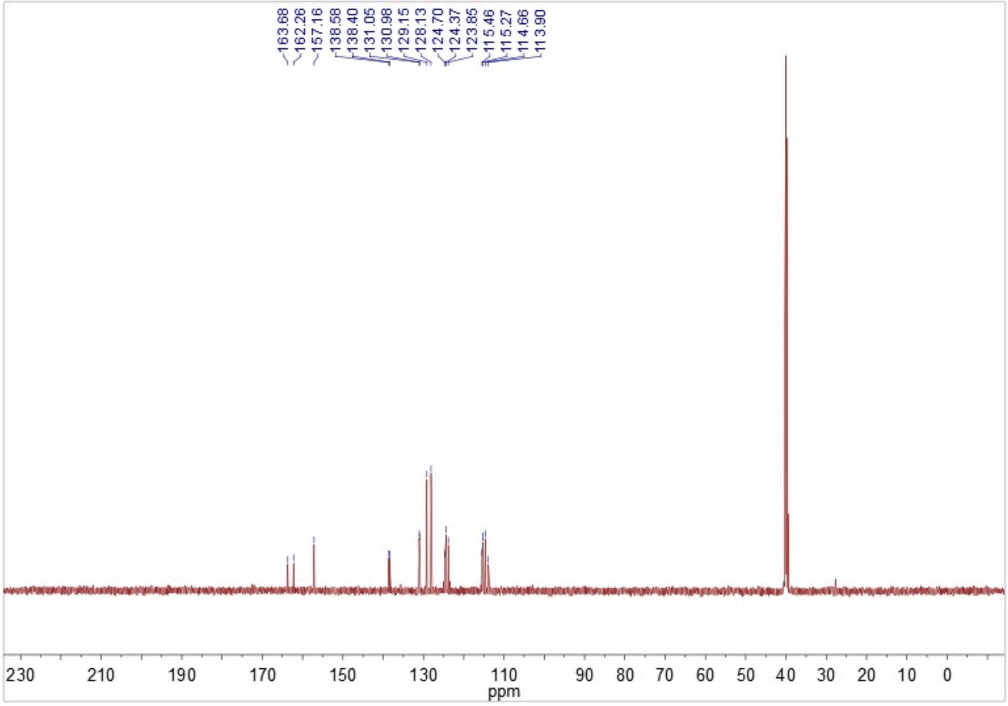

^{13}C NMR Spectrum of **4a**

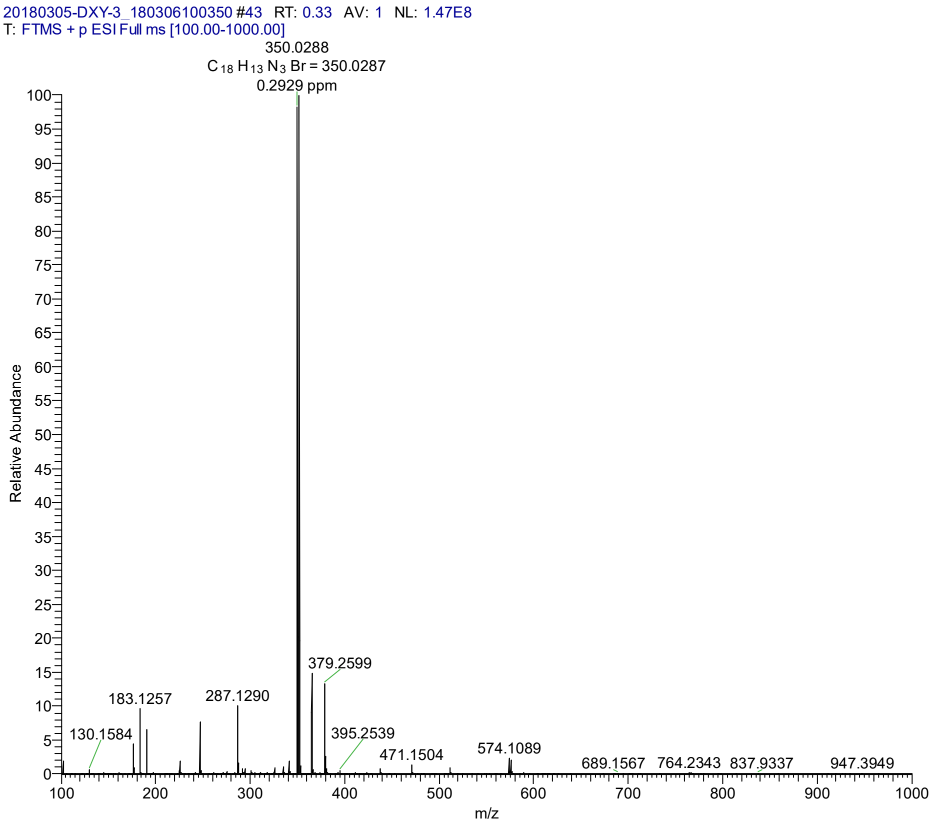

HRMS spectrum of **4a**

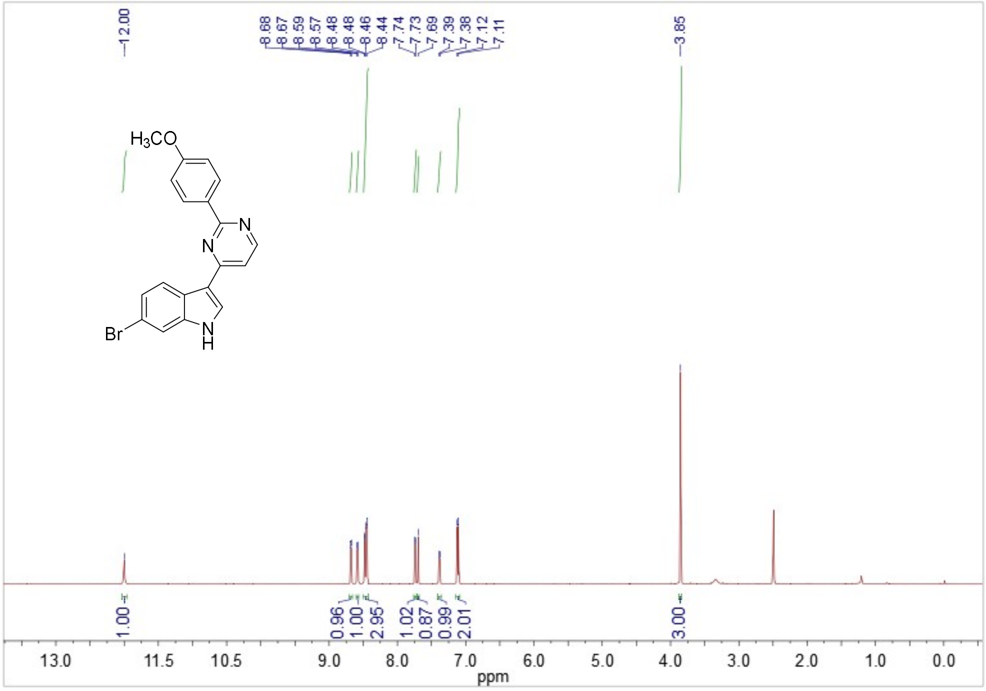

^{1}H NMR Spectrum of **4b**

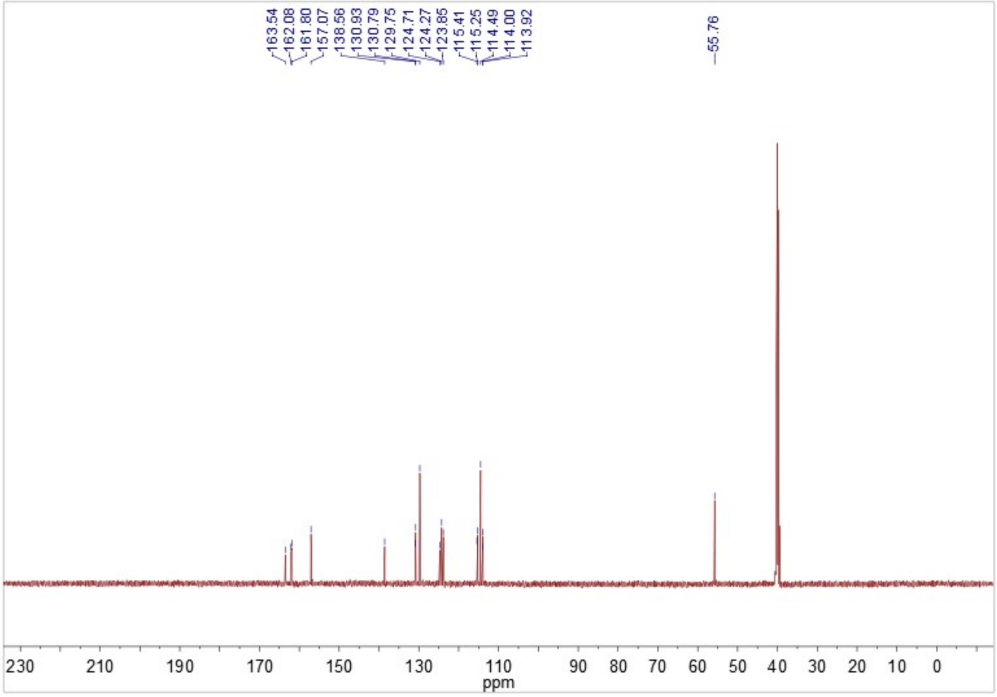

^{13}C NMR Spectrum of **4b**

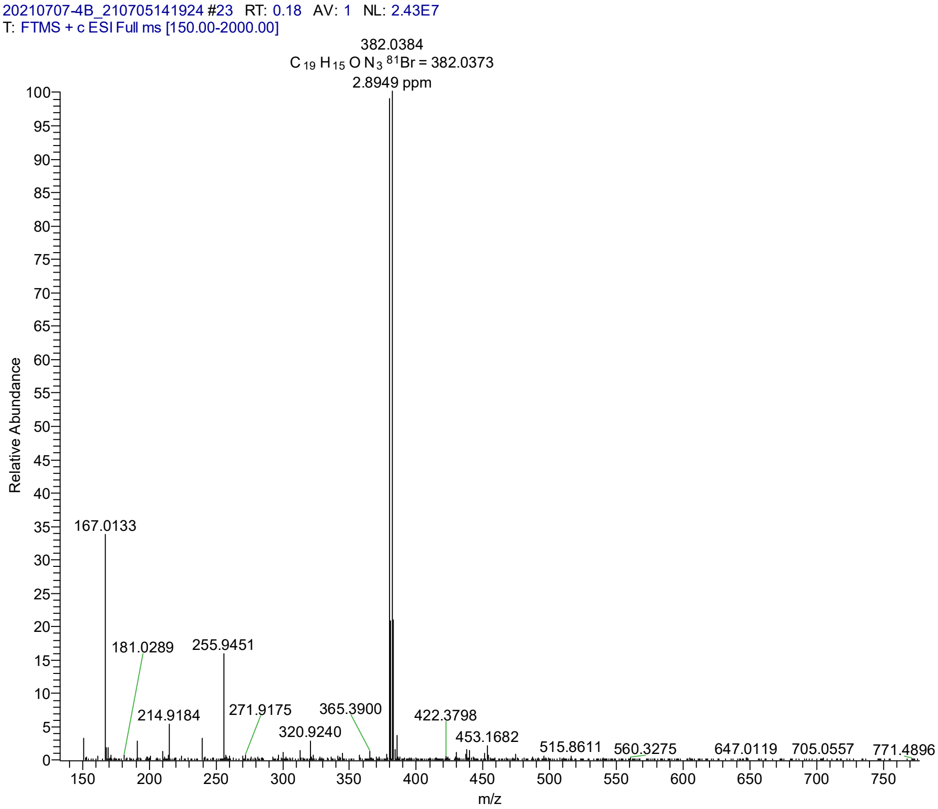

HRMS spectrum of **4b**

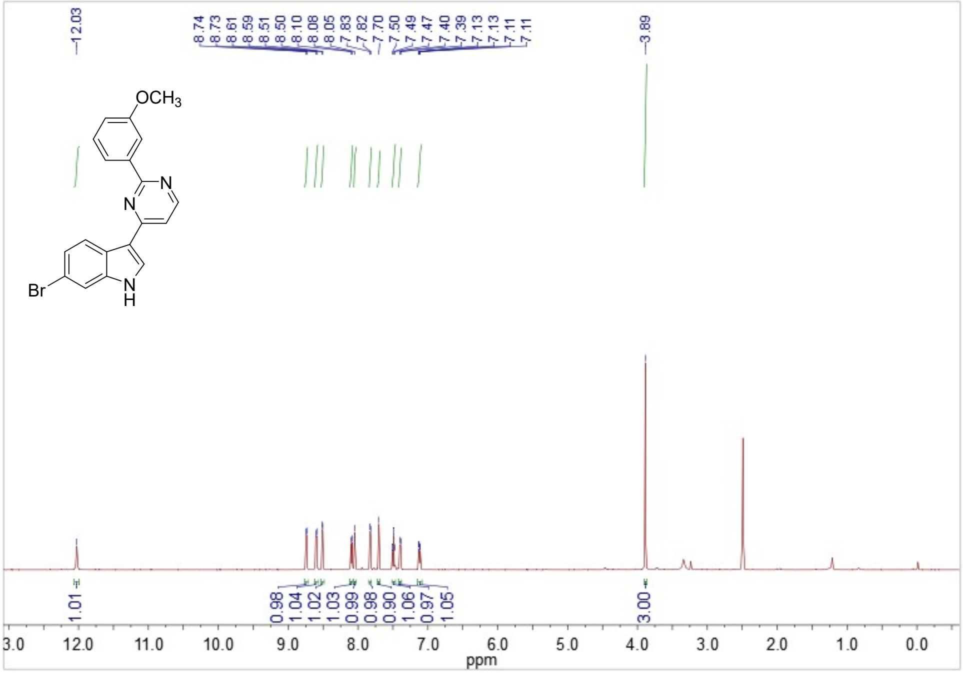

^{1}H NMR Spectrum of **4c**

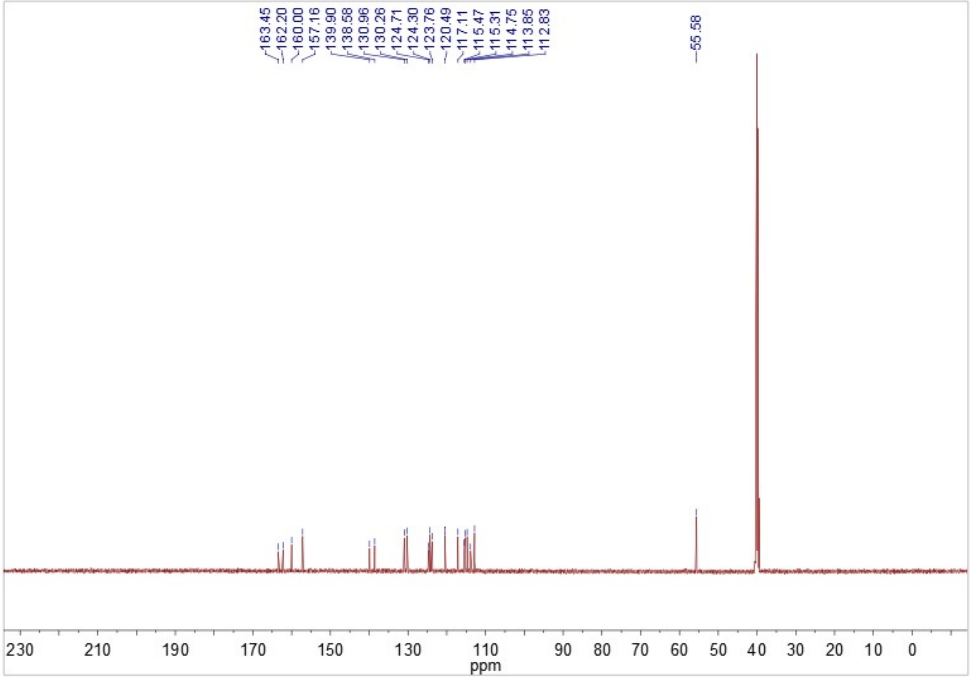

^{13}C NMR Spectrum of **4c**

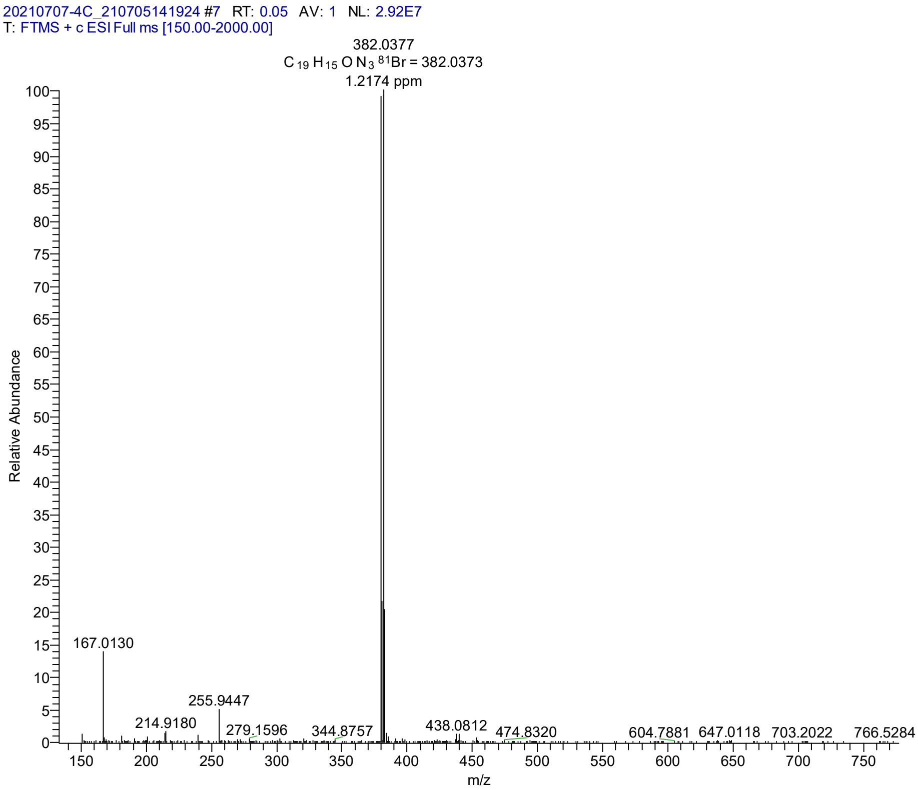

HRMS spectrum of **4c**

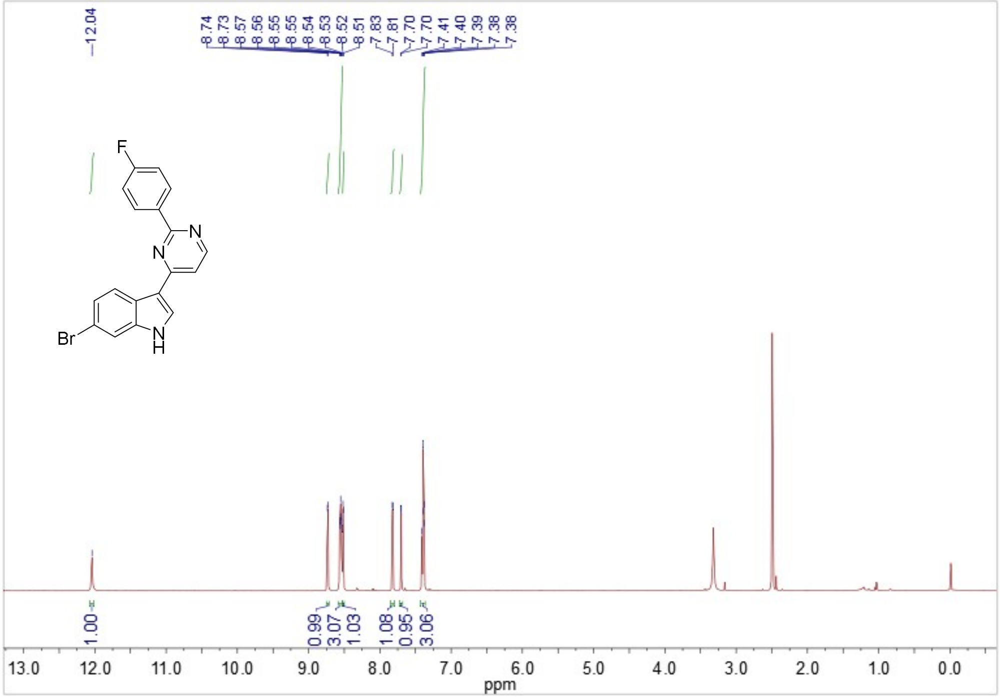

^{1}H NMR Spectrum of **4d**

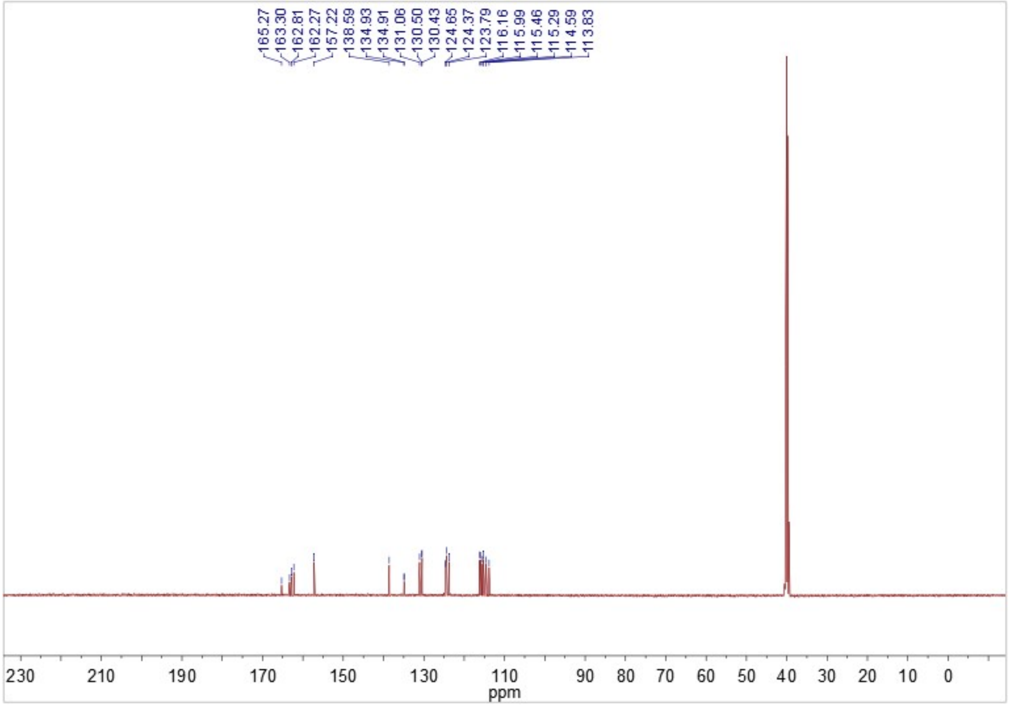

^{13}C NMR Spectrum of **4d**

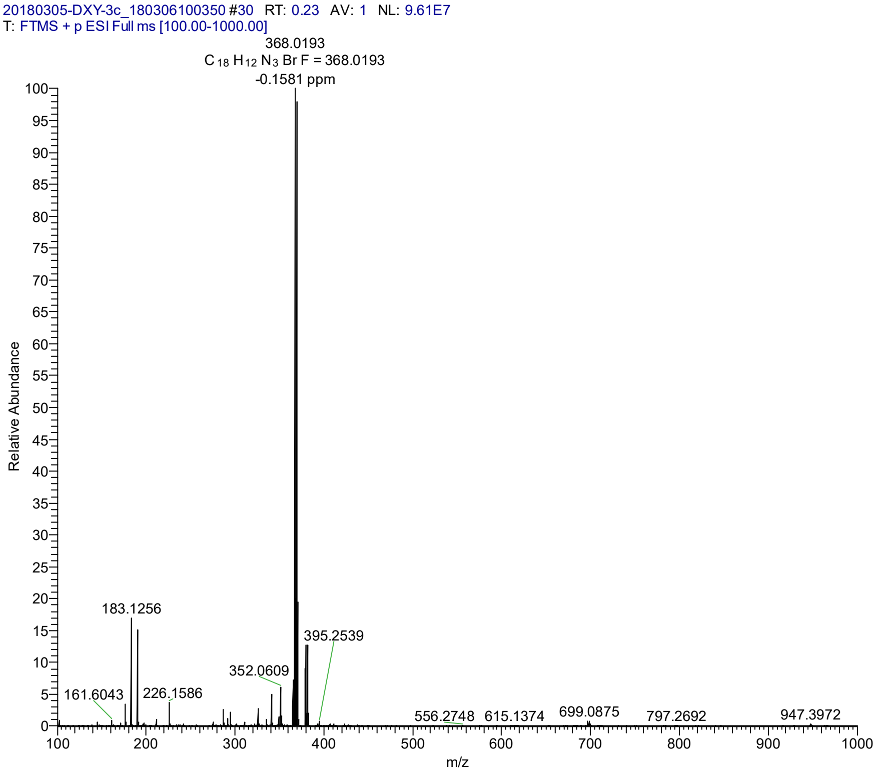

HRMS spectrum of **4d**

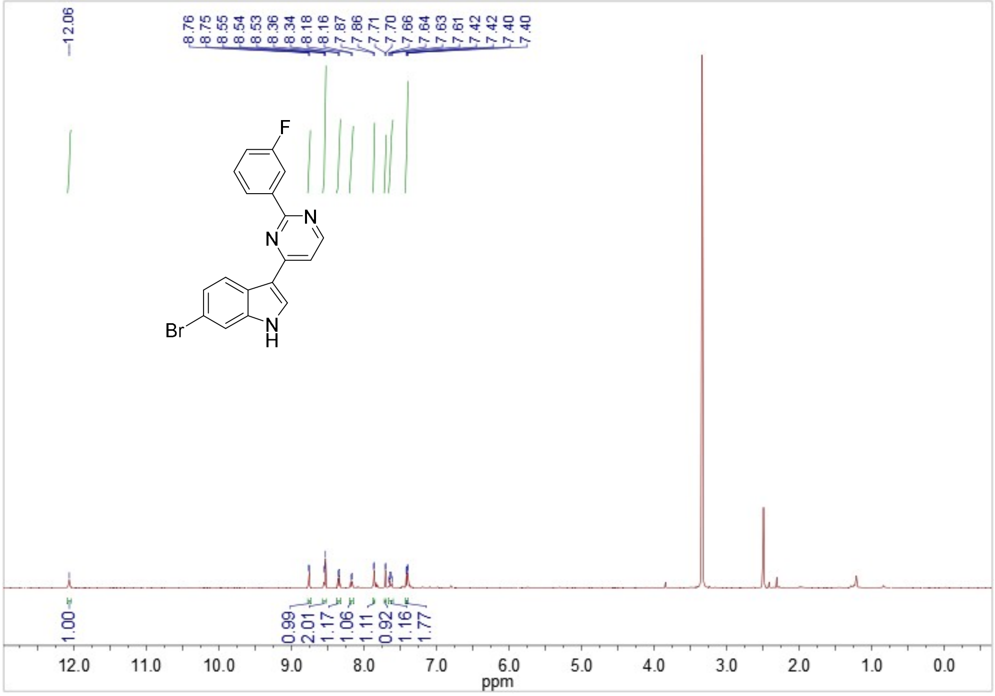

^{1}H NMR Spectrum of **4e**

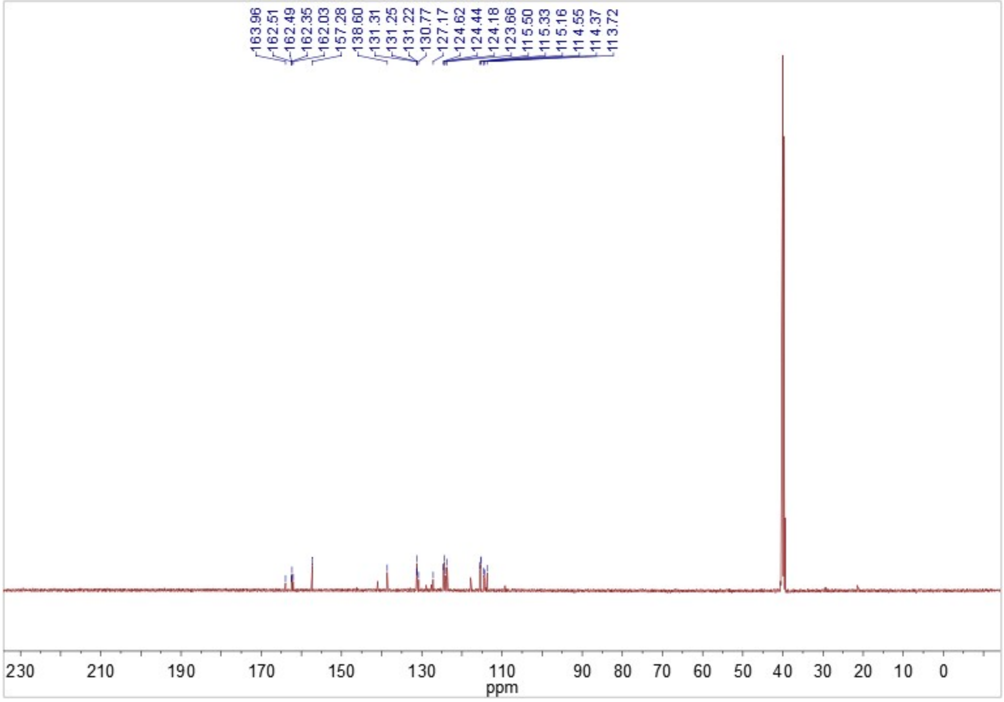

^{13}C NMR Spectrum of **4e**

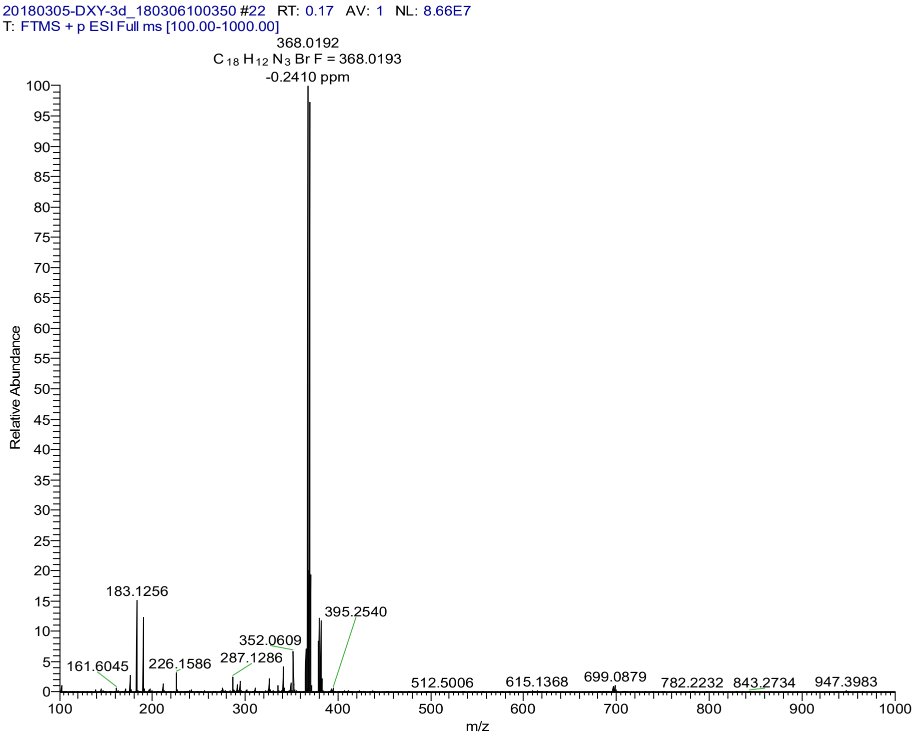

HRMS spectrum of **4e**

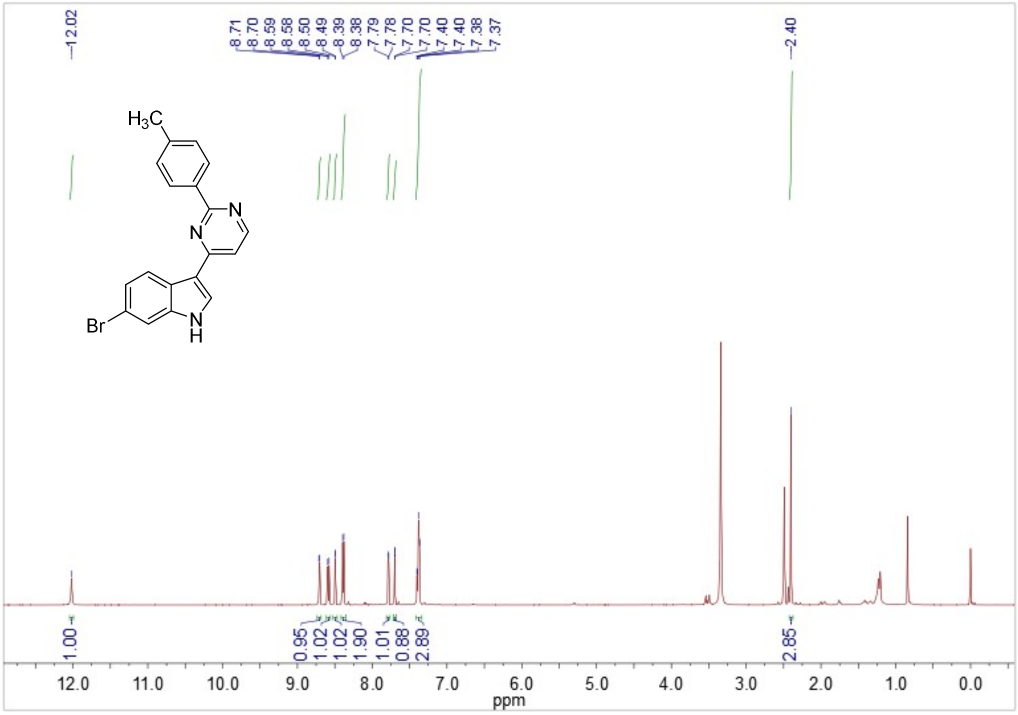

^{1}H NMR Spectrum of **4f**

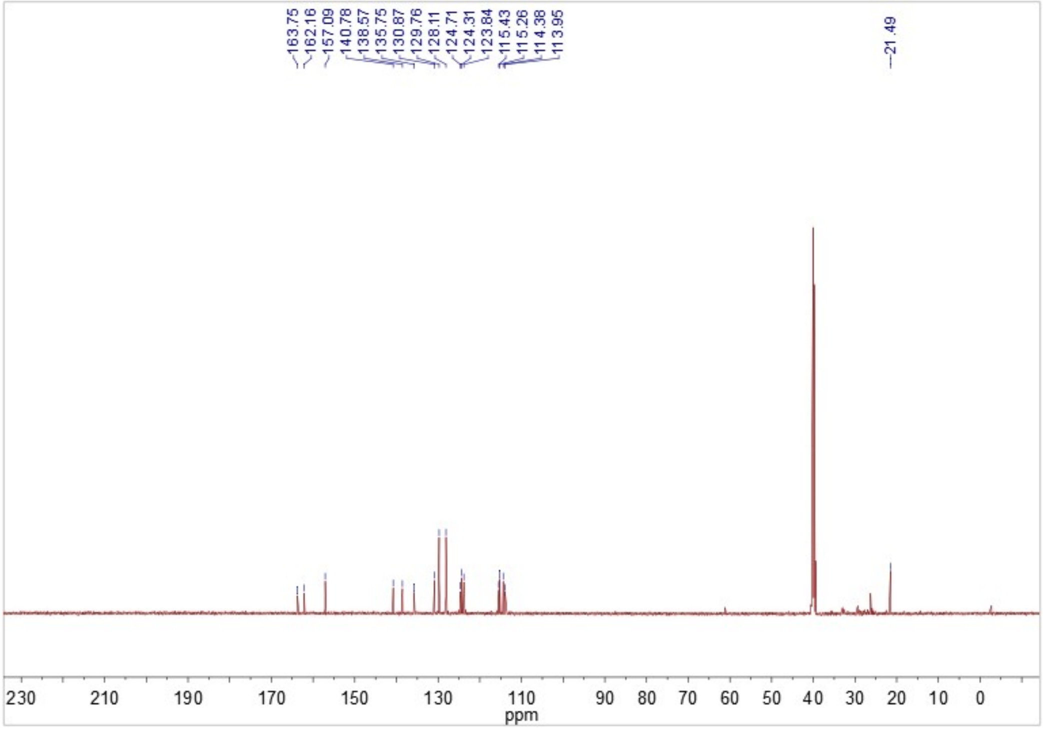

^{13}C NMR Spectrum of **4f**

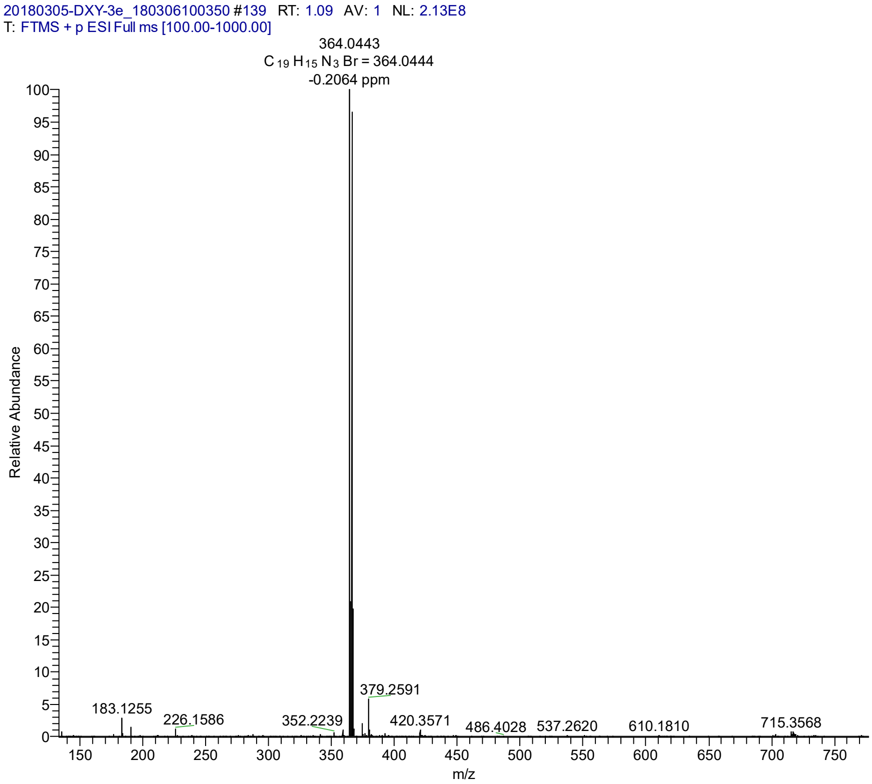

HRMS spectrum of **4f**

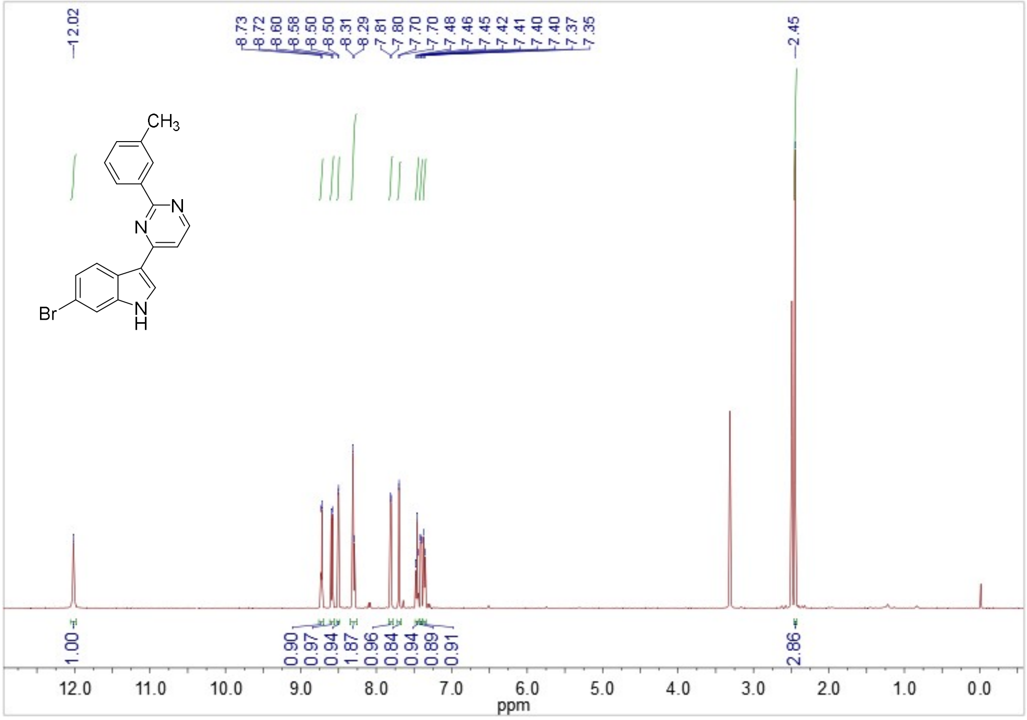

^{1}H NMR Spectrum of **4g**

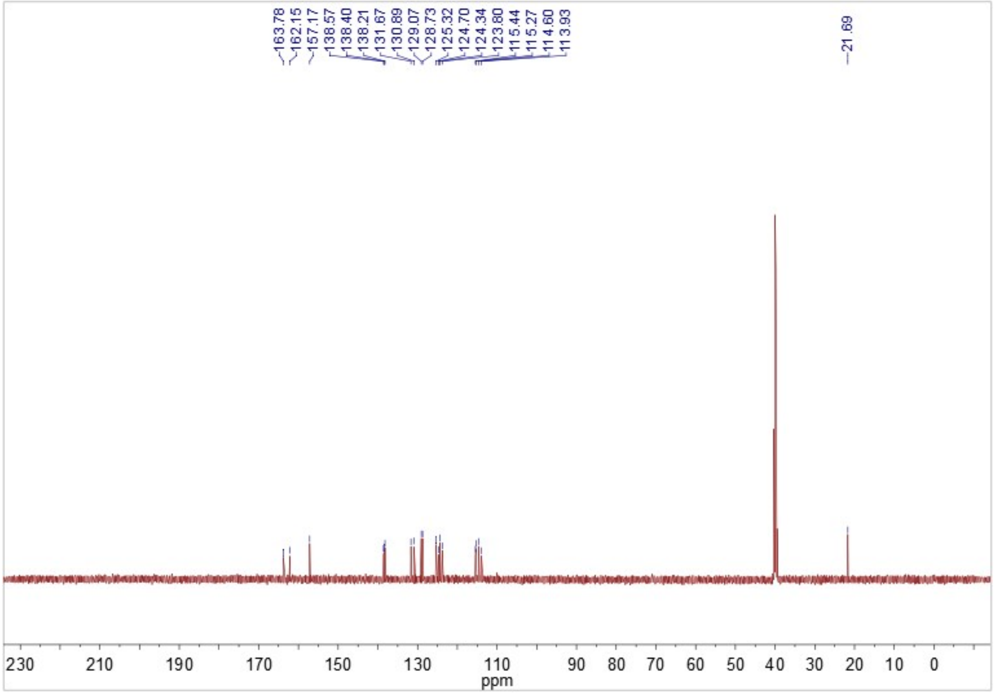

^{13}C NMR Spectrum of **4g**

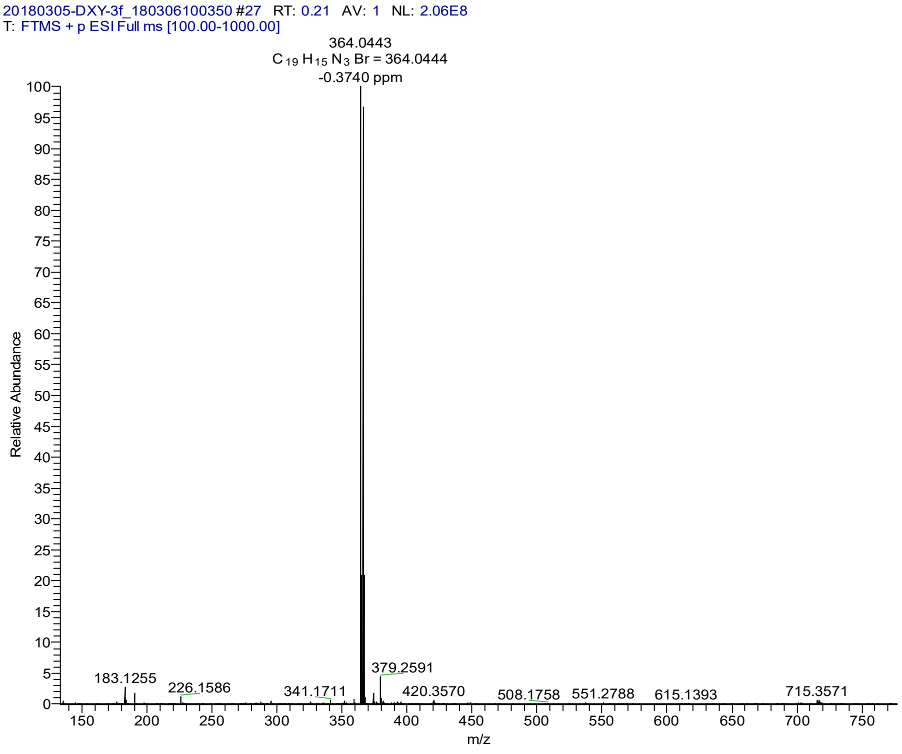

HRMS spectrum of **4g**

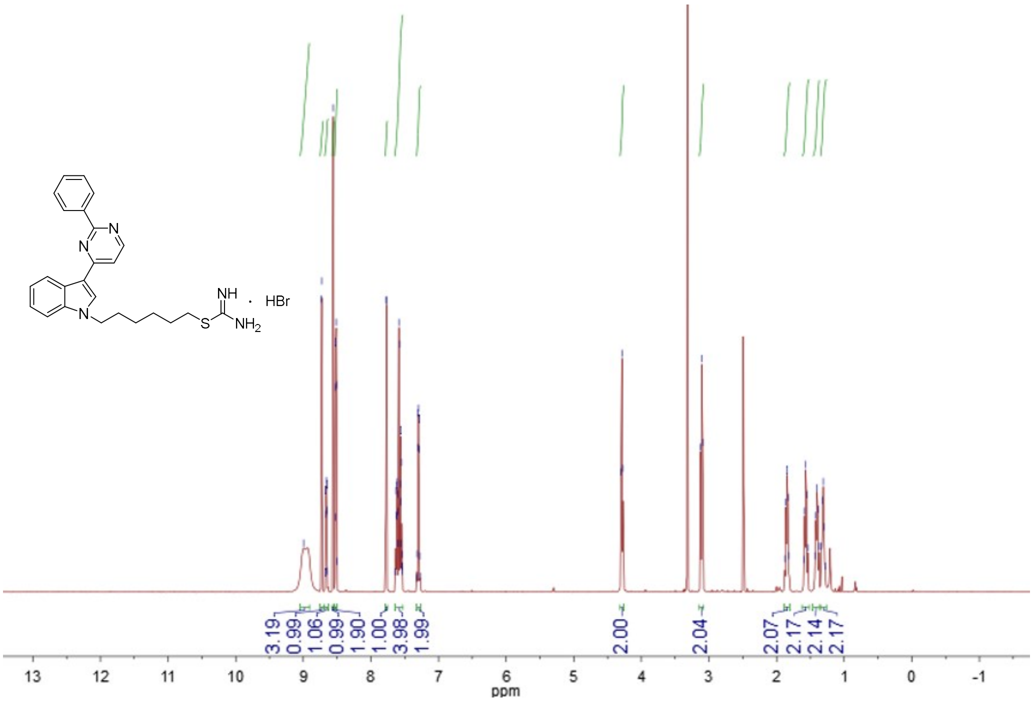

^{1}H NMR Spectrum of **5a**

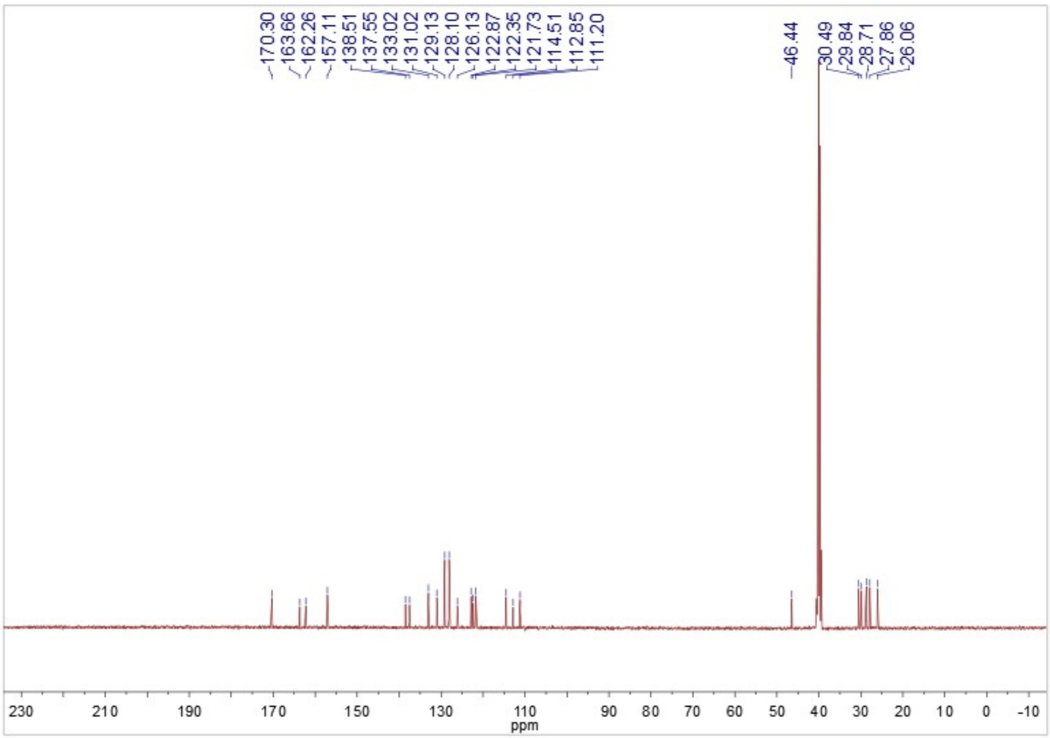

^{13}C NMR Spectrum of **5a**

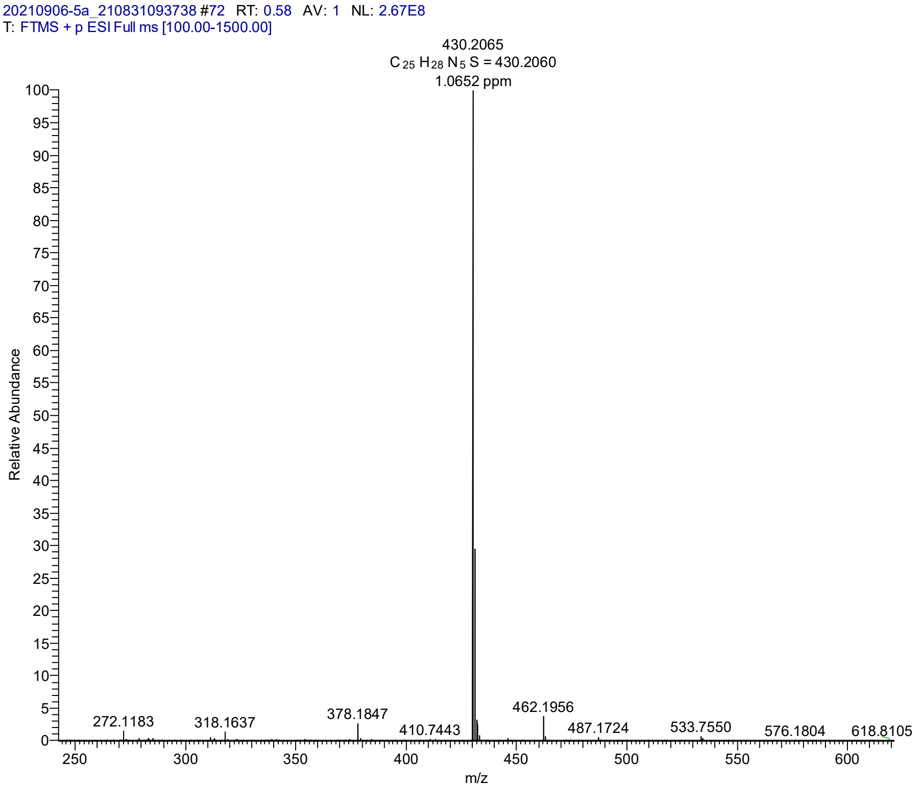

HRMS spectrum of **5a**

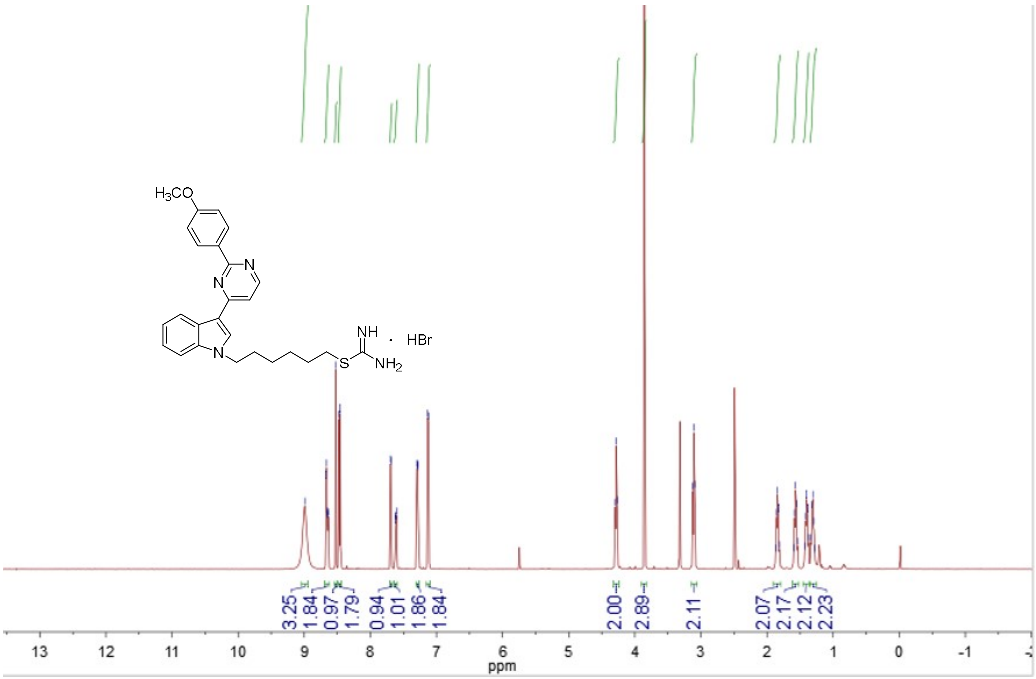

^{1}H NMR Spectrum of **5b**

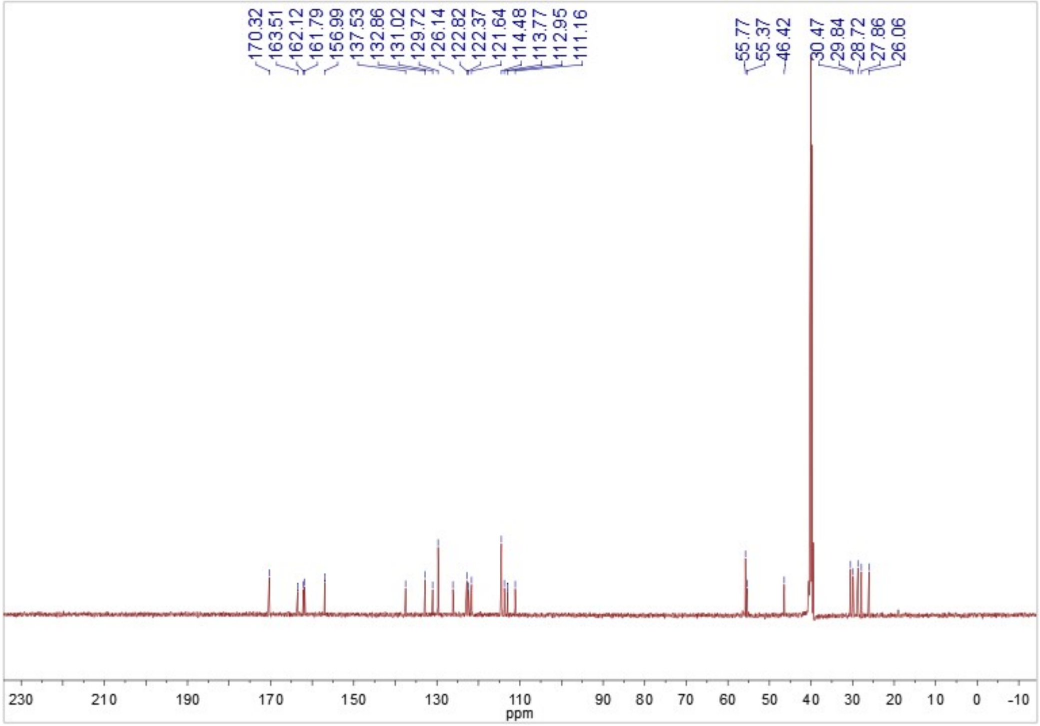

^{13}C NMR Spectrum of **5b**

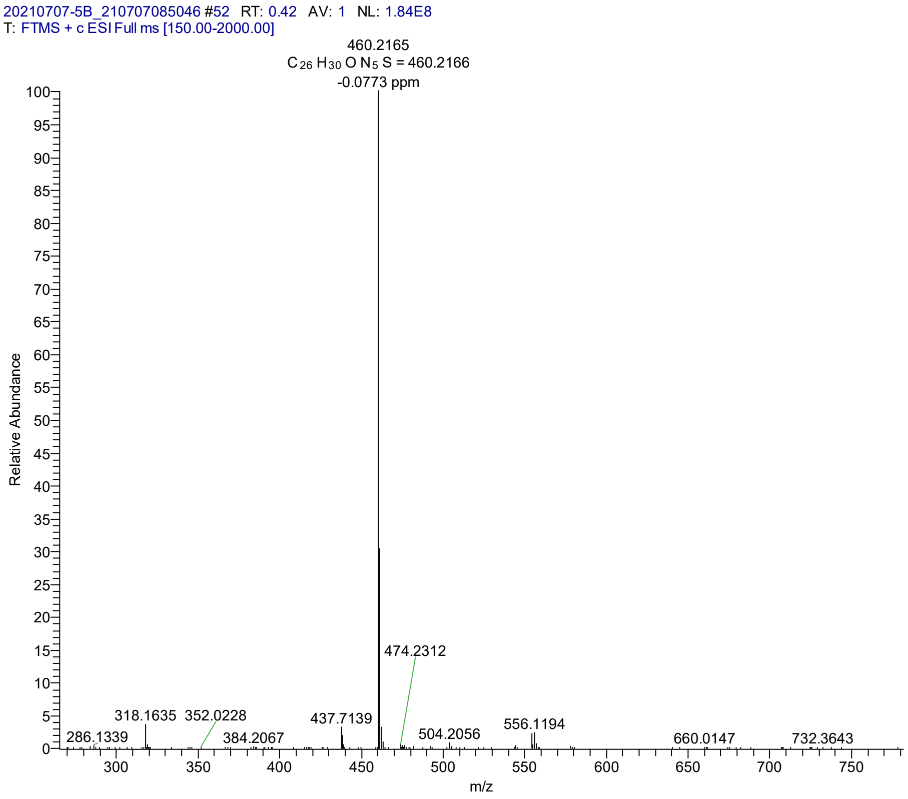

HRMS spectrum of **5b**

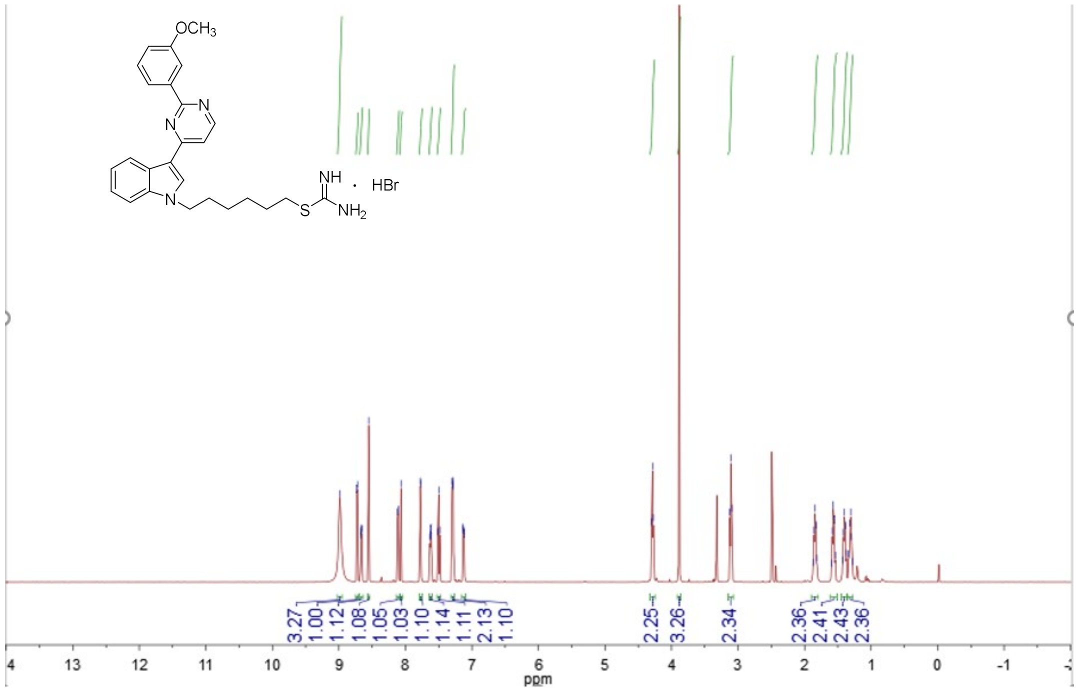

^{1}H NMR Spectrum of **5c**

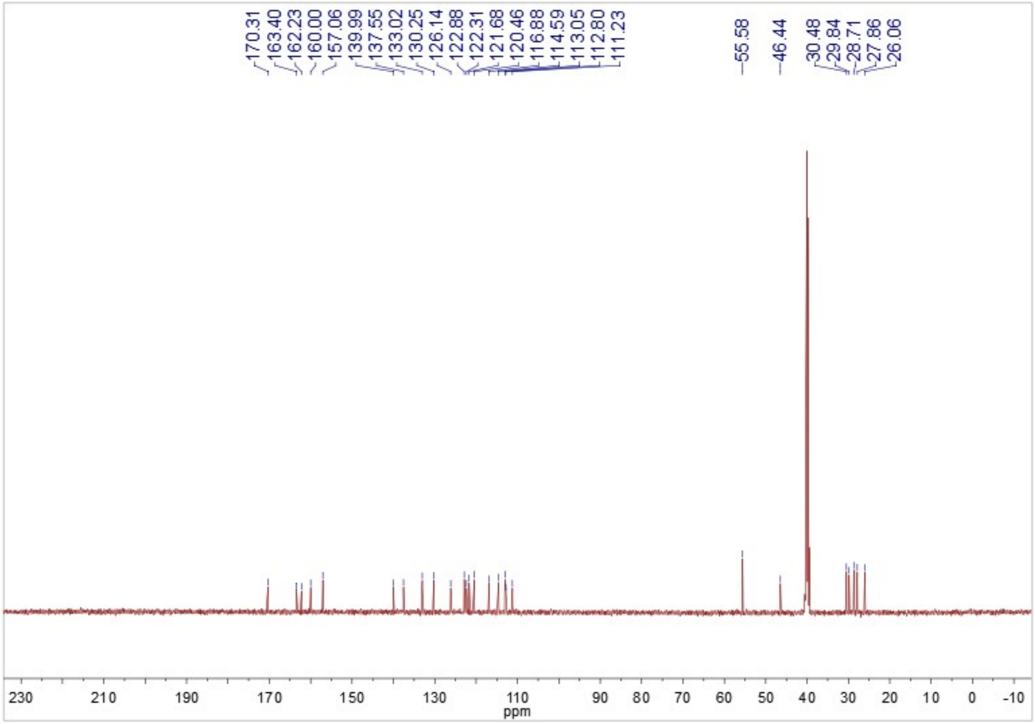

^{13}C NMR Spectrum of **5c**

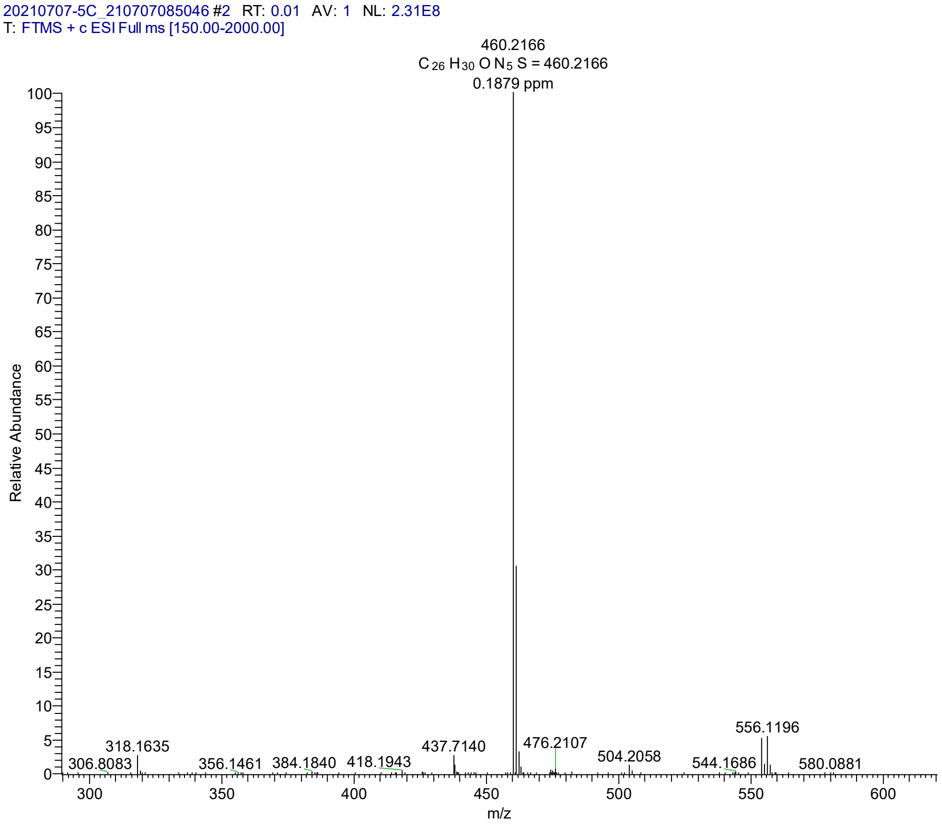

HRMS spectrum of **5c**

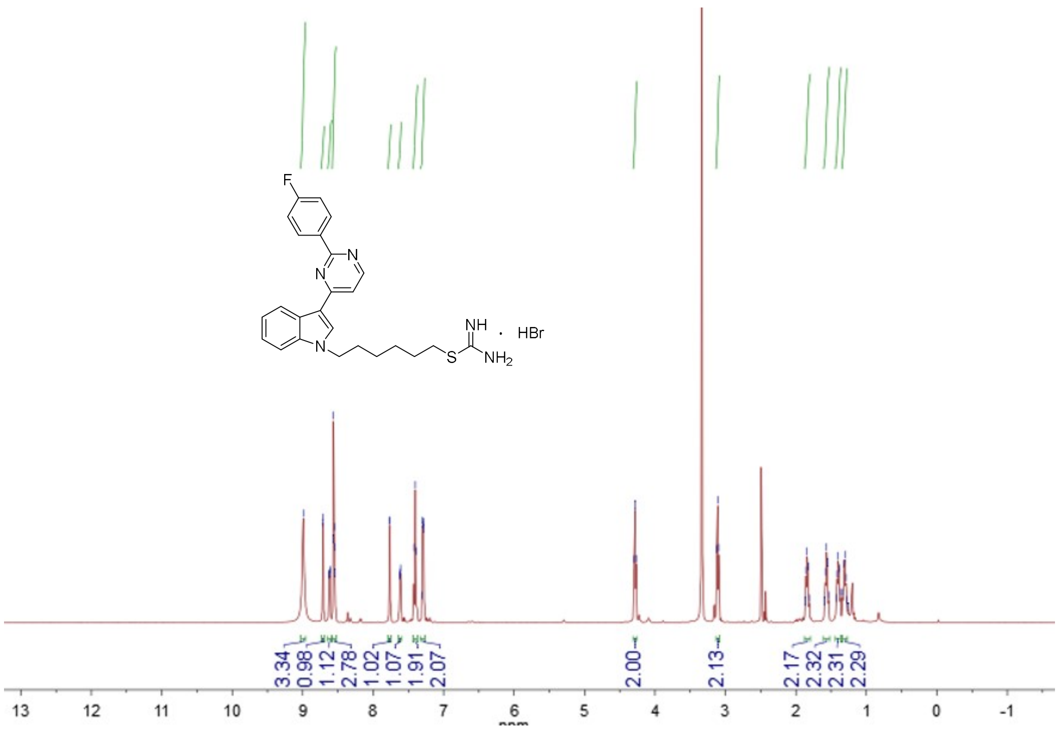

^{1}H NMR Spectrum of **5d**

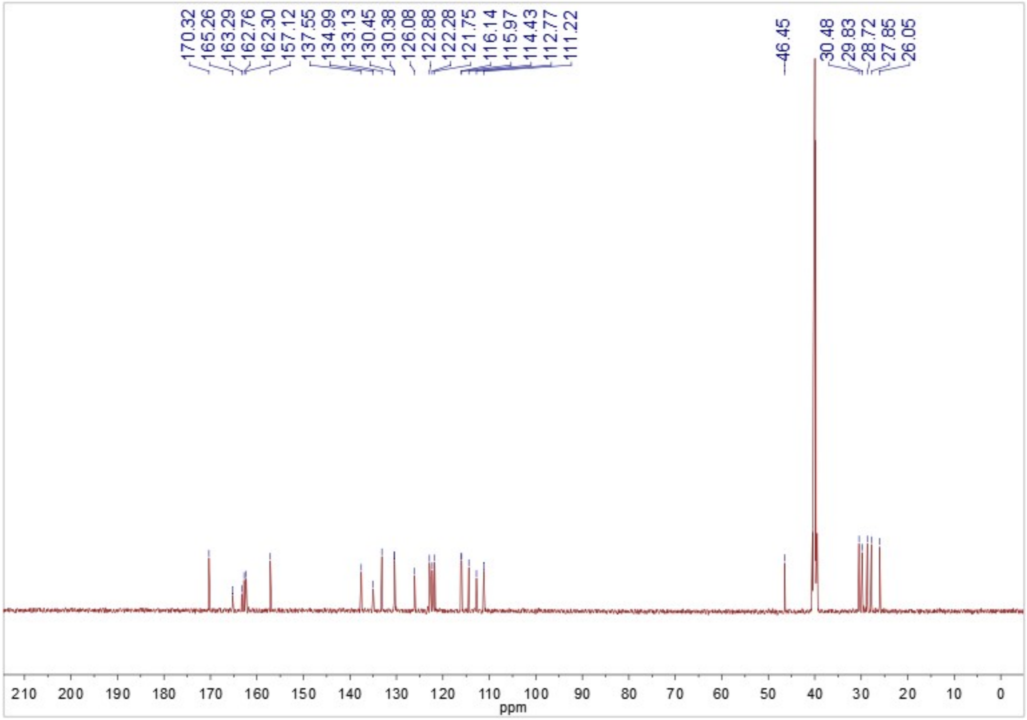

^{13}C NMR Spectrum of **5d**

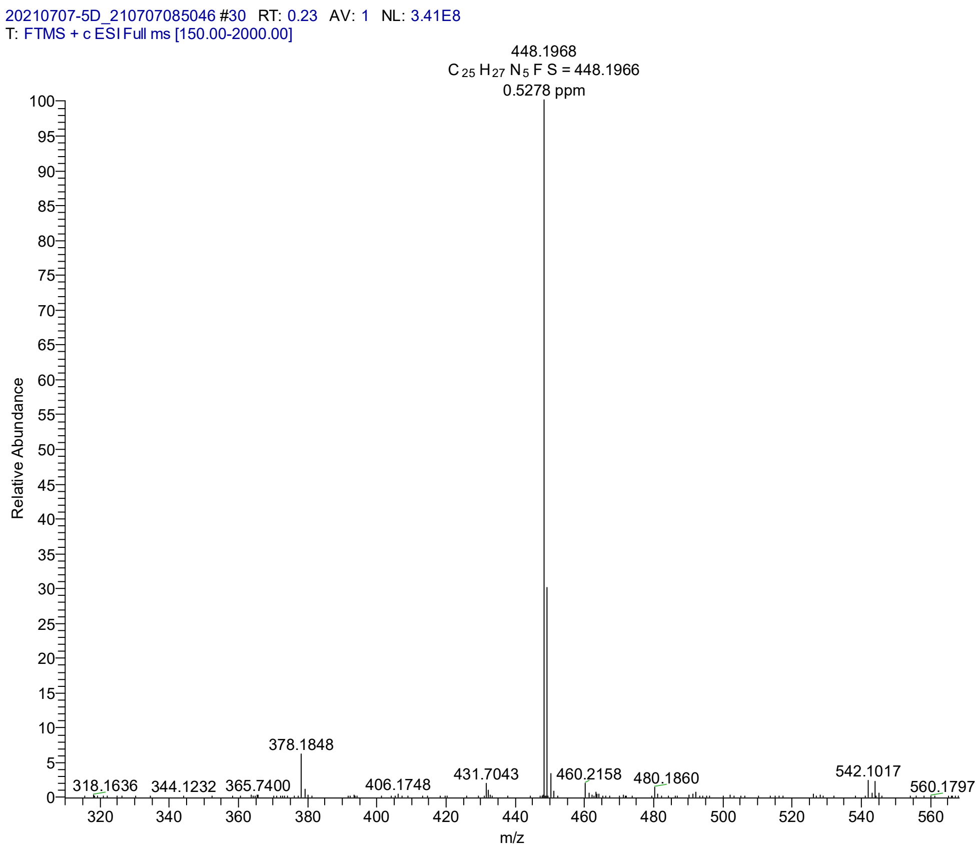

HRMS spectrum of **5d**

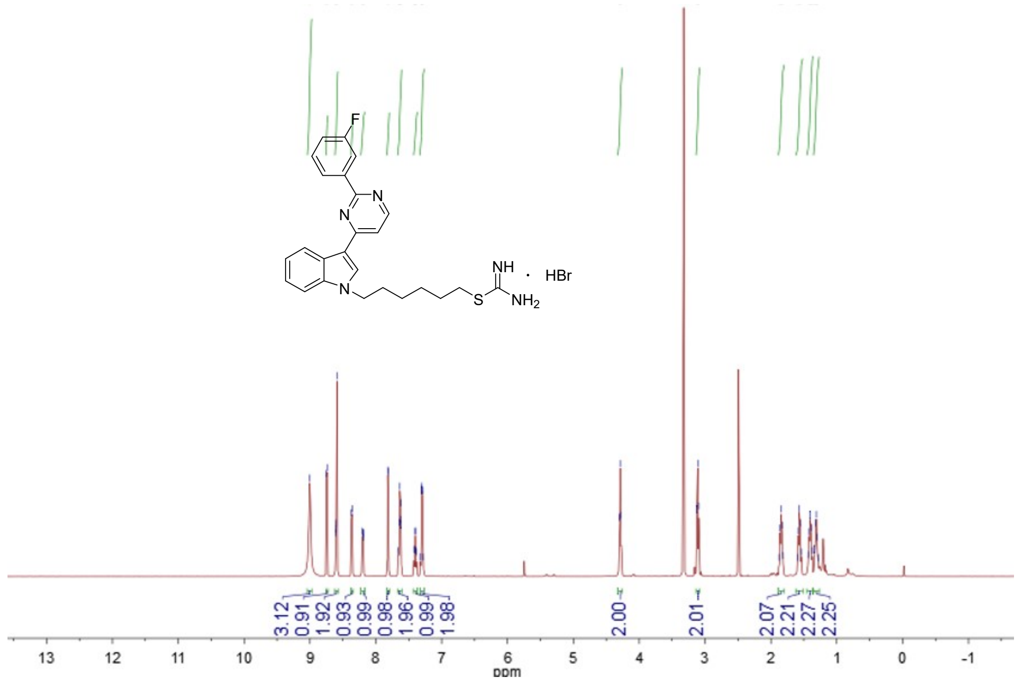

^{1}H NMR Spectrum of **5e**

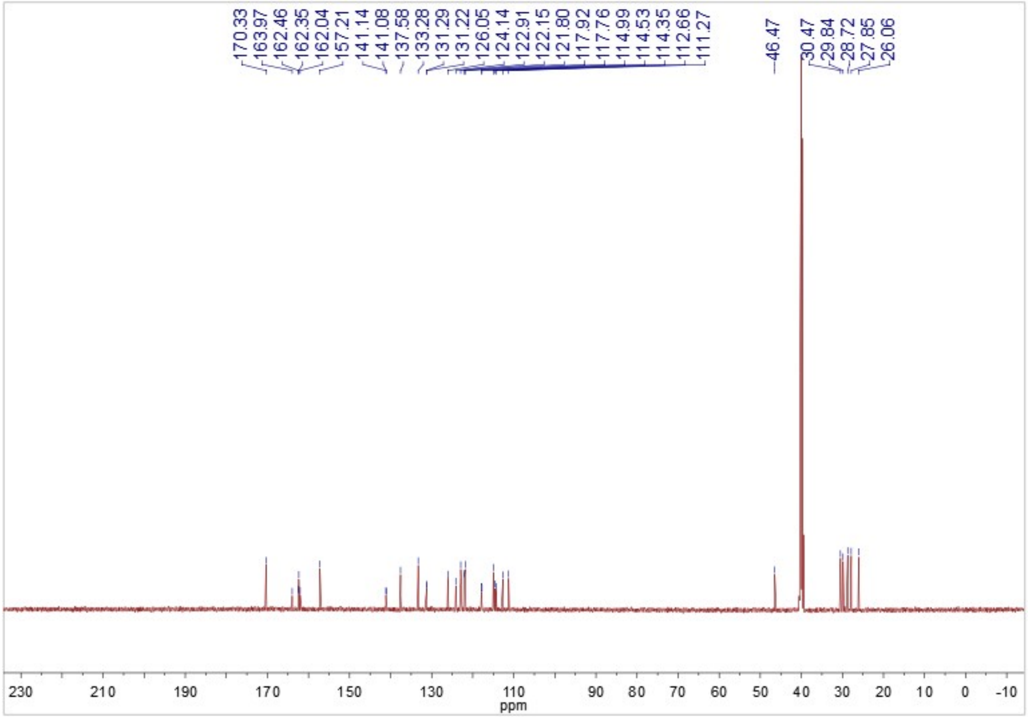

^{13}C NMR Spectrum of **5e**

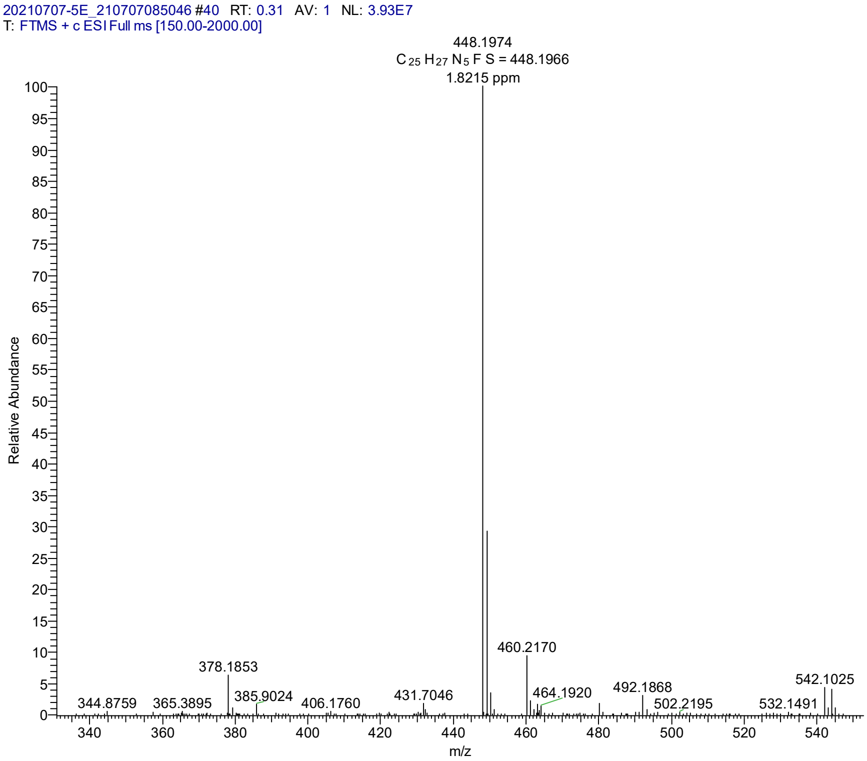

HRMS spectrum of **5e**

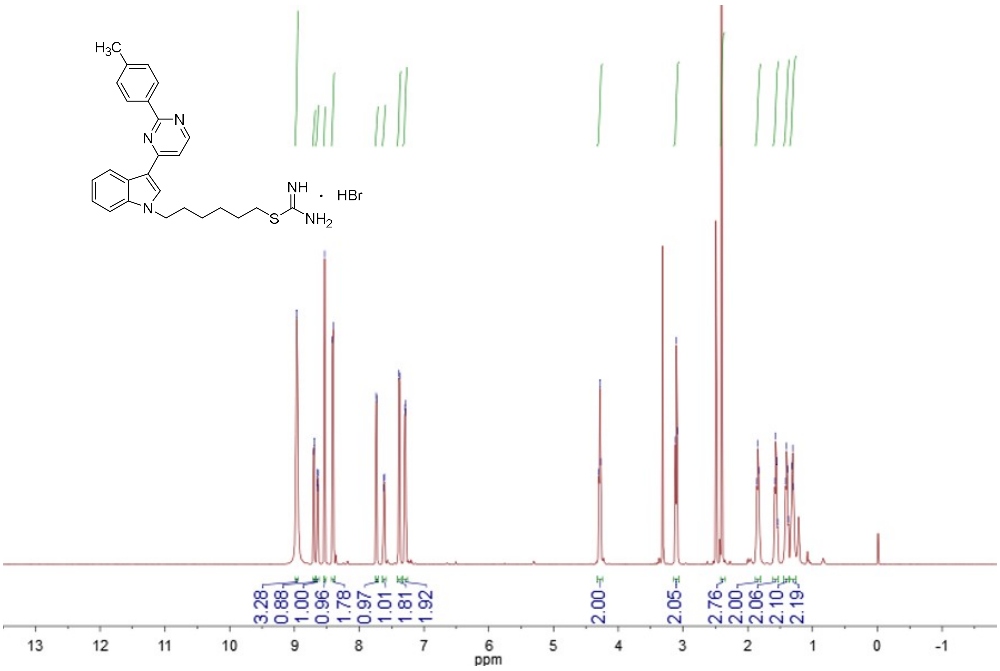

^{1}H NMR Spectrum of **5f**

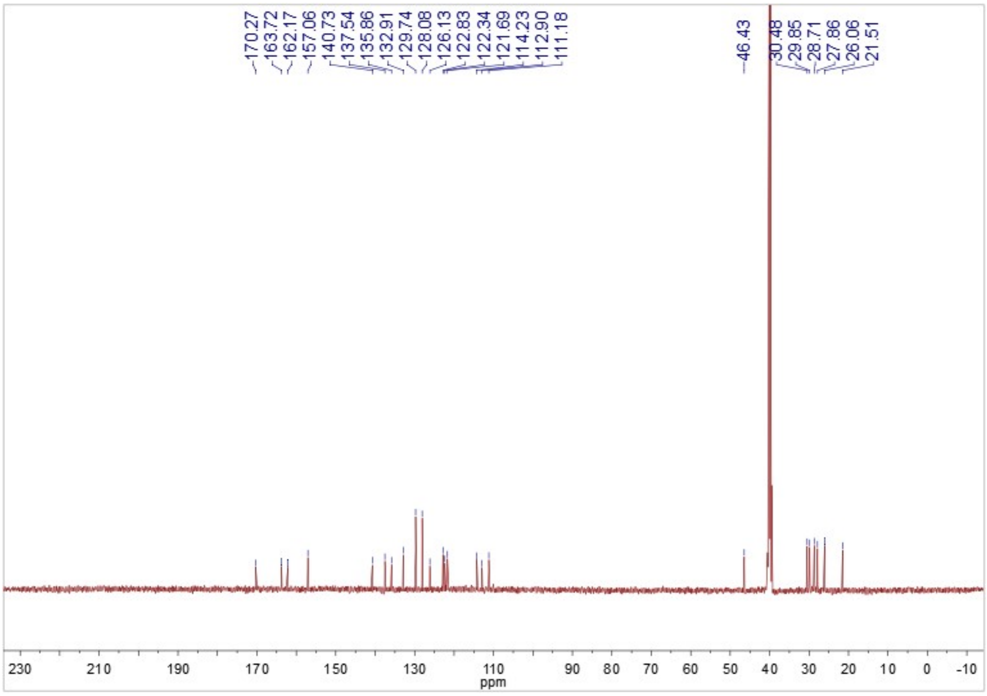

^{13}C NMR Spectrum of **5f**

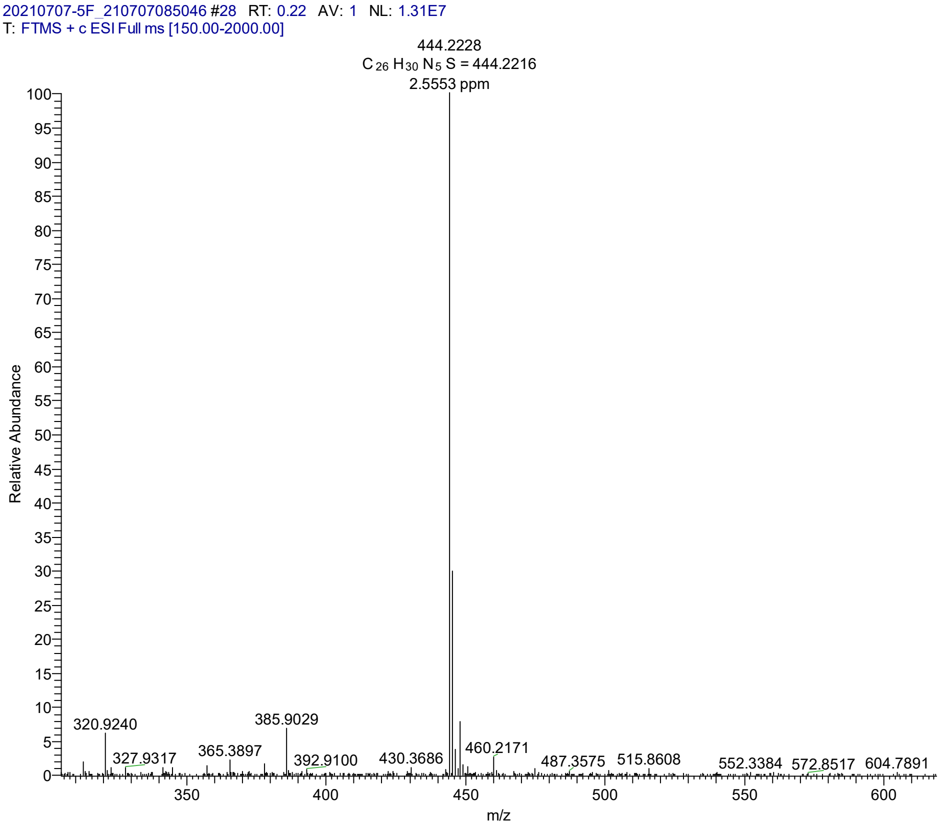

HRMS spectrum of **5f**

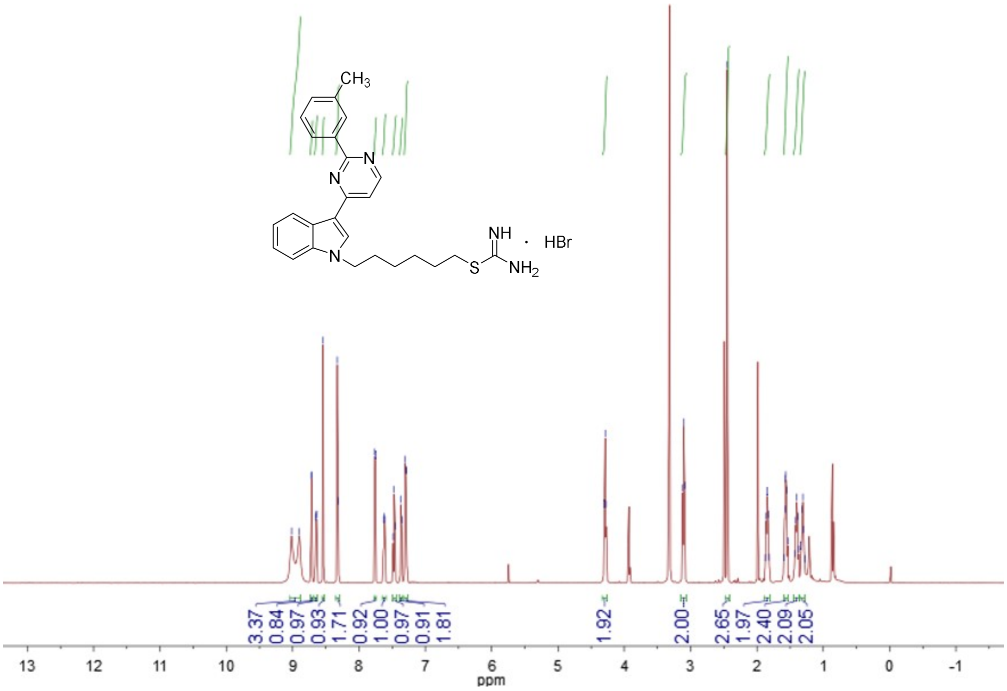

^{1}H NMR Spectrum of **5g**

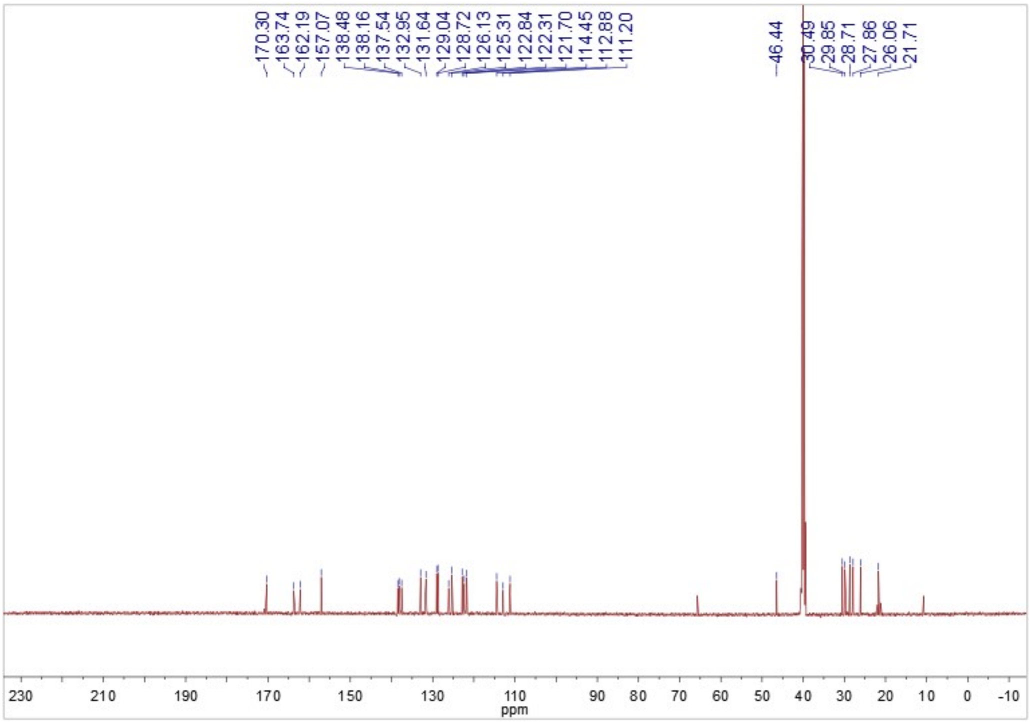

^{13}C NMR Spectrum of **5g**

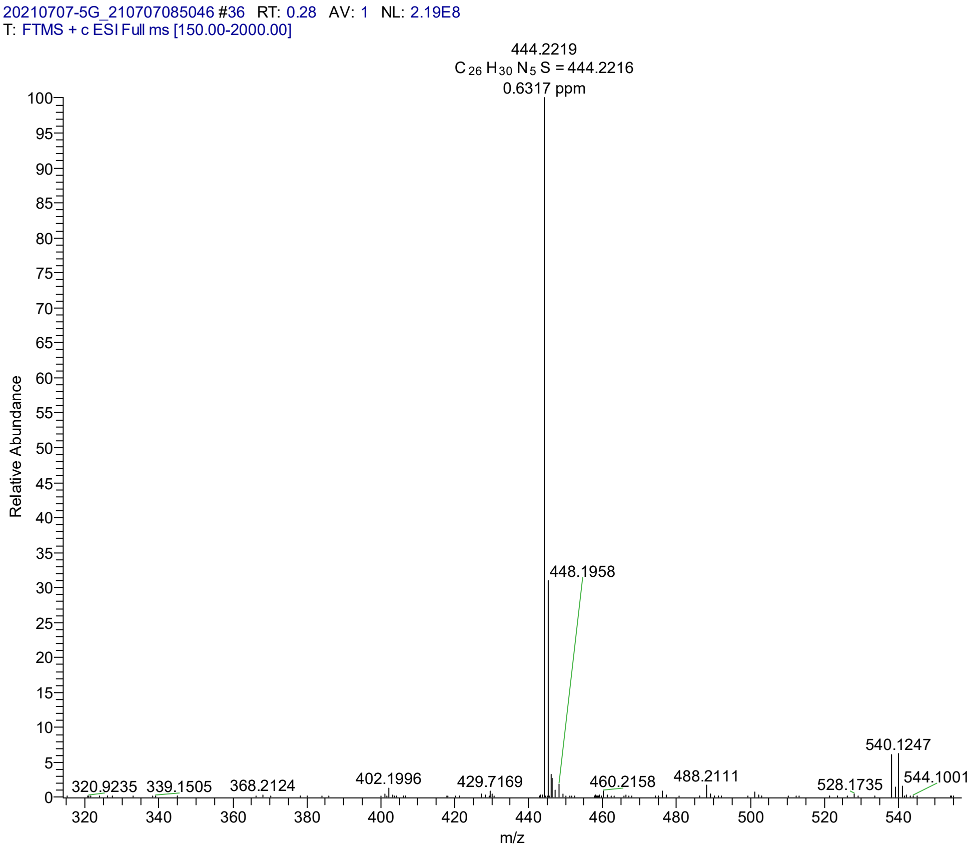

HRMS spectrum of **5g**

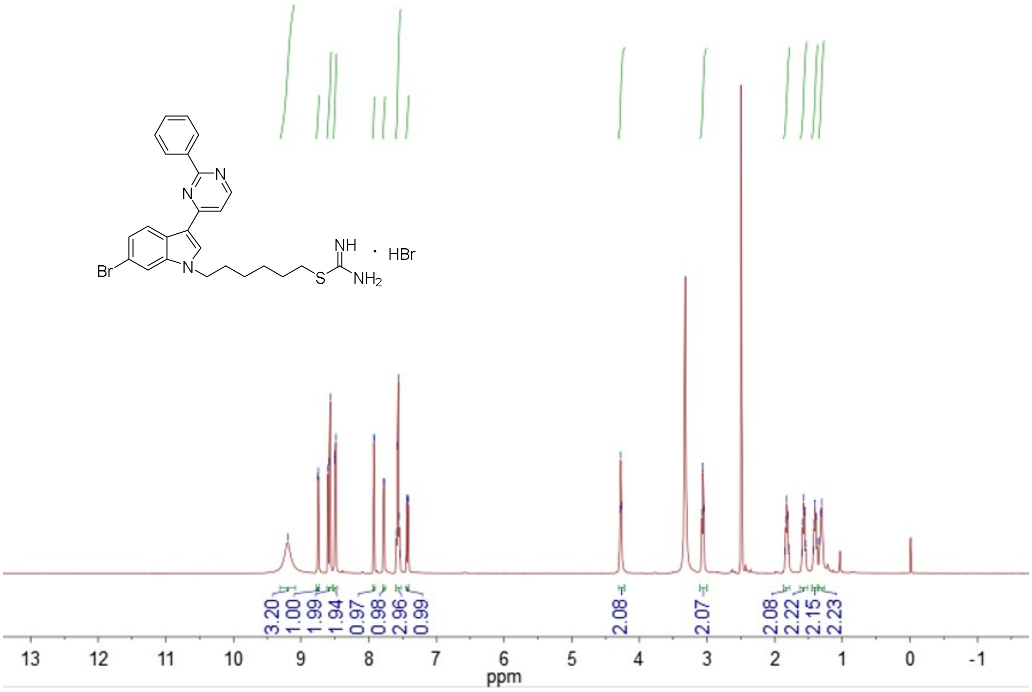

^{1}H NMR Spectrum of **6a**

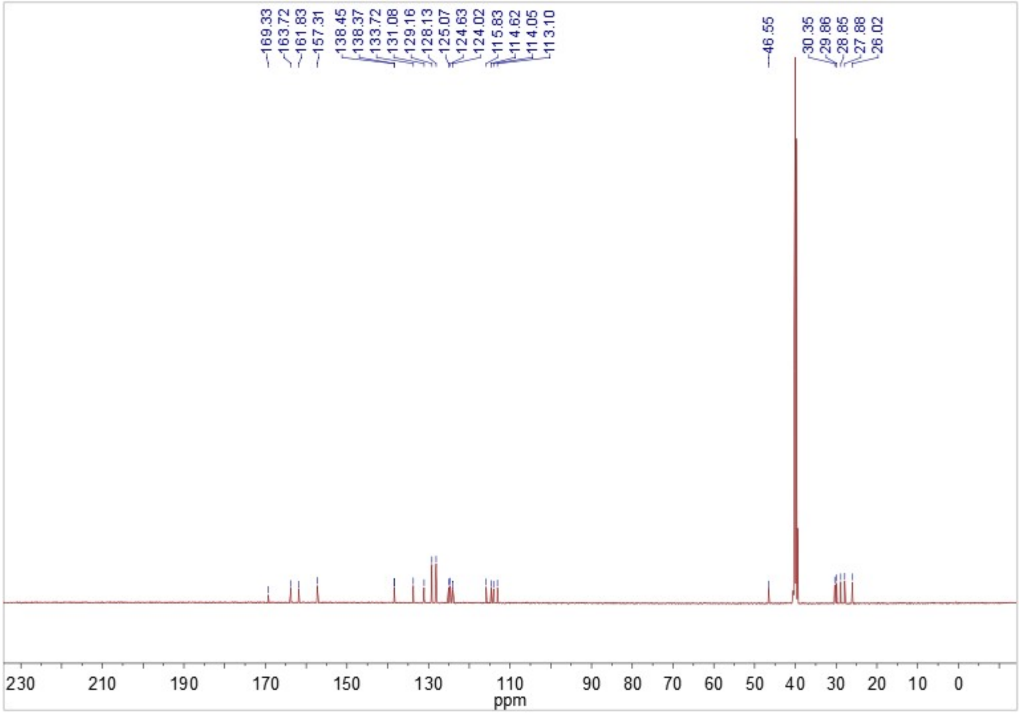

^{13}C NMR Spectrum of **6a**

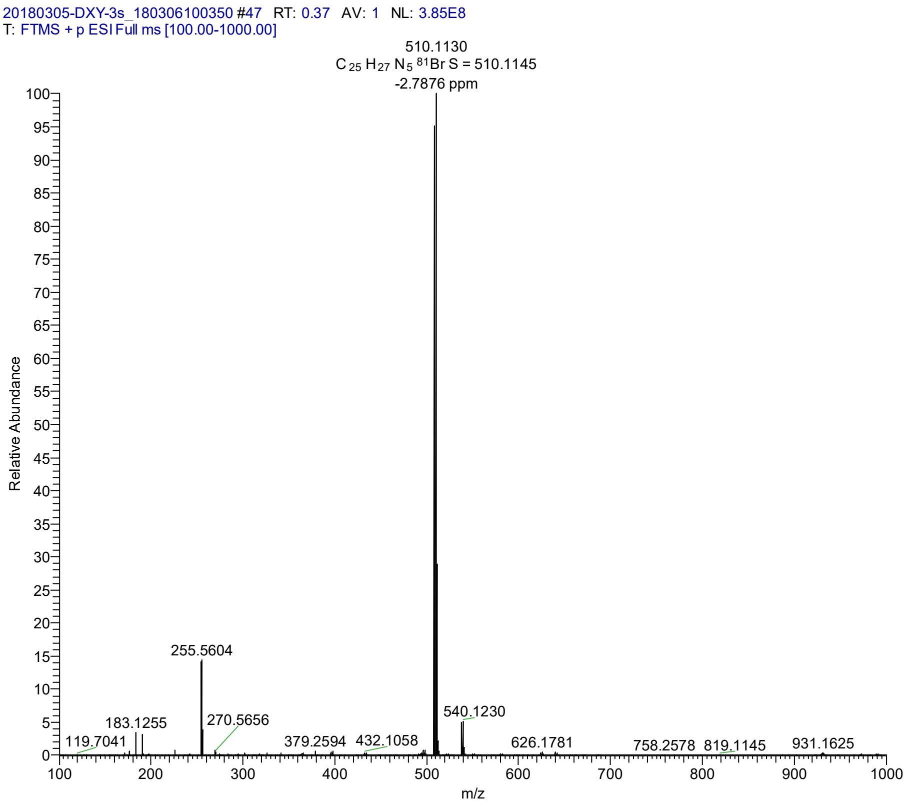

HRMS spectrum of **6a**

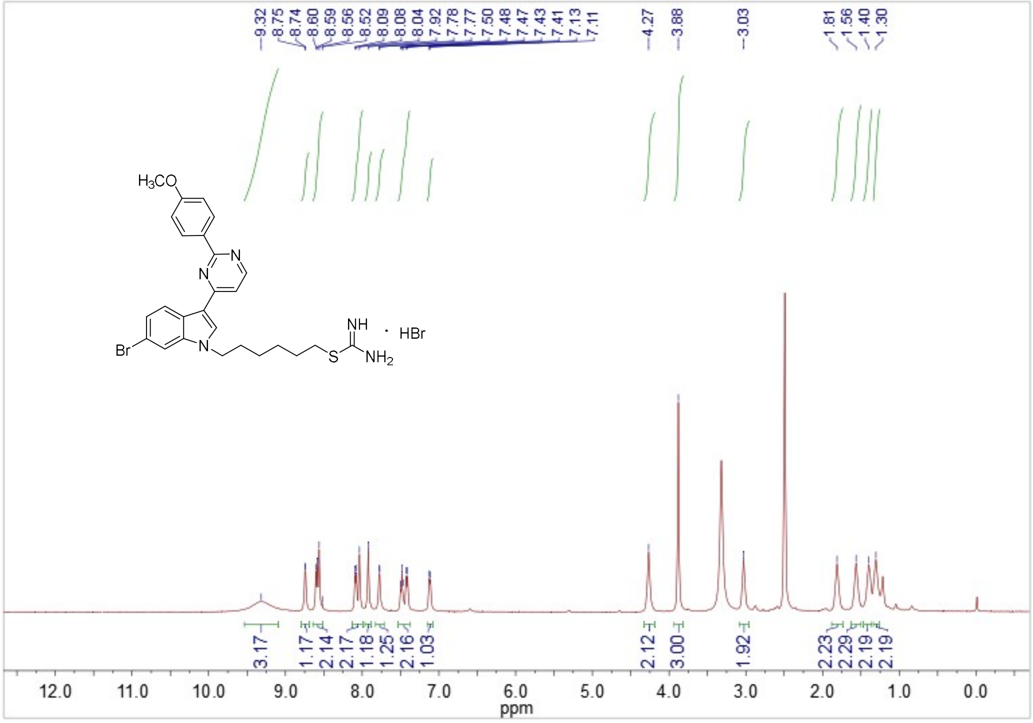

^{1}H NMR Spectrum of **6b**

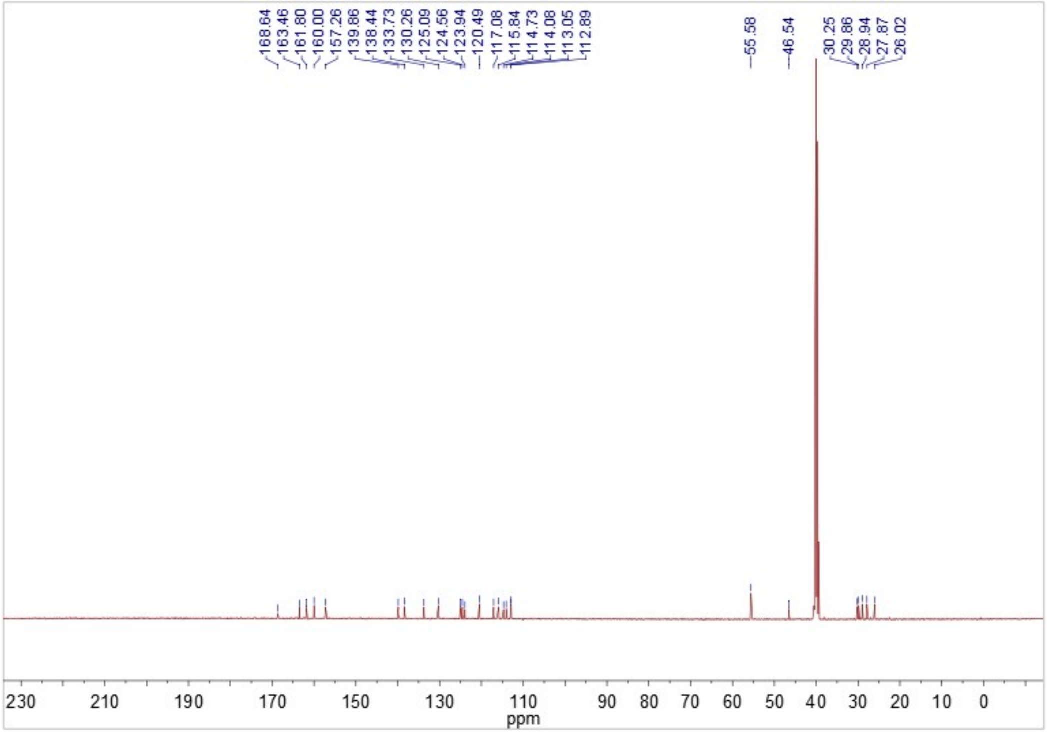

^{13}C NMR Spectrum of **6b**

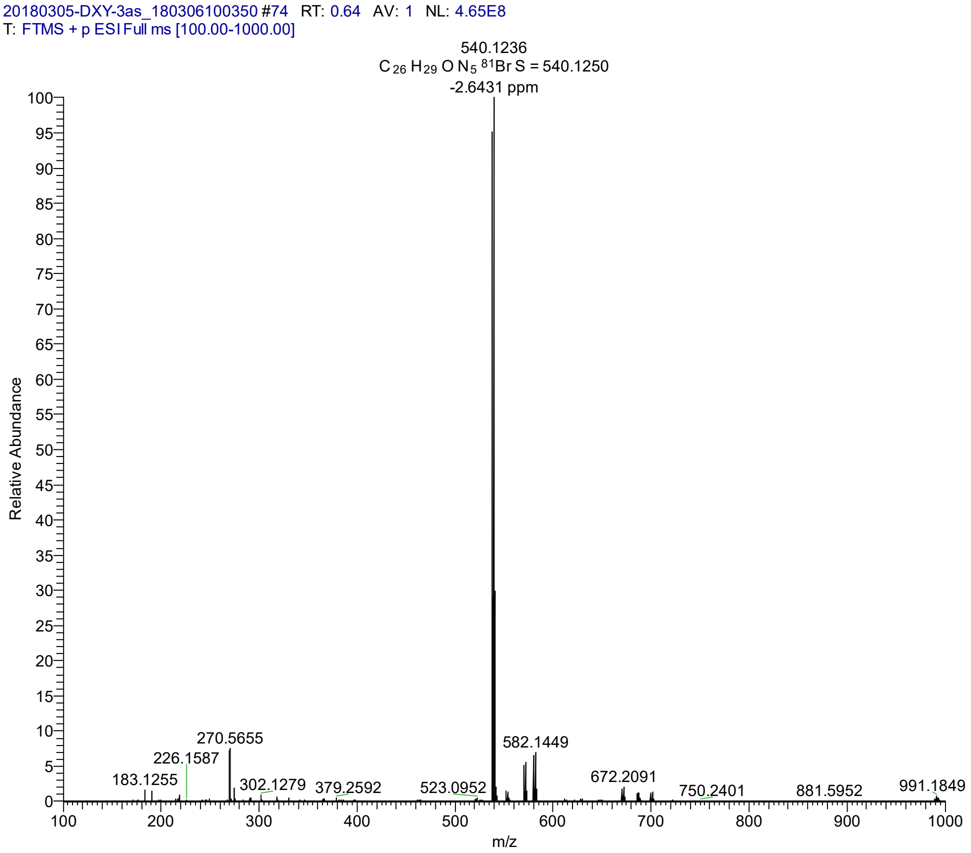

HRMS spectrum of **6b**

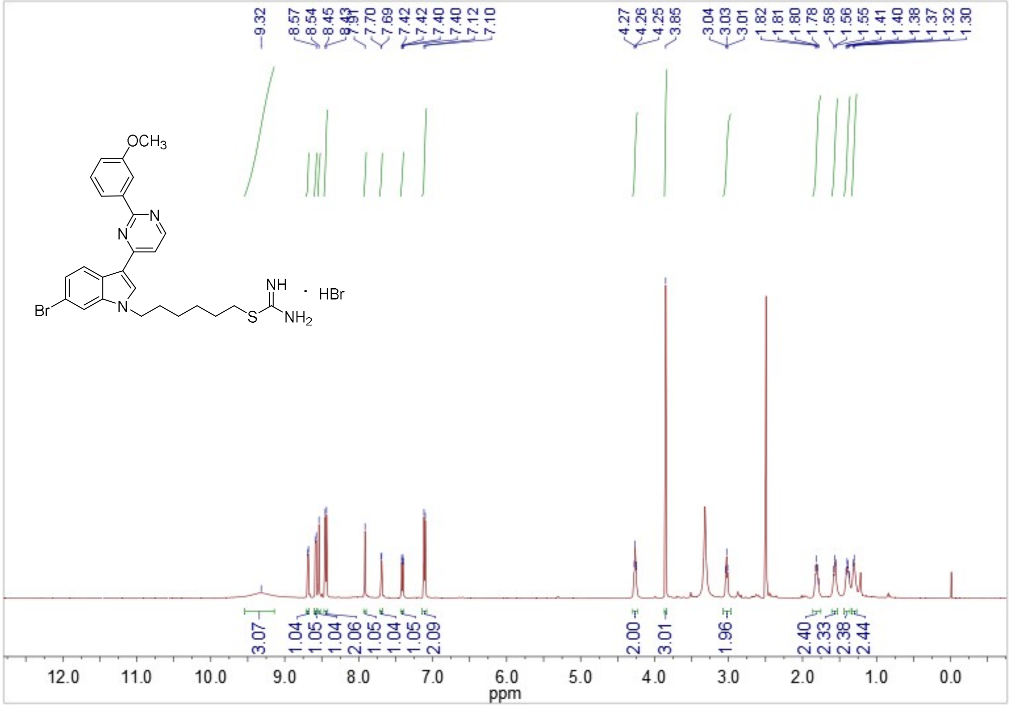

^1H NMR Spectrum of **6c**

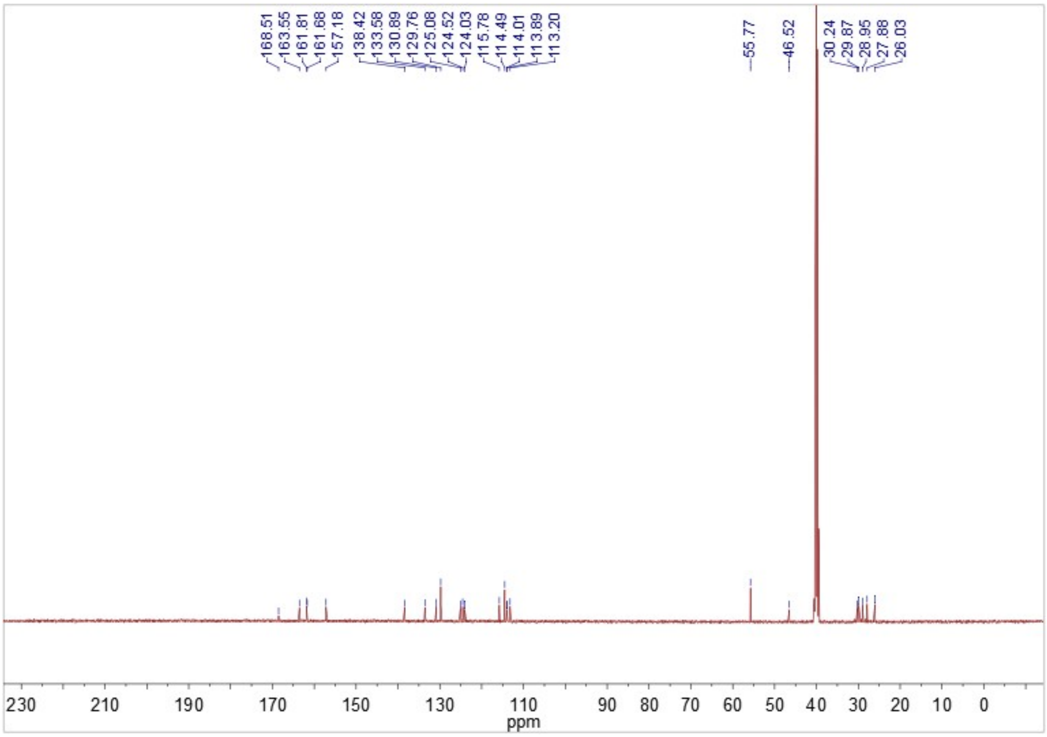

^{13}C NMR Spectrum of **6c**

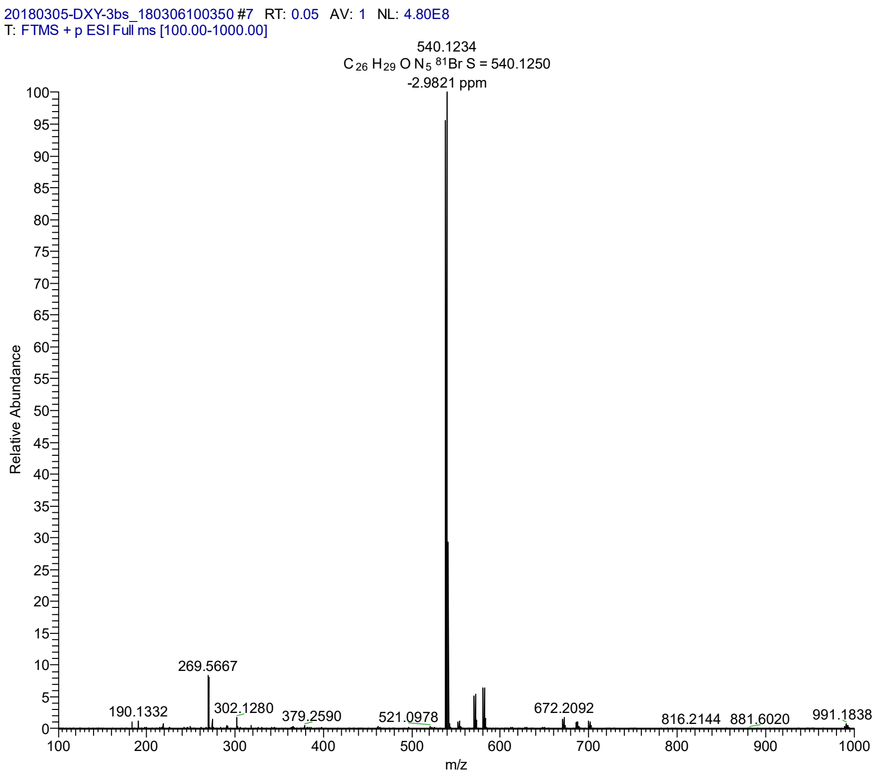

HRMS spectrum of **6c**

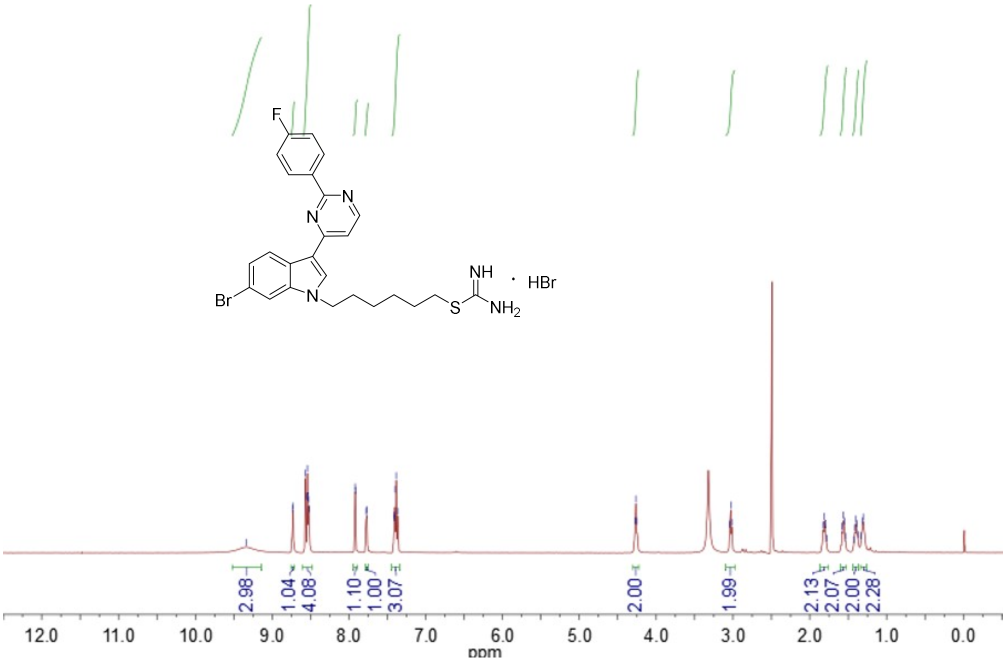

^{1}H NMR Spectrum of **6d**

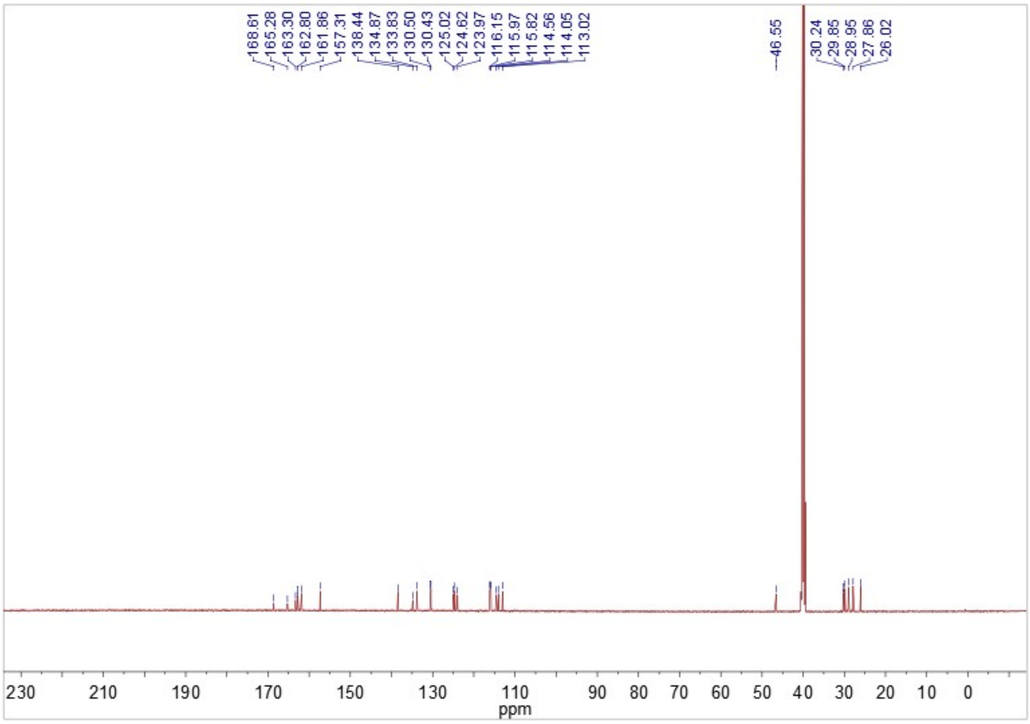

^{13}C NMR Spectrum of **6d**

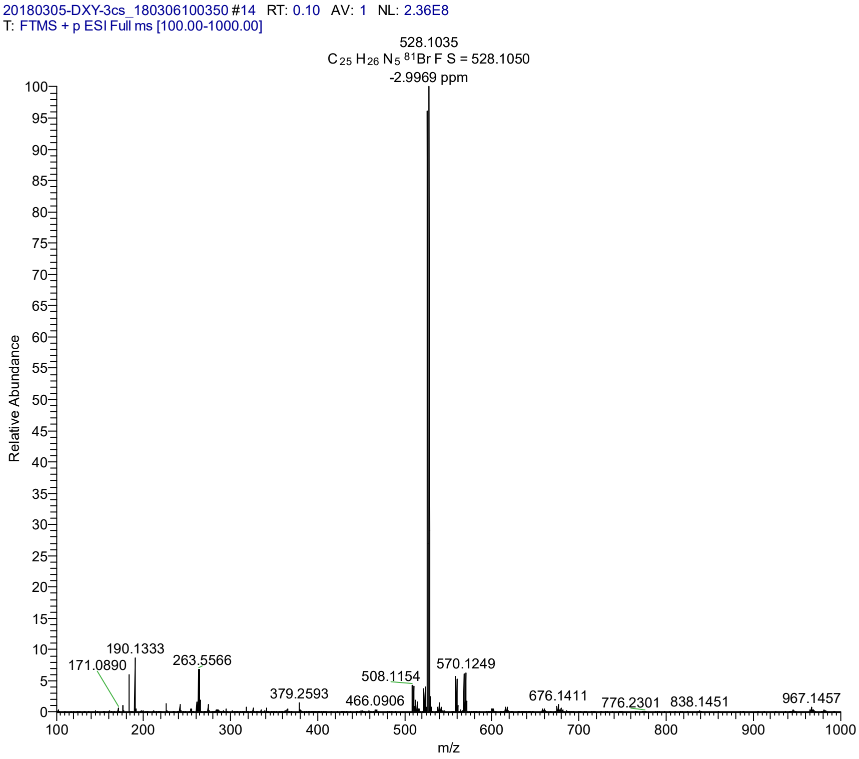

HRMS spectrum of **6d**

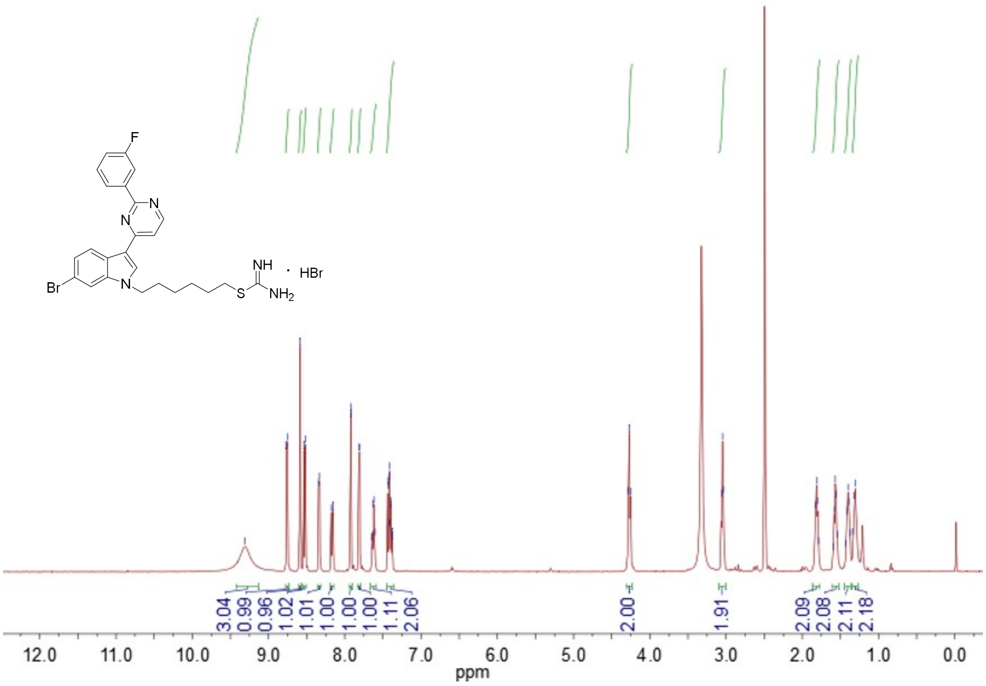

^{1}H NMR Spectrum of **6e**

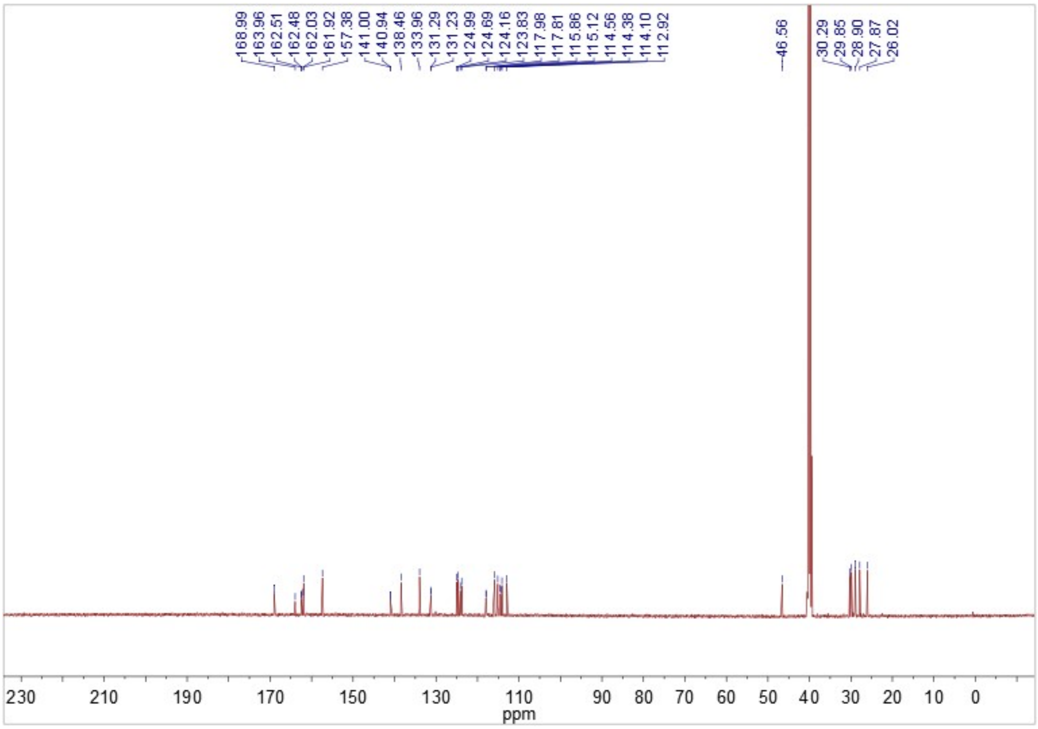

^{13}C NMR Spectrum of **6e**

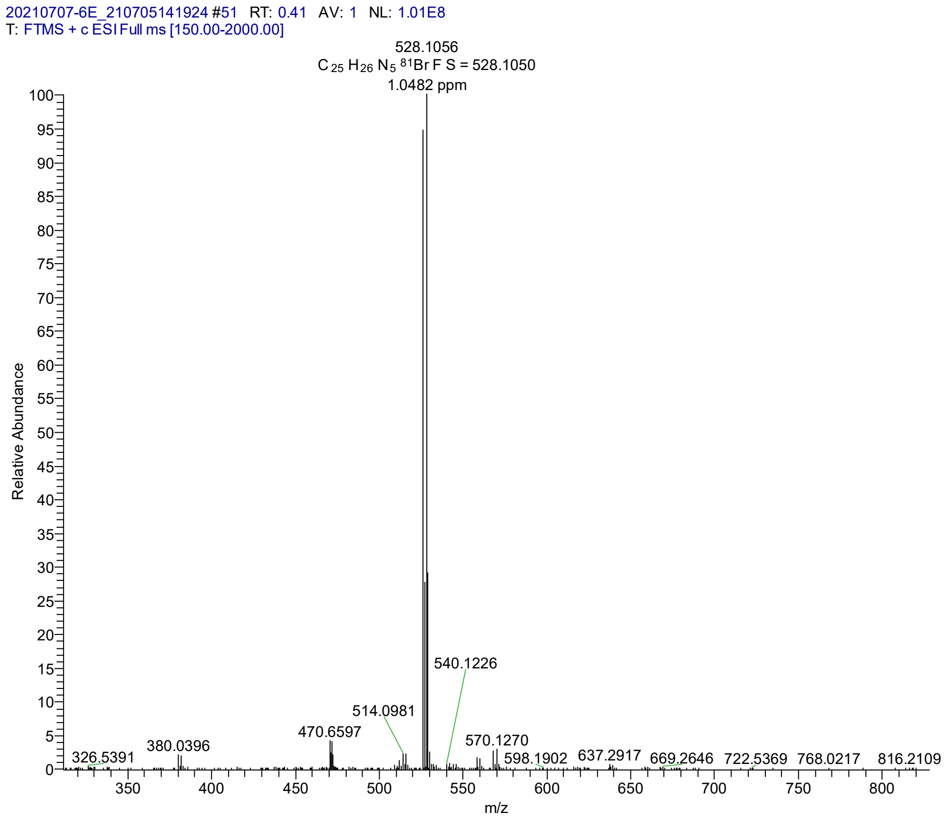

HRMS spectrum of **6e**

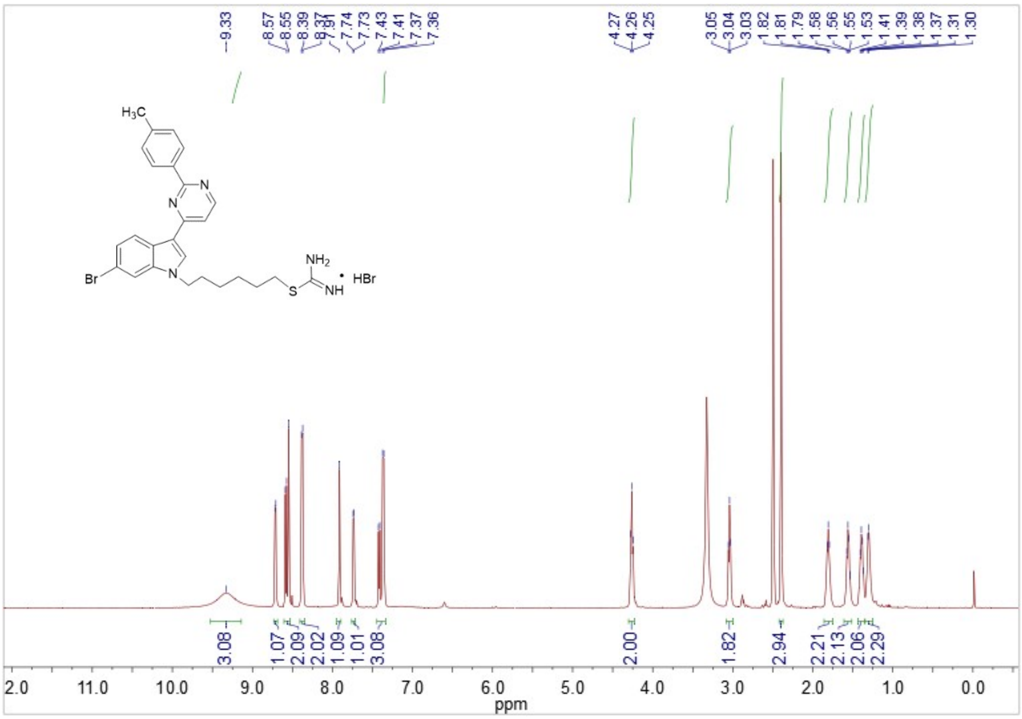

^{1}H NMR Spectrum of **6f**

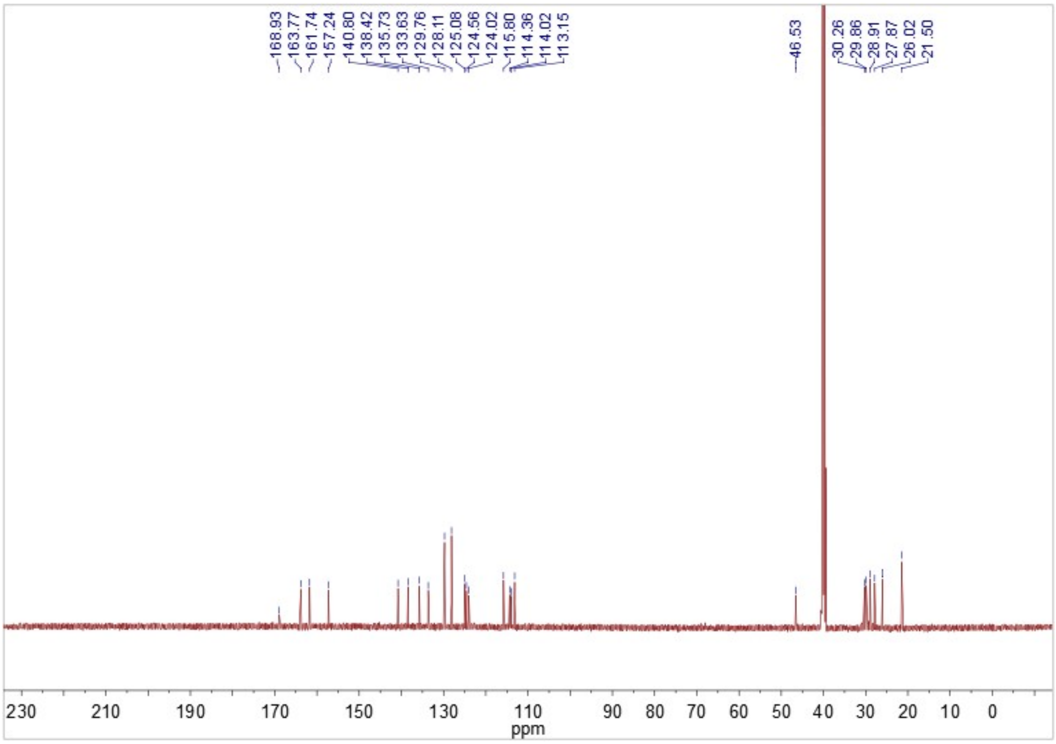

^{13}C NMR Spectrum of **6f**

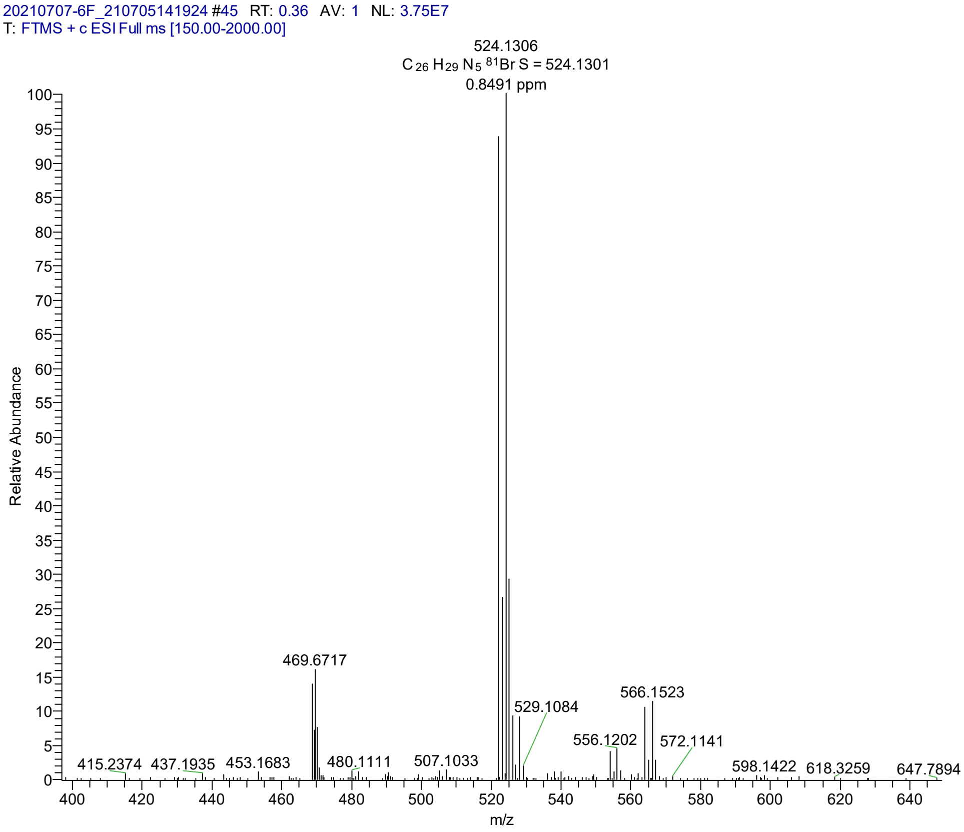

HRMS spectrum of **6f**

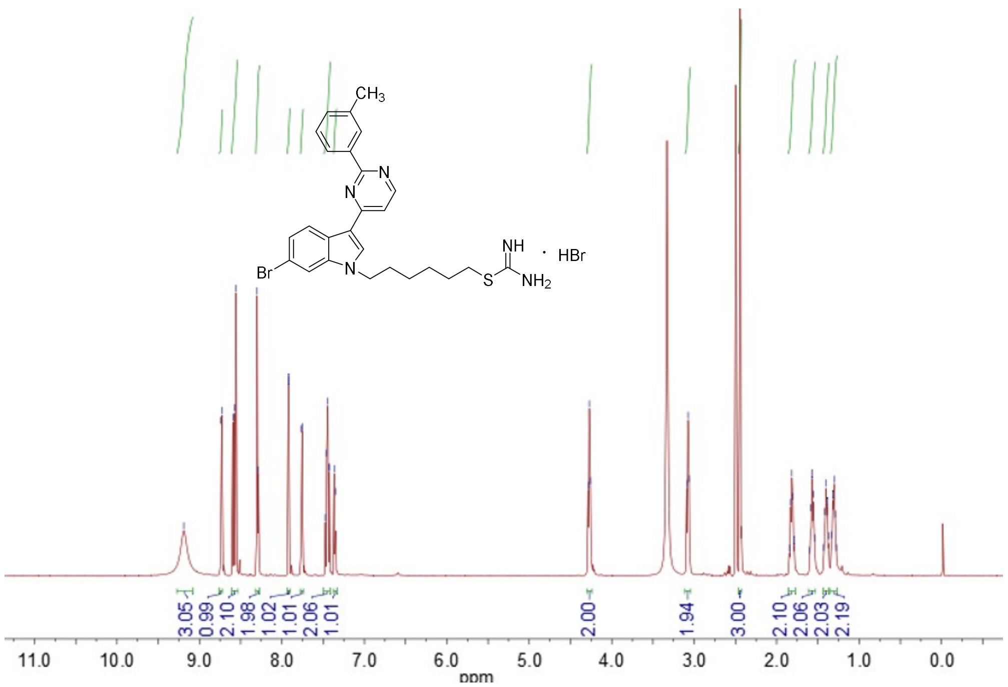

^{1}H NMR Spectrum of **6g**

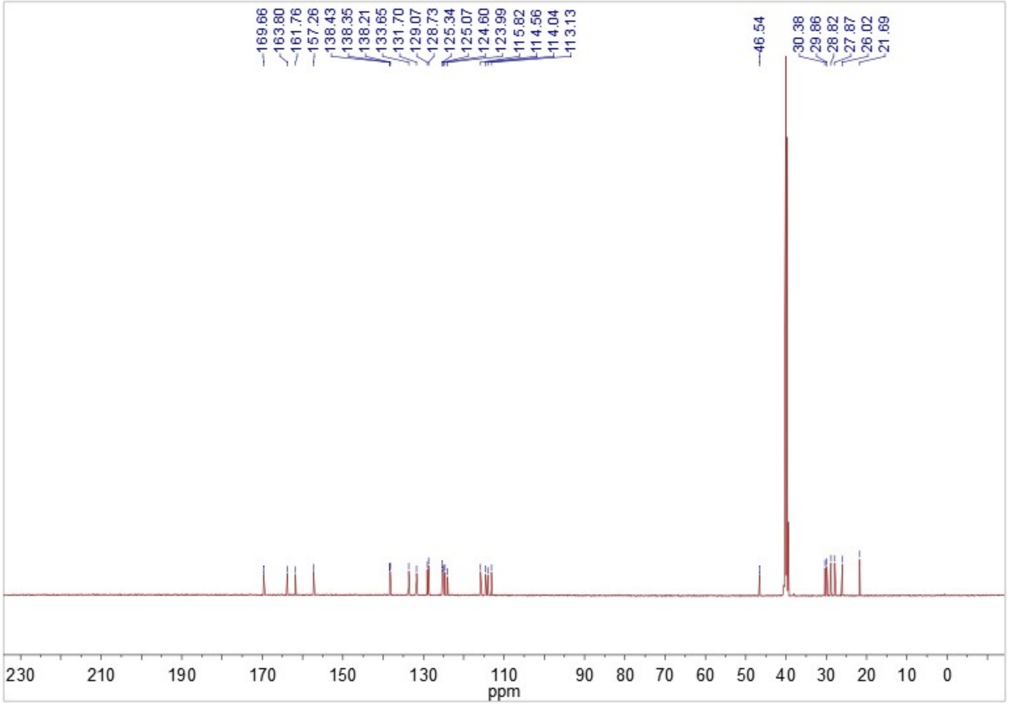

^{13}C NMR Spectrum of **6g**

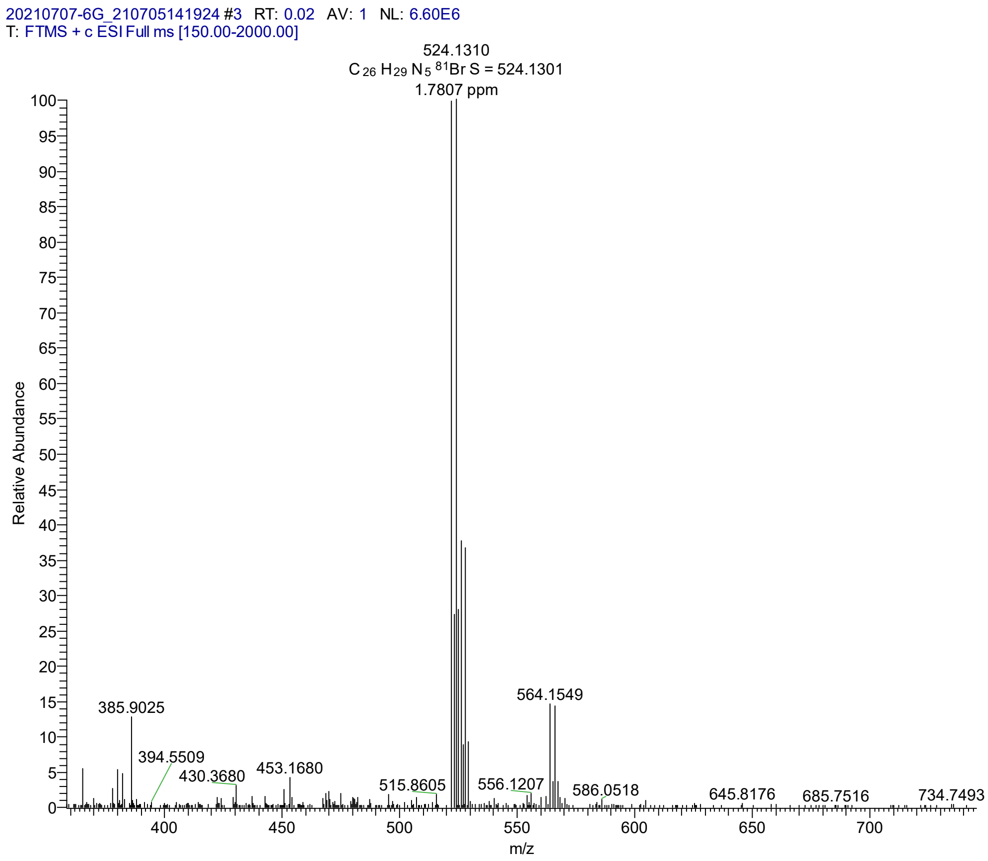

HRMS spectrum of **6g**

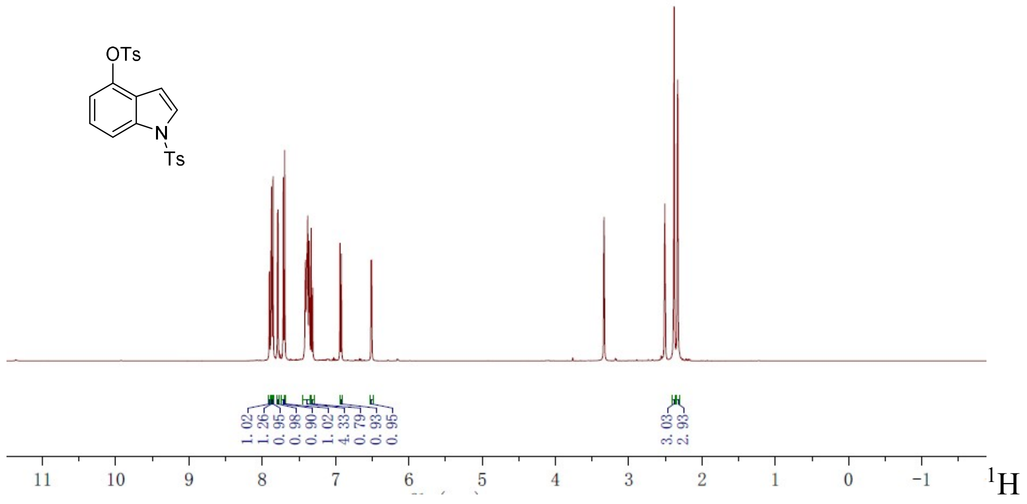

NMR Spectrum of **13**

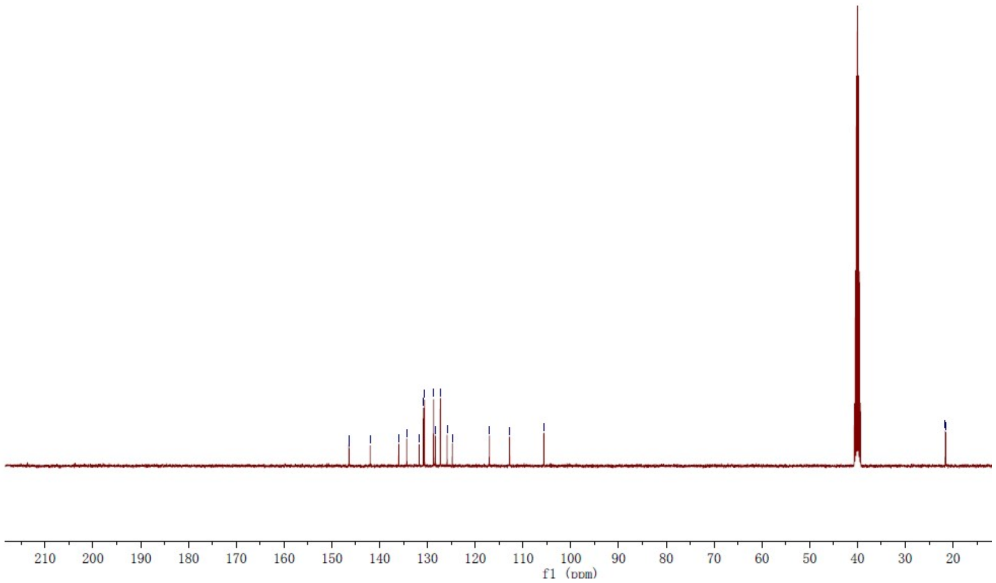

^{13}C NMR Spectrum of **13**

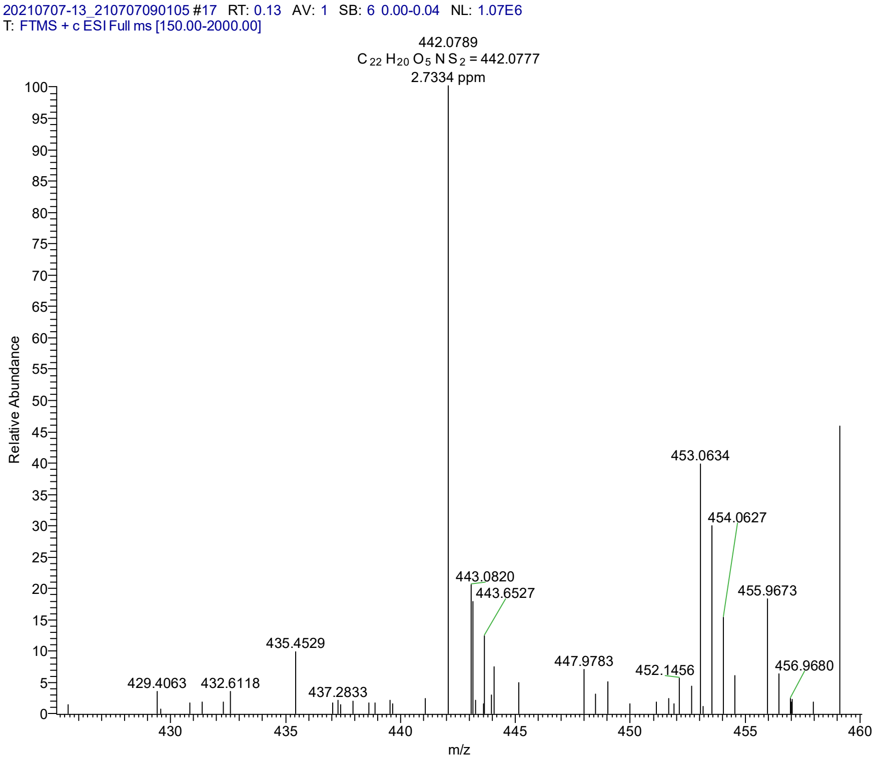

HRMS spectrum of **13**

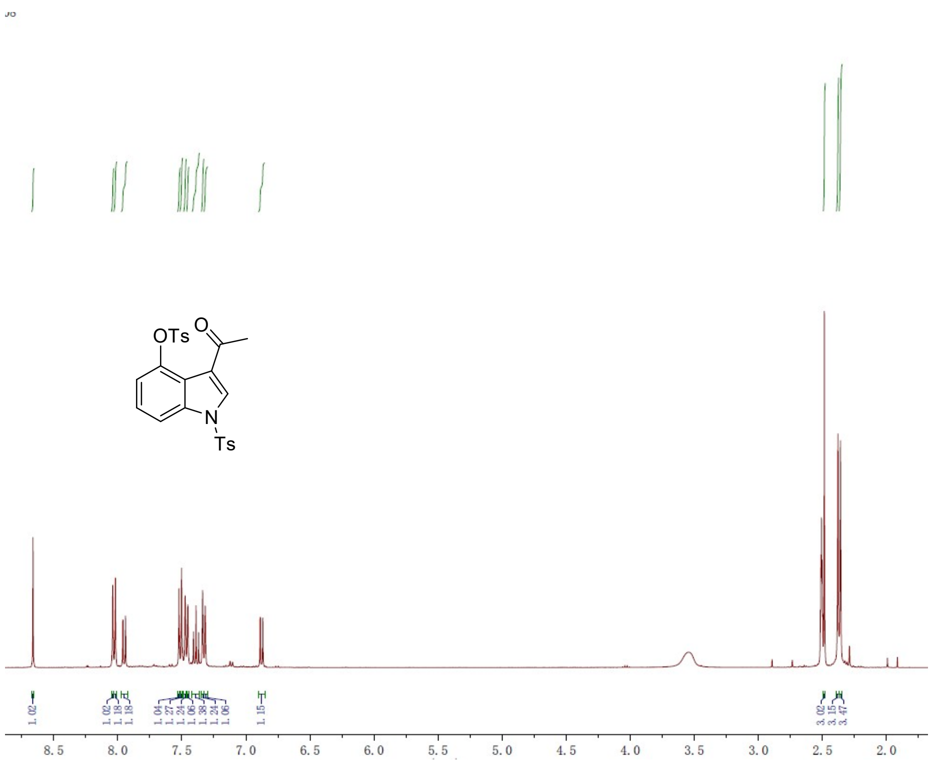

^{1}H NMR Spectrum of **14**

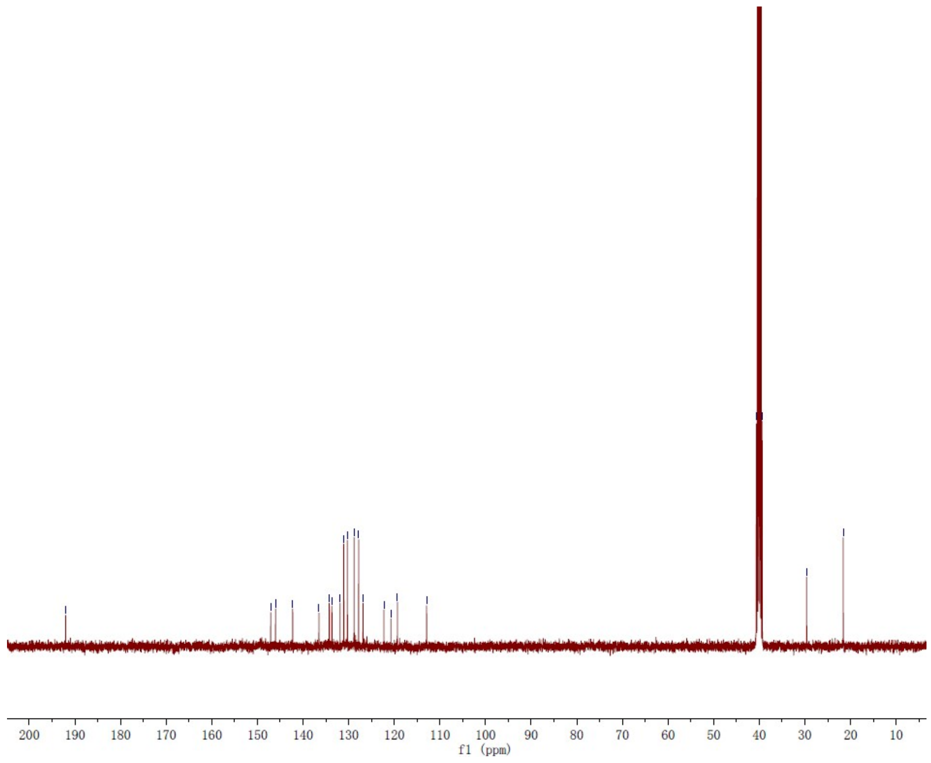

^{13}C NMR Spectrum of **14**

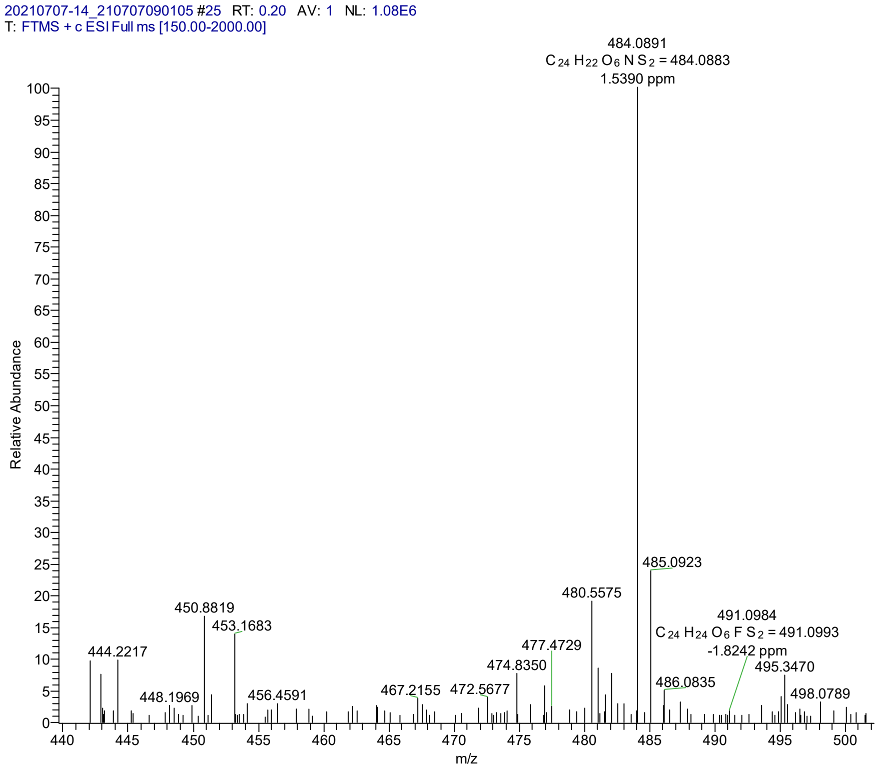

HRMS spectrum of **14**

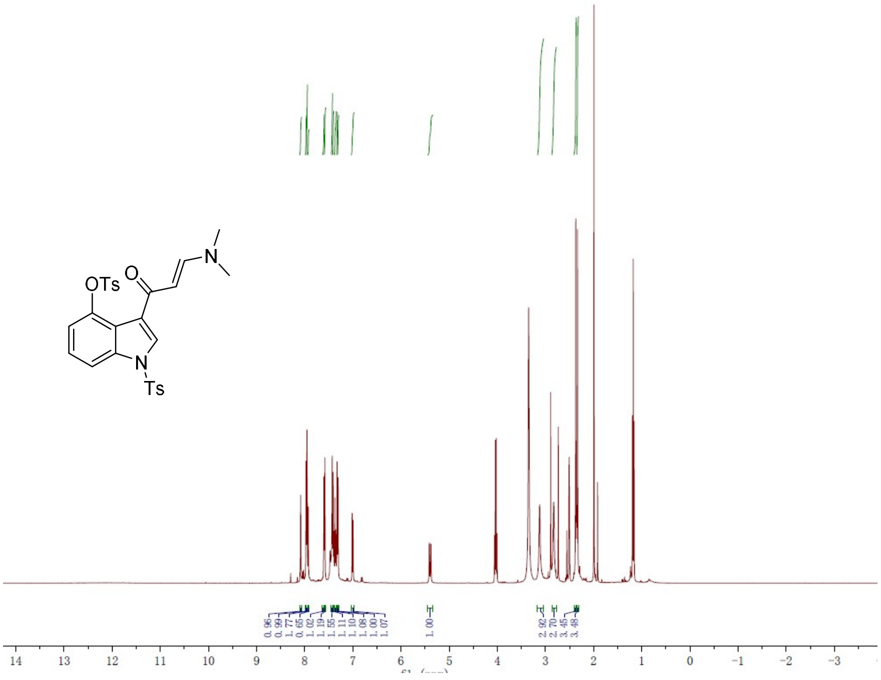

^{1}H NMR Spectrum of **15**

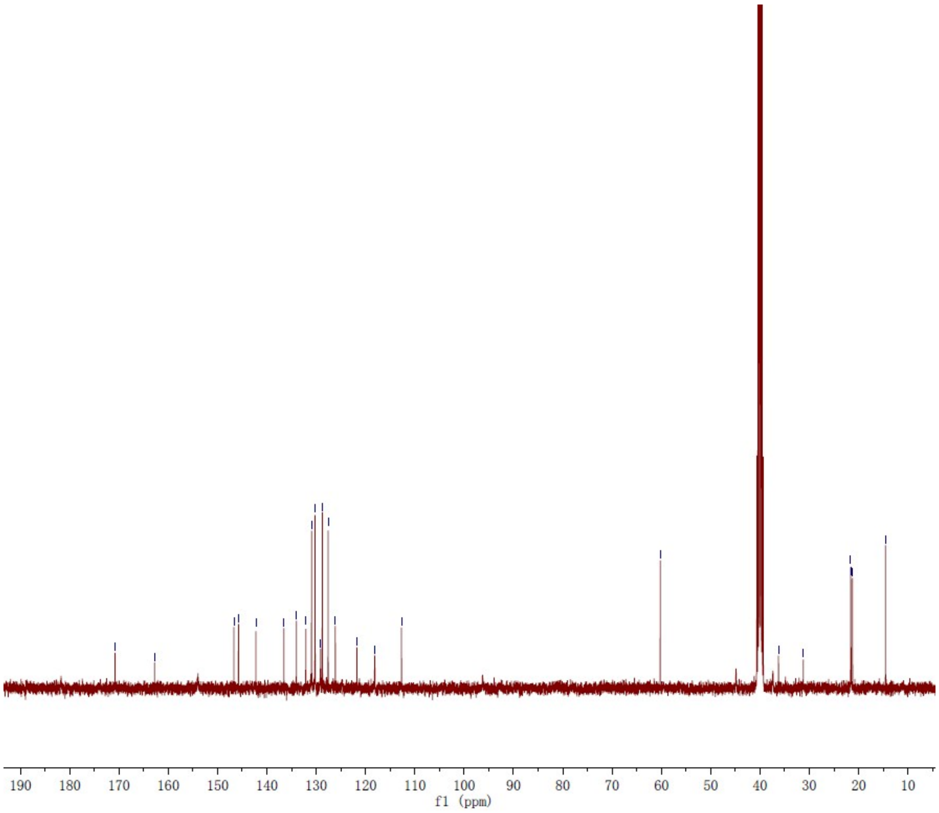

^{13}C NMR Spectrum of **15**

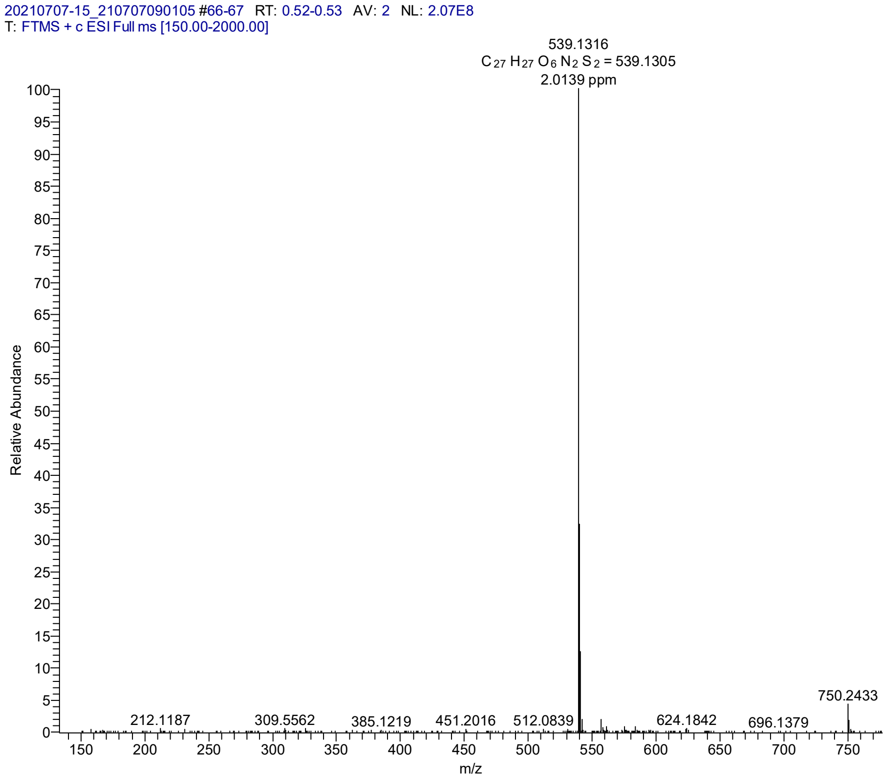

HRMS spectrum of **15**

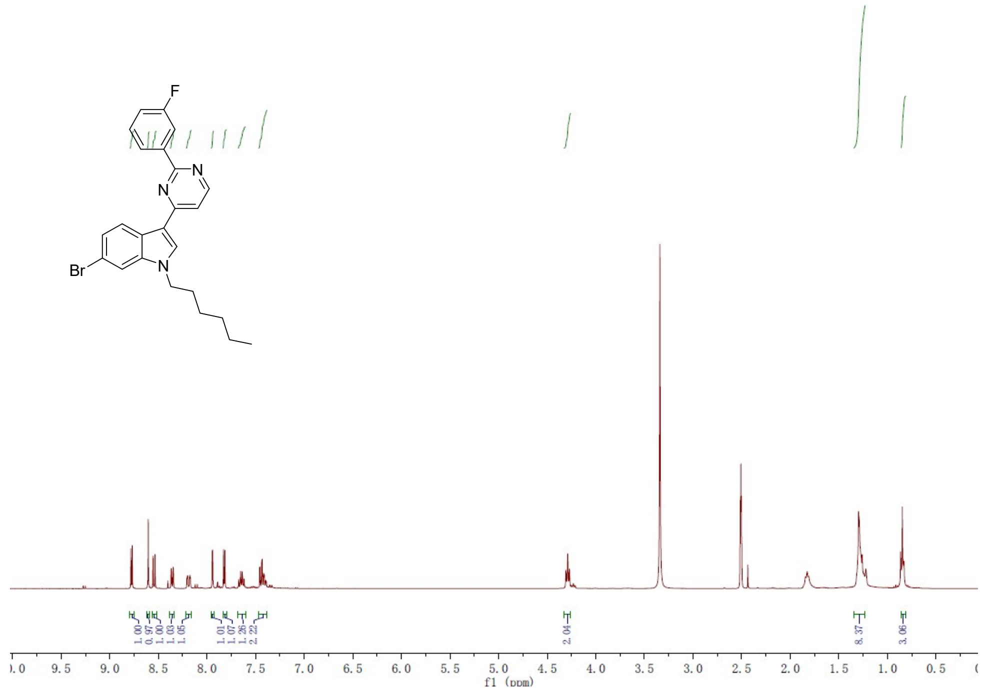

^{1}H NMR Spectrum of **6e-1**

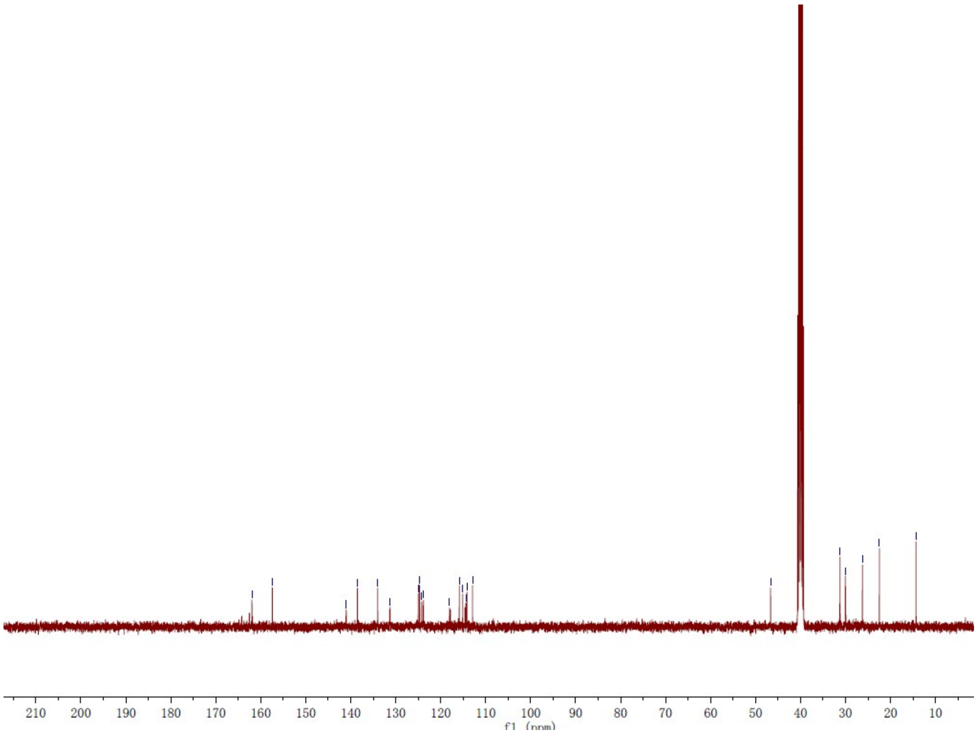

^{13}C NMR Spectrum of **6e-1**

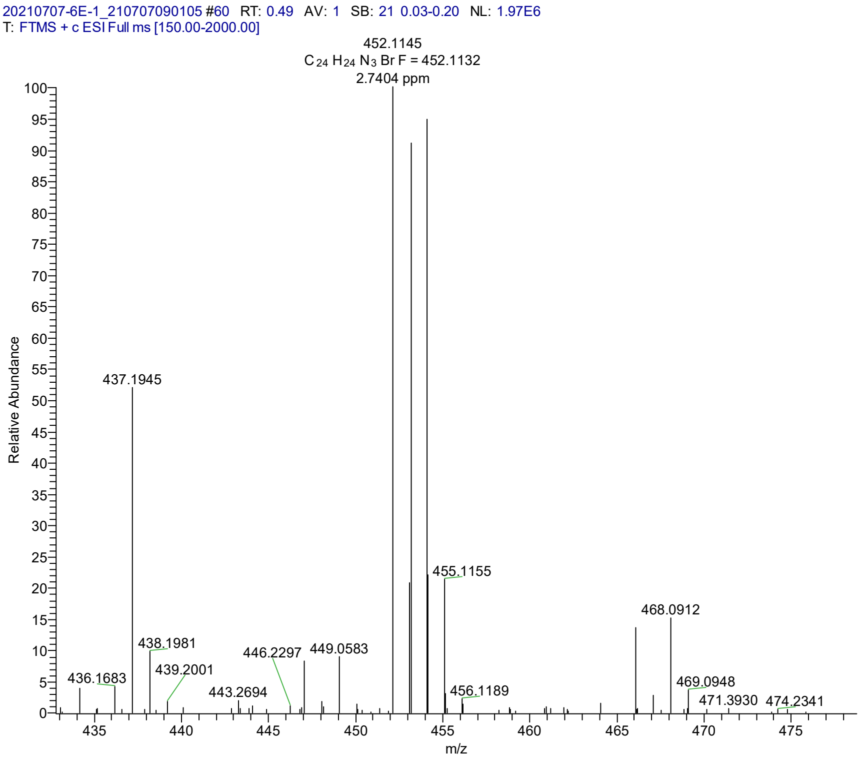

HRMS spectrum of **6e-1**

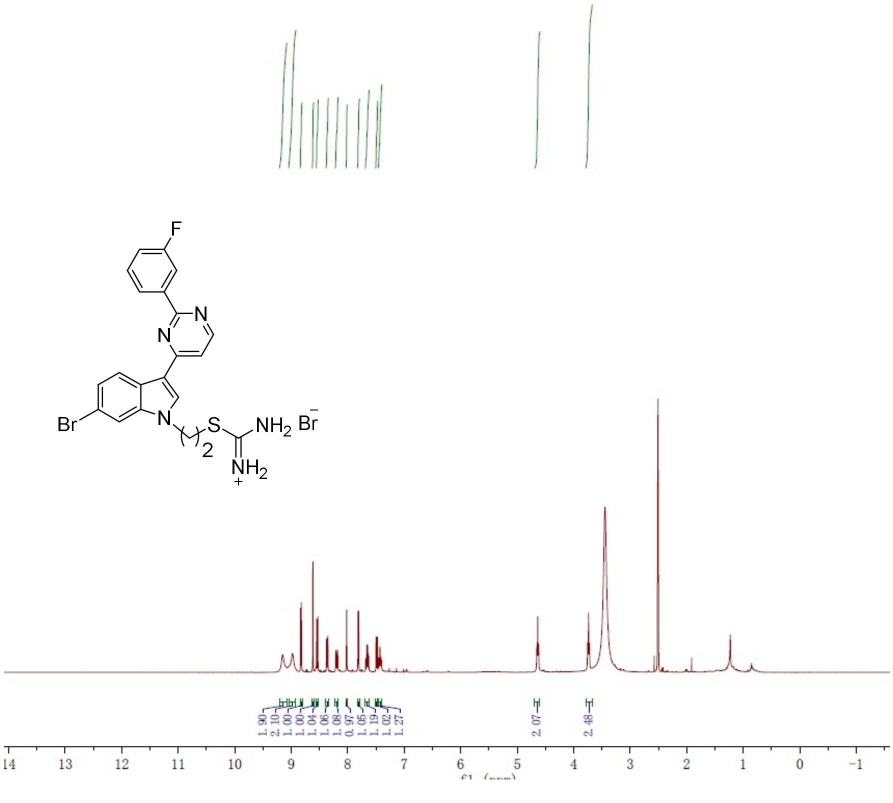

^{1}H NMR Spectrum of **6e-2**

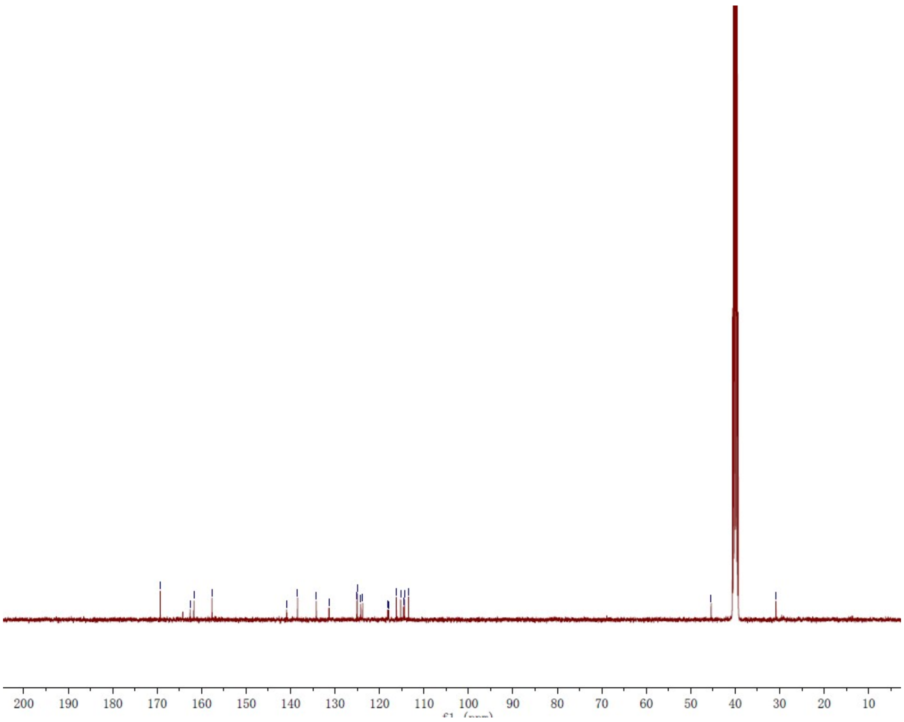

^{13}C NMR Spectrum of **6e-2**

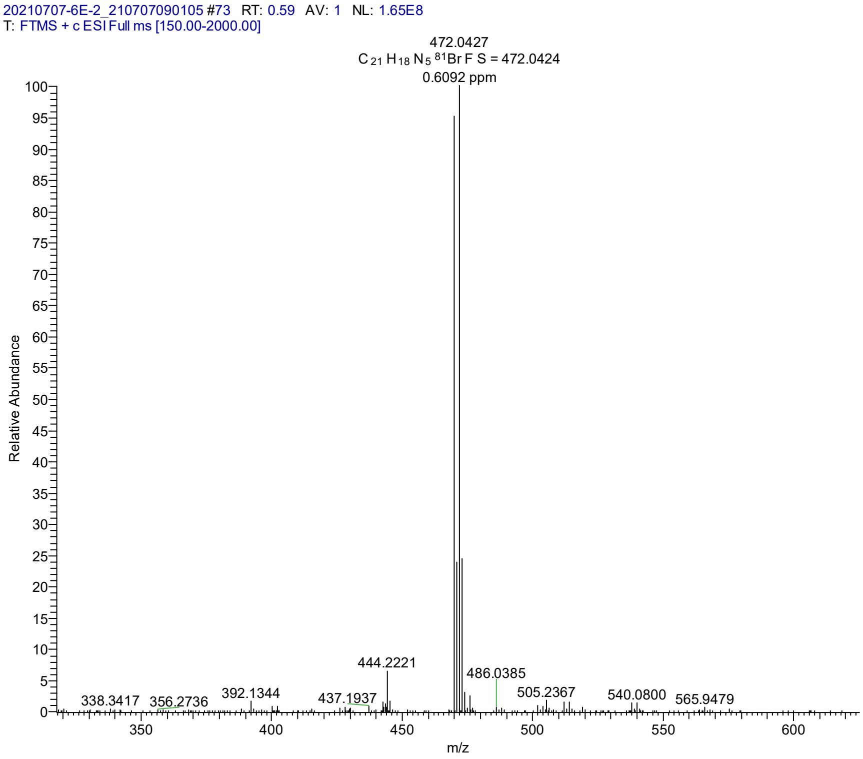

HRMS spectrum of **6e-2**

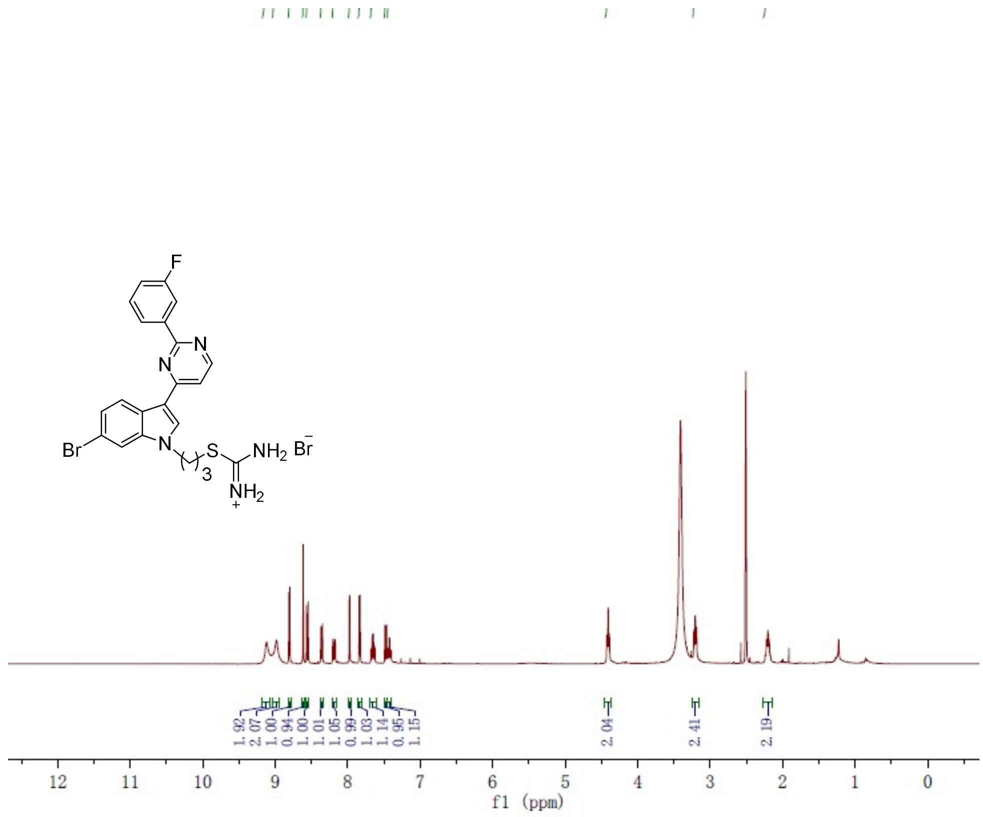

^{1}H NMR Spectrum of **6e-3**

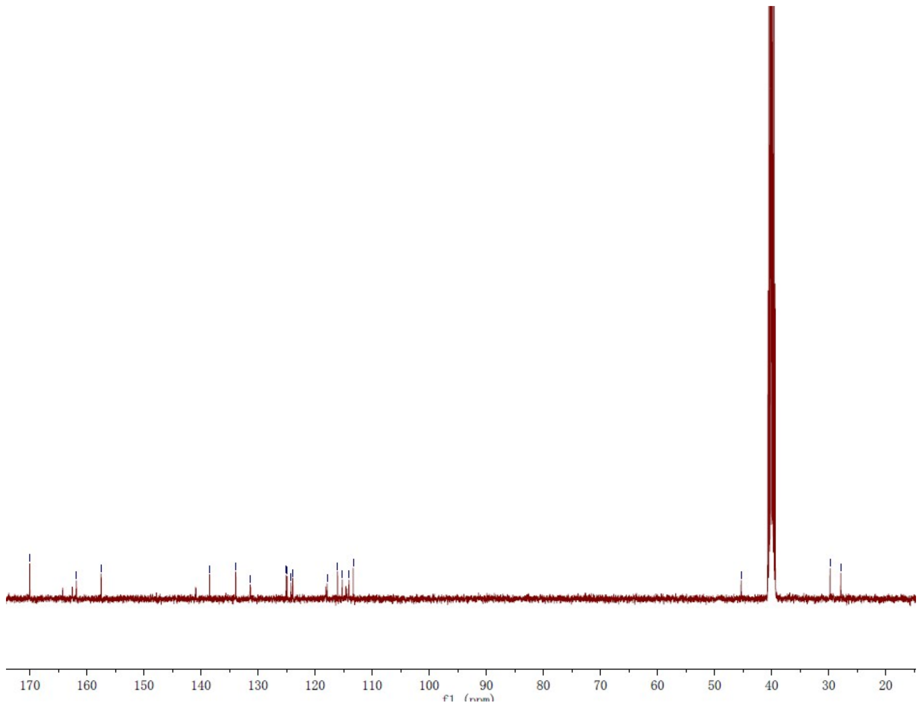

^{13}C NMR Spectrum of **6e-3**

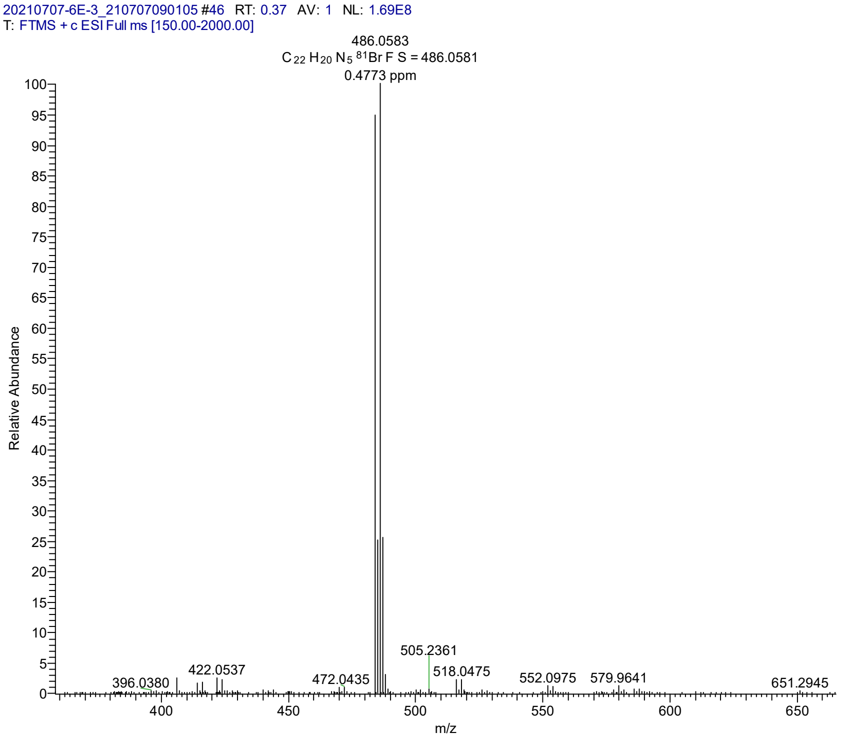

HRMS spectrum of **6e-3**

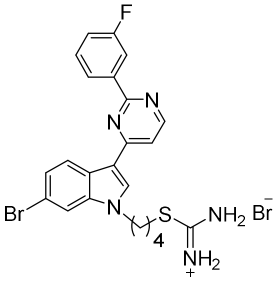

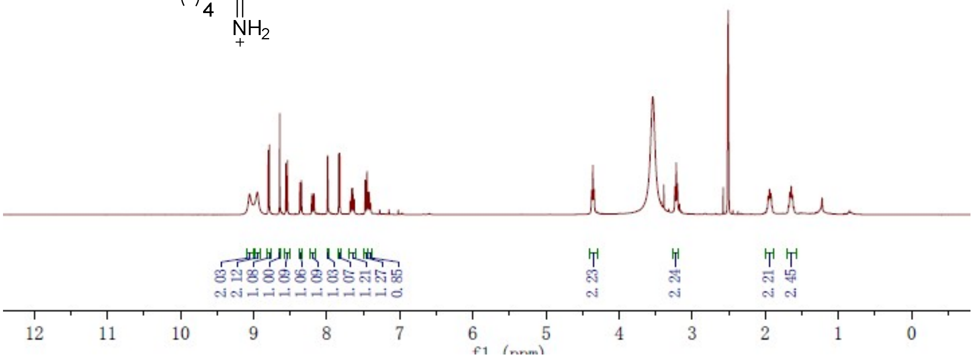

^{1}H NMR Spectrum of **6e-4**

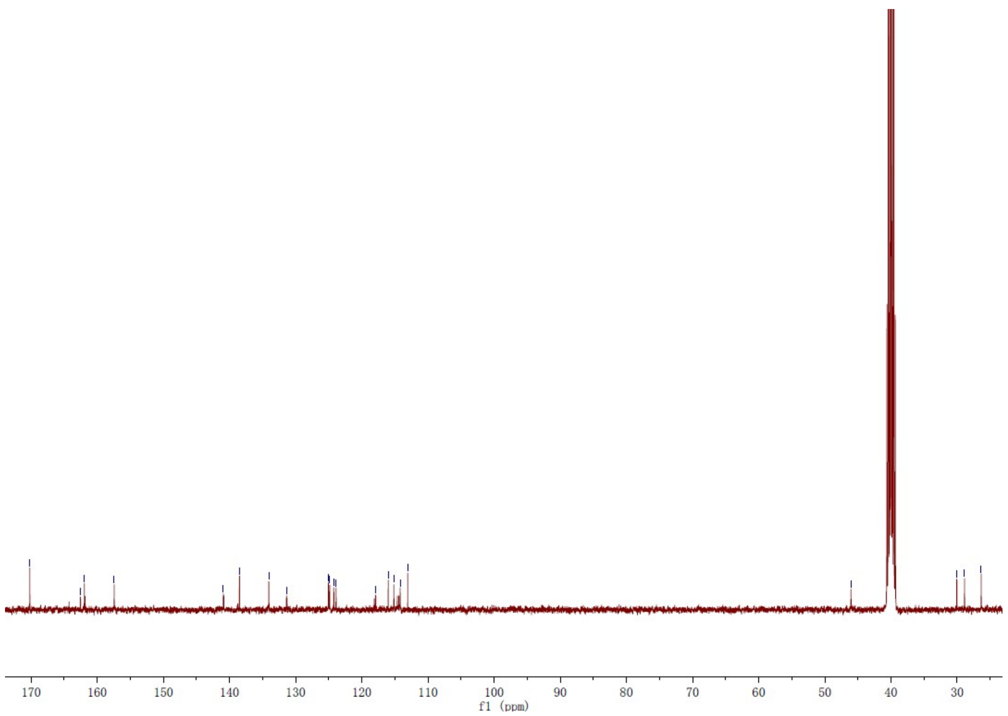

^{13}C NMR Spectrum of **6e-4**

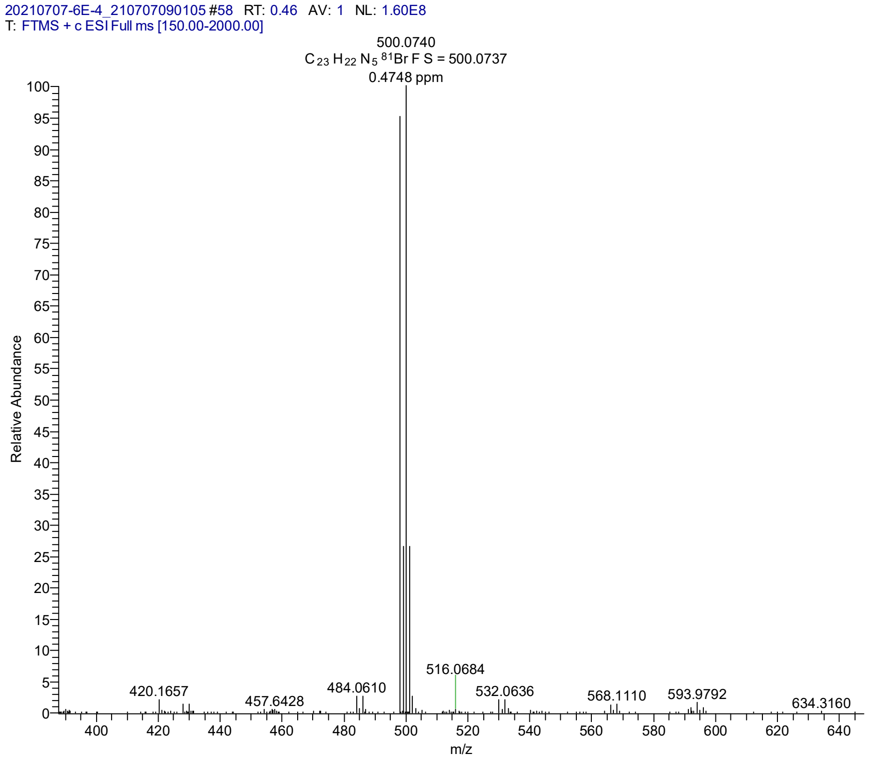

HRMS spectrum of **6e-4**

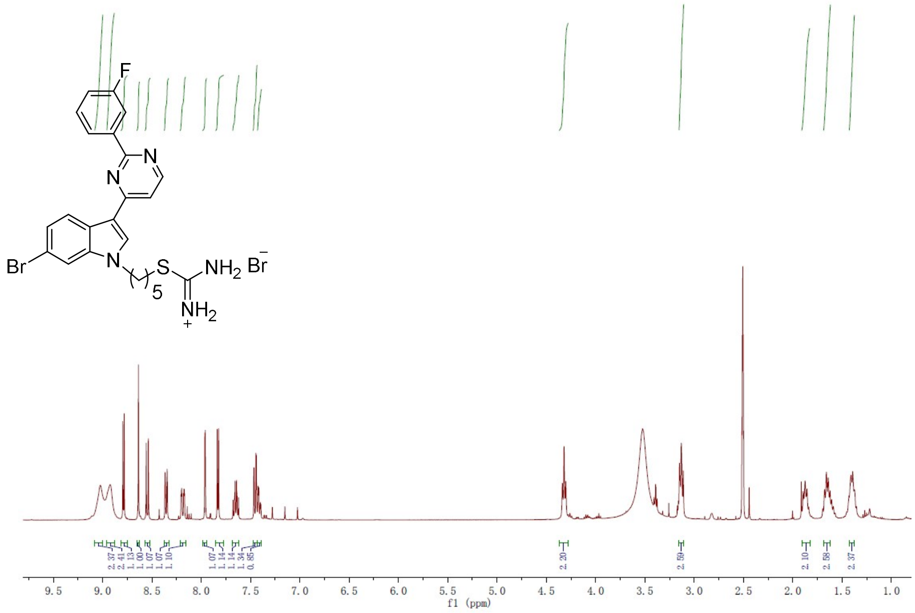

^{1}H NMR Spectrum of **6e-5**

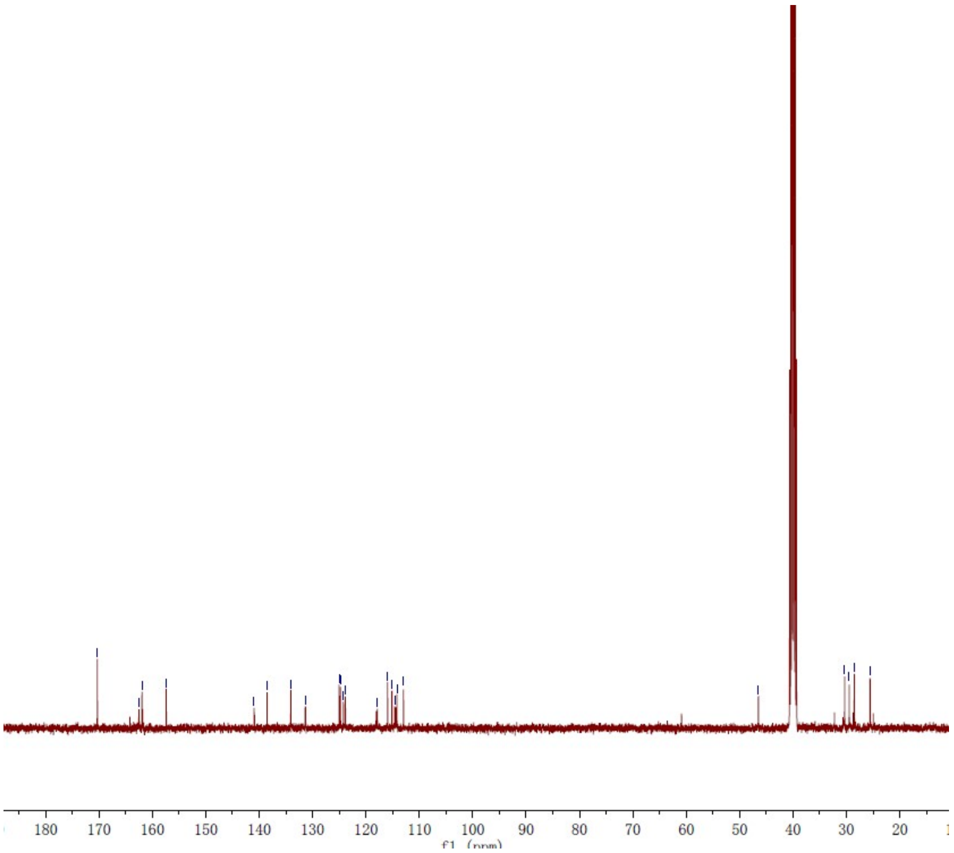

^{13}C NMR Spectrum of **6e-5**

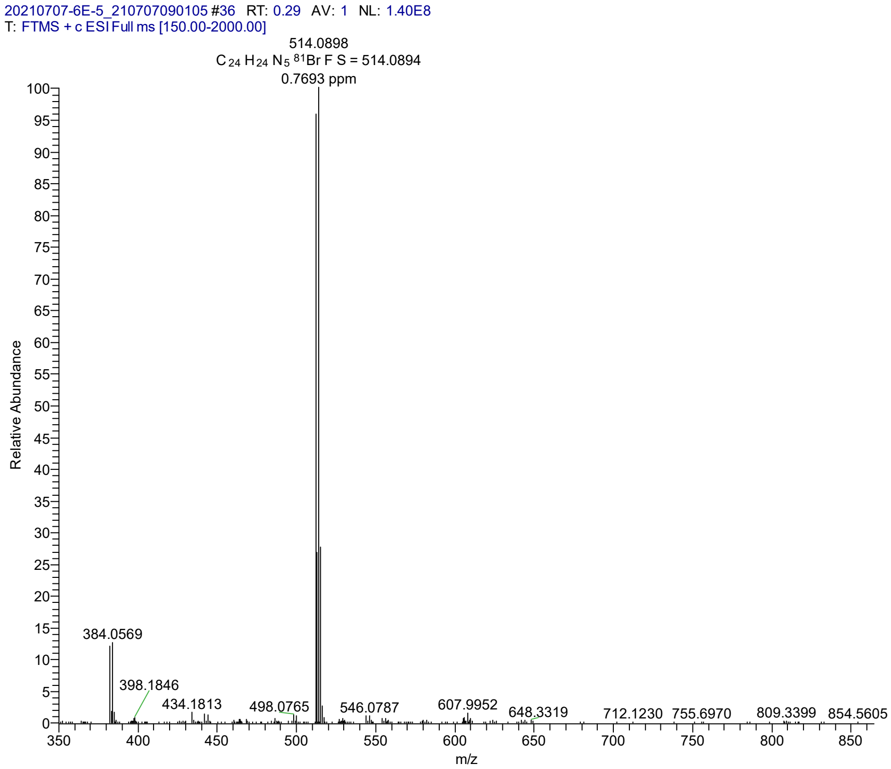

HRMS spectrum of **6e-5**

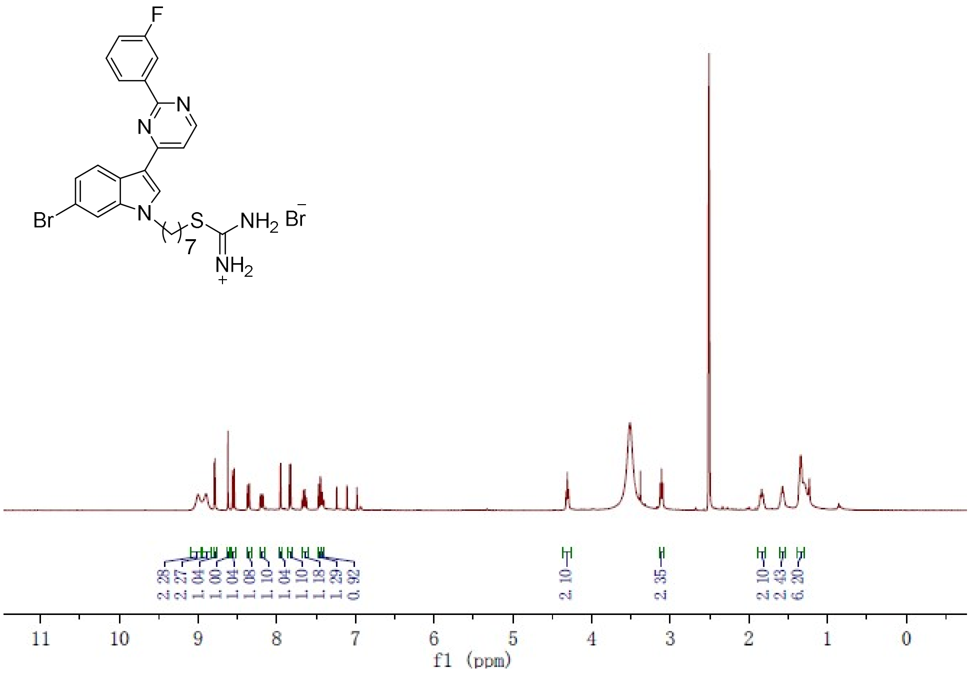

^{1}H NMR Spectrum of **6e-6**

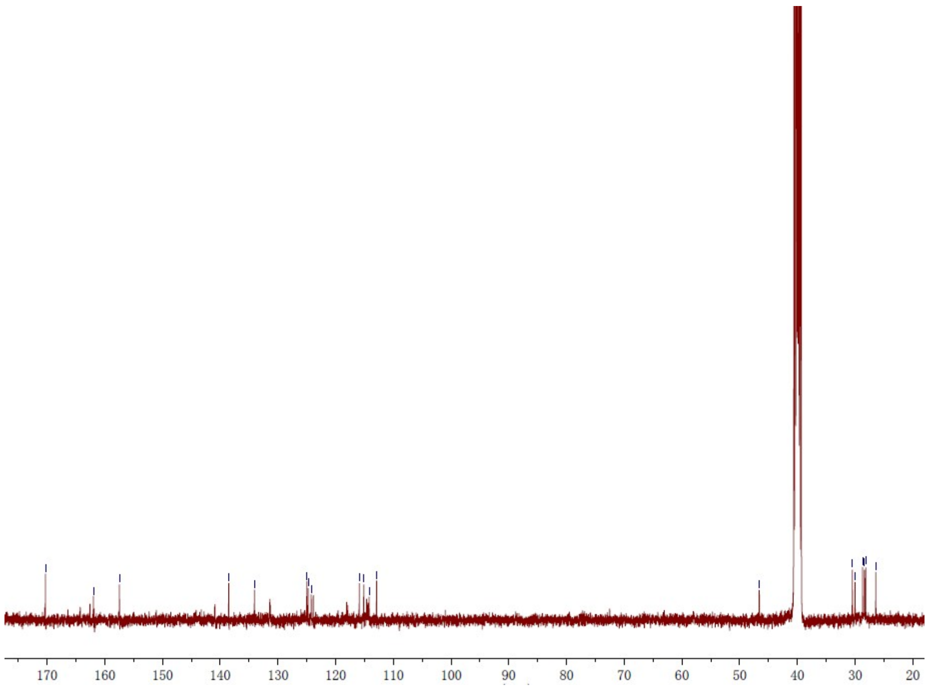

^{13}C NMR Spectrum of **6e-6**

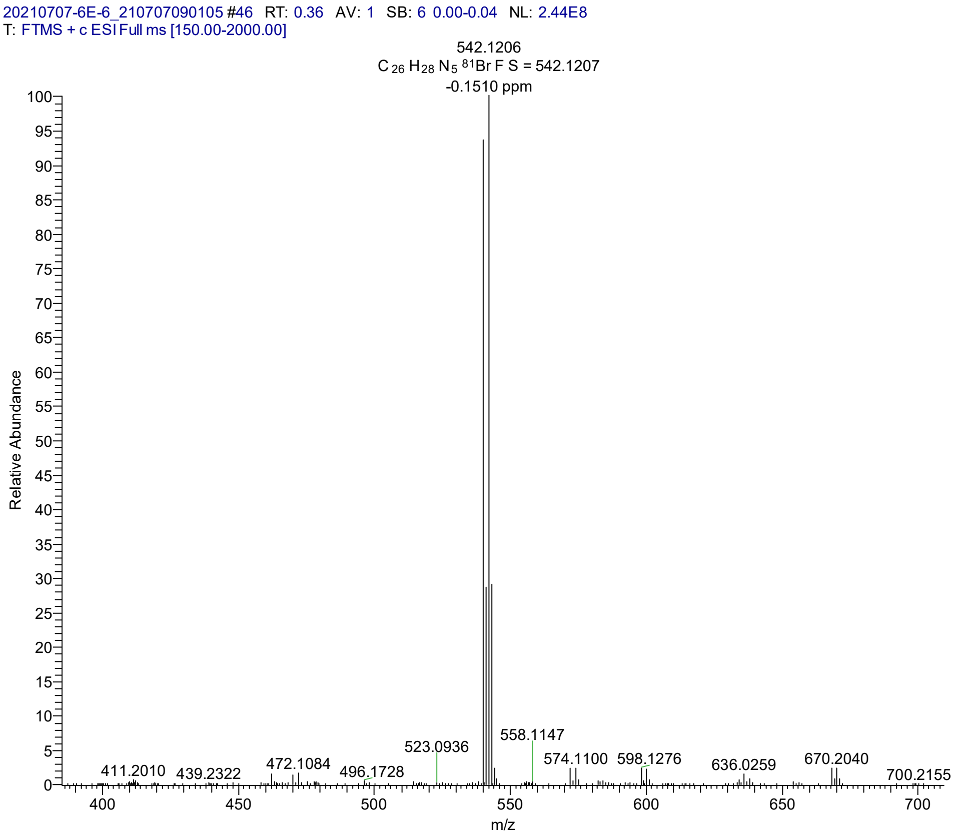

HRMS spectrum of **6e-6**
